# Discovery of 2-phenylethyl chromones as potent and selective CYP1B1 inhibitors

**DOI:** 10.1080/14756366.2025.2598738

**Published:** 2026-01-02

**Authors:** Wenming Chen, Wenchong Ye, Yinghong Long, Ye Zhang, Wen Zhou, Wei Wang

**Affiliations:** ^a^ TCM and Ethnomedicine Innovation & Development International Laboratory, Innovative Materia Medica Research Institute, School of Pharmacy, Hunan University of Chinese Medicine, Changsha, Hunan, P.R. China; ^b^ Department of Pharmaceutical Products Center, The First Hospital of Hunan University of Chinese Medicine, Changsha, Hunan, P.R. China; ^c^ Shanghai Veterinary Research Institute, Chinese Academy of Agricultural Sciences, Shanghai, P.R. China; ^d^ China Key Laboratory of Veterinary Chemical Drugs and Pharmaceutics, Ministry of Agriculture and Rural Affairs, Shanghai Veterinary Research Institute, Chinese Academy of Agricultural Sciences, Shanghai, P.R. China; ^e^ Department of Chinese Medicine, Shenzhen Futian District Maternal and Child Health Hospital, Shenzhen, Guangdong, P.R. China

**Keywords:** Chromone derivatives, synthesis, CYP1B1, selectivity, resistance reversal

## Abstract

Cytochrome P4501B1 (CYP1B1), overexpressed in solid tumours but minimally in healthy tissues, is a promising anticancer target linked to chemoresistance. While CYP1B1 inhibitors can restore drug efficacy, most suffer from limited scaffold diversity and poor selectivity against other CYPs. We identified 2-(2-phenylethyl) chromones as a novel scaffold for anti-CYP1B1 activity and synthesised 24 derivatives with varied ring A/B substituents and established the SAR. Three compounds (**CX-6**, **CX-9**, **CX-22**) showed nanomolar anti-CYP1B1 activity and exceptional selectivity (SI > 230). In CYP1B1-overexpressing cells, the water-soluble and non-cytotoxic **CX-9** (solubility > 100 μM) dose-dependently reversed docetaxel resistance, achieving efficacy at 50 μM comparable to 20 μM of the CYP1B1 inhibitor α-naphthoflavone (ANF). Molecular docking revealed similar binding modes for **CX-9** and ANF in CYP1B1’s active site. This work hints 2-(2-phenylethyl) chromones as a natural-derived scaffold for promising CYP1B1 inhibitor development.

## Graphical Abstract

**Figure:**
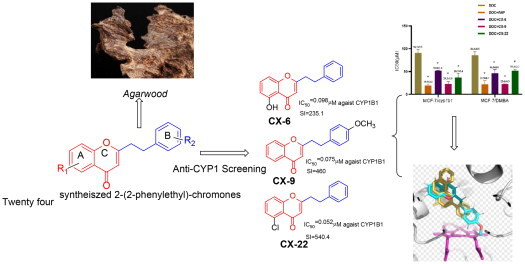

## Introduction

Cytochrome P450 (CYP)1B1, a cancer-specific biomarker, is overexpressed in various malignancies while remaining undetectable in surrounding normal tissues^1^. As a key extrahepatic cytochrome, CYP1B1 plays a well-characterised role in metabolising both exogenous (e.g. polycyclic aromatic hydrocarbons, halogenated aromatic hydrocarbons, and anticancer drugs) and endogenous compounds (e.g. oestradiol conversion to carcinogenic derivatives)^2^. Notably, CYP1B1-mediated metabolism of clinical therapeutics such as docetaxel, paclitaxel, and cyclophosphamide contributes to drug resistance, often resulting in treatment failure^3^. These functional properties underscore CYP1B1’s dual potential as a therapeutic target for cancer treatment^4^ and a preventive target for carcinogenesis.^6^ Consequently, the development of novel, potent CYP1B1 inhibitors has garnered significant research interest.

**Figure 1.**
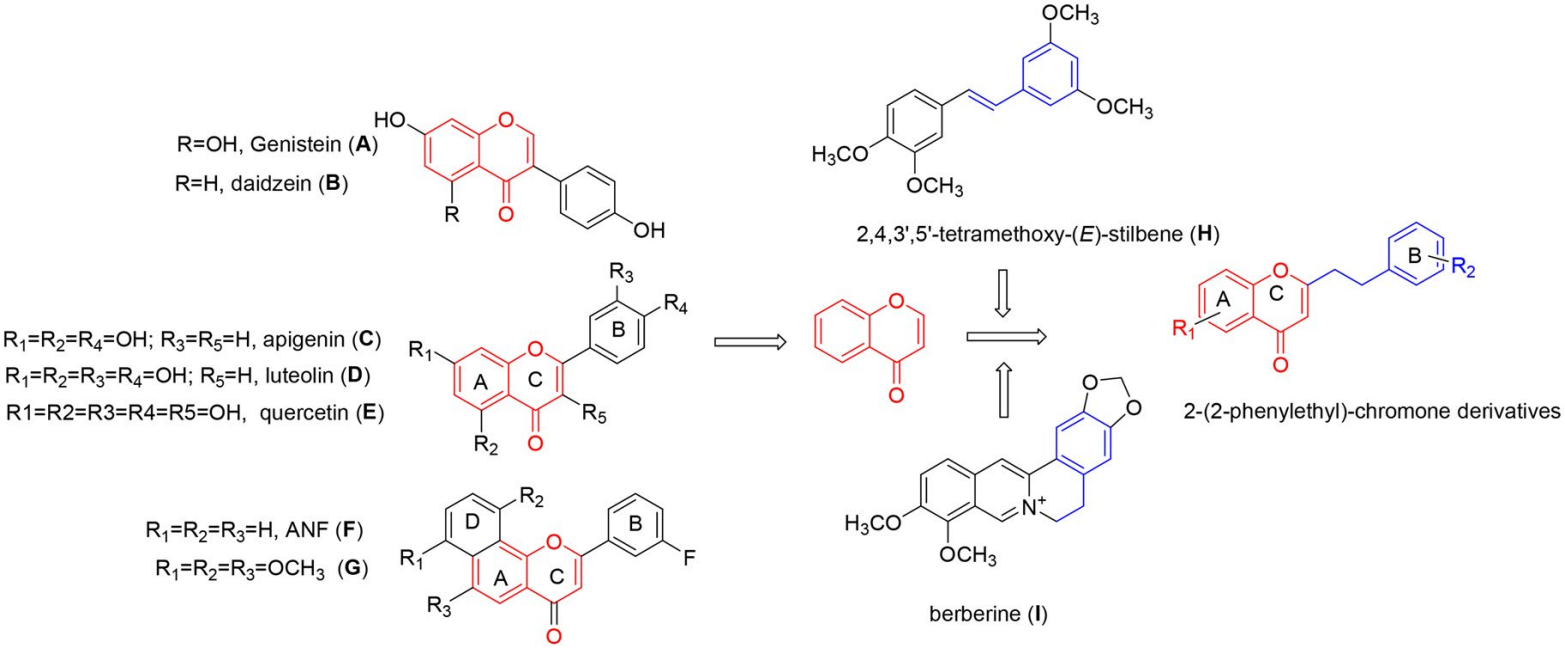
Design of 2–(2-phenylethyl)-chromone derivatives for CYP1B1 inhibitors.

Natural products are a main pathway in the drug discovery. Genistein (**A,** Figure 1) and daidzein (**B**) exhibited anticancer effects in early-stage prostate cancer,^5^ while high intake of soybean rich in phytoestrogens correlated with reduced prostate cancer incidence in Asia^6^. Their mechanisms may involve CYP450 modulation^7^ and suppression of tumour promoters^8^. Flavonoids also impacted breast cancer via antiestrogenic or aromatase-inhibitory effects^9^. Notably, flavonoid-based CYP1B1 inhibitors including apigenin (**C**), luteolin (**D**), and quercetin (**E**)^10^, demonstrated potent activity with IC_50_ values in nM range,^11^ outperforming flavanones with those in mM range. Flavones and flavonols like 3-hydroxyflavones showed stronger CYP1B1 inhibition, further supported by α-naphthoflavone (ANF, **F**, IC_50_ = 5.0 nM) and its derivative (**G**, IC_50_ = 0.043 nM)^12^. Other natural products, stilbenoids like 2,4,3′,5′-tetramethoxy-(*E*)-stilbene (**H**), alkaloids like berberine (**I**)^13^, and cannabinoids, inhibited CYP1B1 at mM concentrations^2^, but their efficacy is limited by poor water solubility, hindering in vivo dosing. Although estrogens^13–16^ and flavonoid-based derivatives^17–18^ have improved CYP1B1 inhibition, none exhibit optimal druggability. Similarly, Li group’s ANF modifications failed to enhance solubility or efficacy significantly^12^. Thus, we would pursue novel anti-CYP1B1 scaffolds with high selectivity and better water solubility for the improvement on the pharmacokinetics.

*Aquilariae Lignum* (also called agarwood), a scarce traditional medicinal resource in Southeast and East Asia, has been historically used to treat anxiety, pain, and inflammation^19–21^. Among its bioactive components, 2–(2-phenylethyl)-chromones, characterised by a unique phenylethyl substituent at the C-2 position of benzopyranone, have recently gained significant attention due to their diverse pharmacological activities, including antitumor, antioxidant, analgesia, anti-inflammation, and antibacterial effects^22–28^. To date, nearly 60 2–(2-phenylethyl)-chromone derivatives have been isolated and characterised, with some demonstrating antitumor potentials^26–28^. However, their anti-tumour mechanisms remain largely unexplored. Given 2–(2-phenylethyl)-chromones derived from the fusion of rings A and C of bioactive flavones and the phenylethyl group of berberine mentioned above (Figure 1), we focus on characterising their anti-CYP1B1 activity and mechanistic investigation.

In this study, we investigated twenty-three naturally occurring 2–(2-phenylethyl)-chromones and one synthetic 2–(2-phenylethyl)-chromone as potential non-toxic, effective CYP1B1 inhibitors. A series of chromones with diverse A- and B-ring substitutions were synthesised and assessed, using ANF as a positive control. Each compound was screened for inhibitory activity against CYP1B1 and CYP1A2 isoenzymes to analyse structure-activity relationships (SARs). Promising candidates demonstrating significant CYP1B1 inhibition and selectivity over CYP1A2 were further evaluated for their ability to reverse docetaxel resistance in CYP1B1-overexpressing MCF-7 cells. To confirm target engagement, we employed PROTAC technology, an emerging protein degradation strategy-complemented by molecular imprinting assays. Molecular docking simulation confirmed the binding of the potential compound with the CYP1B1 enzyme. This integrated approach aimed to identify novel natural-product-derived scaffolds for developing highly selective CYP1B1 inhibitors.

## Experimental section

All chemicals are of reagent grade or higher, purchased from commercial sources, and used as received unless otherwise specified. Solvents are employed without further purification or dried over molecular sieves (4 Å). Reactions are performed under inert atmosphere using standard Schlenk techniques. Key intermediates and final products are characterised by electrospray ionisation mass spectrometry (ESI-MS) (AB Sciex Triple TOF 5600+ system, sample injection rate: 0.1 ml/min; injection amount: 0.1 μL; solvent: LCMS grade methanol; positive-ESI mode; capillary voltage: 2.0 kv; source temperature:120 °C) and NMR spectroscopy (^1^H,^1^³C; Bruker Avance 400 spectrometer: ^1^H at 400 MHz, ^1^³C at 100 MHz). Chemical shifts (δ) are reported in ppm relative to residual solvent peaks as internal standards: Chloroform-*d* (7.26 ppm for ^1^H, 77.16 ppm for^1^³C), Dimethylsulfoxide-*d*_6_ (2.50 ppm for ^1^H, 39.51 ppm for^1^³C), Methanol-*d_4_* (3.31/4.78 ppm for ^1^H, 49.15 ppm for^1^³C), and Acetone-*d_6_* (2.05 ppm for ^1^H, 29.92/206.68 ppm for^1^³C). Purity was confirmed to be >95% by HPLC (Agilent 1260 Infinity II, C_18_ column).

### General procedure for the preparation of benzylated 2–(2-phenylethyl) chromones and target chromones (CX-1 ∼ 20, CX-2 2 ∼ 23)

A slurry of NaH (4.0 mmol) in DMF was heated to 50 °C, and a DMF solution (10 ml) of acetophenone (**4 A**-**4F**, 1.0 mmol) was added dropwise over 10 min. After heating for 1 h, the appropriate ester (**3 A**-**3F**, 1.5 mmol) was added dropwise over 15 min, and the mixture was refluxed for 2–8 h. The reaction was quenched with sat. aq. NH_4_Cl (50 ml), and extracted with EtOAc (40 ml × 3). The combined organic layers were dried by anhydrous Na_2_SO_4_ and concentrated to afford the crude Claisen product.

The unpurified Claisen product was dissolved in MeOH (20 ml), treated with excess 10 M HCl, and refluxed at 90 °C for 45 min. After cooling, the mixture was neutralised with sat. aq. Na_2_CO_3_, extracted with EtOAc (50 ml), and washed with H_2_O (25 ml) and brine (25 ml) in order. Drying with anhydrous Na_2_SO_4_ and concentration yielded crude chromones (**2 A**-2**P, 1 A-1B, 1I-1J, 1 M, 1 V** and **1 W**), which were purified by silica-gel column chromatography with various ratios of petroleum ether (PE) and ethyl EtOAc (V/V = 8 ∼ 4/1) to give the products in 42.6-82.8% yield.

The benzylated 2–(2-phenylethyl) chromones (1.0 mmol, **2 A**-**2P**) obtained above in MeOH (10 ml) were hydrogenated using 0.1% catalytic amounts of 10% Pd/C for 0.5 ∼ 2 h. The mixture was filtered, concentrated *in vacuo*, and purified by silica gel column chromatography with various ratios of PE and EtOAc (V/V = 6 ∼ 4/1) as an eluent to afford the target chromones (**CX-3 ∼ 8**, **CX-1 1 ∼ 12**, **CX-1 4 ∼ 20**, **CX-2 2 ∼ 23**) in 77.5-96.8% yields.

*2-phenethyl-4H-chromen-4-one* **(CX-1)**: Yield, 82.8%. ^1^H NMR (400 MHz, Chloroform-*d*) δ 8.07 (d, *J* = 8.0 Hz, 1H), 7.58 (d, *J* = 2.8 Hz, 1H), 7.31 (d, *J* = 8.0 Hz, 1H), 7.24 (d, *J* = 8.0 Hz, 1H), 7.18 (*t*, *J* = 8.0 Hz, 2H), 7.11 (*t*, *J* = 8.0 Hz, 3H), 6.03 (*s*, 1H), 2.95 (*t*, *J* = 8.0 Hz, 2H), 2.82 (*t*, *J* = 8.0 Hz, 2H); ^13^C NMR (100 MHz, Chloroform-*d*) δ 32.96, 36.01, 110.22, 117.88, 123.73, 125.00, 125.66, 126.58, 128.31, 128.67, 133.55, 139.74, 156.46, 168.47, 178.24. TOF-MS, *m/z*: [M + H^+^], calcd. for C_17_H_15_O_2_^+^, 251.1067, found: 251.1071.

*6-methoxy-2-phenethyl-4H-chromen-4-one* **(CX-2)**:Yield, 78.6%.^1^H NMR (400 MHz, Chloroform-*d*) δ 8.07 (d, *J* = 8.0 Hz, 1H), 7.34-7.30 (*m*, 1H), 7.28-7.24 (*m*, 2H), 7.22-7.16 (*m*, 4H), 6.11 (*s*, 1H), 3.84 (d, *J* = 4.0 Hz, 3H), 3.02-2.99 (*m*, 2H), 2.90-2.87 (*m*, 2H); ^13^C NMR (100 MHz, Chloroform-*d*) δ 32.97, 35.99, 55.84, 104.86, 109.41, 119.24, 123.45, 124.25, 126.53, 128.28, 128.63, 139.76, 151.25, 156.78, 168.21, 178.09. TOF-MS, *m*/*z*: [M + H^+^], calcd. for C_18_H_17_O_3_^+^, 281.1172, found: 281.1174.

*2-(4-(benzyloxy)phenethyl)-4H-chromen-4-one* **(CX-3Bn)** Yield, 63.9%. ^1^H NMR (400 MHz, Chloroform-*d*) δ 8.19 (d, *J* = 8.0 Hz, 1H), 7.65 (*t, J* = 8.0 Hz, 1H), 7.44-7.32 (*m*, 5H), 7.13 (d, *J* = 12.0 Hz, 2H), 6.91(*t*, *J* = 12.0 Hz, 2H), 6.15 (*s*, 1H), 5.04 (*s*, 2H), 3.03-2.99 (*m*, 2H), 2.91-2.88 (*m*, 2H). TOF-MS, *m/z*: [M + H^+^], calcd. for C_24_H_21_O_3_^+^, 357.1485, found: 357.1481.

*2-(4-hydroxyphenethyl)-4H-chromen-4-one* **(CX-3)**: Yield, 96.4%. ^1^H NMR (400 MHz, Acetone-*d6*) δ 8.96 (*s*, 1H), 8.23 (*s*, 1H), 7.46-7.42 (*m*, 2H), 7.26-7.23 (*m*, 1H), 7.09 (d, *J* = 8.0 Hz, 2H), 6.74 (d, *J* = 8.0 Hz, 2H), 6.04 (*s*, 1H), 2.99-2.86 (*m*, 4H); ^13^C NMR (100 MHz, Acetone-*d6*) δ 31.86, 35.96, 108.05, 108.84, 115.22, 119.31, 122.39 124.59, 129.31, 130.91, 150.42, 150.61, 150.90, 168.41,176.83. TOF-MS, *m/z*: [M + Na^+^], calcd. for C_17_H_14_NaO_3_^+^, 289.0835, found: 289.0841.

*2-(4-hydroxyphenethyl)-6-methoxy-4H-chromen-4-one* (**CX-4**): Yield, 84.9%. ^1^H NMR (400 MHz, Chloroform-*d*) 7.54 (d, *J* = 4.0 Hz, 1H), 7.39 (d, *J* = 8.0 Hz, 1H), 7.10-7.01 (*m*, 3H), 6.77 (d, *J* = 8.0 Hz, 3H), 6.13 (*s*, 1H), 3.89 (*s*, 3H), 3.01 (*t*, *J* = 8.0 Hz, 2H), 2.91 (*t*, *J* = 8.0 Hz, 2H); ^13^C NMR (100 MHz, Chloroform-*d*) δ 31.37, 35.25, 55.64, 104.76, 108.92, 115.3, 119.74, 123.03, 123.65, 126.53, 129.01, 129.33, 130.16, 150.55, 155.72, 156.38, 168.71, 176.49. TOF-MS, *m/z*: [M + Na^+^], calcd. for C_18_H_16_NaO_4_^+^, 319.0941, found: 319.0933.

*6-hydroxy-2-phenethyl-4H-chromen-4-one* (**CX-5**): Yield, 93.7%. ^1^H NMR (400 MHz, Acetone-*d6*) δ 8.92 (*s*, 1H), 7.46-7.43 (*m*, 1H), 7.29-7.24 (*m*, 2H), 7.24 (*m*, 5H), 6.03 (*s*, 1H), 3.11-3.01 (*m*, 2H), 2.99-2.97 (*m*, 2H); ^13^C NMR (100 MHz, Acetone-*d6*) δ 32.61, 35.53, 108.06, 108.86, 119.31, 122.39, 124.60, 126.23, 128.42, 140.36, 150.41, 150.60, 154.60, 168.15, 176.76. TOF-MS, *m/z*: [M + Na^+^], calcd. for C_17_H_14_NaO_3_^+^, 289.0835, found: 289.0839.

*5-(benzyloxy)-2-phenethyl-4H-chromen-4-one* **(CX-6Bn):** Yield, 52.1%. ^1^H NMR (400 MHz, Chloroform-*d*) 7.61 (*t, J* = 4.0 Hz, 2H), 7.44-7.35 (*m*, 3H), 7.28-7.25 (*m*, 3H), 7.20-7.18 (d, *J* = 4.0 Hz, 3H), 6.98-6.95 (dd, *J* = 4.0, 4.0 Hz, 1H), 6.80–6.87 (dd, *J* = 4.0, 4.0 Hz, 1H), 6.03 (*s*, 1H), 5.23 (*s*, 2H), 3.01 (*t*, *J* = 4.0 Hz, 2H), 2.85 (*t*, *J* = 4.0 Hz, 2H); ^13^C NMR (100 MHz, Chloroform-*d*) δ 32.82, 35.44, 70.80, 108.27, 110.32, 111.83, 114.91, 126.55, 126.64, 127.65, 128.35, 128.60, 128.67, 133.44, 136.67, 139.86, 158.55, 165.91, 177.94.

*5-hydroxy-2-phenethyl-4H-chromen-4-one* **(CX-6)**: Yield, 89.5%. ^1^H NMR (400 MHz, Chloroform-*d*) δ 12.55 (*s*, 1H), 7.47 (*t*, *J* = 8.0 Hz, 1H), 7.28 (*t*, *J* = 8.0 Hz, 2H), 7.18 (*t*, *J* = 4.0 Hz, 3H), 6.84 (*t*, *J* = 8.0 Hz, 1H), 6.74 (*t*, *J* = 8.0 Hz, 1H), 6.02 (*s*, 1H), 3.0 (*t*, *J* = 8.0 Hz, 2H), 2.90 (*t*, *J* = 8.0 Hz, 2H); ^13^C NMR (100 MHz, Chloroform-*d*) δ 32.82, 36.02, 106.93, 108.78, 110.58, 111.2, 126.69, 128.33, 128.74, 135.19, 139.54, 156.73, 160.81, 169.95, 183.41. TOF-MS, *m/z*: [M + H^+^], calcd. for C_17_H_15_O_2_^+^, 267.1016, found: 267.1020.

*6-(benzyloxy)-2-(4-(benzyloxy)phenethyl)-4H-chromen-4-one* **(CX-7Bn):** Yield, 49.3%. ^1^H NMR (400 MHz, Chloroform-*d*) δ 7.65 (d, *J* = 4.0 Hz,1H), 7.47-7.30 (*m*, 13 H), 7.12 (d, *J* = 12.0 Hz, 2H), 6.91 (d, *J* = 12.0 Hz, 2H), 6.13 (*s*, 1H), 5.13 (*s*, 2H), 6.13 (*s*, 1H), 3.01 (*t*, *J* = 8.0 Hz, 2H), 2.88 (*t*, *J* = 8.0 Hz, 2H). TOF-MS, *m/z*: [M + H^+^], calcd. for C_31_H_27_O_4_^+^, 462.1831, found: 462.1827.

*6-hydroxy-2-(4-hydroxyphenethyl)-4H-chromen-4-one* **(CX-7)**: Yield, 96.8%. ^1^H NMR (400 MHz, Dimethyl sulfoxide-*d6*) δ 9.95 (*s*, 1H), 9.19 (*s*, 1H), 7.47 (d, *J* = 8.0 Hz, 1H), 7.26 (d, *J* = 4.0 Hz, 1H), 7.20 (*t*, *J* = 8.0 Hz, 1H), 7.04 (*t*, *J* = 8.0 Hz, 2H), 6.66 (d, *J* = 8.0 Hz, 2H), 6.09 (*s*, 1H), 2.88 (*s*, 4H); ^13^C NMR (100 MHz, Dimethyl sulfoxide-*d6*) δ 31.81, 35.74, 109.11, 115.59, 119.91, 123.2, 124.43, 129.67, 130.55, 128.33, 150.08, 155.06, 169.01, 177.16. TOF-MS, *m*/*z*: [M + Na^+^], calcd. for C_17_H_14_NaO_3_^+^, 305.0784, found: 305.0778.

*5-(benzyloxy)-2-(4-(benzyloxy)phenethyl)-4H-chromen-4-one* **(CX-8Bn)**: Yield, 47.1%. ^1^H NMR (400 MHz, Chloroform-*d*) δ 7.62 (d, *J* = 8.0 Hz,2H), 7.47-7.29 (*m*, 9H), 7.11(d, *J* = 8.0 Hz, 2H), 7.00 (d, *J* = 8.0 Hz, 1H), 6.91 (d, *J* = 8.0 Hz, 2H), 6.83 (d, *J* = 8.0 Hz, 1H), 6.04 (*s*, 1H), 5.21 (*s*, 2H), 5.03 (*s*, 2H), 2.89 (*t*, *J* = 4.0 Hz, 2H), 2.84 (d, *J* = 8.0 Hz, 2H); ^13^C NMR (100 MHz, Chloroform-*d*) δ 32.01, 35.75, 53.45, 70.07, 70.90, 108.25, 110.32, 111.86, 115.00, 126.63, 127.52, 127.65, 127.96, 128.59, 129.31, 132.17, 133.37, 136.60, 137.14, 157.46, 158.57, 166.00, 174.29.

*5-hydroxy-2-(4-hydroxyphenethyl)-4H-chromen-4-one* **(CX-8)**: Yield, 77.5%. ^1^H NMR (400 MHz, Acetone-*d6*) δ 12.60 (*s*, 1H), 8.10 (*s*, 1H), 7.63 (d, *J* = 4.0 Hz, 1H), 7.11 (d, *J* = 8.0 Hz, 2H), 6.99 (d, *J* = 8.0 Hz, 1H), 6.77-6.73 (d, *J* = 8.0 Hz, 3H), 6.13 (*s*, 1H), 2.99 (*t*, *J* = 4.0 Hz, 4H); ^13^C NMR (100 MHz, Acetone-*d6*) δ 31.70, 35.94, 106.89, 108.49, 115.27, 129.33, 130.66, 135.40, 155.95, 156.83, 160.87, 170.90, 183.40. TOF-MS, *m/z*: [M + Na^+^], calcd. for C_17_H_14_NaO_4_^+^, 305.0784, found: 305.0781.

*5-(benzyloxy)-2-(4-methoxyphenethyl)-4H-chromen-4-one* **(CX-9Bn)**: Yield, 78.3%. ^1^H NMR (400 MHz, Chloroform-*d*) δ 7.61 (*t*, *J* = 8.0 Hz, 2H), 7.49 (*t*, *J* = 8.0 Hz, 1H), 7.41(*t*, *J* = 8.0 Hz, 2H), 7.31 (*t*, *J* = 8.0 Hz, 1H), 7.12 (d, *J* = 8.0 Hz, 2H), 7.00 (d, *J* = 8.0 Hz, 1H), 6.83 (d, *J* = 8.0 Hz, 3H), 6.04 (*s*, 1H), 5.26 (*s*, 2H), 3.78 (*s*, 3H), 2.98 (*t*, *J* = 8.0 Hz, 2H), 2.85 (*t*, *J* = 8.0 Hz, 2H). TOF-MS, *m*/*z*: [M + Na^+^], calcd. for C_25_H_22_NaO_4_^+^, 409.1410, found: 409.1403.

*5-hydroxy-2-(4-methoxyphenethyl)-4H-chromen-4-one* **(CX-9)**: Yield, 92.1%. ^1^H NMR (400 MHz, Chloroform-*d*) δ 12.54 (s, 1H), 7.51 (*t*, *J* = 8.0 Hz, 1H), 7.11 (d, *J* = 4.0 Hz, 2H), 6.88-6.77 (*m*, 3H), 6.82 (d, *J* = 8.0 Hz, 1H), 6.04 (*s*, 1H), 3.78 (*s*, 3H), 3.01 (*t*, *J* = 8.0 Hz, 2H), 2.90 (d, *J* = 8.0 Hz, 2H); ^13^C NMR (100 MHz, Chloroform-*d*) δ 32.01, 36.36, 55.27, 106.90, 108.85, 110.59, 111.21, 114.10, 129.25, 131.47, 135.17, 156.75, 158.32, 160.79, 170.11, 183.51. TOF-MS, *m*/*z*: [M + Na^+^], calcd. for C_18_H_17_O_4_^+^, 297.1121, found: 297.1123.

*6-methoxy-2-(4-methoxyphenethyl)-4H-chromen-4-one* **(CX-10)**: Yield, 68.4%. ^1^H NMR (400 MHz, Chloroform-*d*) δ 7.68 (d, *J* = 4.0 Hz, 1H), 7.51 (d, *J* = 8.0 Hz, 1H), 7.38-7.35 (*m*, 1H), 7.25-7.23 (d, *J* = 8.0 Hz, 2H), 6.98 (d, *J* = 8.0 Hz, 2H), 6.26 (*s*, 1H), 4.00 (*s*, 3H), 3.90 (*s*, 3H), 3.12-3.10 (*m*, 2H), 3.03-2.99 (*m*, 2H); ^13^C NMR (100 MHz, Chloroform-*d*) δ 32.04, 36.20, 55.13, 55.76, 104.80, 109.37, 113.96, 119.20, 123.26, 124.21, 129.19, 131.72, 151.19, 156.70, 158.19, 168.26, 177.95. TOF-MS, *m/z*: [M + H^+^], calcd. for C_19_H_19_O_4_^+^, 311.1278, found: 311.1284.

*6-hydroxy-2-(4-methoxyphenethyl)-4H-chromen-4-one* **(CX-11)**: Yield, 93.2%. ^1^H NMR (400 MHz, Chloroform-*d*) δ 7.88 (d, *J* = 4.0 Hz, 1H), 7.38 (d, *J* = 8.0 Hz, 1H), 7.28 (d, *J* = 8.0 Hz, 1H), 7.12(d, *J* = 8.0 Hz, 2H), 6.84 (d, *J* = 8.0 Hz, 2H), 6.18 (*s*, 1H), 3.78 (*s*, 3H), 3.02-3.00 (*m*, 2H), 2.91-2.88 (*m*, 2H); ^13^C NMR (100 MHz, Chloroform-*d*) δ 32.06, 36.34, 55.09, 109.06, 113.88, 119.04, 123.30, 129.26, 131.57, 147.49, 150.92, 154.22, 158.065, 169.40, 179.03. TOF-MS, *m/z*: [M + Na^+^], calcd. for C_18_H_16_NaO_4_^+^, 319.0941, found: 319.0935.

*2-(4-methoxyphenethyl)-4H-chromen-4-one* **(CX-12)**: Yield, 56.7%. ^1^H NMR (400 MHz, Chloroform-*d*) δ 8.14 (d, *J* = 8.0 Hz, 1H), 7.56 (*t*, *J* = 4.0 Hz, 1H), 7.20-7.18 (*t*, *J* = 8.0 Hz, 1H), 7.35-7.33(d, *J* = 8.0 Hz, 1H), 7.29-7.25(*t, J* = 4.0 Hz, 1H), 7.07 (d, *J* = 8.0 Hz, 2H), 6.79 (d, *J* = 8.0 Hz, 1H), 6.06 (*s*, 1H), 3.67 (d, *J* = 4.0 Hz, 3H), 2.92-2.88 (*m*, 2H), 2.80-2.76 (*m*, 2H); ^13^C NMR (100 MHz, Chloroform-*d*) δ 31.73, 35.93, 54.93, 109.84, 113.84, 117.75, 123.46, 124.80, 125.30, 129.11, 131.61, 133.40, 156.21, 158.07, 168.57, 177.83. TOF-MS, *m/z*: [M + H^+^], calcd. for C_18_H_17_O_3_^+^, 281.1172, found: 281.1179.

*5-methoxy-2-(4-methoxyphenethyl)-4H-chromen-4-one* **(CX-13):** Yield, 42.6%. ^1^H NMR (400 MHz, Chloroform-*d*) δ 7.51 (*t*, *J* = 8.0 Hz, 1H), 7.10 (d, *J* = 12.0 Hz, 2H), 6.98 (d*, J* = 8.0 Hz, 1H), 6.81 (*t*, *J* = 8.0 Hz, 3H), 6.02 (*s*, 1H), 3.95 (*s*, 3H), 3.76 (*s*, 3H), 2.97 (d, *J* = 8.0 Hz, 2H), 2.82 (*t*, *J* = 8.0 Hz, 2H); ^13^C NMR (100 MHz, Chloroform-*d*) δ 31.93, 35.44, 56.44, 55.26, 106.33, 109.98, 111.76, 114.01, 114.34, 129.26, 131.84, 133.53, 158.21, 158.54, 159.73, 166.06, 178.24. TOF-MS, *m/z*: [M + Na^+^], calcd. for C_19_H_18_NaO_4_^+^, 333.1097, found: 333.1099.

*2-(4-(benzyloxyl)-3-methoxyphenethyl)-4H-chromen-4-one* **(CX-14Bn)**: Yield, 71.6%. ^1^H NMR (400 MHz, Chloroform-*d*) δ 8.18 (d, *J* = 8.0 Hz, 1H), 7.65 (*t*, *J* = 8.0 Hz, 1H), 7.43-7.32 (*m*, 7 H), 6.82 (d, *J* = 8.0 Hz, 1H), 6.72 (d, *J* = 8.0 Hz, 2H), 6.10 (*s*, 1H), 5.07 (*s*, 2H), 3.86 (*s*, 3H), 2.97 (*t*, *J* = 8.0 Hz, 2H), 2.86 (d, *J* = 8.0 Hz, 2H); ^13^C NMR (100 MHz, Chloroform-*d*) δ 32.48, 36.28, 56.08, 71.15, 110.31, 112.02, 114.57, 117.84, 120.97, 123.74, 125.03, 125.72, 127.30, 127.86, 128.54, 132.18, 133.62, 137.09, 148.16, 148.46, 148.49, 156.46, 168.50, 178.54.

*2-(4-hydroxy-3-methoxyphenethyl)-4H-chromen-4-one* **(CX-14)**: Yield, 87.8%. ^1^H NMR (400 MHz, Chloroform-*d*) δ 8.17 (d, *J* = 8.0 Hz, 1H), 7.66 (*t*, *J* = 8.0 Hz, 1H), 7.45 (d, *J* = 8.0 Hz, 1H), 7.38 (*t*, *J* = 8.0 Hz, 1H), 6.79 (*s*, 1H), 6.75 (d, *J* = 8.0 Hz, 1H), 6.65 (d, *J* = 8.0 Hz, 1H), 6.14 (*s*, 1H), 3.85 (*s*, 3H), 2.95 (*t*, *J* = 8.0 Hz, 2H), 2.90 (d, *J* = 8.0 Hz, 2H); ^13^C NMR (100 MHz, Chloroform-*d*) δ 32.4, 136.23, 55.99, 104.82, 109.52, 119.28, 119.69, 123.56, 124.27, 133.00, 145.27, 145.70, 151.35, 156.78, 168.33, 178.24. TOF-MS, *m/z*: [M + Na^+^], calcd. for C_18_H_16_NaO_4_^+^, 319.0941, found: 319.0935.

*6-methoxy-2-(3-methoxyphenethyl)-4H-chromen-4-one* **(CX-15)**: Yield, 92.6%. ^1^H NMR (400 MHz, Chloroform-*d*) δ 7.55 (d, *J* = 4.0 Hz, 1H), 7.39 (d, *J* = 8.0 Hz, 2H), 7.27-7.24 (*m,* 1H), 6.85 (*t*, *J* = 8.0 Hz, 1H), 6.69-6.67 (*m*, 2H), 6.16 (*s*, 1H), 3.89 (*s*, 3H), 3.82 (*s*, 3H), 3.01 (*t*, *J* = 8.0 Hz, 2H), 2.92 (*t*, *J* = 8.0 Hz, 2H); ^13^C NMR (100 MHz, Chloroform-*d*) δ 32.84, 36.49, 55.88, 55.94, 104.84, 109.52, 110.86, 114.54, 119.24, 120.92, 123.60, 124.27, 131.64, 144.27, 146.55, 151.32, 156.82, 168.38, 178.29. TOF-MS, *m/z*: [M + Na^+^], calcd. for C_19_H_18_NaO_4_^+^, 333.1097, found: 333.1103.

*2-(3-(benzyloxy)-4-methoxyphenethyl)-4H-chromen-4-one* **(CX-16Bn): **Yield, 57.3%. ^1^H NMR (400 MHz, Chloroform-*d*) δ 8.18 (d, *J* = 8.0 Hz, 1H), 7.63 (*t*, *J* = 8.0 Hz, 1H), 7.44-7.28 (*m*, 7 H), 6.81 (d, *J* = 8.0 Hz, 1H), 6.73 (*s*, 1H), 6.67 (d, *J* = 8.0 Hz, 1H), 6.15 (*s*, 1H), 5.11 (*s*, 2H), 3.81 (*s*, 3H), 3.01 (*t*, *J* = 8.0 Hz, 2H), 2.91 (d, *J* = 8.0 Hz, 2H); ^13^C NMR (100 MHz, Chloroform-*d*) δ 32.68, 36.32, 55.99, 71.15, 110.27, 112.16, 114.32, 117.84, 120.25, 123.72, 125.04, 125.71, 127.31, 127.83, 128.55, 132.95, 133.58, 137.23, 146.86, 149.71, 156.47, 168.56, 178.31.

*2-(3-hydroxy-4-methoxyphenethyl)-4H-chromen-4-one* **(CX-16)**: Yield, 93.8%. ^1^H NMR (400 MHz, Chloroform-*d*) δ 8.18 (d, *J* = 8.0 Hz, 1H), 7.66 (*t*, *J* = 8.0 Hz, 1H), 7.46-7.37 (*m, J* = 8.0 Hz, 2H), 6.85 (*t*, *J* = 8.0 Hz, 1H), 6.73-6.67 (*t*, *J* = 8.0 Hz, 3H), 6.16(*s*, 1H), 3.81 (*s,* 3H), 3.01-2.91 (d, *J* = 8.0 Hz, 2H), 2.90-2.88 (*t*, *J* = 8.0 Hz, 2H); ^13^C NMR (100 MHz, Chloroform-*d*) δ 32.27, 36.19, 55.93, 110.18, 110.76, 114.46, 117.84, 119.61, 123.62, 124.95, 125.64, 132.89, 133.50, 145.26, 145.68, 156.44, 168.56, 178.35. TOF-MS, *m*/*z*: [M + Na^+^], calcd. for C_18_H_16_NaO_4_^+^, 319.0941, found: 319.0944.

*2-(3-(benzyloxy)-4-methoxyphenethyl)-6-methoxy-4H-chromen-4-one* **(CX-17Bn)**: Yield, 75.2%. ^1^H NMR (400 MHz, Chloroform-*d*) δ 7.54 (d, *J* = 4.0 Hz, 1H), 7.41-7.33 (*m*, 5H), 7.30-7.22 (*m*, 3H), 6.81(d, *J* = 8.0 Hz, 1H), 6.75-6.71 (*m*, 2H), 6.08 (*s*, 1H), 5.08 (*s*, 2H), 3.88 (*s*, 3H), 3.85 (*s*, 3H), 2.98 (*t*, *J* = 8.0 Hz, 2H), 2.85 (d, *J* = 8.0 Hz, 2H); ^13^C NMR (100 MHz, CDCl_3_) δ 32.54, 36.25, 55.93, 56.07, 71.14, 104.84, 109.56, 112.00, 114.55, 119.26, 120.96, 123.54, 124.28, 127.31, 127.86, 128.54, 132.22, 137.09, 148.16, 148.44, 151.29, 156.81, 168.20, 178.11.

*2-(3-hydroxy-4-methoxyphenethyl)-6-methoxy-4H-chromen-4-one* **(CX-17)**: Yield, 94.5%. ^1^H NMR (400 MHz, Chloroform-*d*) δ 7.54 (d, *J* = 4.0 Hz, 1H), 7.38 (d, *J* = 8.0 Hz, 1H), 7.27-7.20 (*m,* 2H), 6.80 (*t*, *J* = 8.0 Hz, 2 H), 6.66 (*t*, *J* = 8.0 Hz, 1H), 6.13 (*s*, 1H), 3.89 (*s*, 3H), 3.86 (*s*, 3H), 2.99-2.94 (*m*, 2H), 2.91-2.87 (*m*, 2H); ^13^C NMR (100 MHz, Chloroform-*d*) δ 32.41, 36.23, 55.99, 104.82, 109.52, 119.28, 119.69, 123.56, 124.27, 133.00, 145.27, 145.70, 151.35, 156.78, 168.33, 178.28. TOF-MS, *m*/*z*: [M + Na^+^], calcd. for C_19_H_18_NaO_5_^+^, 349.1046, found: 349.1050.

*6-(benzyloxy)-2-(4-(benzyloxy)-3-methoxyphenethyl)-4H-chromen-4-one* **(CX-18Bn)**: Yield, 54.7%. ^1^H NMR (400 MHz, Chloroform-*d*) δ 7.65 (d, *J* = 4.0 Hz, 1H), 7.64-7.25 (*m*, 12H), 6.81(d, *J* = 8.0 Hz, 1H), 6.71 (*s*, 1H), 6.67 (d, *J* = 8.0 Hz, 1H), 6.15 (*s*, 1H), 5.13 (d, *J* = 4.0 Hz, 4H), 3.82 (*s*, 3H), 3.00 (*t*, *J* = 8.0 Hz, 2H), 2.91 (d, *J* = 8.0 Hz, 2H); ^13^C NMR (100 MHz, Chloroform-*d*) δ 32.76, 36.30, 55.01, 70.63, 71.15, 106.10, 109.56, 112.15, 114.30, 119.34, 120.24, 123.54, 124.28, 127.31, 127.61, 127.73, 127.25, 128.56, 128.68, 132.97,136.29, 137.24, 146.85, 149.69, 151.42, 155.94, 168.31, 178.18.

*6-hydroxyl-2-(4-hydroxyl-3-methoxyphenethyl)-4H-chromen-4-one* **(CX-18)**: Yield, 86.4%. ^1^H NMR (400 MHz, Methanol-*d_4_*) δ 7.45 (d, *J* = 12.0 Hz, 1H), 7.37 (d, *J* = 8.0 Hz, 1H), 7.23 (ddd, *J* = 12.0, 8.0, 4.0 Hz, 1H), 6.87 (*s*, 1H), 6.68 (d, *J* = 8.0 Hz, 1H), 6.62 − 6.57 (*m*, 3H), 6.08 (*s*, 1H), 3.72 (*s*, 3H), 2.97-2.85 (*m*, 4H). ^13^C NMR (100 MHz, Chloroform-*d*) δ 31.69, 35.13, 55.41, 107.56, 108.44, 112.30, 115.62, 118.94, 119.63, 122.26, 124.64, 132.74, 146.05, 146.53, 149.82, 155.74, 168.52, 176.92. TOF-MS, *m*/*z*: [M + Na^+^], calcd. for C_18_H_16_NaO_5_^+^, 335.0890, found: 335.0895.

*5-(benzyloxy)-2-(4-(benzyloxy)-3-methoxyphenethyl)-4H-chromen-4-one* **(CX-19Bn)**: Yield, 74.6%. ^1^H NMR (400 MHz, Chloroform-*d*) δ 7.62 (d, *J* = 4.0 Hz, 2H), 7.40-7.30 (*m*, 7H), 7.29-7.27 (d, *J* = 8.0 Hz, 2H), 6.98-6.96 (d, *J* = 8.0 Hz, 1H), 6.88-6.78 (*t*, *J* = 8.0 Hz, 2H), 6.73 (d, *J* = 4.0 Hz, 1H), 6.67 (d, *J* = 8.0 Hz, 1H), 6.06 (*s*, 1H), 5.26 (*s*, 2H), 5.12 (*s*, 2H), 3.83 (*s*, 3H), 2.99-2.95(*t*, *J* = 8.0 Hz, 2H), 2.85-2.82 (d, *J* = 8.0 Hz, 2H); ^13^C NMR (100 MHz, Chloroform-*d*) δ 32.54, 35.67, 56.00, 70.84, 71.16, 108.29, 111.83, 112.15, 114.29, 114.93, 120.24, 126.64, 127.31, 127.82, 128.60, 133.07, 136.60, 137.26, 146.83, 149.69, 158.50, 165.90, 177.91.

*5-hydroxy-2-(4-hydroxy-3-methoxyphenethyl)-4H-chromen-4-one* **(CX-19)**: Yield, 93.4%. ^1^H NMR (400 MHz, Chloroform-*d*) δ7.51 (*t*, *J* = 8.0 Hz, 1H), 6.89-6.79 (*m,* 2H), 6.71(d, *J* = 4.0 Hz, 1H), 6.69 (*t*, *J* = 8.0 Hz, 2 H), 6.07 (*s*, 1H), 3.83 (*s*, 3H), 3.01-2.96 (*m*, 2H), 2.90-2.87 (*m*, 2H); ^13^C NMR (100 MHz, Chloroform-*d*) δ 32.69, 36.49, 55.89, 106.85, 108.88, 110.81, 111.29, 114.57, 120.92, 131.33, 135.22, 144.33, 146.57, 156.74, 160.75, 169.99, 183.52. TOF-MS, *m*/*z*: [M + H^+^], calcd. for C_18_H_17_O_5_^+^, 313.1071, found: 313.1073.

*6-hydroxy-2-(3-hydroxy-4-methoxyphenethyl)-4H-chromen-4-one* (**CX-20**): Yield, 87.6%. ^1^H NMR (400 MHz, Methanol-*d_4_*) δ 7.46 (*t*, *J* = 8.0 Hz, 1H), 7.39 (*s*, 1H), 7.24 (d*, J* = 12.0 Hz, 1H), 6.80 (d, *J* = 8.0 Hz, 1H), 6.68 (*s*, 1H), 6.63 (d, *J* = 8.0 Hz, 1 H), 6.08 (*s*, 1H), 3.79 (*s*, 3H), 2.94 (*s*, 4H); ^13^C NMR (100 MHz, Methanol-*d_4_*) δ 32.05, 35.88, 55.02, 102.52, 107.30, 108.36, 111.45, 115.03, 119.16, 127.23, 127.85, 132.79, 146.18, 150.68, 155.03, 170.08, 179.22. TOF-MS, *m/z*: [M + H^+^], calcd. for C_18_H_17_O_5_^+^, 313.1071, found: 313.1075.

### 5-hydroxy-2-(3-hydroxy-4-methoxyphenethyl)-4H-chromen-4-one (CX-21)

A stirred solution of **5B** (1.0 mmol) and **7 A** (1.1 equiv.) in DCM was treated with DCC (1.3 equiv.) and DMAP (0.3 equiv.). After stirring at r.t. for 4 h, the mixture was filtered through Celite, and the filtrate was concentrated *in vacuo*. Flash chromatography with Et_2_O and EtOAc (V/V = 1:1) as an eluent yielded **12**.

To a solution of **12** (1.0 mmol) and KOH (1.5 equiv.) in dry pyridine was added and stirred at 100 °C for 4 h. The reaction was quenched with sat. aq. NH_4_Cl and extracted with DCM (15 ml × 3). The combined organic layers were washed with dd H_2_O (25 ml × 2), brine (25 ml), dried Na_2_SO_4_, and concentrated under the reduced pressure. The crude product was dissolved in DMSO, treated with *p*-TsOH (1.5 equiv.), and stirred at 100 °C for 2 h. After solvent removal, the mixture was extracted with DCM (15 ml × 3), and the combined layer was dried Na_2_SO_4_, and then concentrated under the reduced pressure. Purification by silica-gel column chromatography with PE and EtOAc (V/V = 5:1) as an eluent afforded **13**, and the latter was further hydrogenated using catalytic amount of 10% Pd/C to furnish crude product, which was further purified by PE and EtOAc ((V/V = 6:1) to yield **CX-21**.

*(E)-5-hydroxy-2-(3-hydroxy-4-methoxystyryl) chroman-4-one* (**13**): Yield, 60.2%. ^1^H NMR (400 MHz, Chloroform-*d*) δ 12.72 (*s*, 1H), 7.61-7.57 (*m*, 2H), 7.34 (*s*, 1H), 7.27 (*s*, 1H), 7.16 (d, *J* = 16.0 Hz, 1H), 7.06 (d, *J* = 12.0 Hz, 1H), 6.94 (d, *J* = 12.0 Hz, 1 H), 6.87 (d, *J* = 12.0 Hz, 1H), 6.28 (*s*, 1H), 4.02 (*s*, 3H); ^13^C NMR (100 MHz, Chloroform-*d*) δ 35.06, 56.19, 106.96, 108.53, 110.82, 112.86, 117.87, 121.73, 128.57, 135.35, 138.03, 146.21, 148.65, 156.35, 160.96, 163.42, 183.68.

**CX-21**, yield, 82%, in terms of **13**, ^1^H NMR (400 MHz, Methanol-*d_4_*) δ 12.52 (*s*, 1H), 7.51 (*t*, *J* = 8.0 Hz, 1H), 6.88 (d, *J* = 4.0 Hz, 1H), 6.77 (dd*, J* = 4.0, 8.0 Hz, 3H), 6.64 (d, *J* = 8.0 Hz, 1H), 6.04 (*s*, 1H), 3.85 (*s*, 3H), 2.97-2.85 (*m*, 4H); ^13^C NMR (100 MHz, Methanol-*d_4_*) δ 32.23, 36.21, 55.97, 106.00, 108.88, 110.60, 111.21, 114.43, 119.66, 132.64, 135.15, 145.34, 159.76, 160.79, 169.94, 183.53. TOF-MS, *m/z:* [M + H^+^], calcd. for C_18_H_17_O_5_^+^, 313.1071, found: 313.1074.

*5-chloro-2-phenethyl-4H-chromen-4-one* **(CX-22)**: Yield, 58.6%. ^1^H NMR (400 MHz, Chloroform-*d*) δ 7.48 (*t*, *J* = 8.0 Hz, 1H), 7.41-7.22 (*m*, 4H), 7.20-7.17 (*m*, 3 H), 6.07 (*s*, 1H), 3.04 (*t*, *J* = 8.0 Hz, 2H), 2.89 (*t*, *J* = 8.0 Hz, 2H); ^13^C NMR (100 MHz, Chloroform-*d*) δ 32.71, 35.45, 111.54, 117.19, 120.72, 126.60, 127.91, 128.30, 128.68, 130.30, 132.65, 139.60, 158.00, 166.72, 176.80. TOF-MS, *m/z*: [M + H^+^], calcd. for C_17_H_14_ClO_2_^+^, 285.0677, found: 285.0680.

*2-(3- (benzyloxy)phenethyl)-4H-chromen-4-one* **(CX-23Bn**): Yield, 78.4%. ^1^H NMR (400 MHz, Chloroform-*d*) δ 8.18 (*t*, *J* = 8.0 Hz, 1H), 7.67-7.65 (*t*, *J* = 8.0 Hz, 1H), 7.40-7.25 (*m*, 7H), 7.23-7.19(*t, J* = 8.0 Hz, 1H), 6.84-6.80 (*m*, 3H), 6.15 (*s*, 1H), 5.02 (*s*, 2H), 3.05 (*t*, *J* = 8.0 Hz, 2H), 2.94 (t, *J* = 8.0 Hz, 2H); ^13^C NMR (100 MHz, Chloroform-*d*) δ 32.99, 35.99, 68.97, 110.27, 112.70, 115.13, 117.87, 120.94, 123.74, 125.04, 125.73, 127.52, 128.01, 128.61, 133.58, 136.94, 141.36, 156.49, 159.08, 168.47, 178.34.

*2-(3-hydroxyphenethyl)-4H-chromen-4-one* **(CX-23**): Yield, 93.4%. ^1^H NMR (400 MHz, Chloroform-*d*) δ 8.20 (d, *J* = 8.0 Hz, 1H), 7.68 (*t*, *J* = 8.0 Hz, 1H), 7.42-7.22 (*t*, *J* = 8.0 Hz, 2H), 7.17(*t*, *J =* 8.0 Hz,1H), 6.70–6.71 ((*t*, *J* = 8.0 Hz, 3 H), 6.21 (*s*, 1H), 3.03 (*t*, *J* = 8.0 Hz, 2H), 2.94 (*t*, *J* = 8.0 Hz, 2H); ^13^C NMR (100 MHz, Chloroform-*d*) δ 32.71, 35.89, 110.21, 113.58, 115.26, 117.84, 120.58, 123.66, 125.04, 125.71, 129.81, 133.57, 141.52, 155.91, 156.50, 168.51, 178.47. TOF-MS, *m*/*z*: [M + H^+^], calcd. for C_17_H_15_O_3_^+^, 267.1016, found: 267.1019.

*6,7-dimethoxy-2-phenethyl-4H-chromen-4-one* **(CX-24):** Yield, 86.5%. ^1^H NMR (400 MHz, Chloroform-*d*) δ 7.76 (s 1H), 7.37-7.32 (*m*, 5H), 7.06 (*s*, 1H), 6.33 (*s*, 1H), 3.85 (*s*, 3H), 3.77 (*s*, 3H), 2.97-2.91 (*m*, 4H); ^13^C NMR (100 MHz, Chloroform-*d*) δ 32.28, 35.18, 55.51, 98.73, 103.60, 108.75, 125.69, 128.42, 128.77, 128.80, 138.91, 146.39, 151.66, 154.84, 167.60, 176.89. TOF-MS, *m/z*: [M + H^+^], calcd. for C_19_H_19_O_4_^+^, 267.1016, found: 267.1019.

### Synthesis of ethyl 3-(3-hydroxy-4-methoxyphenyl) propanoate (9 A) and ethyl 3-(4-hydroxy-3-methoxyphenyl) propanoate (9B)

A solution of commercially available ferulic acid (**7 A**, 19.4 g, 100 mmol) or isoferulic acid (**7B**, 19.4 g, 100 mmol) in ethanol (100 ml) was treated dropwise with concentrated H_2_SO_4_ (0.5 ml) and refluxed for 4 h. Upon completion, the ethanol was removed *in vacuo*, and the residue was dissolved in water (50 ml). The pH was adjusted to neutrality with K_2_CO_3_, and the product was extracted with EtOAc (50 ml × 3). The combined organic layers were dried by anhydrous Na_2_SO_4_, and the filtrate was concentrated under reduced pressure to afford crude **8 A** or **8B**, which was used directly in the next step without further purification.

To a stirred solution of olefin **8 A** or **8B** (11.1 g, 50.0 mmol) in methanol (75 ml) at r. t. was added 10% Pd/C (704 mg). The suspension was hydrogenated until TLC analysis indicated complete consumption of the starting material. The reaction mixture was filtered through a Celite pad to remove the catalyst, and the filtrate was concentrated under reduced pressure. The resulting colourless solid was recrystallized from a mixture of EtOAc and PE to afford the product as colourless crystals **9 A** or **9B**, respectively.

**9A**, yield, 78.2% in terms of **7 A**.^1^H NMR (400 MHz, Chloroform-*d*) δ 6.76 (d, *J* = 4.0 Hz, 2H), 6.68 (d, *J* = 4.0 Hz, 1H), 5.57 (br 1H), 4.15 (*m*, 2H), 3.87 (*s*, 3H), 2.88 (*t*, *J* = 8.0 Hz, 2H), 2.58 (*t*, *J* = 8.0 Hz, 2H), 1.26 (*m*, 3H); ^13^C NMR (100 MHz, Chloroform-*d*) δ 14.24, 30.40, 36.13, 56.00, 60.41, 110.67, 114.52, 119.66, 133.93, 145.03, 145.53, 173.00.

**9B**, yield, 82.4% in terms of **7B**. ^1^H NMR (400 MHz, Chloroform-*d*) δ 6.84 (d, *J* = 4.0 Hz, 1H), 6.71-6.68 (m, 2H), 5.64 (br 1H), 4.13(t, *J* = 4.0 Hz, 2H), 3.86 (*s,* 3H), 2.90 (*t*, *J* = 8.0 Hz, 2H), 2.61 (*t*, *J* = 8.0 Hz, 2H), 1.26 (*t*, *J* = 4.0 Hz, 3H); ^13^C NMR (100 MHz, Chloroform-*d*) δ 14.36, 55.93, 60.42, 109.36, 114.77, 115.58, 123.03, 127.01, 144.75, 146.83, 147.96, 167.39.

### Synthesis of 1-(2-(benzyloxy)-6-hydroxyphenyl) ethan-1-one (4D)

In a 50 ml round-bottom flask, 2,6-dihydroxyl acetophenone (1.0 g, 6.6 mmol) was added under a nitrogen atmosphere and dissolved in 10 ml of acetone. Subsequently, 0.94 ml of benzyl bromide (BnBr, 1.2 equiv.), potassium iodide (1.8 g, 1.6 equiv.), and K_2_CO_3_ (3 g, 3.2 equiv.) were added sequentially. The reaction mixture was heated to 60 °C and stirred under refluxing conditions overnight. The reaction progress was monitored by TLC (PE/EtOAc =10:1). After completion of the reaction, the mixture was allowed to cool to r.t. naturally, followed by filtration and concentration of the filtrate under reduced pressure. The residue was extracted with EtOAc, washed sequentially with water and saturated brine, and then concentrated *in vacuo*. The crude product was purified by column chromatography on silica gel using PE and EtOAc (V/V = 25/1) to afford 538 mg of **4D**. Similarly, other α-hydroxyl acetophenone the benzylation procedure.

**4D**, Yield, 34%. ^1^H NMR (400 MHz, Chloroform-*d*) δ 13.25 (s, 1H), 7.47-7.29 (m, 6H), 6.71 (m, 2H), 6.60 (d, *J* = 8.0 Hz, 1H), 6.48 (d, *J* = 8.0 Hz, 1H), 5.13(s, 2H), 2.62 (s, 3H). The ^1^H NMR spectrum was in line with the reference^38^.

### Synthesis of 2-(4-methoxyphenethyl)-4-oxo-4H-chromen-5-yl 4- ethynylbenzoate (15a) and 4-oxo-2-phenethyl-4H-chromen-5-yl 4-ethynylbenzoate (15b)

**CX-12** (20 mg, 0.068 mmol) and 4-acetylbenzoic acid (23.0 mg, 0.163 mmol, 2.4 equiv.) were dissolved in anhydrous DCM (3.0 ml) under N_2_ atmosphere. After stirred for 5 min, DCC (33.0 mg, 0.163 mmol, 2.4 equiv.) and DMAP (1.7 mg, 0.014 mmol, 0.2 equiv.) were added under lower than 30 °C, and the reaction was monitored by TLC. When the reaction was done, the addition of petroleum ether precipitated the DCU, and the filtrate was washed with saturated NH_4_Cl solution, water and brine in order, and the organic layer was evaporated under reduced pressure to afford the crude residue, which was purified by silica gel-based column chromatography with a mixed eluent of PE and EtOAc (V/V = 15:1) to afford 17.4 mg of **15a** as a light-yellow solid.

Yield, 62.0%. ^1^H NMR (400 MHz, Chloroform-*d*) δ 8.20 (d, *J* = 8.0 Hz, 2H), 7.71 − 7.59 (*m,* 3H), 7.38 (d, *J* = 8.0 Hz, 1H), 7.13-7.09 (*m*, 3H), 6.85-6.81 (*m*, 2H), 5.97 (*s*, 1H), 3.78 (*s*, 3H), 3.25 (*s*, 1H), 3.00-2.96 (*m*, 2H), 2.86-2.84 (*m*, 2H);^13^C NMR (100 MHz, CDCl_3_) δ 28.68, 30.96, 34.90, 54.23, 75.68, 76.00, 76.31, 79.08, 81.93, 110.23, 113.04, 115.22, 115.88, 118.13, 126.10, 128.18, 128.86, 129.24, 130.57, 131.16, 132.15, 148.26, 156.67, 157.23, 163.79, 166.41, 175.72. TOF-MS, *m/z*: [M + Na^+^], calcd. for C_27_H_20_NaO_5_^+^, 447.1203, found: 447.1207.

The synthetic procedure for **15b** was similar to that of **15a**, only difference in the starting martial **CX-12** replaced by **CX-6**.

**15b**, a light-yellow solid, yield, 33.0%.^1^H NMR (400 MHz, Chloroform-*d*) δ 8.20 (d, *J* = 8.0 Hz, 2H), 7.68 − 7.59 (*m*, 3H), 7.38 (d, *J* = 8.0 Hz, 1H), 7.31-7.19 (*m*, 6H), 7.12 (d, *J* = 8.0 Hz, 1H), 5.99 (*s*, 1H), 3.24 (*s*, 1H), 3.03-3.01 (*m*, 2H), 2.89-2.87 (*m*, 2H). TOF-MS, *m/z*: [M + Na^+^], calcd. for C_26_H_18_NaO_4_^+^, 417.1097, found: 417.1110.

### Synthesis of 2-(4-methoxyphenethyl)-4-oxo-4H-chromen-5-yl 4-(1-(6-((2- (2,6-dioxopiperidin-3-yl)-1,3-dioxoisoindolin-5-yl)oxy)hexyl)-1H-1,2,3-triazol-4-yl)benzoate (17a) and 4-oxo-2-phenethyl-4H-chromen-5-yl 4-(1-(6-((2–(2,6- dioxopiperidin-3-yl)-1,3-dioxoisoindolin-5-yl)oxy)hexyl)-1H-1,2,3-triazol-4-yl)benzoate (17b)

Under N_2_ atmosphere, to a DMF solution of **15a** (20 mg, 0.047 mmol) and **16** (28 mg,0.212 mmol, 1.5 equiv.) were added t-BuOH and an aqueous solution of NaVc (42 mg, 0.212 mmol, 4.5 equiv.) and Cu_2_SO_4_·5H_2_O (17.6 mg, 0.071 mmol, 1.5 equiv.). At 60 °C heated by ultrasonic wave, the reaction was stirred for 2.0 h. After filtered, the resulting solution was diluted with DCM, and the organic layer was washed with water and saturated brine, and dried by anhydrous Na_2_SO_4_, and filtered and dried under reduced pressure to afford the residue, which was purified by silica gel-based column chromatography with a mixed eluent of DCM and menthol (V/V = 100:1) to obtain 15.0 mg of **17a** as a light-yellow solid.

**17a**, yield, 56.7%. ^1^H NMR (400 MHz, CDCl_3_) δ 8.31(t, *J* = 8.0 Hz, 2H), 7.98 (d, *J* = 8.0 Hz, 2H), 7.68-7.66 (*m*, 2H), 7.45 (d, *J* = 8.0 Hz, 1H), 7.39 (d, *J* = 8.0 Hz, 1H), 7.20 (d, *J* = 8.0 Hz, 1H), 7.18-7.09 (*m*, 3H), 6.83 (d, *J* = 8.0 Hz, 2H), 5.99 (*s*, 1H), 4.95-4.91 (*m*, 1H), 4.48 (*t*, *J* = 8.0 Hz, 2H), 4.18-4.09 (*m*, 2H), 3.78 (*s*, 3H), 2.99-2.60 (*m*, 9H), 2.13-2.07 (*m*, 2H),1.91-1.87 (*m*, 2H), 1.47-1.29 (*m*, 4H);^13^C NMR (100 MHz, CDCl_3_) δ 14.34, 22.74, 22.82, 25.36, 25.97, 28.67, 29.83, 30.08, 31.56, 32.13, 36.07, 49.28, 55.40, 69.20, 76.32, 111.40, 114.20, 116.27, 117.16, 118.99, 119.42, 125.67, 129.35, 131.21, 131.79, 133.31, 133.92, 135.54, 149.59, 156.68, 157.84, 158.38, 165.29, 165.90, 167.17, 167.54, 168.27, 171.06, 176.98. TOF-MS, m/z: [M + H^+^], calcd. for C_46_H_42_N_5_O_10_^+^, 826.2926, found: 826.2927; [M + Na^+^], calcd. for C_46_H_41_N_5_NaO_10_^+^, 846.2746, found: 846.2747.

The synthetic procedure for **17b** was similar to that of **17a**, only difference in the starting martial **15a** replaced by **15b**.

**17b**, a light-yellow solid, yield, 43.1%. ^1^H NMR (400 MHz, DMSO-*d*_6_) δ 11.09 (s, 1H), 8.78 (*s*, 1H), 8.31 (*s*, 1H), 8.17 (d, *J* = 8.0 Hz, 2H), 8.07 (d, *J* = 8.0 Hz, 2H), 7.86-7.77 (*m*, 2H), 7.59 (d, *J* = 8.0 Hz, 1H), 7.50 (d, *J* = 8.0 Hz, 1H), 7.48 (d, *J* = 8.0 Hz, 1H), 7.30-7.26 (*m*, 5H), 7.21-7.19 (*m*, 1H), 6.07 (*s*, 1H), 5.10-5.06 (*m*, 1H), 4.47 (d, *J* = 8.0 Hz, 2H), 4.21(d, *J* = 8.0 Hz, 2H), 3.02-2.98 (*m*, 4H), 2.61-2.54 (*m*, 2H), 2.04-2.01 (*m*, 1H), 1.95-1.91 (*m*, 2H), 1.79-1.76 (*m*, 2H), 1.55-1.51 (*m*, 2H), 1.40-1.36 (*m*, 2H). TOF-MS, *m/z*: [M + H^+^], calcd. for C_45_H_40_N_5_O_9_^+^, 794.2821, found: 794.2834; [M + Na^+^], calcd. for C_45_H_39_N_5_NaO_9_^+^, 816.2640, found: 816.2653.

### Enzyme assay

The recombinant human CYP1B1 and 1A2 enzymes co-expressed with P450 reductase (Supersomes) were obtained from BD Biosciences, while 7-ethoxyresorufin, NADP^+^, glucose 6-phosphate (G-6-P), glucose-6-phosphate dehydrogenase (G-6-PD), and fatty acid-free BSA were sourced from Sigma-Aldrich and Shanghai Seebio Biotech. Enzyme inhibition assays were performed using a Thermo Scientific Varioskan Flash microplate reader with excitation and emission filters at respective 545 and 590 nm *via* the EROD method with 150 nM of 7-ethoxyresorufin and a NADPH regeneration system (1.3 mM NADP^+^, 3.3 mM G-6-P, 0.5 U/mL G-6-PD). Incubation times were optimised for each isoform CYP1B1 (35 min) and 1A2 (50 min), and IC_50_ values were calculated using Probit analysis in IBM SPSS Statistics V21. All other reagents were of analytical grade. Each assay was repeated in triple.

### Cell-based EROD assay

The MCF7 cell line was obtained from Chinese Academy of Sciences Cell Bank. CYP1B1 mutant-expressing MCF7 cells were generated by lentiviral transduction of mutant CYP1B1 cDNA (Fubio Biological Technology) followed by puromycin selection (2.5 μg/mL, 72 h). MCF7/DMBA cells were established by treating parental MCF7 cells with 5 μM DMBA (Maclin) for 72 h. All cell lines were cultured in RPMI-1640 medium (Thermo Fisher) supplemented with 10% foetal bovine serum (Thermo Fisher) at 37 °C in a 5% CO_2_ humidified incubator. The additional reagents included ANF (Maclin), DAPI, and MTT (Beyotime Biotechnology), with PBS (Thermo Fisher) used for washing steps. According to our previous procedure,^12^ each cell EROD assay was performed independently for three times. Statistical analysis was performed using Student’s t-test. Differences were considered statistically significant from the untreated group with a value of *P* < 0.05 with 95% confidence intervals.

### Western-blot and cytotoxicity assay

Immunoblot assay was conducted according to the standard protocol.^27^ Mouse monoclonal antibody to CYP1B1 (Proteintech, Cat. No. 67033-1-Ig) was used at a dilution of 1:6000, while mouse monoclonal antibody to β-actin (Proteintech, Cat. No. 66009-1-Ig) was applied at 1:10000 dilution. Both primary antibodies were prepared in western primary antibody dilution buffer. A horseradish peroxidase (HRP)-conjugated goat anti-mouse IgG (H + L) secondary antibody (Beyotime Biotechnology) was diluted 1:1000 in 5% BSA. Proteins were visualised with an enzyme-linked chemiluminescence detection kit. We performed the analysis using the Tanon 5200 Multi fully automated chemiluminescence/fluorescence imaging system, which features a Peltier-cooled CCD designed for deep cooling to enable long-exposure chemiluminescent detection. Images were acquired using the Tanon Bio-Imaging Software, which supports real-time capture of nucleic acid gels, protein gels, and chemiluminescent signals. The software features automatic exposure control with cumulative imaging capability, allowing both single long exposures and multi-frame time-lapse acquisition. Images can be selectively saved, and the system supports automated sequential imaging without the need for manual frame-by-frame storage. It also enables merged display and analysis of two images. In this experiment, a sequential exposure protocol was used with durations of 1 s, 3 s, 5 s, 10 s, 30 s, and 60 s. The optimal exposure times selected for CYP1B1 and β-Actin were 5 s each.

The *in vitro* cytotoxicity was evaluated using the standard MTT assay. The cells were seeded in 96-well plates at a density of 10,000 cells per well. After adhesion, the cells were treated with docetaxel, the test compounds, or a combination of docetaxel and CYP1B1 inhibitors for 72 h prior to MTT assessment. ANF (15 μM) was used as the negative control and no inhibition of cells was observed at the concentration of all the CYP1B1 inhibitors. IC_50_ values were calculated by Prism 5 (GraphPad, USA). Data were presented as mean ± SD from three independent experiments, each was performed in triplicate. Statistical significance was defined as *p* values <0.05 based on one-way ANOVA.

### Water solubility assay

The aqueous solubility was determined using a modified HPLC method based on previous literature,^33^ employing an Agilent Technologies 1260 Infinity II system with the following parameters: gradient elution with 150 mM ammonium acetate as A phase and methanol as B phase (seen in supplementary materials) at a flow rate of 0.2 ml/min, column temperature maintained at 35 °C, and detection wavelength set at 254 nm using an Agilent Eclipse Plus column (250 × 4.6 mm, 5 μm, No. USUXA26257). Calibration curves for **CX-6**, **CX-9**, **CX-22**, and ANF were prepared by dissolving 4 mg of each compound in 50 ml of DMSO, followed by serial dilution (5.0 ml diluted to 20 ml with DMSO, and then 5.0 ml adjusted for chromatographic analysis). The peak areas were used to construct the respective calibration curves. For solubility measurement, excess amounts of each compound were dissolved in 0.1 M phosphate buffer (0.2 ml, pH 7.4) and gently agitated at 25 °C for 24 h. The saturated solution was filtered through a 0.22 μm membrane, diluted (100 μL to 1.5 ml), and 10 μL was injected for HPLC analysis. The resulting peak areas were quantified using the corresponding calibration curves to determine the aqueous solubility values. Each measurement was repeated in triple.

### Molecule docking

Molecular docking was performed using AutoDock Vina 1.2.3 to study the binding of **CX-9** and reference ligand ANF to human CYP1B1 (PDB: 3PM0, 2.70 Å). The protein was preprocessed in AutoDock Tools 1.5.7 by removing crystallographic waters/co-crystallized ligands, adding polar hydrogens, assigning Gasteiger charges, and merging nonpolar hydrogens before conversion to PDBQT format. Ligands were prepared by constructing **CX-9** in ChemDraw (2D), converting it to 3D via Open Babel (–gen3d, –minimize), and optimising with ChemBio3D 14.0. Rotatable bonds and Gasteiger charges were assigned in AutoDock Tools and both ligands were exported as PDBQT files.

A cubic grid box of 24 Å × 24 Å × 24 Å centred on the ANF binding site was used to ensure full coverage of the binding pocket. Docking runs employed an exhaustiveness of 20 and a fixed random seed (10^9^) to ensure reproducibility. AutoDock Vina used an empirical scoring function to approximate ligand–receptor binding free energy and reported predicted affinities in kcal·mol^−1^. For each ligand, the lowest-energy pose (i.e., the conformation with the most negative predicted binding affinity) reported by Vina was selected for interaction analysis. Resulting poses were inspected and illustrated using PyMOL v2.5.2.

### Molecular dynamics simulation

Molecular dynamics (MD) simulations were performed using GROMACS 2025.3 with the CHARMM36m force field and TIP3P water model. The starting structure was human CYP1B1 (PDB: 3PM0, including haem). Two complexes were prepared *via* CHARMM-GUI:CYP1B1 bound to **CX-9** or the reference ligand ANF. Systems (∼50,000 atoms) were solvated in a periodic cubic box with ≥10 Å padding and neutralised with counterions.

Energy minimisation used steepest descent until F_max <1000 kJ mol^−1 ^nm^−1^. Equilibration consisted of 500 ps NVT followed by 500 ps NPT at 303.15 K and 1 bar, using the V-rescale thermostat and Parrinello-Rahman barostat. Production runs spanned 100 ns with a 2 fs timestep, employing LINCS constraints, PME for electrostatics (1.2 nm cut-off), and a 1.2 nm *van der Waals* cut-off with force-switching. Coordinates were saved every 100 ps. Trajectories were unwrapped for periodic boundary conditions and aligned to protein Cα atoms for analysis.

## Results and discussion

### Chemical synthesis

**Scheme 1.**
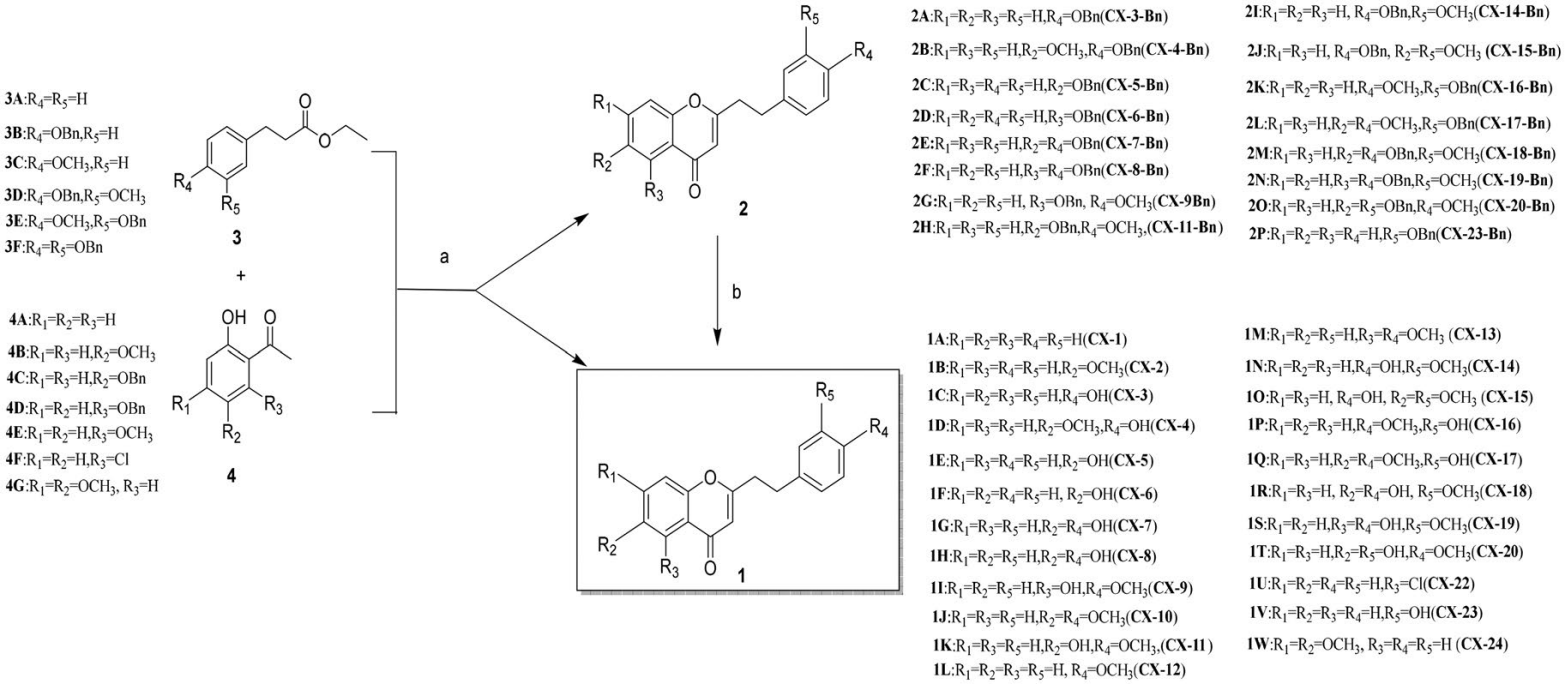
** ** A general synthetic route to 2–(2-phenylethyl) chromone derivatives (**CX-1 ∼ 20**, **CX-2 2 ∼ 24**). Conditions and agents: (a) i. NaH, DMF, 50 °C, 2 ∼ 8 h; ii. 10 M HCl, 90 °C, CH_3_OH, 0.5-2 h, yields, 42.6 ∼ 82.8%; (b) 10% Pd/C, H_2_, r.t, yields, 77.5 ∼ 96.8%.

**Scheme 2.**
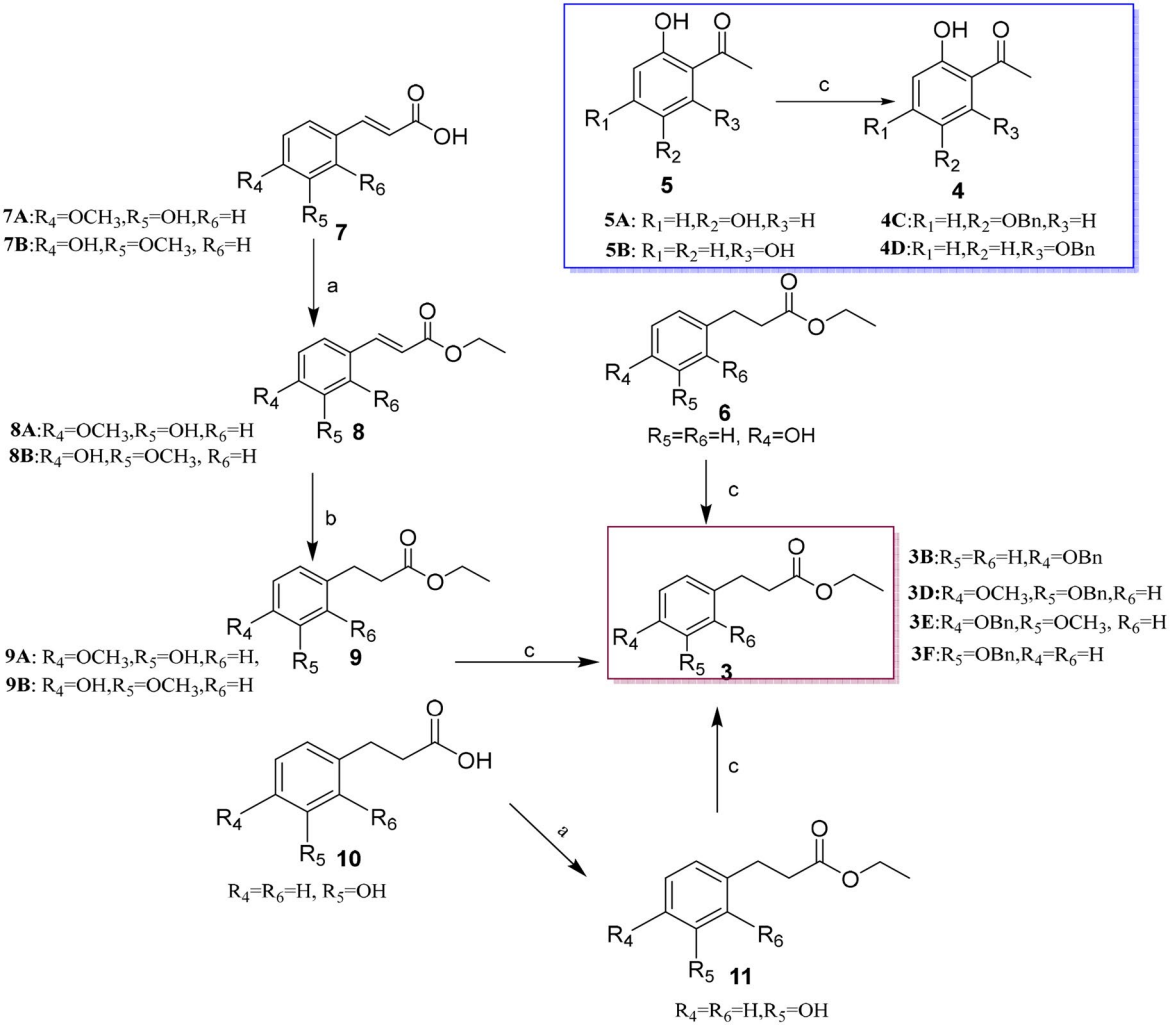
Synthesis of two types of key commercially unavailable intermediates. Conditions and agents: (a) i. CH_3_CH_2_OH, H_2_SO_4_, refluxing, 4 h; (b) 10% Pd/C, H_2_, r.t.; c) BnBr, anhydrous K_2_CO_3_.

**Scheme 3.**
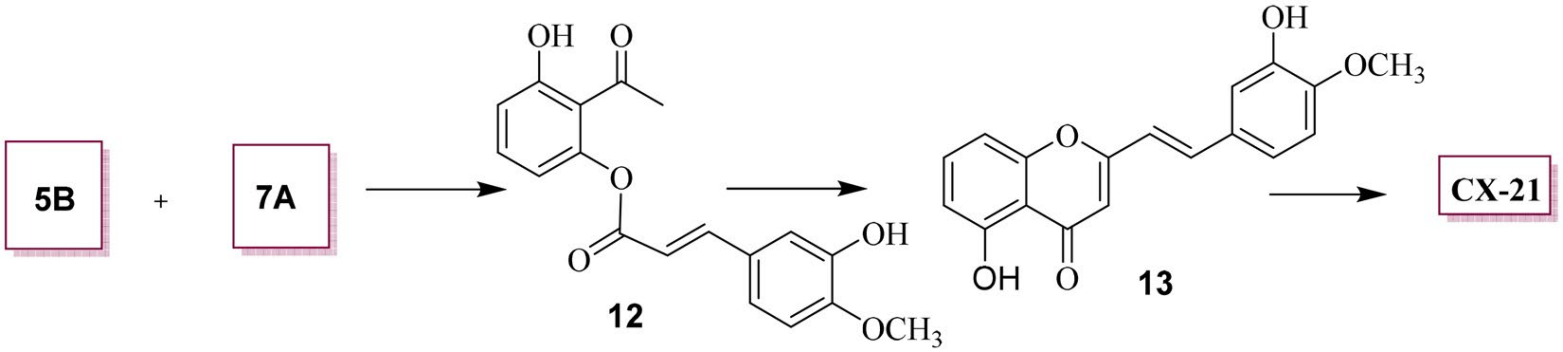
** ** A synthetic route to 2–(2-phenylethyl) chromone **CX-21**. Conditions and agents: (a) DCC, DMAP, dry DCM, r.t., 4h, yield 36.4%; (b) i. KOH, dry pyridine, 100 °C; ii. *p*-toluenesulfonic (PTS) acid, DMSO, 100 °C, 1h, yield, 60.5%; c) 10% Pd/C, H_2_, r.t, yield, 82.7%.

The synthesis of 2–(2-phenylethyl) chromone derivatives **CX-1** ∼ **24** was accomplished as outlined in Schemes 1 and 3. All the compounds but **CX-21** employed the classic Claisen condensation to construct the chromone core according to Lo SN’ synthetic method^29^. Key ketones **4 C**-**4D** were prepared *via* benzylation of commercial ortho-hydroxyarylalkylketones in the presence of bromobenzene and K_2_CO_3_^30^. Ester intermediates **3B** and **3D**-**3F** were synthesised from substituted ethyl phenylpropionate (**6**), cinnamic acids (**7 A**-**7B**), or phenylpropionic acid (**10**), respectively (Scheme 2). Acids **7 A**-**7B** were esterified by EtOH, hydrogenated, ^31^ and benzylated to yield **3D**-**3E**. Ethyl 3-(4-hydroxyphenyl) propanoate (**6**) was directly benzylated to afford **3B**, while the esterification and benzylation of acid **10** produced **3 F**.

Under basic conditions like NaH and DMF, the enolates derived from ketones (**4 A**-**4F**) reacted with carboxylic esters (**3 A**-**3G**), respectively, followed by 10 M HCl treatment using conventional heating, to afford the target 2–(2-phenylethyl) chromones (**CX-1 ∼ 2**, **CX-9** ∼ **10**, **CX-13**, **CX-22**) and benzylated derivatives (**2 A**-**2P**) in moderate yields^25^^,^^31^. Subsequent debenzylation of intermediates **2 A**-**2P** using 10% Pd/C yielded the final chromone derivatives bearing hydroxyl-, or methoxyl-substituents on ring A or B in excellent yields (77.5–96.8%) (Scheme 1). Notably differing from most 2–(2-phenylethyl) chromones, **CX-21** was synthesised *via* a three-step sequence: selective esterification of 2,6-dihydroxyacetophenone **5B** with isoferulic acid **7 A** catalysed by DCC and DMAP in a 36.4% yield, KOH mediated intramolecular Baker-Venkatamaran rearrangement of ester **12**, and final hydrogenation of intermediate **13** (Scheme 3)^31^. All target compounds and key intermediates were characterised by NMR and HRMS analyses.

**Scheme 4.**
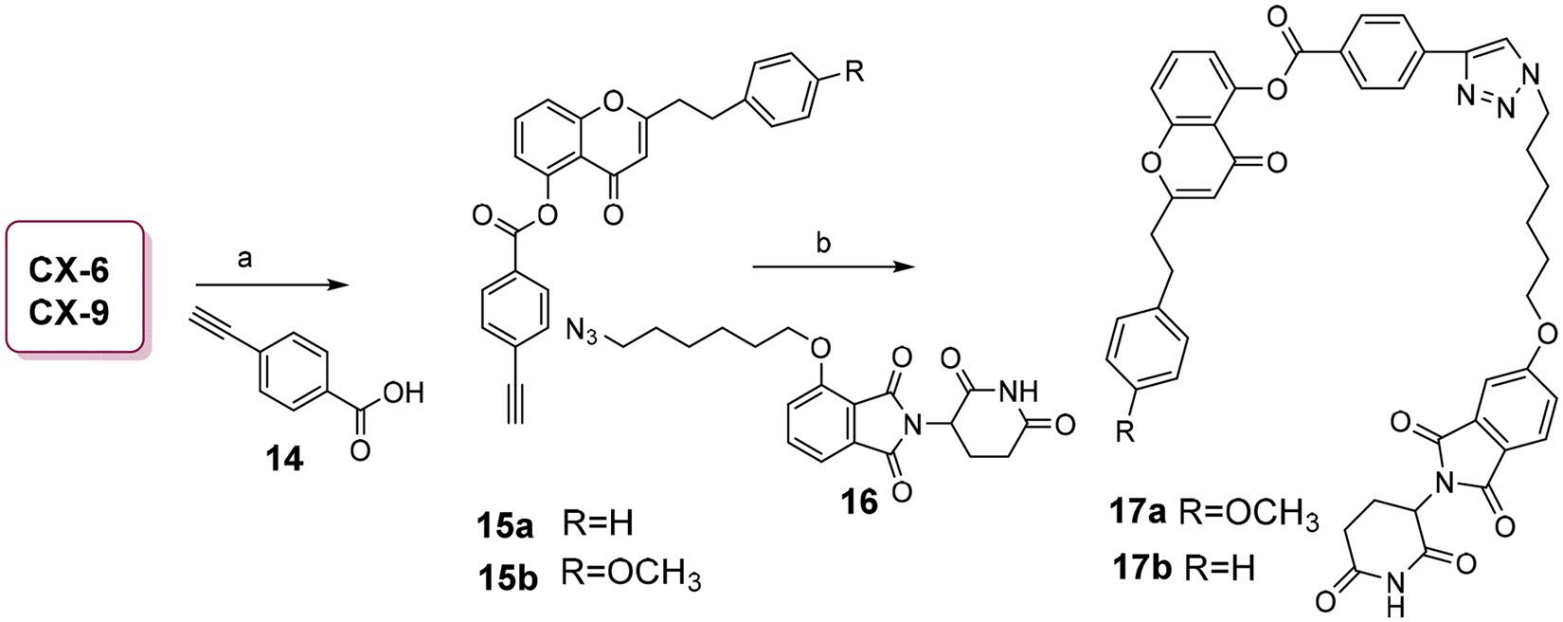
Synthesis of two chromone-based PROTACs **17a**-**17b**. Conditions and agents: (a) DCC, DMAP, **14**, r.t. 62.0% for **15a**, 33.0% for **15b**; (b) CuSO_4_.5H_2_O, **16**, NaVc, BuOH/H_2_O (V/V = 1/4), 60 °C heated by ultrasonic wave, 57.6% for **17a**, 43.1% for **17b**.

To validate chromone-based CYP1B1 targeting, we designed PROTACs **17a**-**17b** using chromone derivatives **CX-9** and **CX-6** as templates. Hydroxyl groups on these chromones were esterified with 4-ethynylbenzoic acid (**14**) via DCC and DMAP catalysis, yielding intermediates **15a**-**15b** in moderate yields. The E3 ligase-binding azide **16** was prepared as previously reported^36^. Final Cu^2+^-catalysed azide-alkyne cycloaddition between **15a**-**15b** and **16** under mild conditions furnished PROTACs **17a** (57.6%) and **17b** (43.1%), respectively^32^.

### Biological evaluation

#### Structure-activity relationships of 2–(2-phenylethyl) chromone analogues against CYP1B1 enzyme

The inhibitory activities of target compounds against recombinant human CYP1B1 and CYP1A2 were assessed using the standard EROD assay, a well-established method for evaluating CYP1 enzyme activity.^12^ ANF (HPLC purity >99%) served as the positive control. The enzymatic inhibitory potency of tested 2–(2-phenylehthyl) chromone compounds and ANF was displayed by IC_50_ values illustrated in Table 1.

**Table 1.** Inhibitory potency of 2–(2-phenylethyl) chromone derivatives (**CX-1** ∼ **24**) against CYP1B1 and 1A2 using the standard EROD assay^12,^^34^.

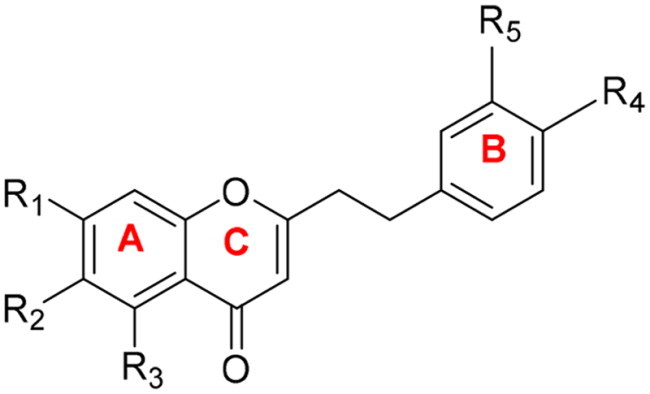								
No.	R1	R2	R3	R4	R5	CYP1B1 (IC50 μM)*	CY1A2 (IC50 μM)*	Ratio (IC50= CYP1A2/CYP1B1)
**CX-1**	H	H	H	H	H	0.88 ± 0.04	11.2 ± 0.21	12.7
**CX-2**	H	OCH_3_	H	H	H	0.33 ± 0.04	34.3 ± 1.34	103.9
**CX-3**	H	H	H	OH	H	2.65 ± 0.25	23.3 ± 2.03	8.8
**CX-4**	H	OCH_3_	H	OH	H	1.54 ± 0.05	16.7 ± 1.31	10.8
**CX-5**	H	OH	H	H	H	1.15 ± 0.32	8.3 ± 1.20	6.9
**CX-6**	H	H	OH	H	H	0.098 ± 0.005	23.1 ± 2.14	235.1
**CX-7**	H	OH	H	OH	H	0.20 ± 0.02	13.4 ± 2.12	67
**CX-7Bn**	H	OBn	H	OBn	H	0.084 ± 0.004	16.1 ± 1.23	191.7
**CX-8**	H	H	OH	OH	H	0.12 ± 0.01	23.7 ± 2.54	197.5
**CX-9**	H	H	OH	OCH_3_	H	0.075 ± 0.003	34.5 ± 2.78	460
**CX-9Bn**	H	H	OBn	OCH_3_	H	0.16 ± 0.04	28.3 ± 1.44	176.5
**CX-10**	H	OCH_3_	H	OCH_3_	H	1.91 ± 0.03	23.6 ± 1.05	12.2
**CX-11**	H	OH	H	OCH_3_	H	1.33 ± 0.05	17.8 ± 0.82	13.4
**CX-12**	H	H	H	OCH_3_	H	0.21 ± 0.05	43.6 ± 3.72	207.6
**CX-13**	H	H	OCH_3_	OCH_3_	H	6.4 ± 0.54	43.6 ± 4.12	6.8
**CX-14**	H	H	H	OH	OCH_3_	1.8 ± 0.05	26.8 ± 1.08	14.8
**CX-15**	H	OCH_3_	H	OH	OCH_3_	3.3 ± 0.22	17.4 ± 2.14	5.2
**CX-16**	H	H	H	OCH_3_	OH	4.5 ± 0.47	43.7 ± 3.06	9.7
**CX-17**	H	OCH_3_	H	OCH_3_	OH	3.8 ± 0.08	27.3 ± 0.94	7.2
**CX-17Bn**	H	OCH_3_	H	OCH_3_	OBn	0.62 ± 0.03	62.1 ± 3.54	100.2
**CX-18**	H	OH	H	OH	OCH_3_	5.80 ± 0.64	39.8 ± 3.65	6.8
**CX-19**	H	H	OH	OH	OCH_3_	3.06 ± 0.24	37.1 ± 3.14	12.1
**CX-19Bn**	H	H	OBn	OBn	OCH_3_	0.24 ± 0.05	25.3 ± 2.06	105.4
**CX-20**	H	OH	H	OCH_3_	OH	0.83 ± 0.03	48.1 ± 4.08	57.9
**CX-21**	H	H	OH	OCH_3_	OH	0.46 ± 0.03	76.9 ± 5.12	167.2
**CX-22**	H	H	Cl	H	H	0.052 ± 0.004	28.1 ± 1.46	540.4
**CX-23**	H	H	H	H	OH	3.80 ± 0.48	26.9 ± 1.73	7.1
**CX-24**	OCH_3_	OCH_3_	H	H	H	8.50 ± 0.83	48.9 ± 2.67	5.7
**17a**						20.7 ± 1.69	160.3 ± 5.78	7.7
**17b**						28.4 ± 1.87	186.6 ± 6.56	6.5
**ANF**						0.008 ± 0.002	0.010 ± 0.003	1.2

* All results are expressed as the mean ± SD, *n* = 3 for each compound.

All synthesised 2–(2-phenylethyl) chromone analogues showed CYP1B1 inhibition at micromolar concentrations, with CYP1A2 selectivity strongly dependent on ring A/B substitution patterns. Three derivatives exhibited exceptional potency: **CX-6** (R_3_-hydroxyl, IC_50_=0.098 ± 0.005 μM), **CX-9** (R_3_-hydroxyl, R_4_-methoxyl, IC_50_=0.075 ± 0.003 μM), and **CX-22** (R_3_-chloro, IC_50_=0.052 ± 0.004 μM), all demonstrating outstanding selectivity (SI > 230). Structure-activity analysis revealed key trends: (i) Methoxylation at R_2_ (**CX-2**) enhanced CYP1B1 inhibition more than 2-fold (IC_50_=0.33 ± 0.04 μM) versus parent **CX-1** (IC_50_=0.88 ± 0.04 μM) while improving selectivity (SI = 103.9), whereas R_4_-hydroxylation (**CX-4**, IC_50_=1.54 ± 0.05 μM) or methoxyl-to-hydroxyl conversion at R_2_ (**CX-5**, IC_50_=1.15 ± 0.32 μM) reduced both activity and selectivity; (ii) R_4_-hydroxylation (**CX-3**, IC_50_=2.65 ± 0.25 μM) decreased activity, while dihydroxylation (**CX-7**) unexpectedly enhanced inhibition; (iii) Benzylation dramatically boosted potency and selectivity, e.g., **CX-7Bn**, IC_50_=0.084 ± 0.004 μM, SI = 191.7, though at the cost of water solubility. This benzylation effect was consistently observed across multiple derivatives, with **CX-19Bn** (IC_50_=0.24 ± 0.05 μM) demonstrating 12.8-fold greater potency than **CX-19** (IC_50_=3.06 ± 0.24 μM), and **CX-17Bn** (IC_50_=0.62 ± 0.03 μM) showing 6.1-fold enhanced activity compared to **CX-17** (IC_50_=3.8 ± 0.08 μM). The enhanced activity and selectivity of the introduced benzyl group may be attributed to its hydrophobicity, which favours binding to the active centre, and its ability to form π–π interactions, thereby improving affinity. Collectively, these findings highlight subtle modifications, particularly at R_3_ of ring A and R_4_ of ring B, profoundly impact CYP1B1 inhibition and selectivity.

**Figure 2.**
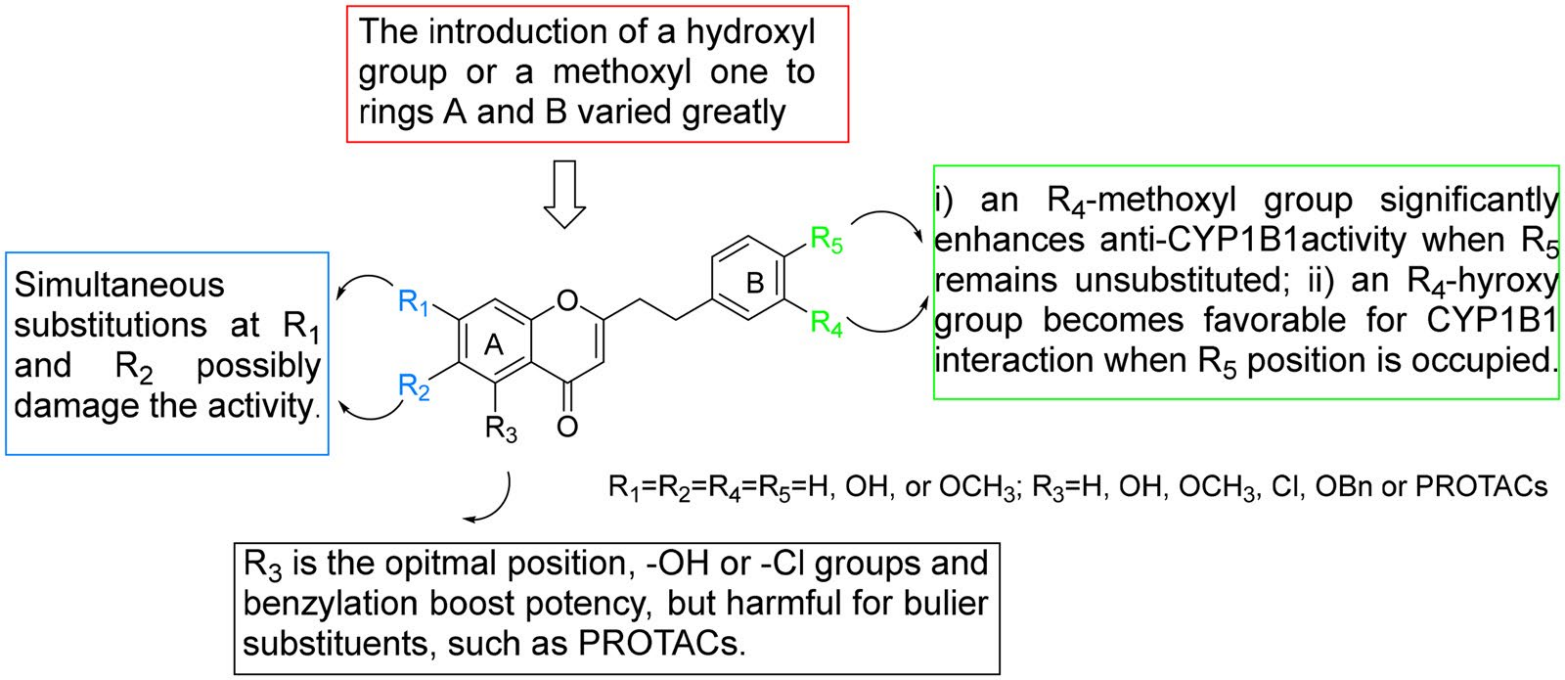
Structure–activity relationship (SAR) of 2–(2-phenylethyl) chromone derivatives against CYP1B1 activity.

Due to **CX-6**’s high potency (IC_50_=0.098 ± 0.005 μM), we took it as the second template compound for further modification. Introducing R_4_-hydroxyl group to **CX-6**, forming **CX-8**, reduced activity >2-fold. However, the benzyloxyl group at R_3_ and methoxyl group introduced at R_4_ (**CX-9Bn**) showed no loss of activity or selectivity compared to **CX-8**, and the removal of the hydroxyl group at R_3_ from **CX-8 (CX-12**) resulted in comparable performance. We conclude that R_3_ is the optimal site for modification. This was further support by **CX-22**’s maintained activity and enhanced selectivity through halogen substitution. These outcomes indicated that the R_3_ position has appropriate space to accommodate interactions with CYP1B1 residues. Additional modifications at R_5_ (**CX-19**, IC_50_=3.06 ± 0.24 μM; **CX-21**, IC_50_=0.46 ± 0.03 μM) or R_3_-to-R_5_ hydroxyl migration (**CX-23**, IC_50_=3.80 ± 0.48 μM) significantly decreased activity, possibly implying R_5_ substitution harmful to anti-CYP1B1 activity. Notably, R_4_-hydroxyl/R_5_-methoxyl combinations (**CX-14**/**CX-15**) outperformed their positional isomers (**CX-16**/**CX-17**), potentially due to interactions with CYP1B1’s haem group during biocatalysis. Contrary to expectations, dual R_1_/R_2_- methoxylation failed to enhance activity, and the increase in electron density from the two methoxyl groups had no effect on CYP1B1 binding, diverging from ANF’s structure-activity profile,^12^ These results systematically map critical structural determinants for optimising chromone-based CYP1B1 inhibitors (Figure 2). R_3_ is the optimal modification site, where -OH or -Cl groups and benzylation boost potency. Conversely, R_5_ substitution, R_4_-hydroxylation, or R_1_/R_2_-dimethoxylation reduces activity.

As reported previously^34^^,^^37^, rational structural modifications enabled fluorescence probes to track CYP1B1 expression/distribution and degraders to eliminate CYP1B1. Inspired by this, we employed click chemistry to synthesise dual-functional molecules **17a**-**17b** for CYP1B1 recognition and degradation. Compared to precursors **CX-6** and **CX-9**, **17a** (IC_50_=20.7 ± 1.69 μM) and **17b** (IC_50_=28.4 ± 1.87 μM) showed markedly reduced anti-CYP1B1 activity, indicating bulkier R_3_ substituents (*vs.* benzyl group) impair function. Contrary to our prior findings^34^^,^^37^, the ring A modification at R_3_ compromised fluorescence probe development.

#### Evaluation of the drug resistance due to overexpressed CYP1B1 enzyme

**Figure 3.**
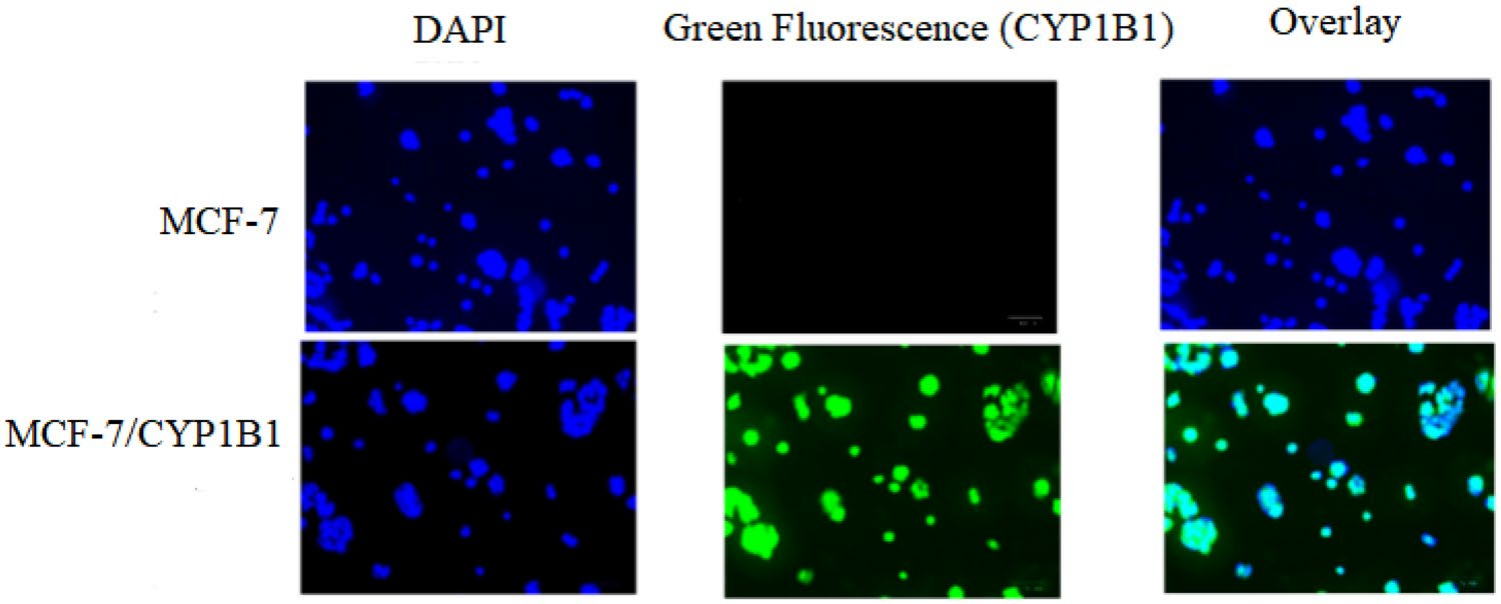
Construction of MCF-7/*cyp1b1* cell lines *via* MCF-7 infected lentivirus carrying *cyp1b1* and green fluorescence. (MCF-7 cells and MCF-7/*cyp1b1* cells were all nuclear stained by DAPI for 15 min and washed by PBS for three times. Then, they were observed and photographed under the fluorescence microscope. Blue fluorescence indicates DAPI nuclear staining, Green fluorescence indicates CYP1B1 mutant expressing.).

**Figure 4.**
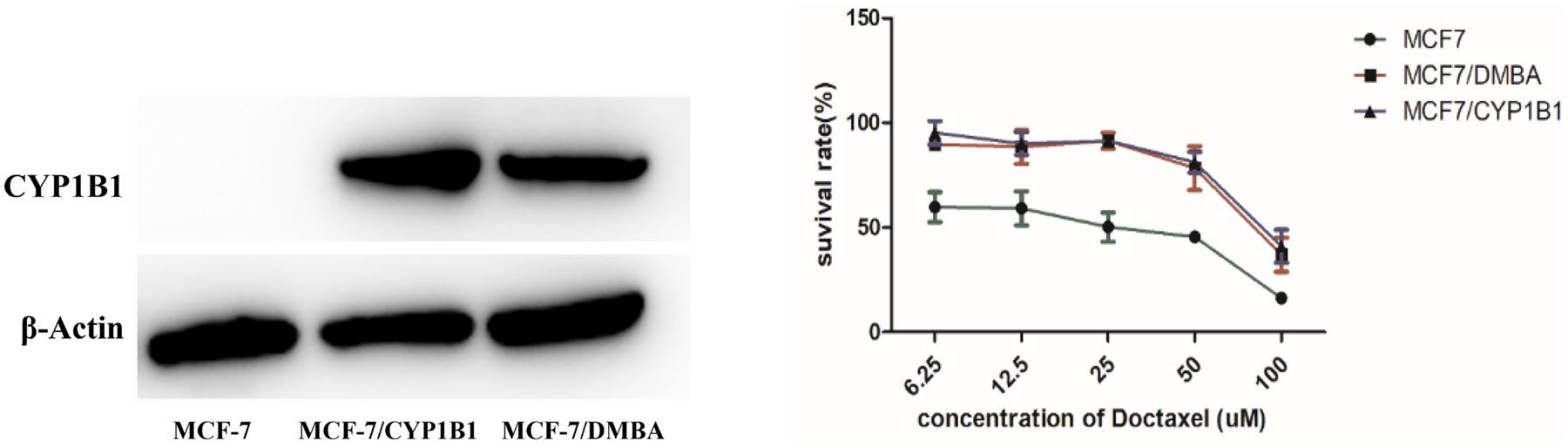
Expression of CYP1B1 enzyme detected by western-blotting in MCF-7, MCF-7/DMBA and MCF-7/*cyp1b1* cell lines; Survival curve of cells treated with docetaxel.

Previous studies have demonstrated that CYP1B1 overexpression in MCF-7 cells confers resistance to docetaxel^33^, and its expression can be induced by 2,3,7,8-tetrachlorodibenzo-*p*-dioxin (TCDD) ^12^ or dimethylbenzanthracene (DMBA)^34–36^. To investigate this mechanism, we established CYP1B1-overexpressing MCF-7 cell lines using two approaches. Lentiviral transduction was employed to generate MCF-7/*cyp1b1* cells, which exhibited green fluorescence (Figure 3), confirming successful infection. Additionally, MCF-7 cells were treated with 5 μM DMBA for 72h to induce CYP1B1 expression (MCF-7/DMBA). Western blot analysis confirmed CYP1B1 overexpression in both cell lines, with recombinant human CYP1B1 as a positive control. In contrast, parental MCF-7 cells showed no detectable CYP1B1 expression (Figure 4).

The docetaxel (DOC) resistance of the engineered cell lines was subsequently evaluated. Both MCF-7/*cyp1b1* and MCF-7/DMBA exhibited significantly higher IC_50_ values compared to parental MCF-7 cells, confirming drug resistance (Figure 4). Specifically, the IC_50_ values of DOC were 90.7 ± 7.5 μM for MCF-7/*cyp1b1* and 86.0 ± 8.0 μM for MCF-7/DMBA, whereas MCF-7 cells showed an IC_50_ of 24.0 ± 5.6 μM. These results validate the successful construction of CYP1B1-overexpressing cell models for resistance evaluation. The consistent elevation in IC_50_ values between lentiviral-transduced and DMBA-induced cells further supported the role of CYP1B1 in docetaxel resistance, aligning with previous findings.^12^

#### Chromone derivatives reverse the drug-resistance against docetaxel

To verify chromones’ resistance-reversal capability, we evaluated three derivatives **CX-6**, **CX-9** and **CX-22** for synergistic effects with docetaxel in CYP1B1-overexpressing MCF-7 cells using EROD assays. These model compounds were selected for their superior CYP1B1 inhibition, with ANF as positive control. Since aqueous solubility critically influences cellular activity, we determined these properties that all three chromones showed excellent solubility (>100 μM) and no cytotoxicity (IC_50_>100 μM) in both MCF-7/*cyp1b1* and MCF-7/DMBA cells, enabling testing below 100 μM (Table 2). In contrast, ANF exhibited poor solubility and significant toxicity above 20 μM (Table 2).

**Table 2.** The solubility and cytotoxicity of three chromone derivatives and ANF.

	ANF	CX-6	CX-9	CX-22
Solubility (μM)^a^	<20	>100	>100	>100
IC_50_ values (μM)	>20	>100	>100	>100

^a^ indicates the water solubility determined by HPLC

**Figure 5.**
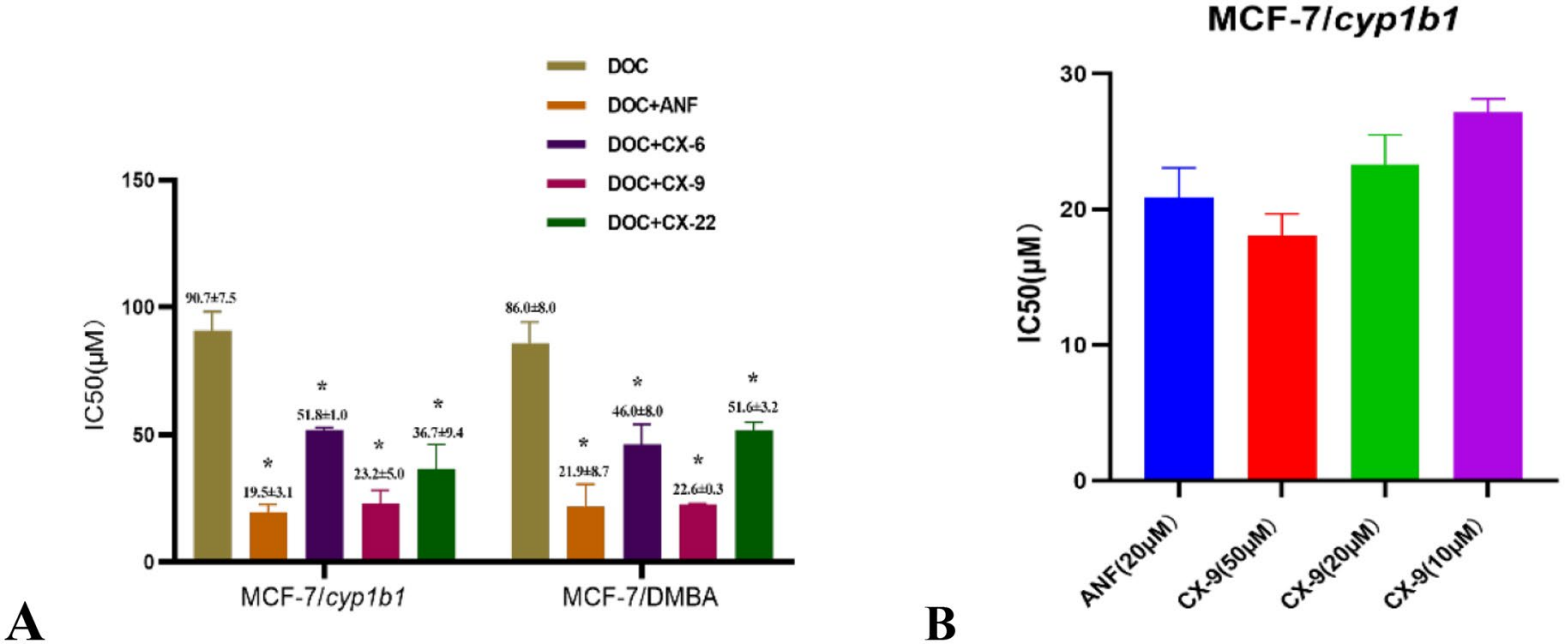
Reversal of drug resistance by chromones in CYP1B1-overexpressing cell lines. (A. Three chromones reversed drug resistance of MCF-7/c*yp1b1* and MCF-7/DMBA cell lines to docetaxel (DOC); B. **CX-9** reversed MCF-7/*cyp1b1* resistant to DOC dose-dependently.). The data were expressed as the mean ± SD (*n* = 3). *p < 0.05 compared with the docetaxel-treating cells.

In resistance reversal assays, ANF (20 μM) effectively reduced docetaxel’s IC_50_ values from 90.7 ± 7.5 μM to 19.5 ± 3.1 μM (MCF-7/*cyp1b1*) and from 86.0 ± 8.0 μM to 21.9 ± 8.7 μM (MCF-7/DMBA). All the three tested chromone derivatives showed reversal activity, with **CX-9** being most potent (IC_50_=23.2 ± 5.1 μM against MCF-7/*cyp1b1* and 22.6 ± 0.3 μM against MCF-7/DMBA, Figure 5(A) and Table 3). Notably, **CX-9**’s reversal effect was concentration-dependent, achieving comparable efficacy at 50 μM of ANF (Figure 5(B)), demonstrating its superior therapeutic potential.

**Table 3.** The IC_50_ values of two CYP1B1-overexpressing cell lines in different treatments.

	IC_50_ values (μM)				
	DOC	DOC + ANF	DOC +**CX-6**	DOC +**CX-9**	DOC+**CX-22**
MCF-7	24.0 ± 5.6	/	/	/	/
MCF-7/*cyp1b1*	90.7 ± 7.5	19.5 ± 3.1	51.8 ± 1.0	23.2 ± 5.1	36.7 ± 9.4
MCF-7/DMBA	86.0 ± 8.0	21.9 ± 8.7	46.0 ± 8.0	22.6 ± 0.3	51.6 ± 3.2

#### Molecular docking and molecular dynamics (MD) simulation

**Figure 6.**
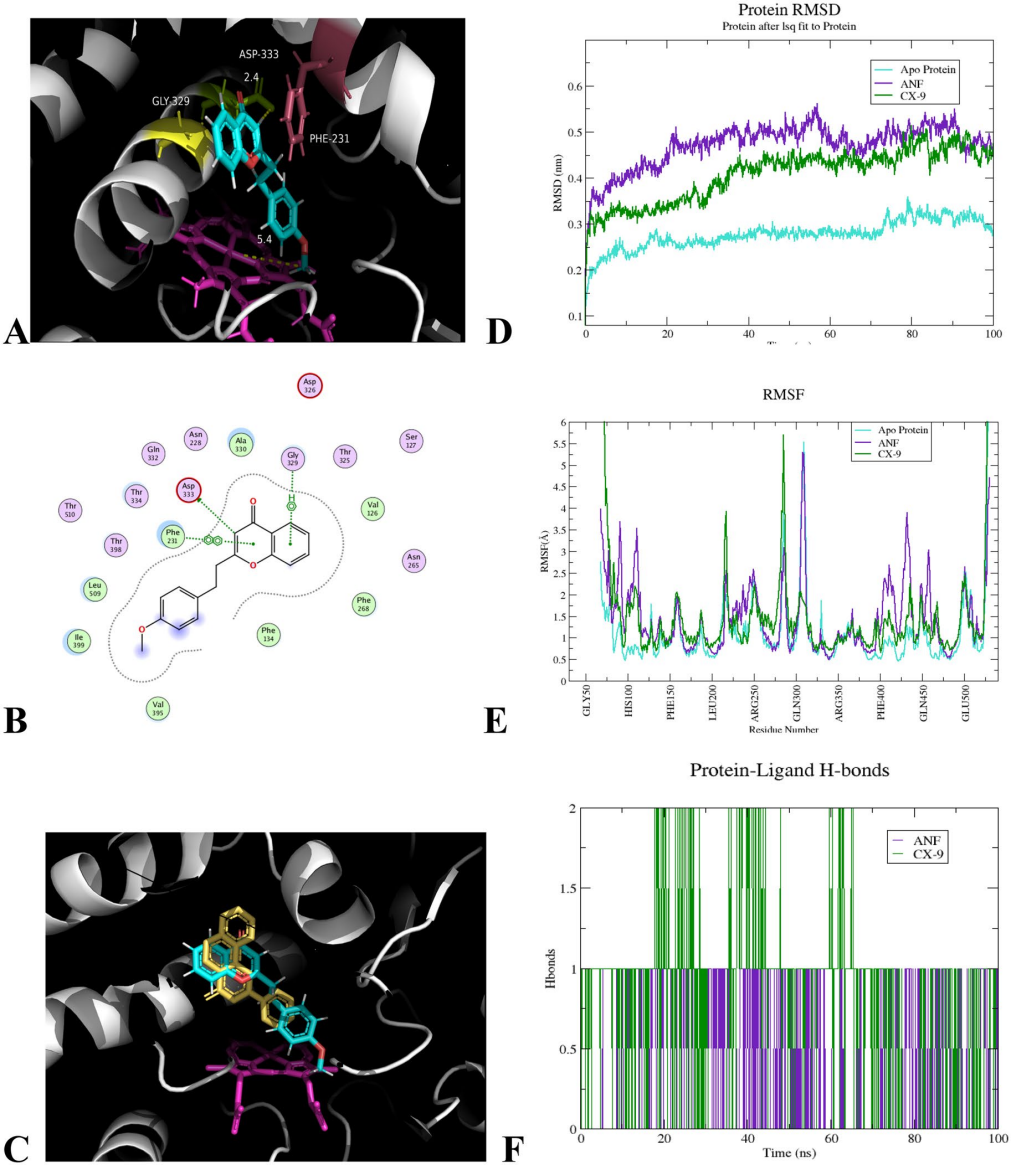
Molecule docking and molecular dynamics simulation of CYP1B1 (PDB ID: 3PM0) and **CX-9** and ANF, respectively. (A. Docking pose of **CX-9** (green sticks) in the CYP1B1-ANF complex. Phe^231^ (purple), the haem prosthetic group (violet), and Gly^329^ (yellow) are shown. B. 2D docked model of **CX-9** into CYP1B1, hydrogen bonds of Asp^333^, Phe^231^ and Gly^329^ are shown by dashed lines. C. Superimposed structures of CYP1B1 enzyme bound to **CX-9** and ANF. D. Protein RMSD, backbone RMSD (fit to protein) over 100 ns for apo (cyan), ANF (purple), and **CX-9** (green). E. RMSF by residue. RMSF (Å) highlighting B-C (∼110–130), I-helix/SRS-4 (∼290–370), F-G (∼370–410), and haem-binding (∼430-470) regions. F. Protein-ligand hydrogen bonds. Instantaneous hydrogen-bond counts using a 0.35 nm/135° geometric criterion.).

Given the similar CYP1B1 inhibition potency of **CX-9** and ANF in MCF-7 cells, we investigated their binding modes with CYP1B1 (PDB ID: 3PM0). Molecular docking revealed **CX-9**’s lowest-energy pose with predicted affinity −7.4668 *k*cal·mol^−1^ (Figure 6(A)) forms key hydrogen bonds with Asp^333^, Phe231 and Gly329 (Figure 6(B)), respectively, stabilising its position. The ligand’s aromatic system engages in π-π stacking (face-to-face/edge-to-face) with Phe231 at 3.61 Å, replicating ANF’s interaction pattern. Hydrophobic residues Val395, Leu509, Ile399 further stabilise **CX-9** through *van der Waals* interactions in the nonpolar pocket. PyMOL-based structural alignment demonstrated **CX-9**’s aromatic rings spatially overlap with ANF’s in the binding pocket (Figure 6(C)), while planar structures and polar group arrangements show strong pharmacophore conservation.

Analysis of 100-ns MD simulations of CYP1B1 in Apo, ANF-bound, and **CX-9**-bound states revealed distinct conformational dynamics. All systems equilibrated within ∼20 ns. The backbone RMSD of the **CX-9** complex (0.40 ± 0.02 nm) was lower than that of the ANF complex (0.48 ± 0.02 nm) (Figure 6(D)), indicating **CX-9** induces smaller global rearrangements. Similarly, the radius of gyration for (Rg) **CX-9** (2.29 ± 0.01 nm) closely matched the apo state (2.27 ± 0.01 nm), while ANF caused a clear expansion (2.36 ± 0.01 nm) (Figure S85 in Supplementary Materials), suggesting a more loosely packed catalytic domain. Residual fluctuation (RMSF) analysis showed ANF increased CYP1B1’s flexibility in key loops (the B-C loop (residues 110–130), I-helix/SRS-4 (290–370), and F-G loop (370–410)) by 0.1–0.2 nm, whereas **CX-9** suppressed fluctuations near active-site helices by 0.05–0.1 nm, more closely resembling the Apo state (Figure 6(E)). The haem-binding region remained rigid across all systems. Ligand stability analysis and Fe–ligand centre-of-mass distance further confirmed that **CX-9** exhibited lower RMSD (0.10–0.18 nm) and maintained a closer, stable distance to the haem iron (1.1–1.8 nm), while ANF showed greater positional variation (RMSD 0.25–0.35 nm; Fe-distance 1.8–2.5 nm) (Figure S86–87). Both ligands formed few transient hydrogen bonds ((Figure 6(F)), with stabilisation primarily mediated by π-π stacking with Phe^231^ and hydrophobic interactions with residues such as Val395, Leu509, and Ile399.

Taken together, these results from analysis of molecular docking and MD simulation demonstrated **CX-9**’s effective binding site occupation and structural mimicry of ANF, explaining its comparable even superior activity, while **17a**-**17b**’s modified E3 ligase-recognizing moiety maybe disrupt the key interactions and then abolished anti-CYP1B1 function.

## Conclusion

In this study, we synthesised twenty-four 2–(2-phenylethyl) chromone derivatives, assessed their anti-CYP1B1 activity that is implicated in clinical anti-tumour drug resistance and selectivity towards tissue-distributed CYP1A2, and established structure-activity relationships for ring A/B substituents. Notably, **CX-6**, **CX-9**, and **CX-22** exhibited the strongest anti-CYP1B1 effects with high selectivity. In CYP1B1-overexpressing cell models, **CX-9** showed dose-dependent docetaxel resistance reversal, matching 20 μM ANF’s efficacy at 50 μM. Molecular docking and molecular dynamics simulation confirmed similar binding modes of **CX-9** and **ANF** within CYP1B1’s active cavity. The tested active derivatives demonstrated >100 μM solubility and no cytotoxicity. These findings hint a novel natural-derived scaffold, 2–(2-phenylethyl) chromone, for developing promising CYP1B1 inhibitors.

## Supplementary Material

Graphic Abstract.doc

supporting information（revision）.pdf

## Data Availability

Author confirm that the data supporting the findings of this study within the article and its Supplementary Materials
