# Supplementary material for: Discovery of 2-phenylethyl chromones as potent and selective CYP1B1 inhibitors
Source: J Enzyme Inhib Med Chem. 2026 Jan 2;41(1):2598738. doi: 10.1080/14756366.2025.2598738 (PMC12777840; doi:10.1080/14756366.2025.2598738)
Supplement: Graphic Abstract.doc [file IENZ_A_2598738_SM3802.doc]

**Graphic abstract**

**Discovery of 2-phenylethyl chromones as potent and selective CYP1B1 inhibitors**

Wenming Chen^1,3#^, Wenchong Ye^2,4^, Yinghong Long^1,3^, Ye Zhang^5^, Wen Zhou2,4[[1]](#footnote-2)*, Wei Wang1,3[[2]](#footnote-3)*

*^1^TCM and Ethnomedicine Innovation & Development International Laboratory, Innovative Materia Medica Research Institute, School of Pharmacy, Hunan University of Chinese Medicine, Changsha, Hunan, 410208, P.R. China*

*^2^ Shanghai Veterinary Research Institute, Chinese Academy of Agricultural Sciences, Shanghai, 200241, P.R. China.*

*^3^Department of Pharmaceutical Products Center, The First Hospital of Hunan University of Chinese Medicine, 95, Shaoshan* *Rd, Changsha, Hunan, 41007, P.R.China.*

*^4^China Key Laboratory of Veterinary Chemical Drugs and Pharmaceutics, Ministry of Agriculture and Rural Affairs, Shanghai Veterinary Research Institute, Chinese Academy of Agricultural Sciences, Shanghai, 200241, P.R. China*

*^5^Department of Chinese Medicine, Shenzhen Futian District Maternal and Child Health Hospital, Shenzhen, Guangdong, 518000, P.R. China*

Tumor-selective CYP1B1 inhibitors were developed using novel 2-(2-phenylethyl) chromones.

**CX-9** showed nanomolar potency and reversed docetaxel resistance (50 μM comparable to 20 μM ANF), providing this scaffold for selective inhibitor development.

1. ^^ Correspondent. E-mail: zhouwen60@126.com [↑](#footnote-ref-2)
2. ^^ Correspondent. E-mail: wangwei402@hotmail.com [↑](#footnote-ref-3)
