# Supplementary material for: Discovery of 2-phenylethyl chromones as potent and selective CYP1B1 inhibitors
Source: J Enzyme Inhib Med Chem. 2026 Jan 2;41(1):2598738. doi: 10.1080/14756366.2025.2598738 (PMC12777840; doi:10.1080/14756366.2025.2598738)
Supplement: supporting information（revision）.pdf [file IENZ_A_2598738_SM3801.pdf]

Discovery of 2-phenylethyl chromones as potent and selective CYP1B1 inhibitors

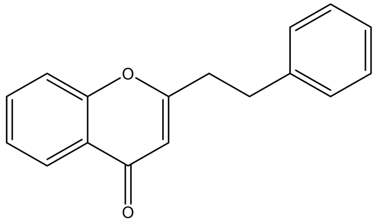

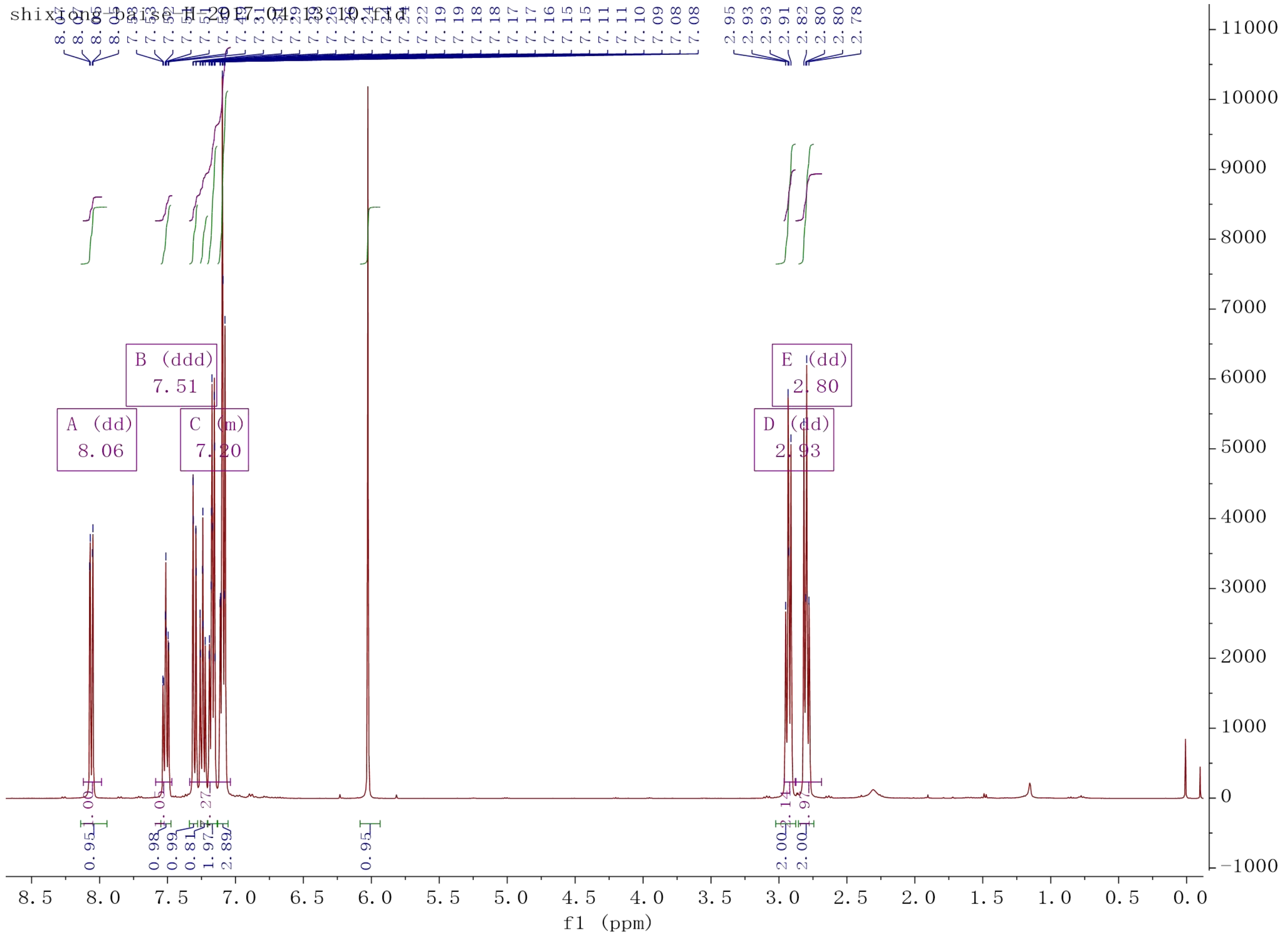

Fig.S1 ¹HNMR of 2-phenethyl-4H-chromen-4-one (400MHz, $CDCl_3$)

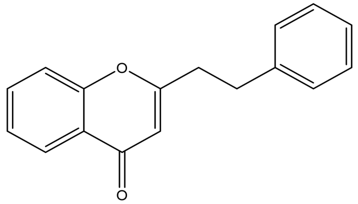

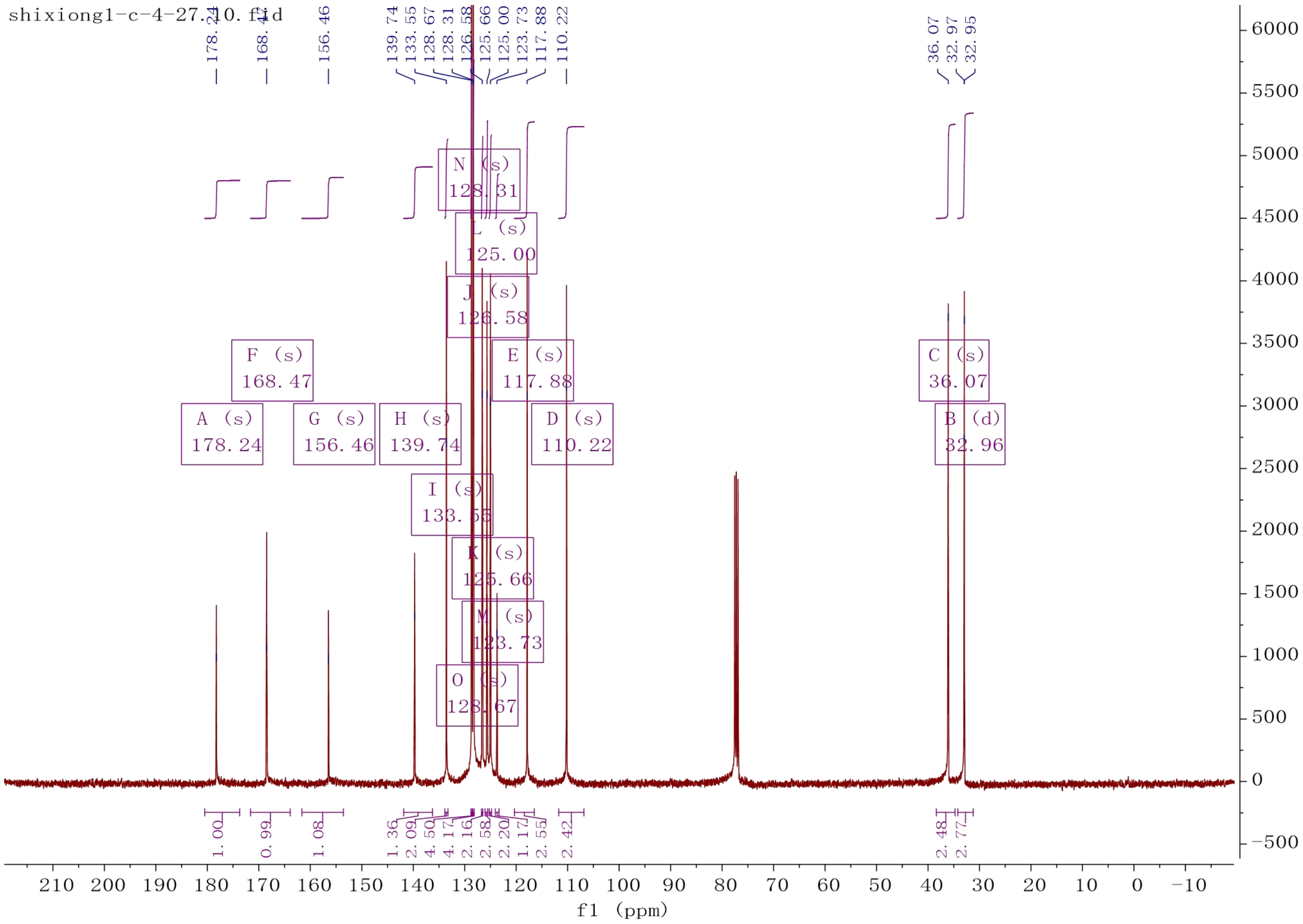

Fig.S2 ^{13}CNMR of 2-phenethyl-4H-chromen-4-one (100MHz, $CDCl_3$)

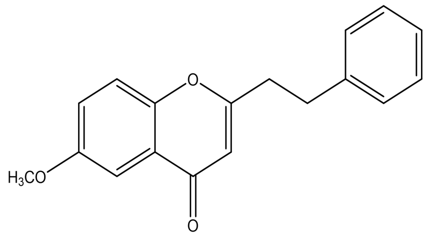

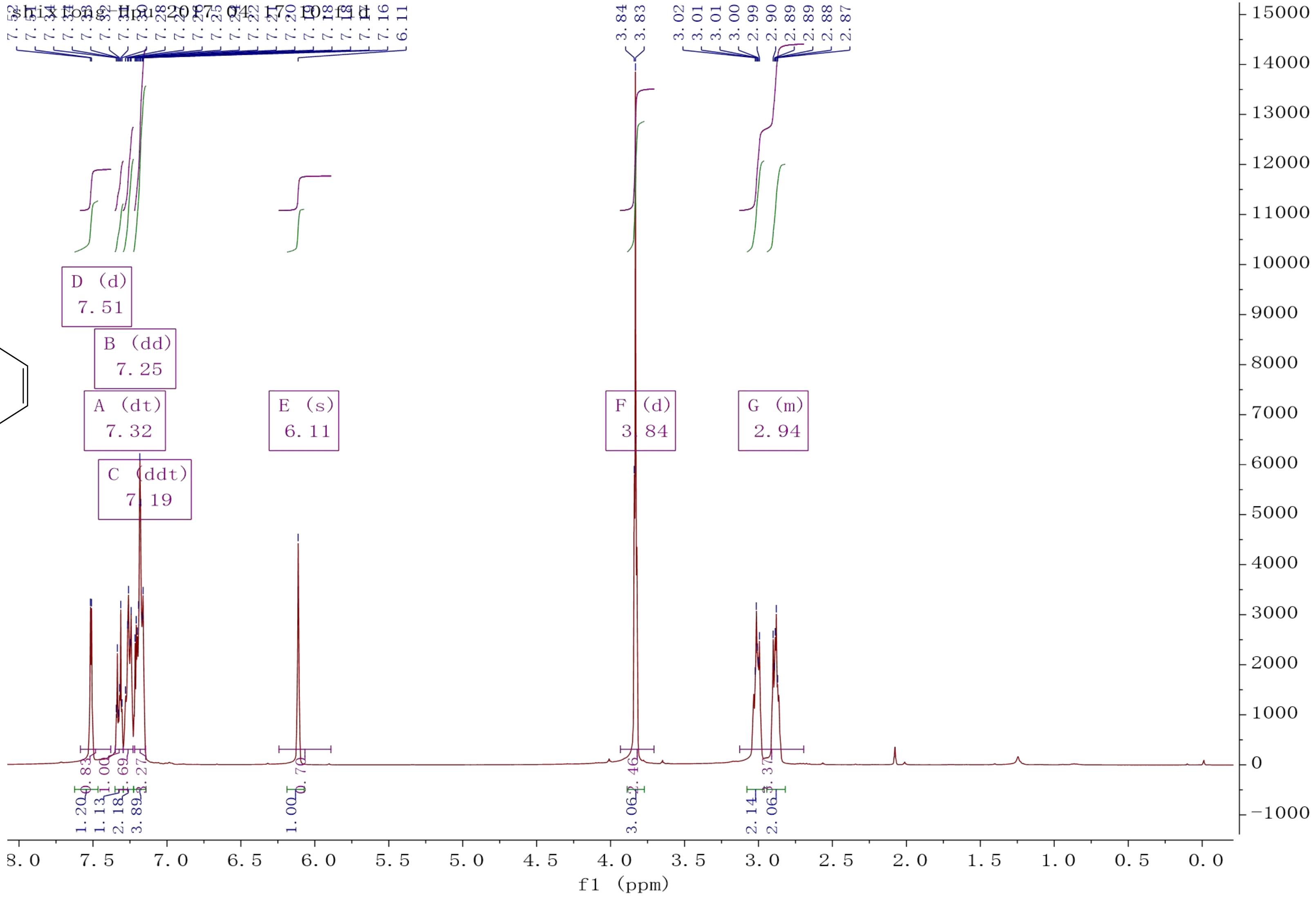

Fig.S3 ^{1}HNMR of 6-methoxy-2-phenethyl-4H-chromen-4-one (400MHz, $CDCl_3$)

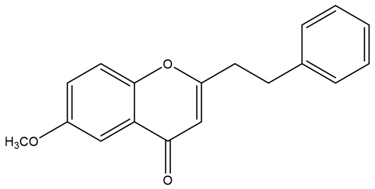

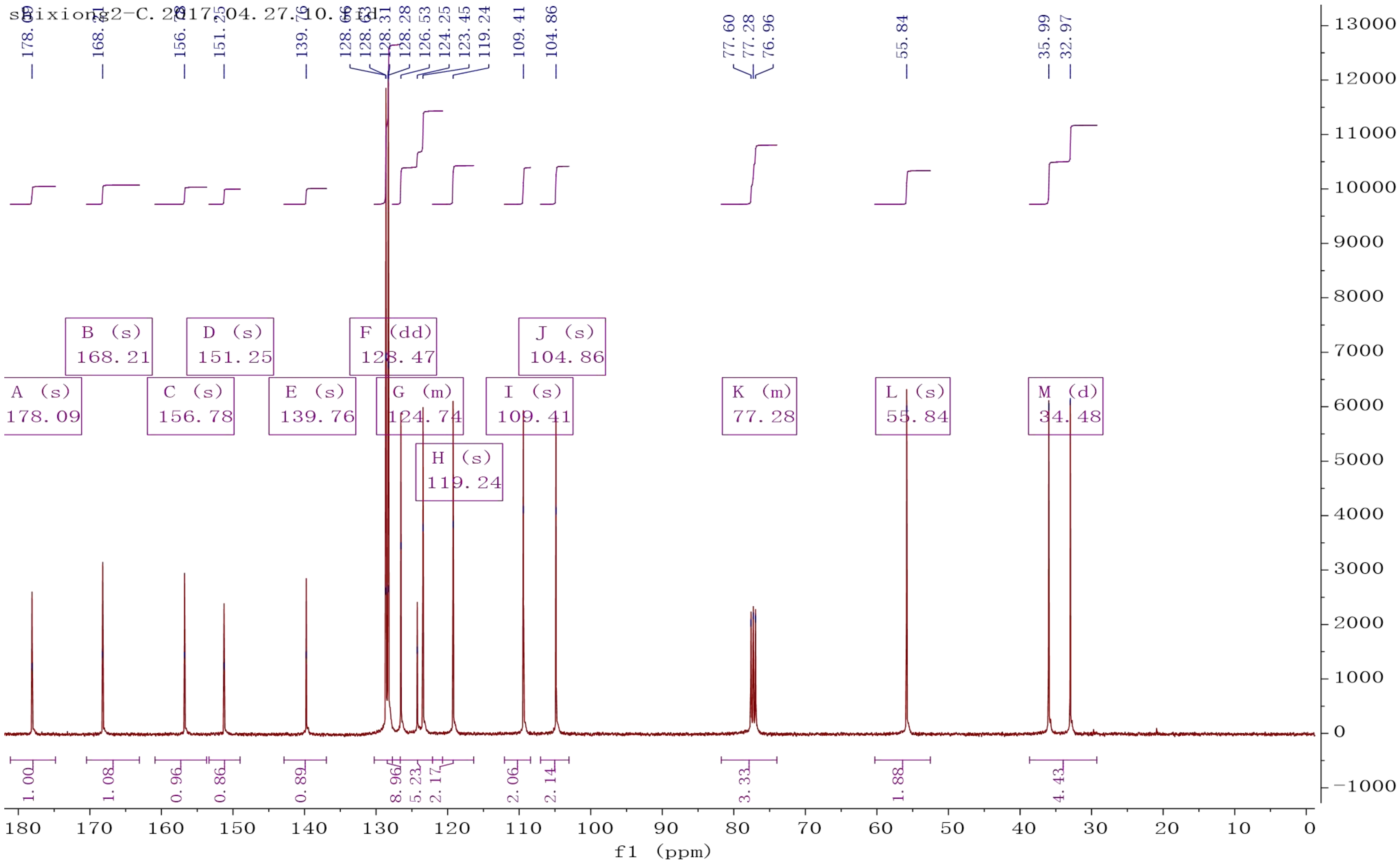

Fig.S4 13CNMR of 6-methoxy-2-phenethyl-4H-chromen-4-one (100MHz, $CDCl_3$)

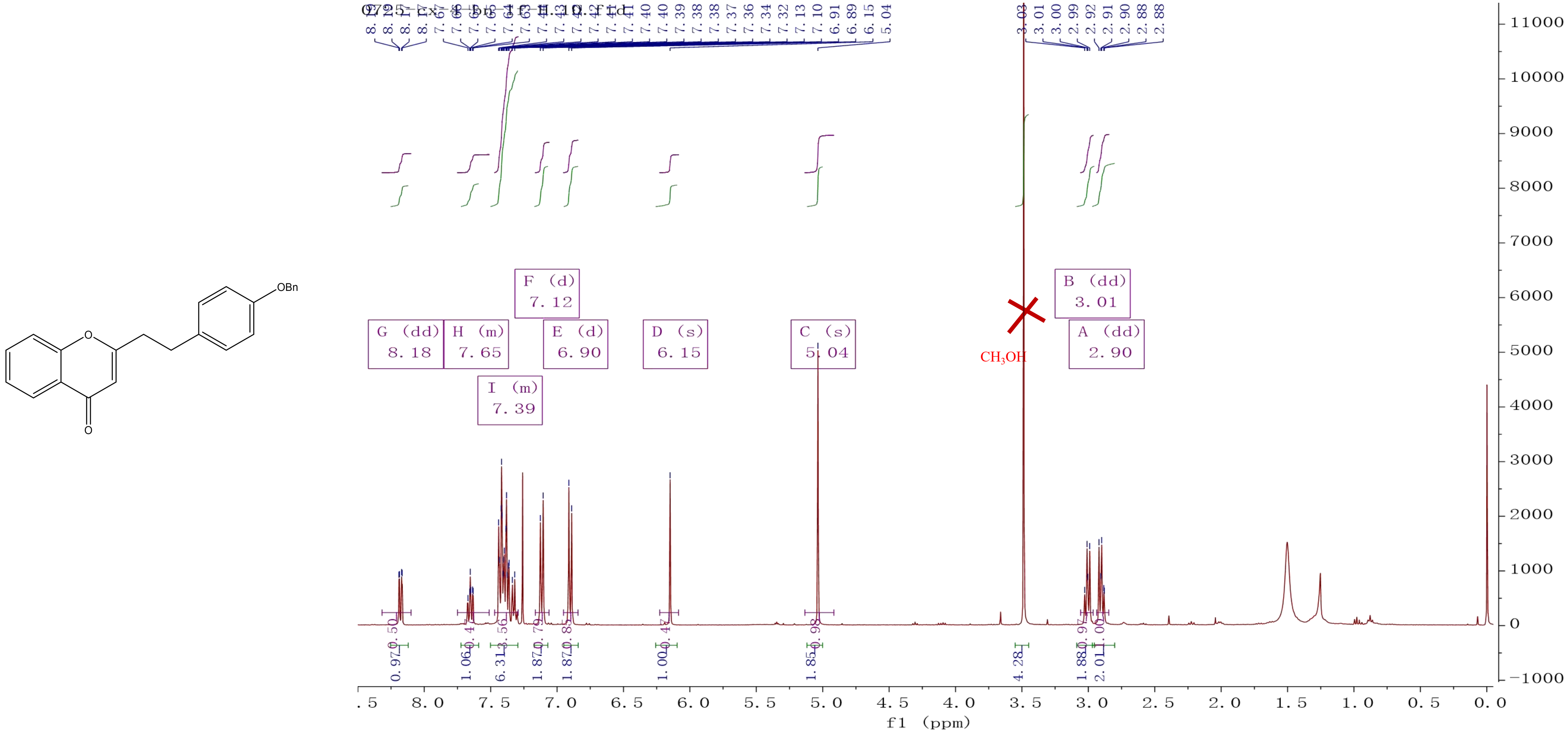

Fig. S5 ^{1}H NMR of 2-(4-(benzyloxy)phenethyl)-4H-chromen-4-one (400MHz, $CDCl_3$)

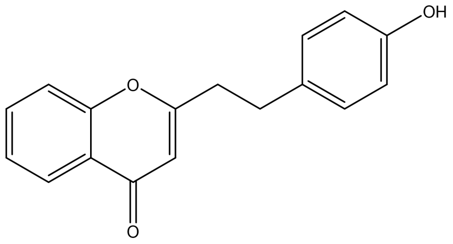

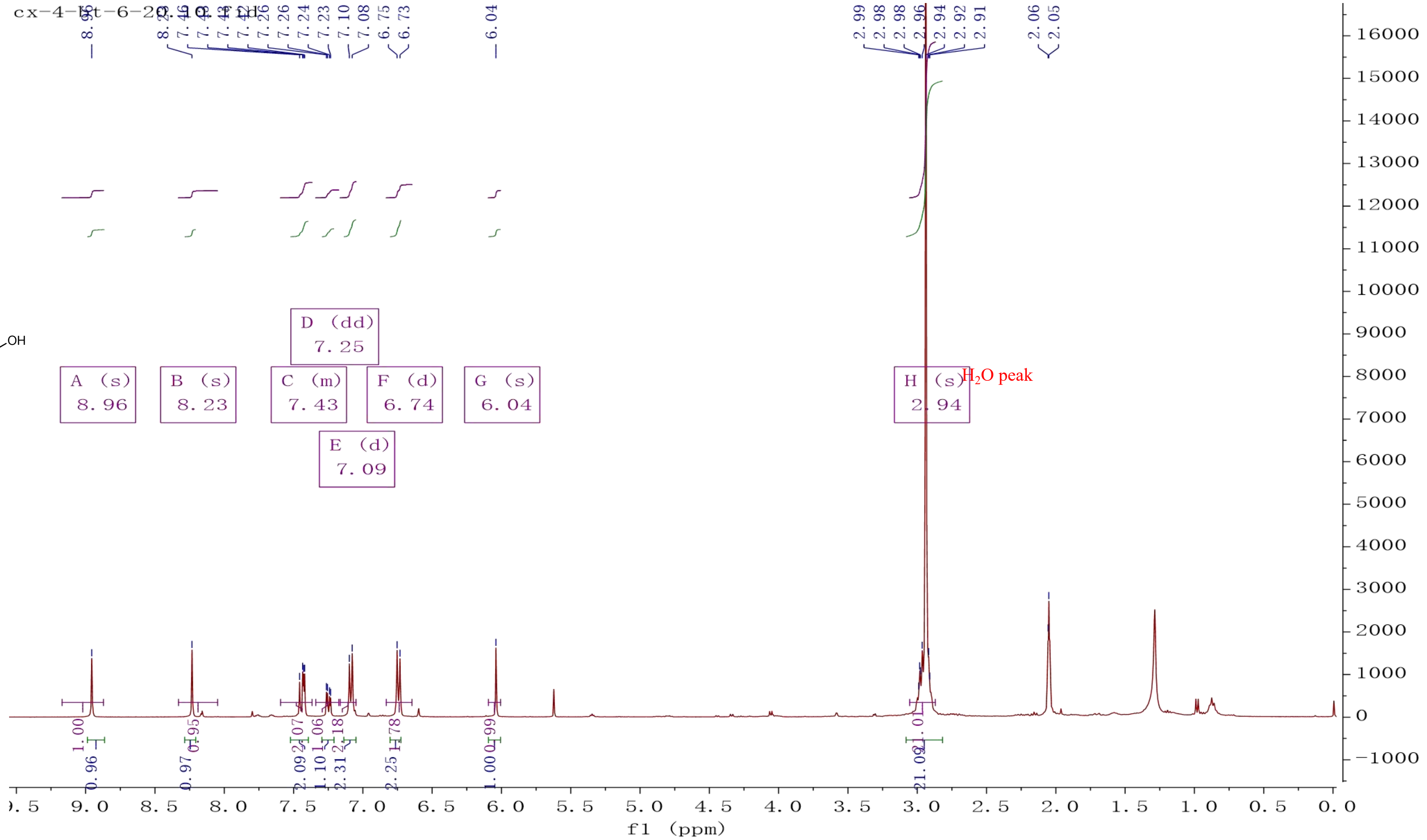

Fig.S6 ^{1}HNMR of 2-(4-hydroxyphenethyl)-4H-chromen-4-one (400MHz, CD_3COCD_3)

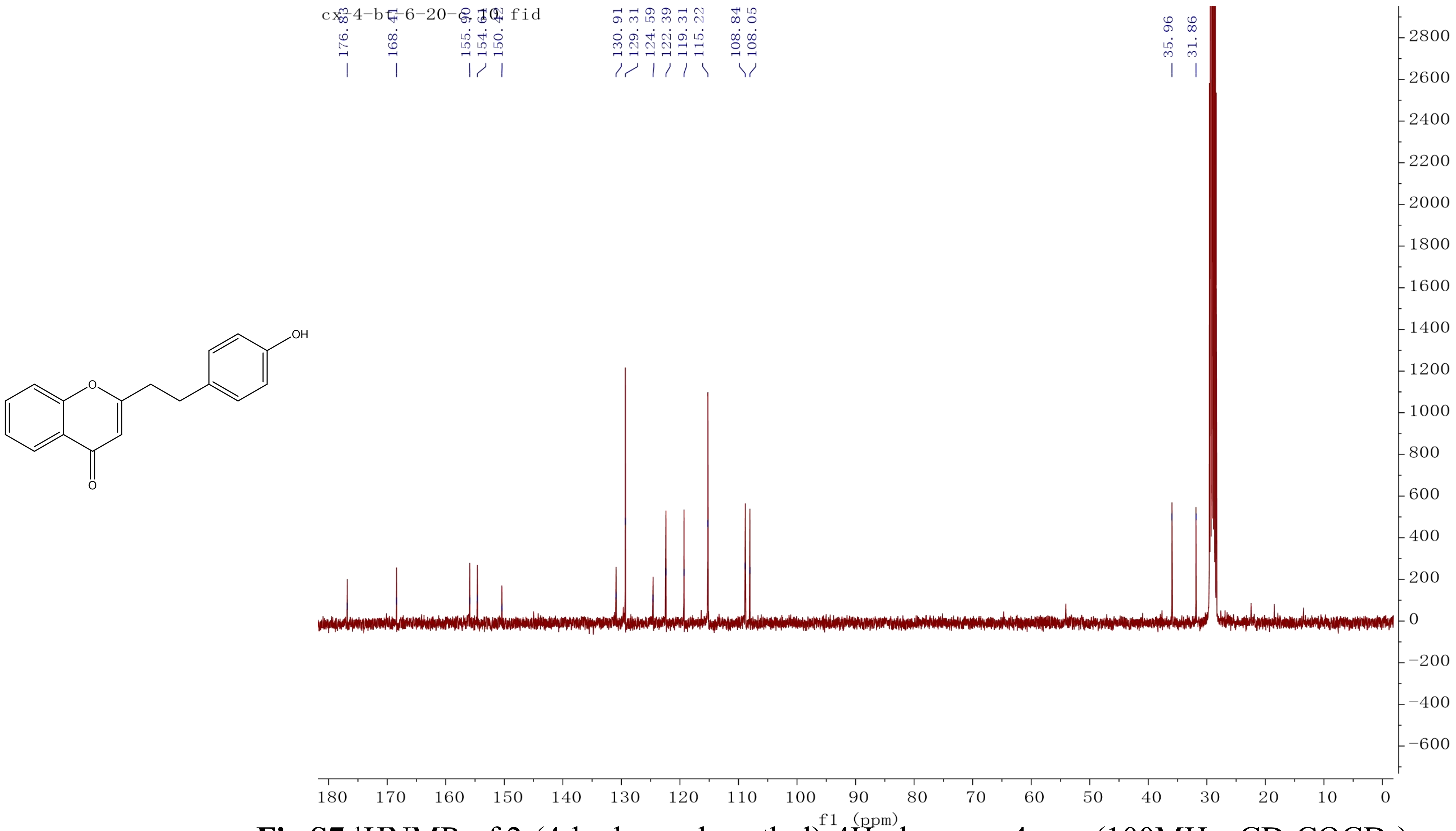

Fig.S7 ^{1}HNMR of 2-(4-hydroxyphenethyl)-4H-chromen-4-one (100MHz, CD_3COCD_3)

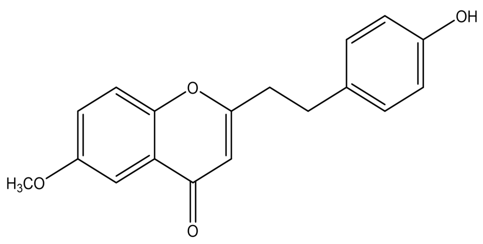

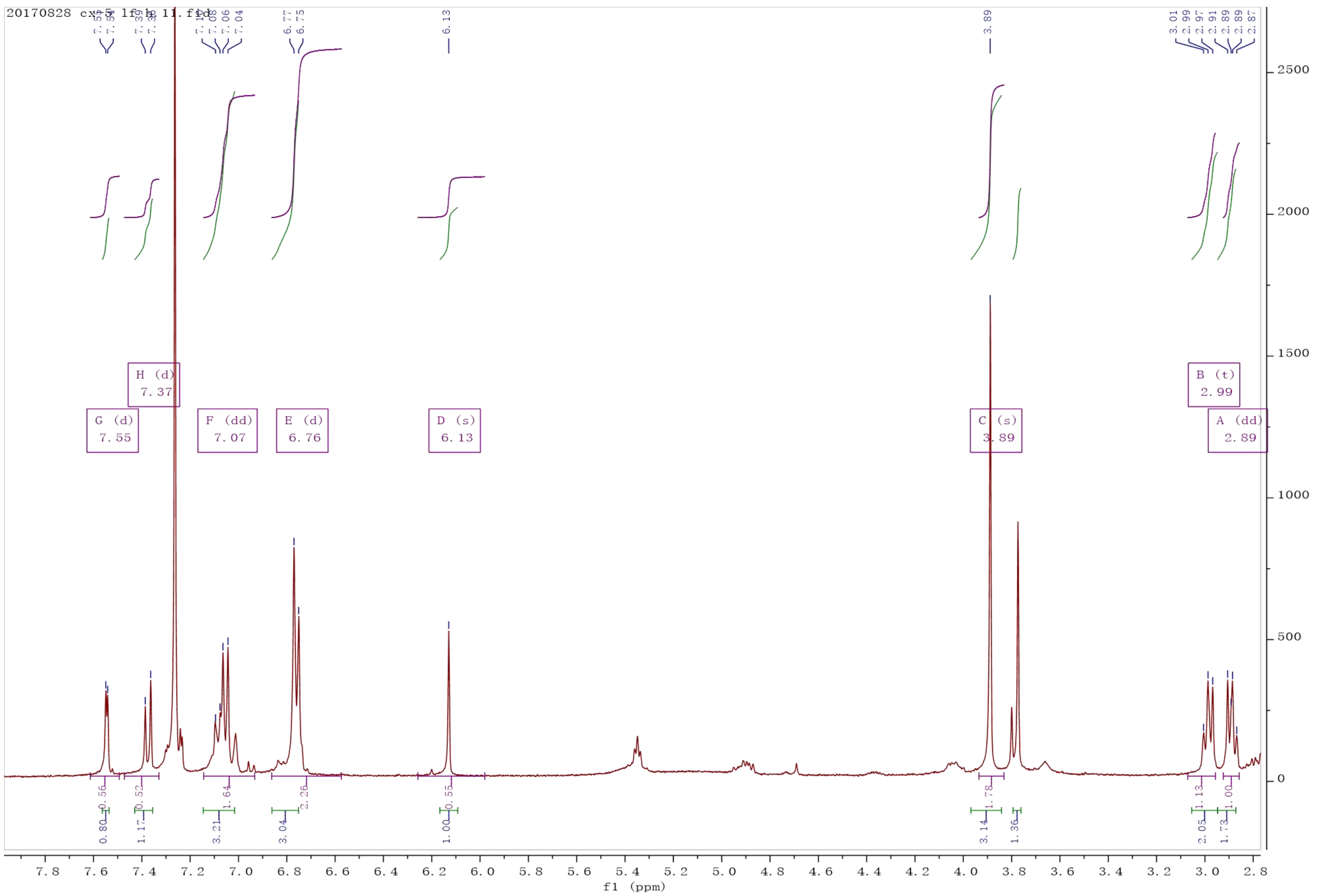

Fig.S8 ^{1}H NMR of 2-(4-hydroxyphenethyl)-6-methoxy-4H-chromen-4-one (400MHz, $CDCl_3$)

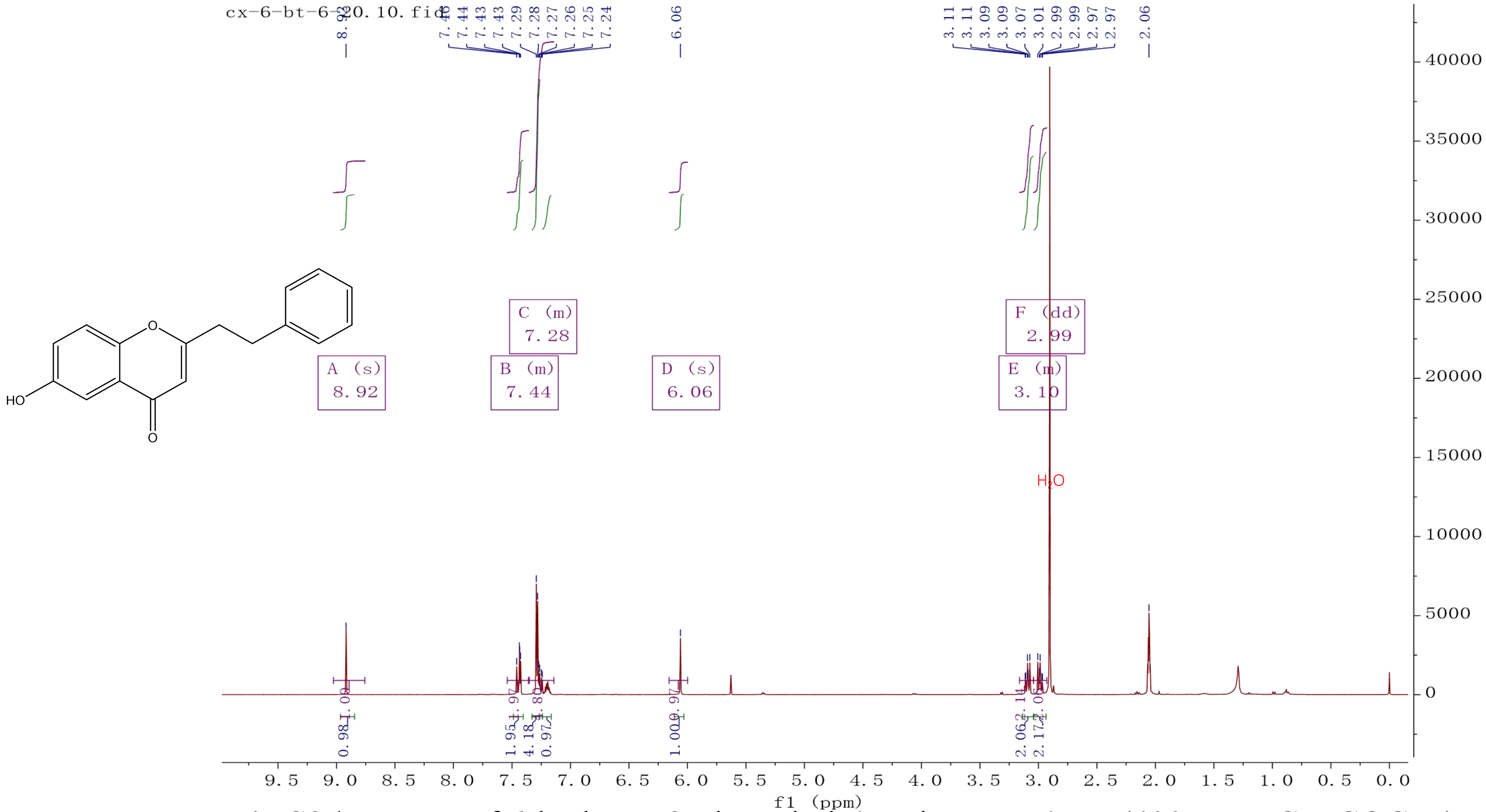

Fig.S9 ^{1}H NMR of 6-hydroxy-2-phenethyl-4H-chromen-4-one(400MHz, CD_3COCD_3)

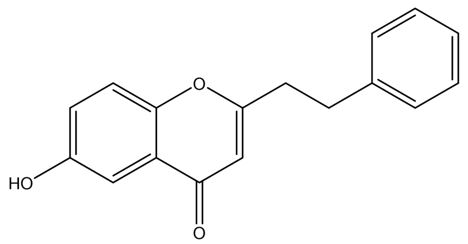

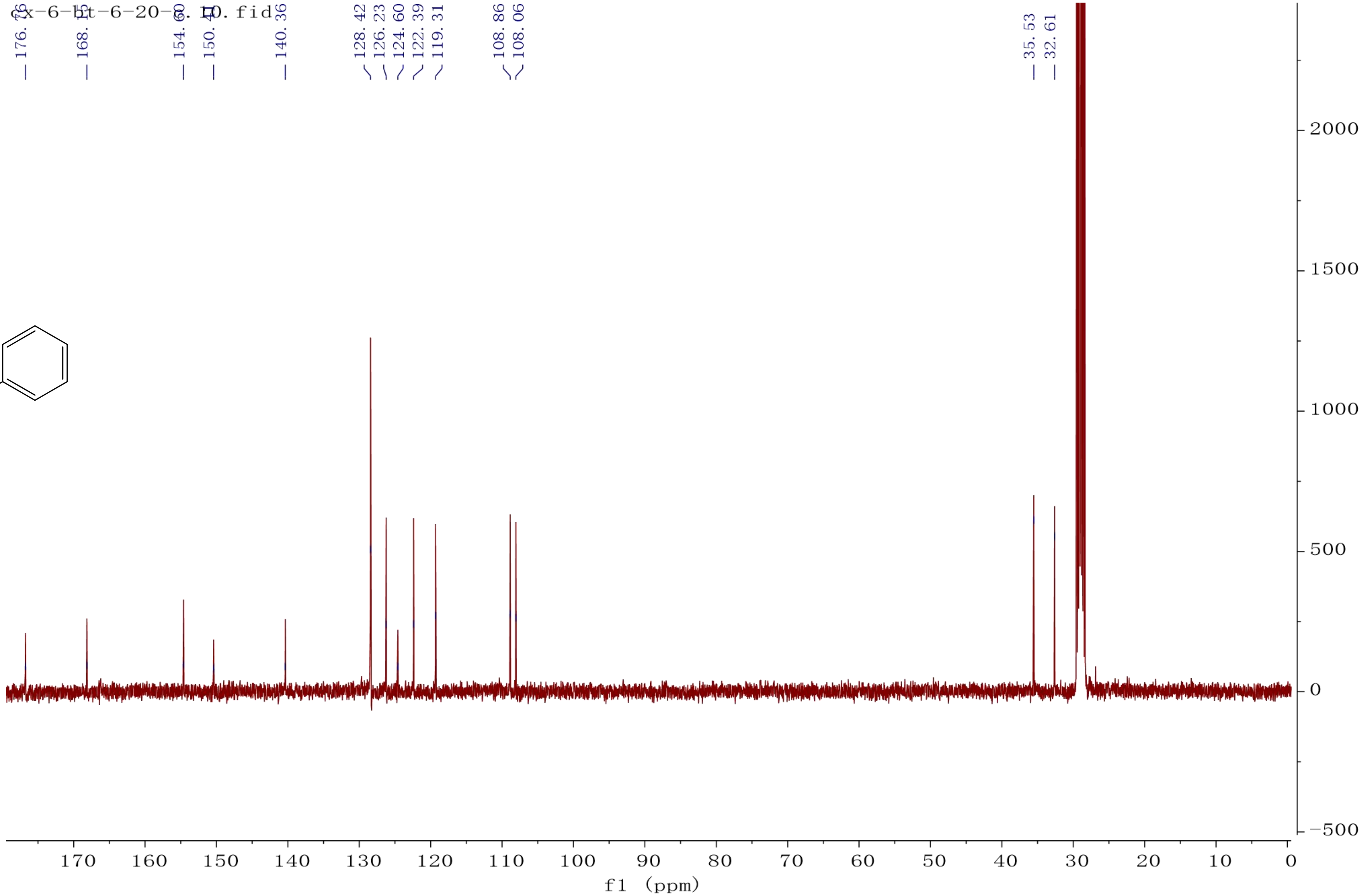

Fig.S10 ^{13}C NMR of 6-hydroxy-2-phenethyl-4H-chromen-4-one (100MHz, CD_3COCD_3)

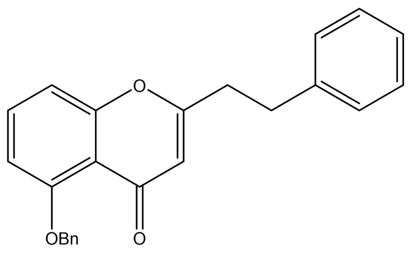

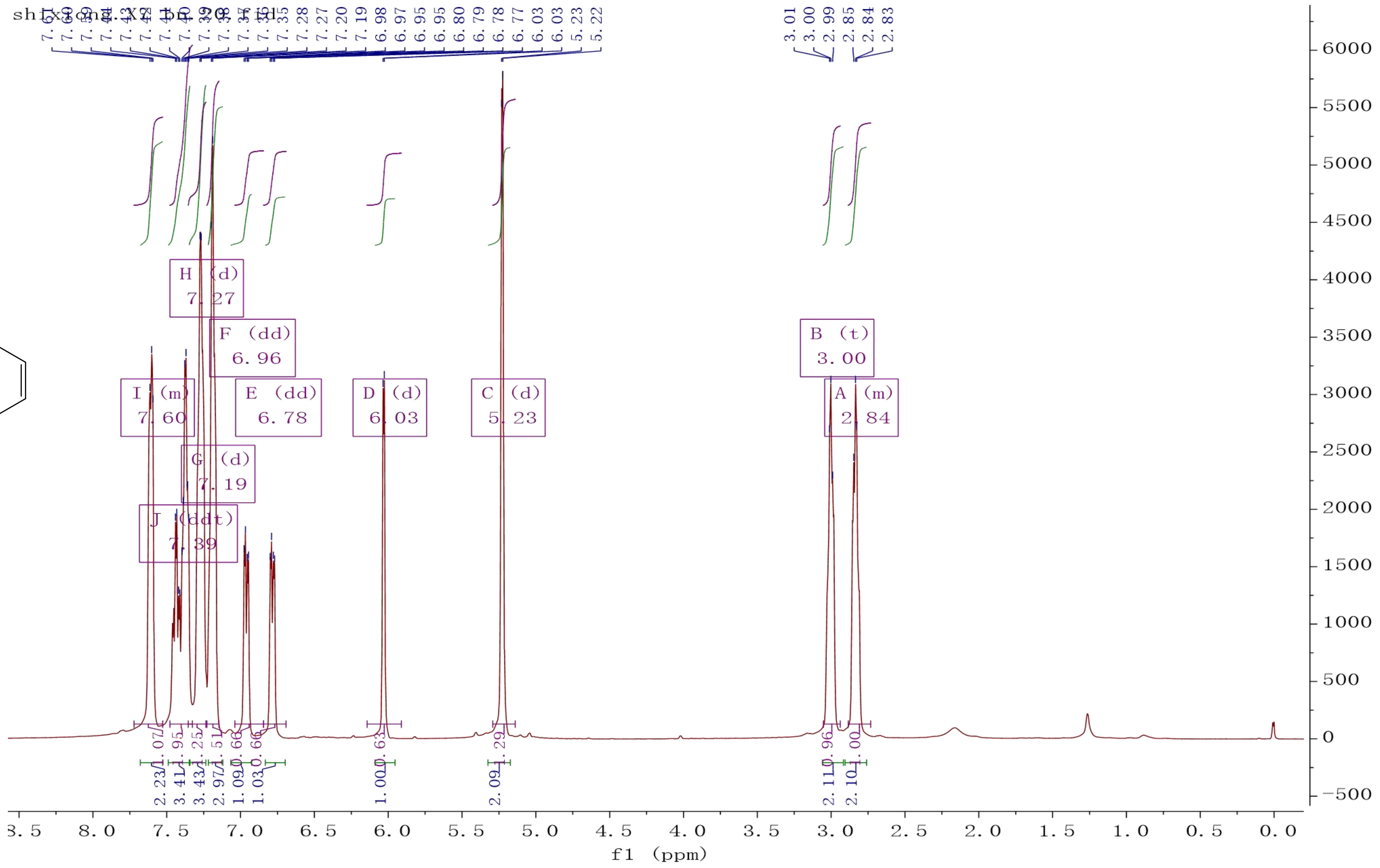

Fig. S11 ¹HNMR 5-(benzyloxy)-2-phenethyl-4H-chromen-4-one (400MHz, $CDCl_3$)

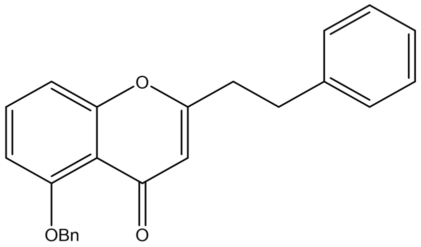

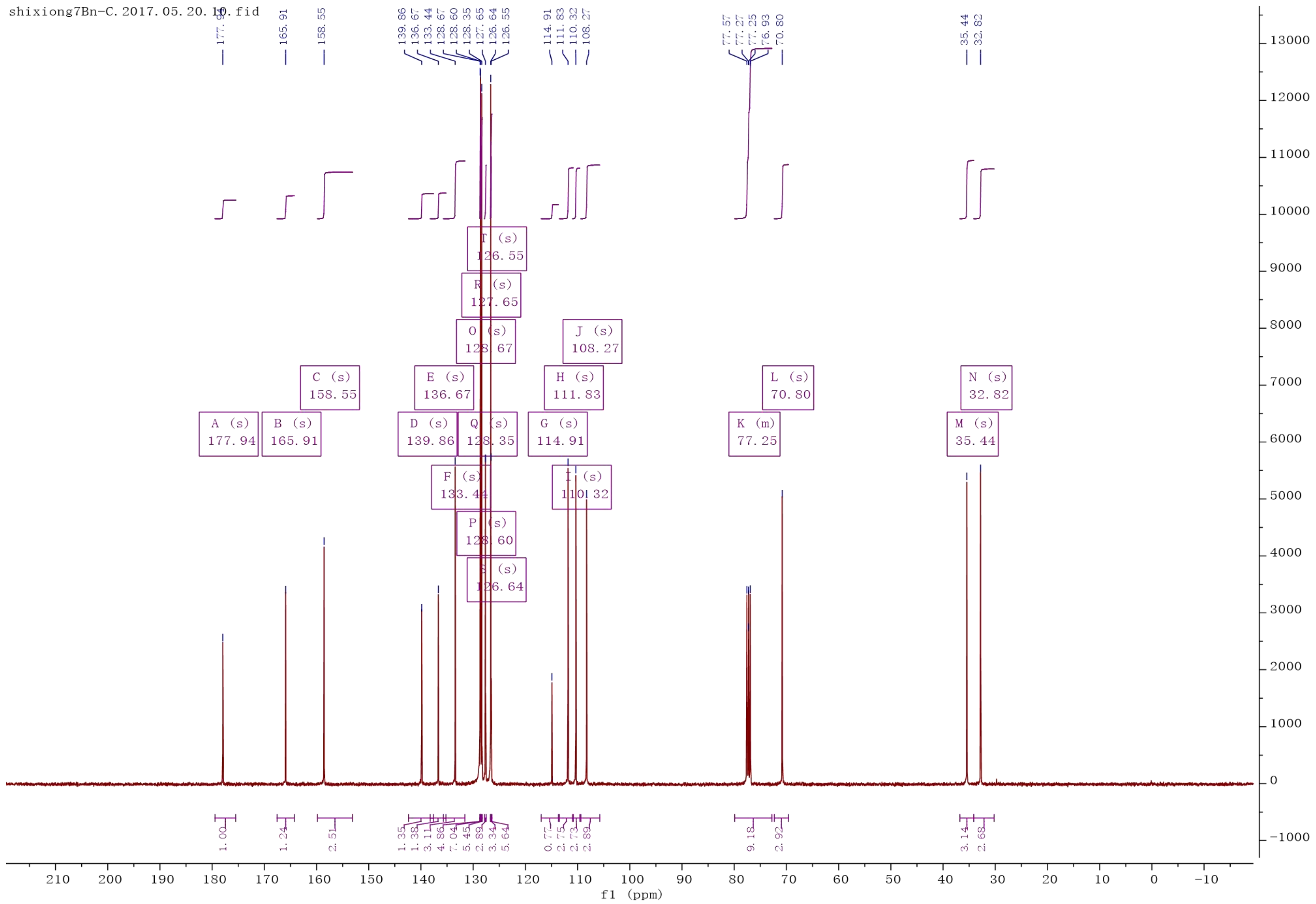

Fig. S12 ^{13}CNMR 5-(benzyloxy)-2-phenethyl-4H-chromen-4-one (100MHz, $CDCl_3$)

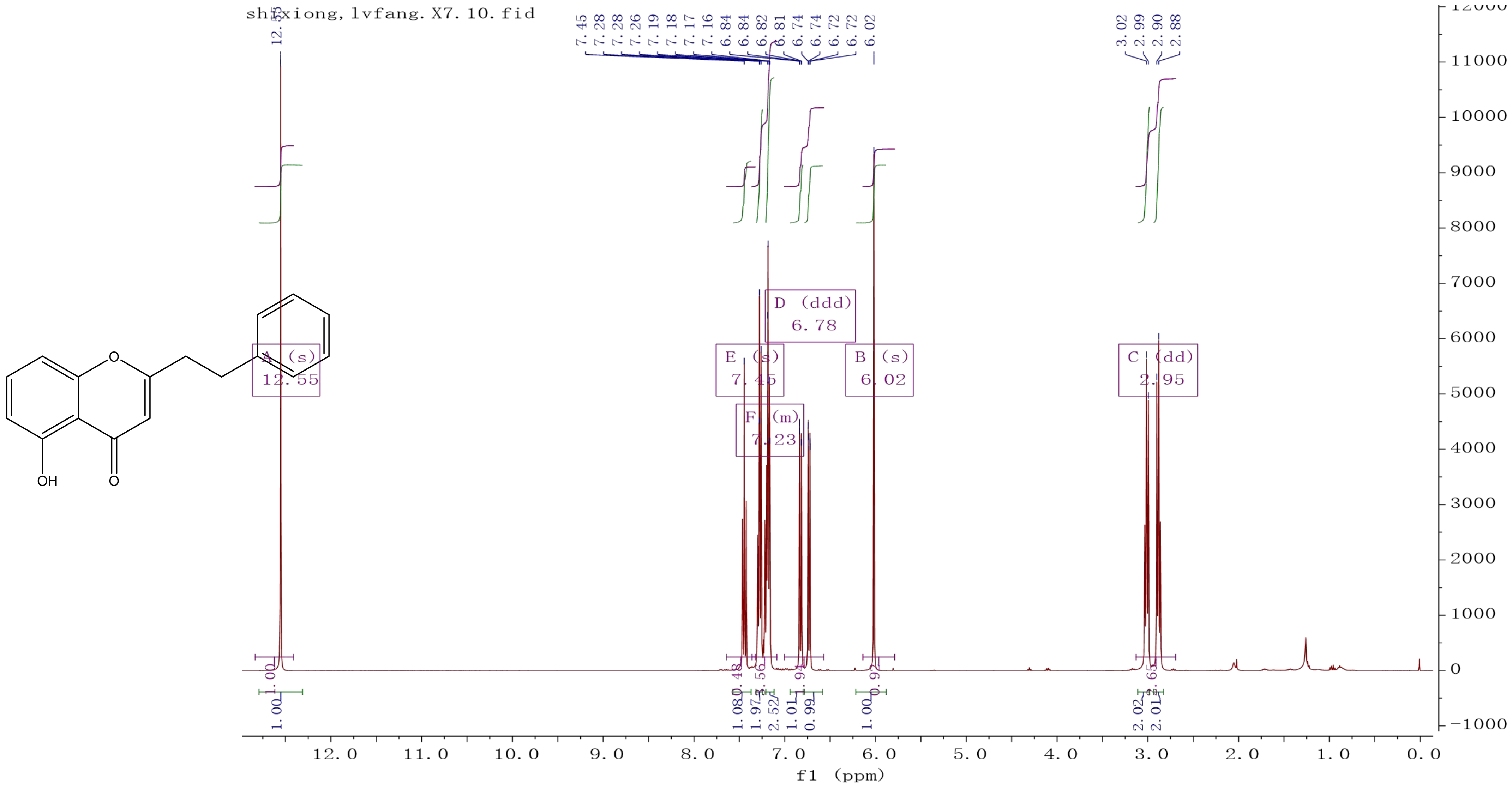

Fig. S13 ^{1}HNMR of 5-hydroxy-2-phenethyl-4H-chromen-4-one (400MHz, $CDCl_3$)

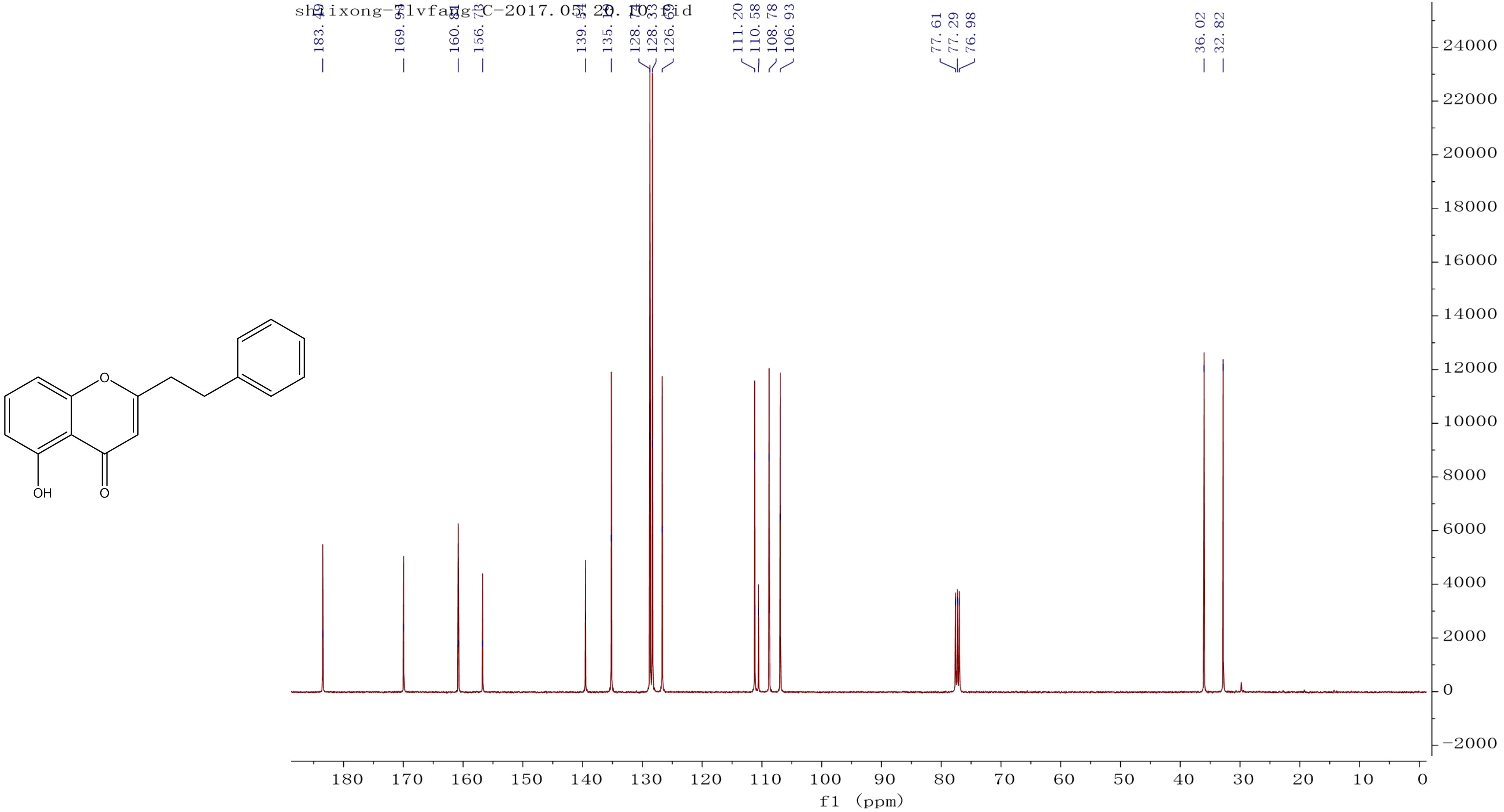

Fig. S14 ^{13}CNMR of 5-hydroxy-2-phenethyl-4H-chromen-4-one (100MHz, $CDCl_3$)

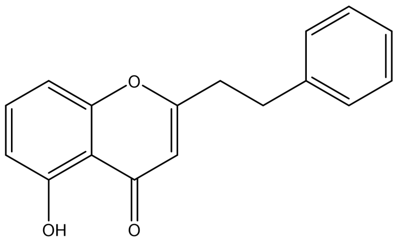

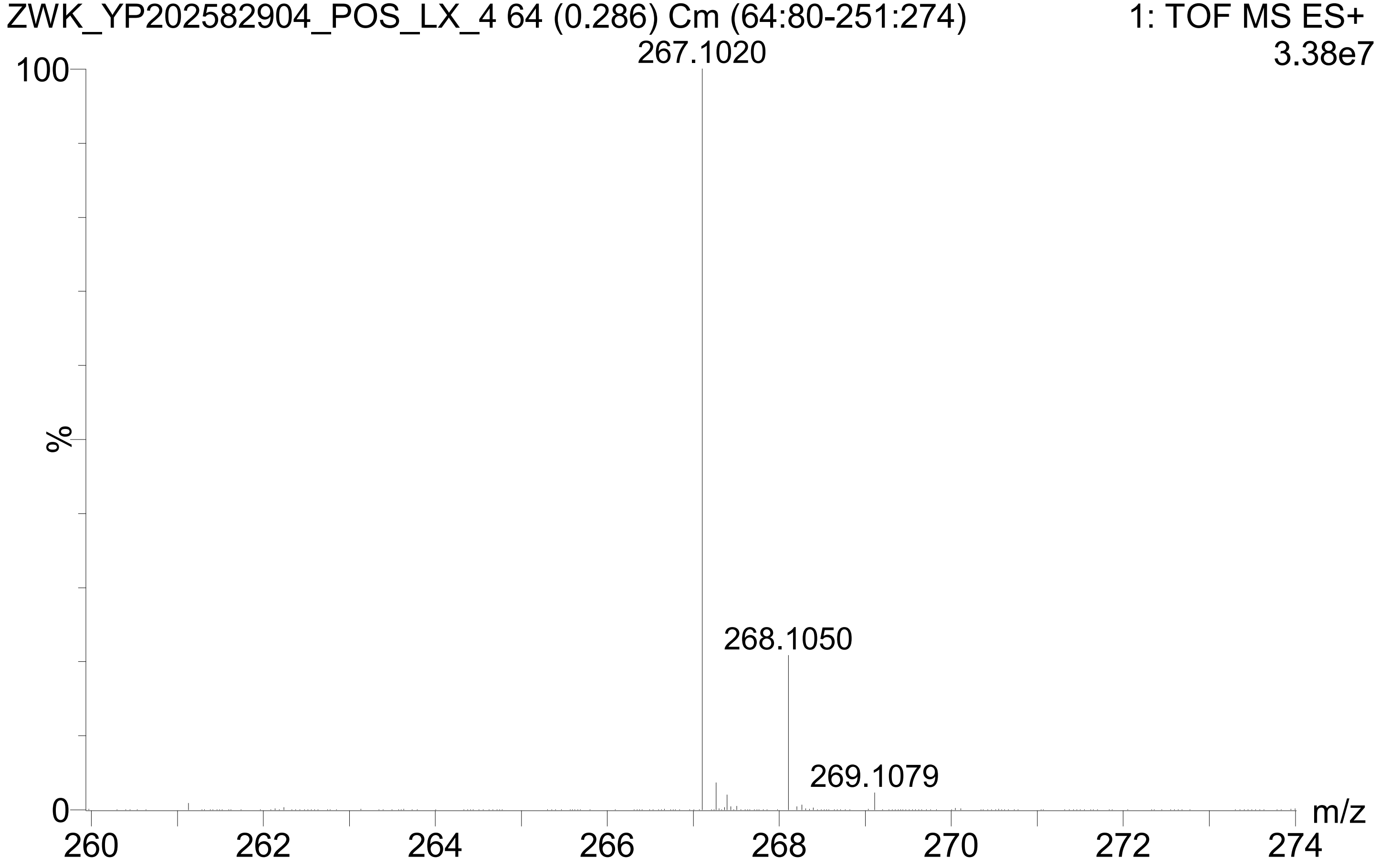

Fig. S15 ESI (+) -HRMS of 5-hydroxy-2-phenethyl-4H-chromen-4-one

HPLC conditions：Gradient elution

Time (min)	Flow rate（mL/min）	A- ammonium acetate(150 mM) (%)	B-methanol (%)
0	0.2	40	60
2	0.2	40	60
5	0.2	10	90
15	0.2	10	90
17	0.2	40	60
20	0.2	40	60

Wavelength：254nm; Injection volume: 10 μL

Fig. S16 Detailed HPLC conditions for analyzing all the synthesized compounds.

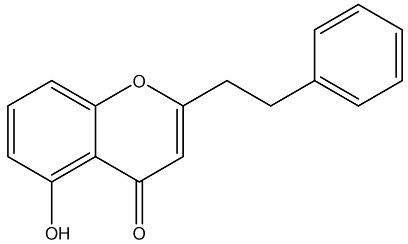

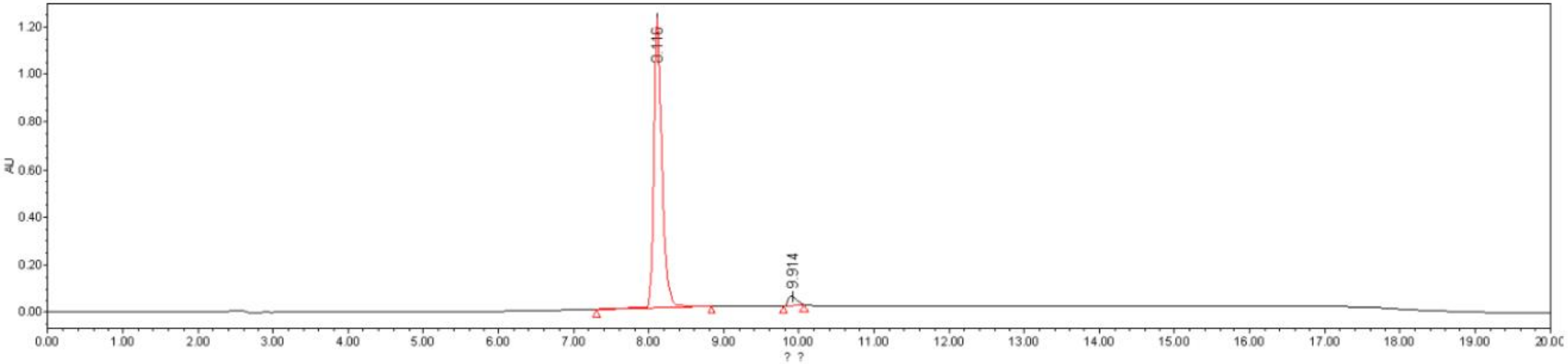

		Retention time	Area	Area ratio (%)
1		8.116	9093978	96.73
2		9.914	307856	3.27

Fig. S17 HPLC of 5-hydroxy-2-phenethyl-4H-chromen-4-one (RT=8.116 min, purity= 96.73%)

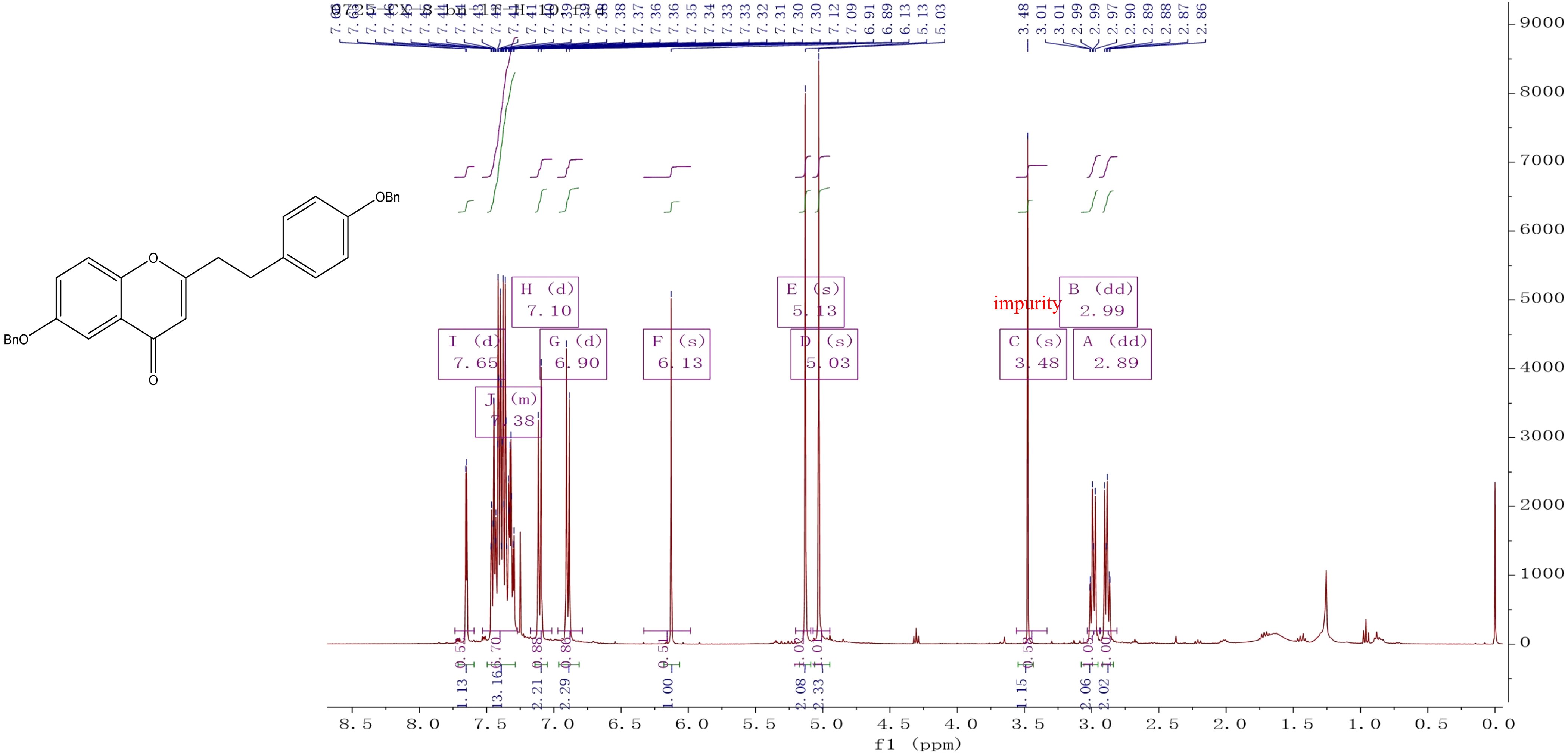

Fig.S18. ^{1}H NMR of 6-(benzyloxy)-2-(4-(benzyloxy)phenethyl)-4H-chromen-4-one (400MHz, $CDCl_3$)

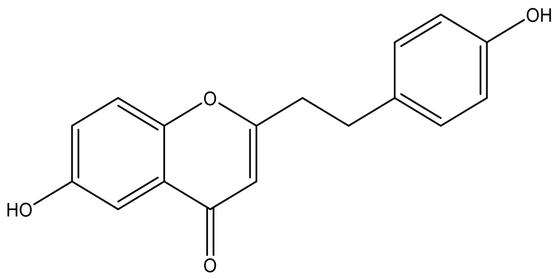

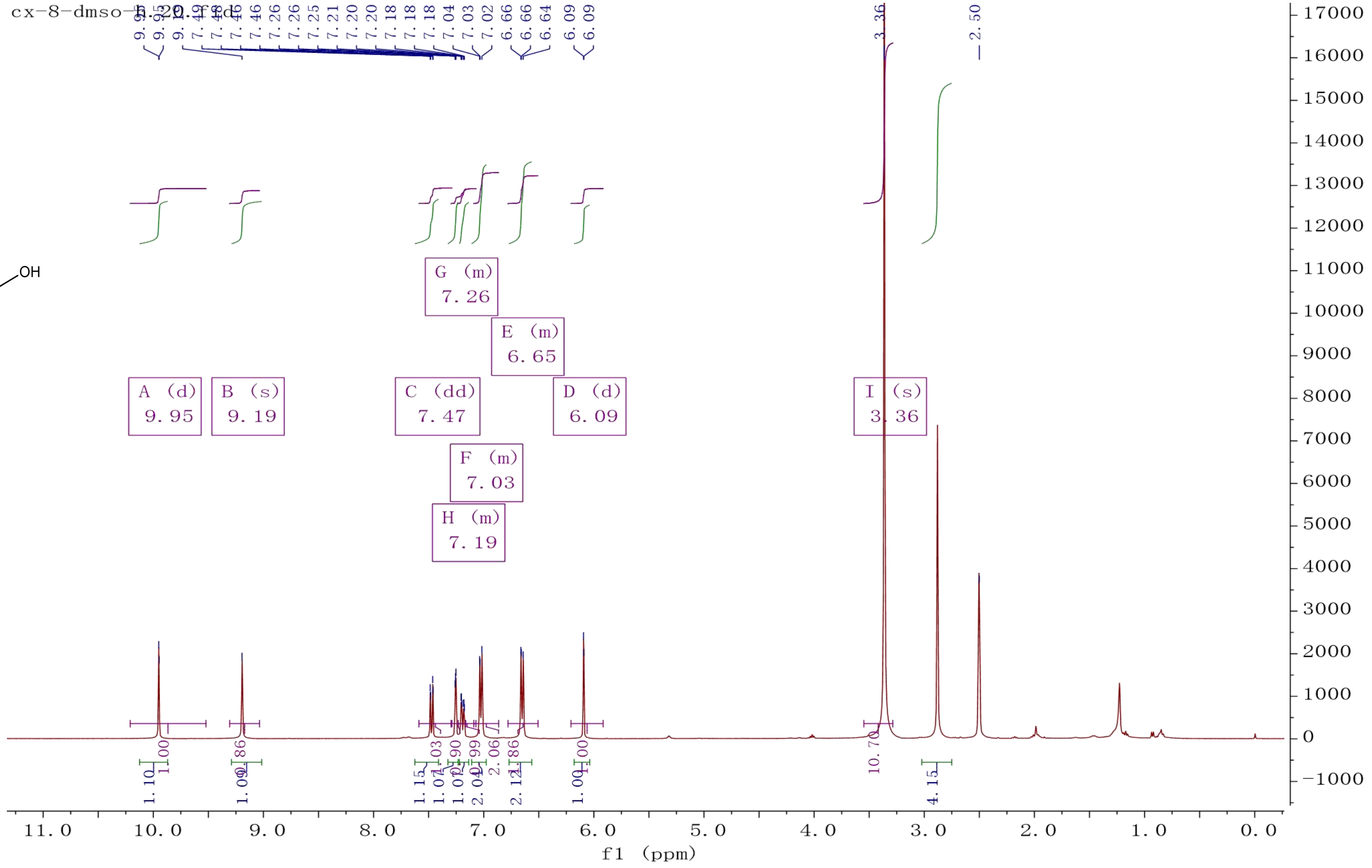

Fig.S19 ^{1}HNMR of 6-hydroxy-2-(4-hydroxyphenethyl)-4H-chromen-4-one (400MHz, DMSO-d_6)

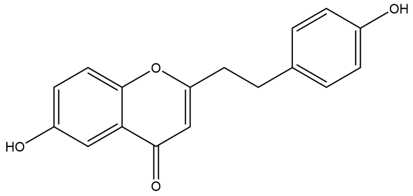

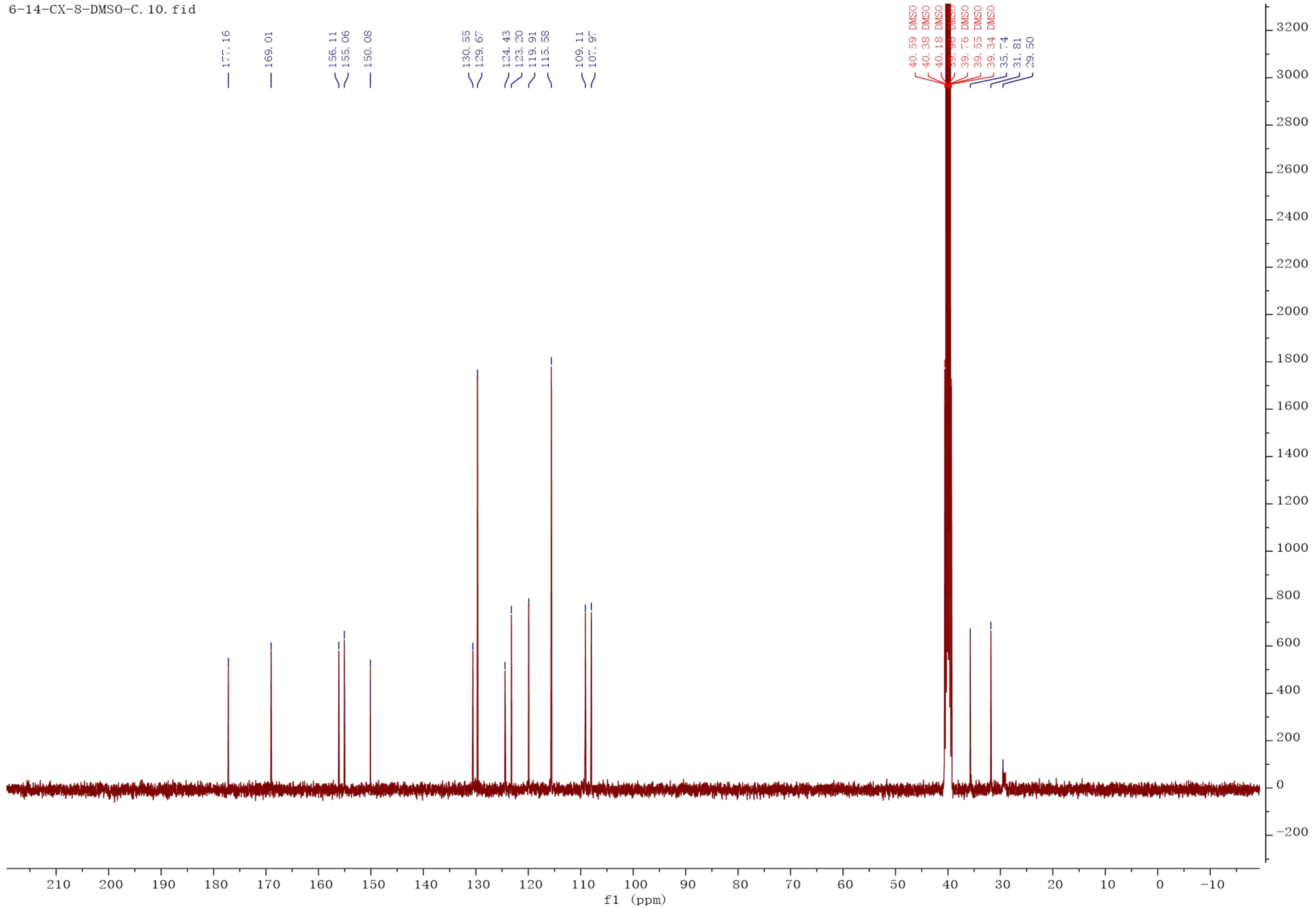

Fig.S20 ^{13}CNMR of 6-hydroxy-2-(4-hydroxyphenethyl)-4H-chromen-4-one (100MHz, DMSO-d_6)

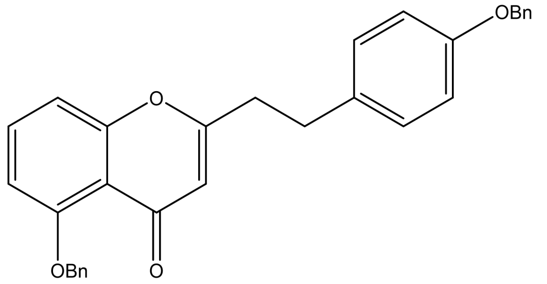

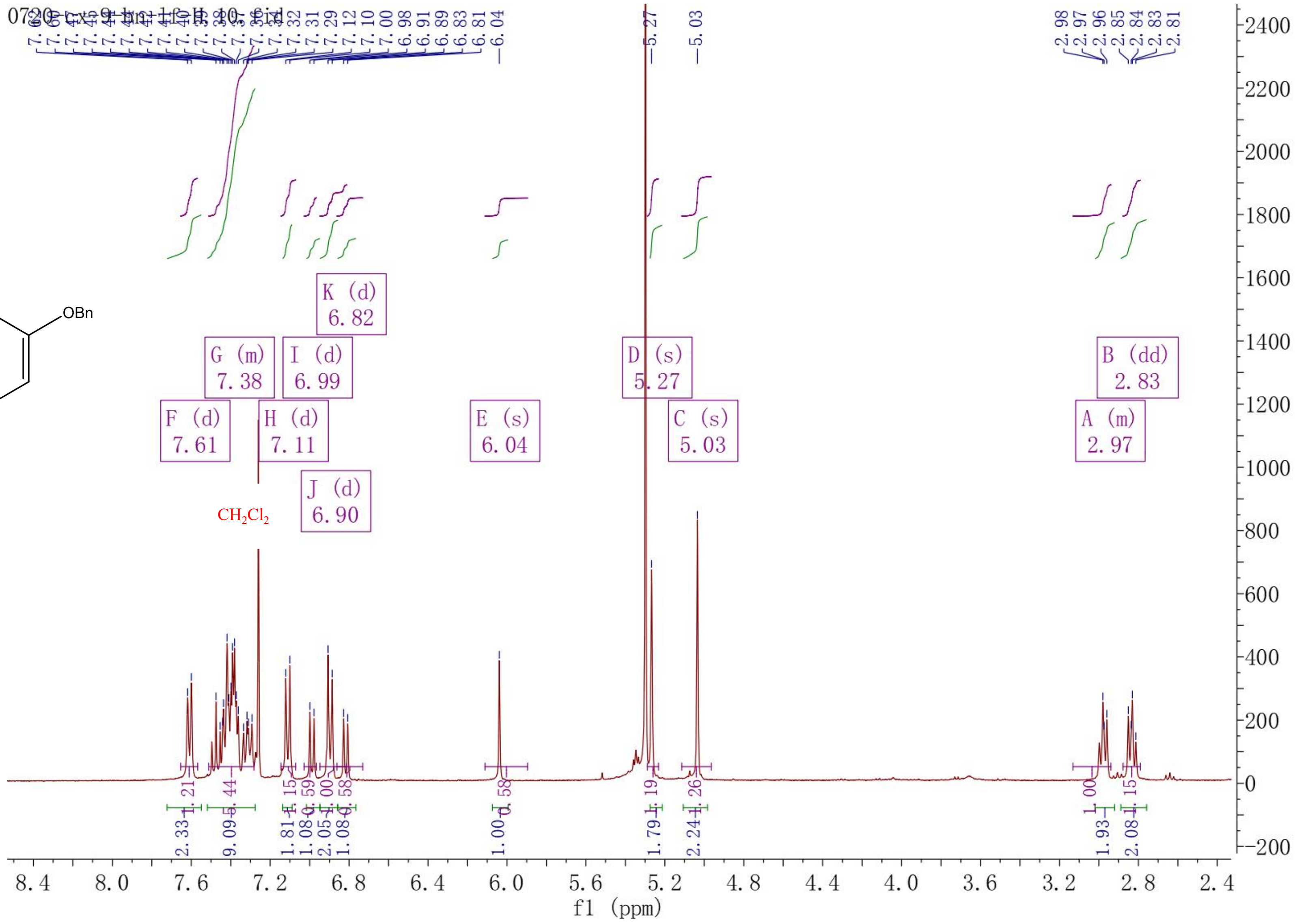

Fig S21 ^{1}HNMR of 5-(benzyloxy)-2-(4-(benzyloxy)phenethyl)-4H-chromen-4-one (400MHz, $CDCl_3$)

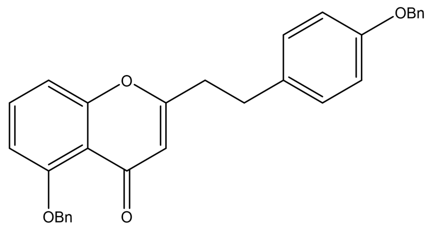

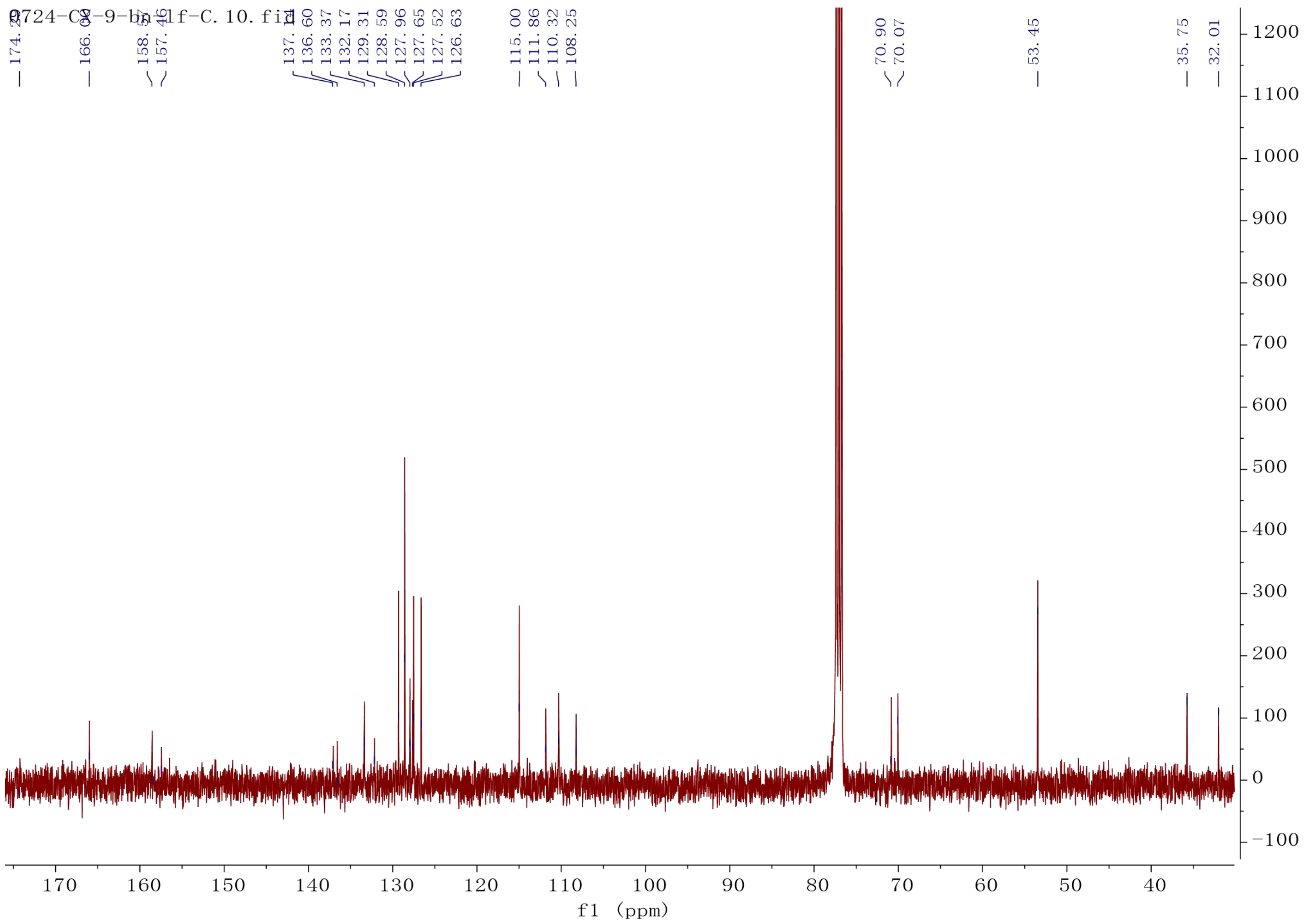

Fig S22 ^{13}C NMR of 5-(benzyloxy)-2-(4-(benzyloxy)phenethyl)-4H-chromen-4-one (100 MHz, $CDCl_3$)

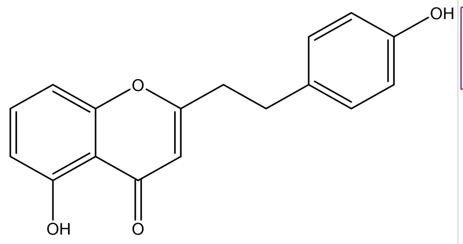

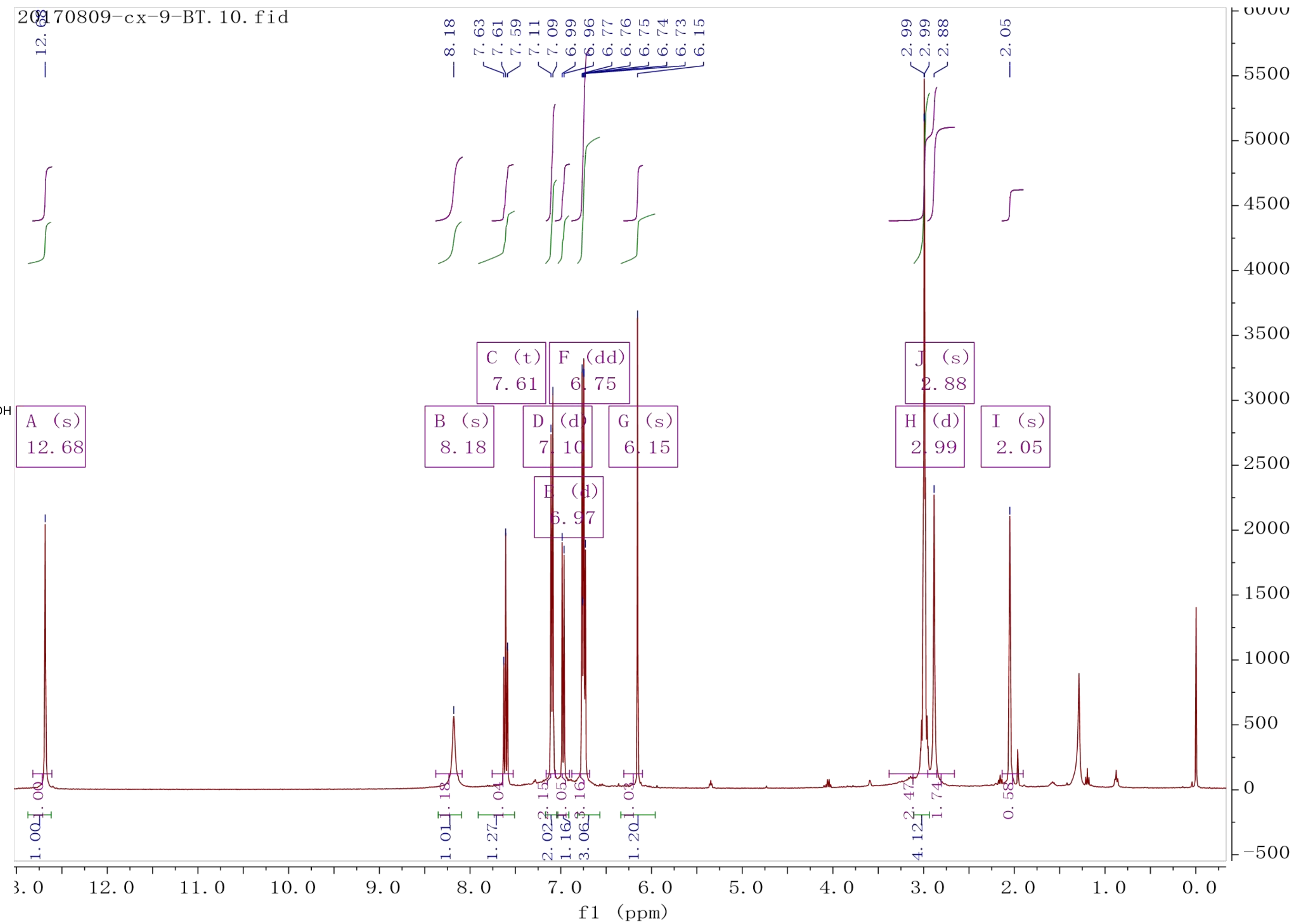

Fig S23 ^{1}HNMR of 5-(hydroxy)-2-(4-hydroxyl-phenethyl)-4H-chromen-4-one (400 MHz, CD_3COCD_3)

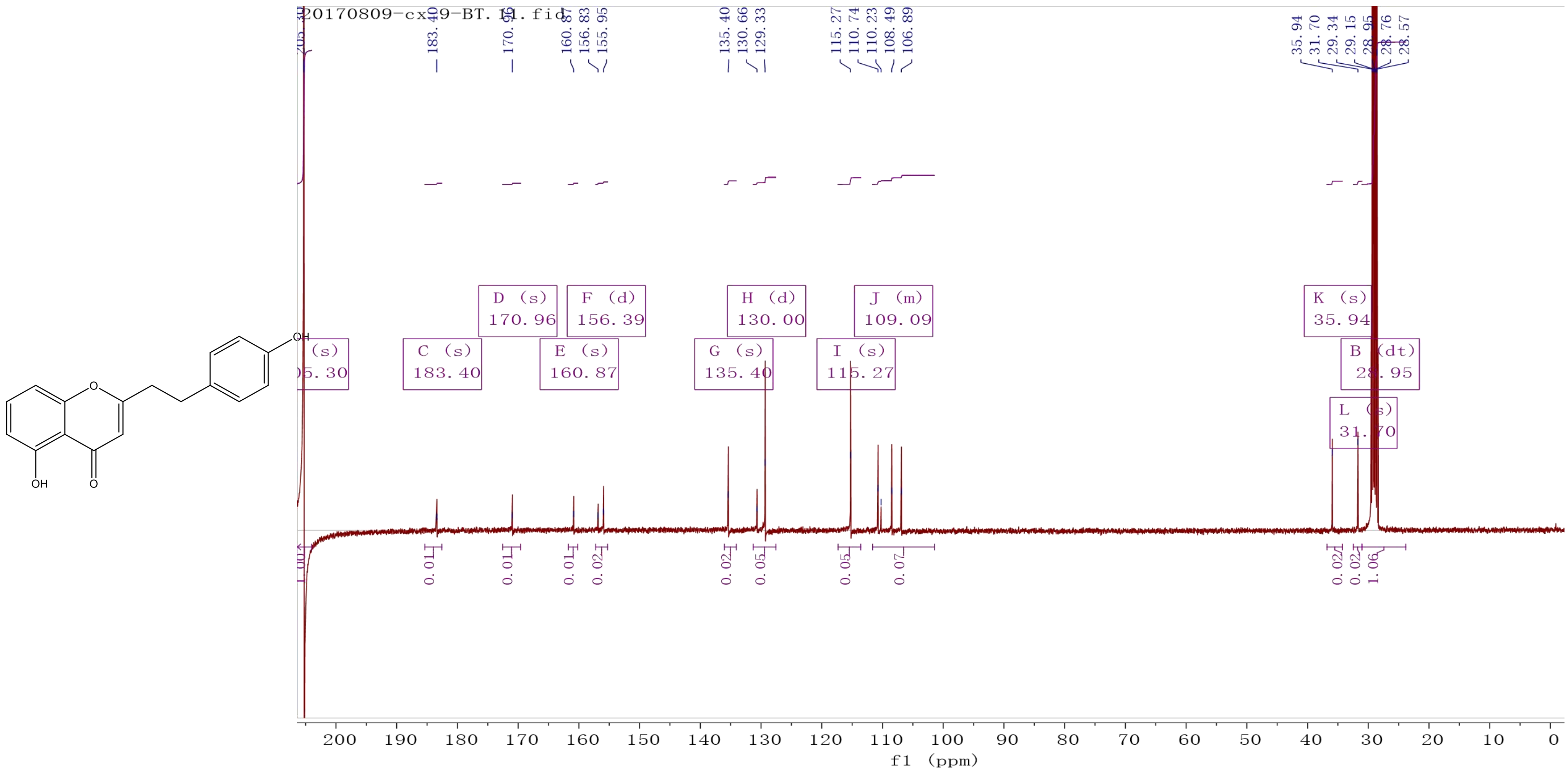

Fig S24 ^{13}CNMR of 5-(hydroxy)-2-(4-hydroxyl-phenethyl)-4H-chromen-4-one(100MHz, CD_3COCD_3)

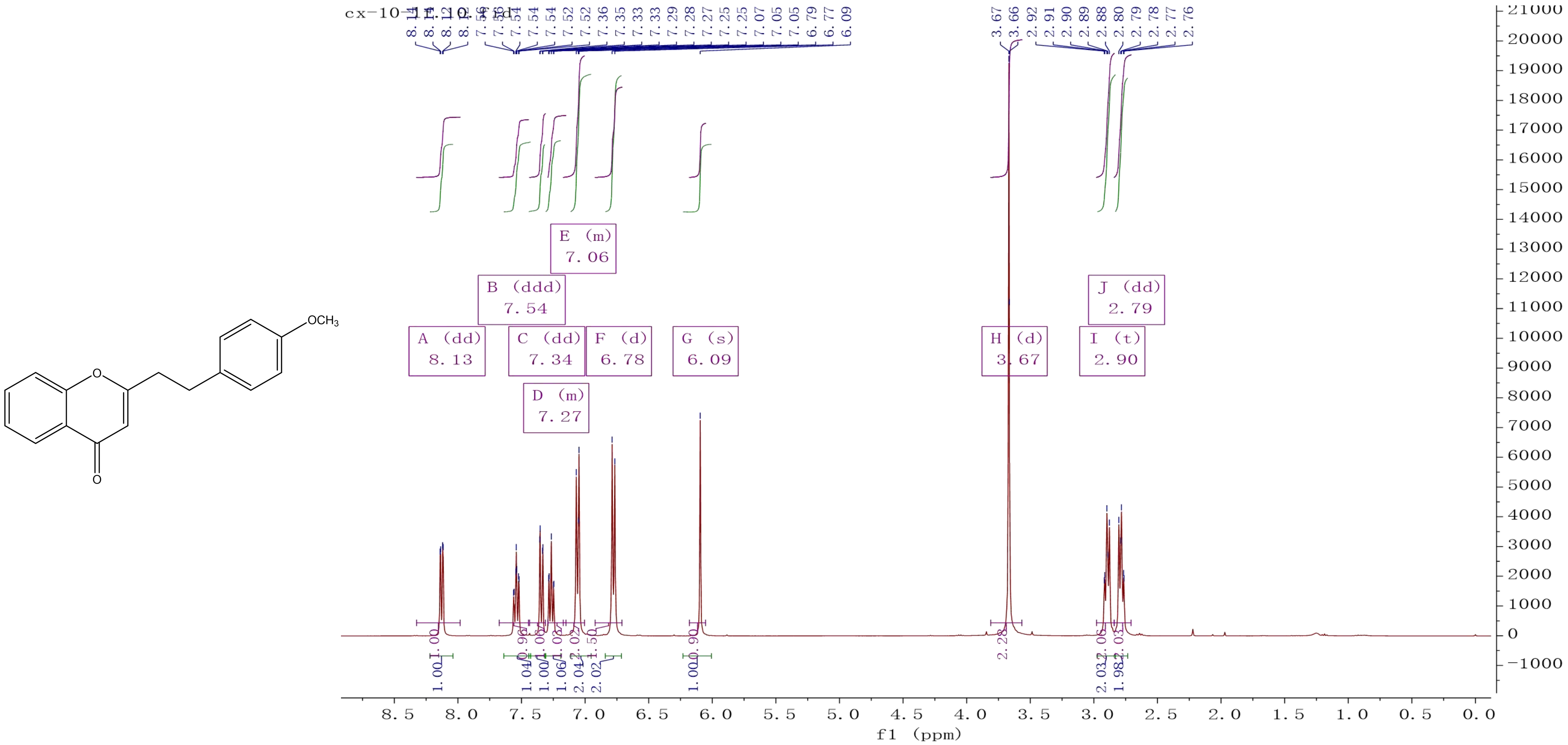

Fig S25 1HNMR of 2-(4-methoxyphenethyl)-4H-chromen-4-one (400MHz, $CDCl_3$)

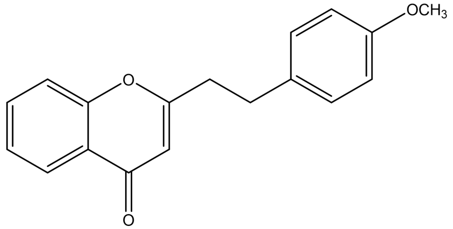

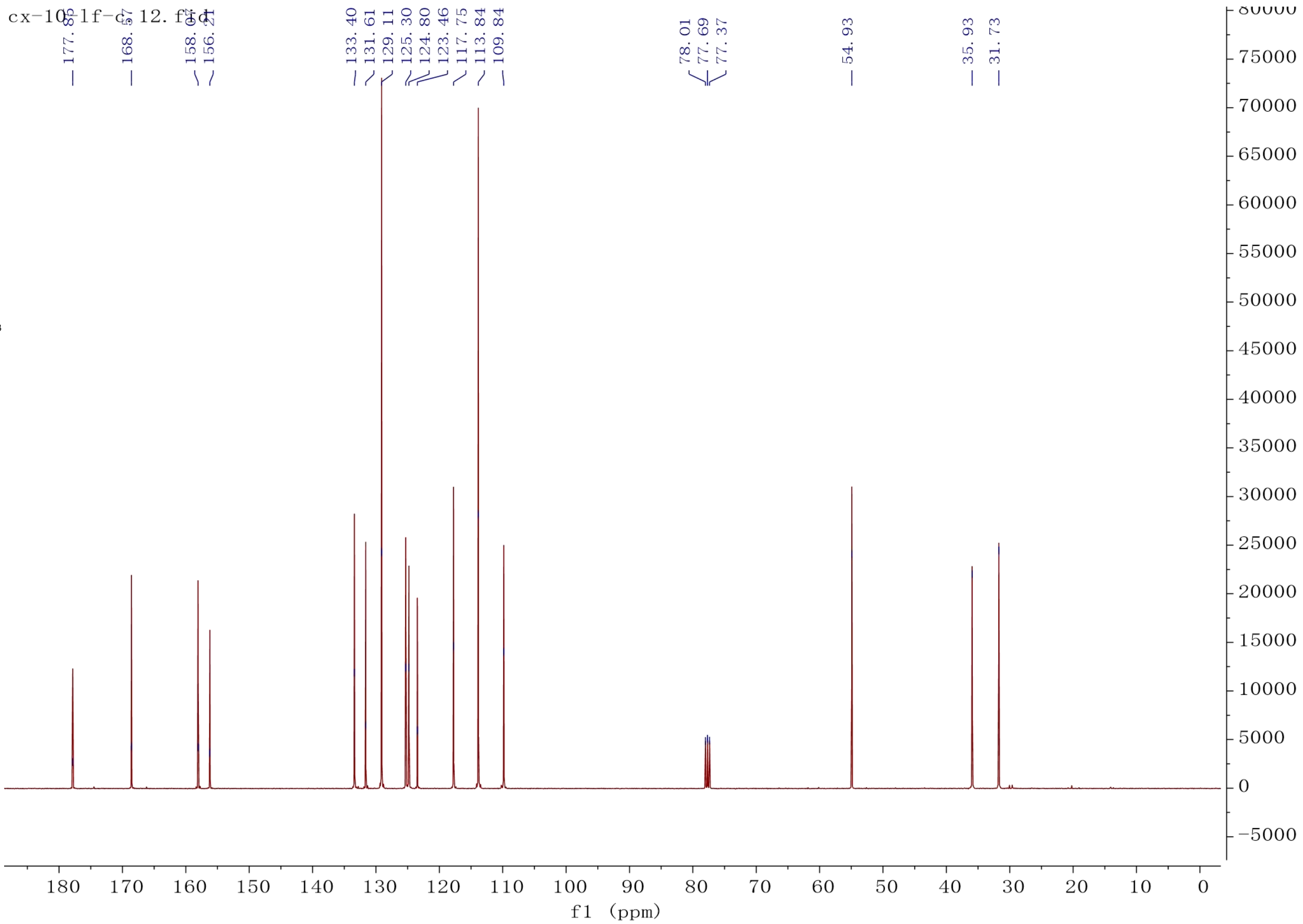

Fig S26 ^{13}CNMR of 2-(4-methoxyphenethyl)-4H-chromen-4-one (100MHz, $CDCl_3$)

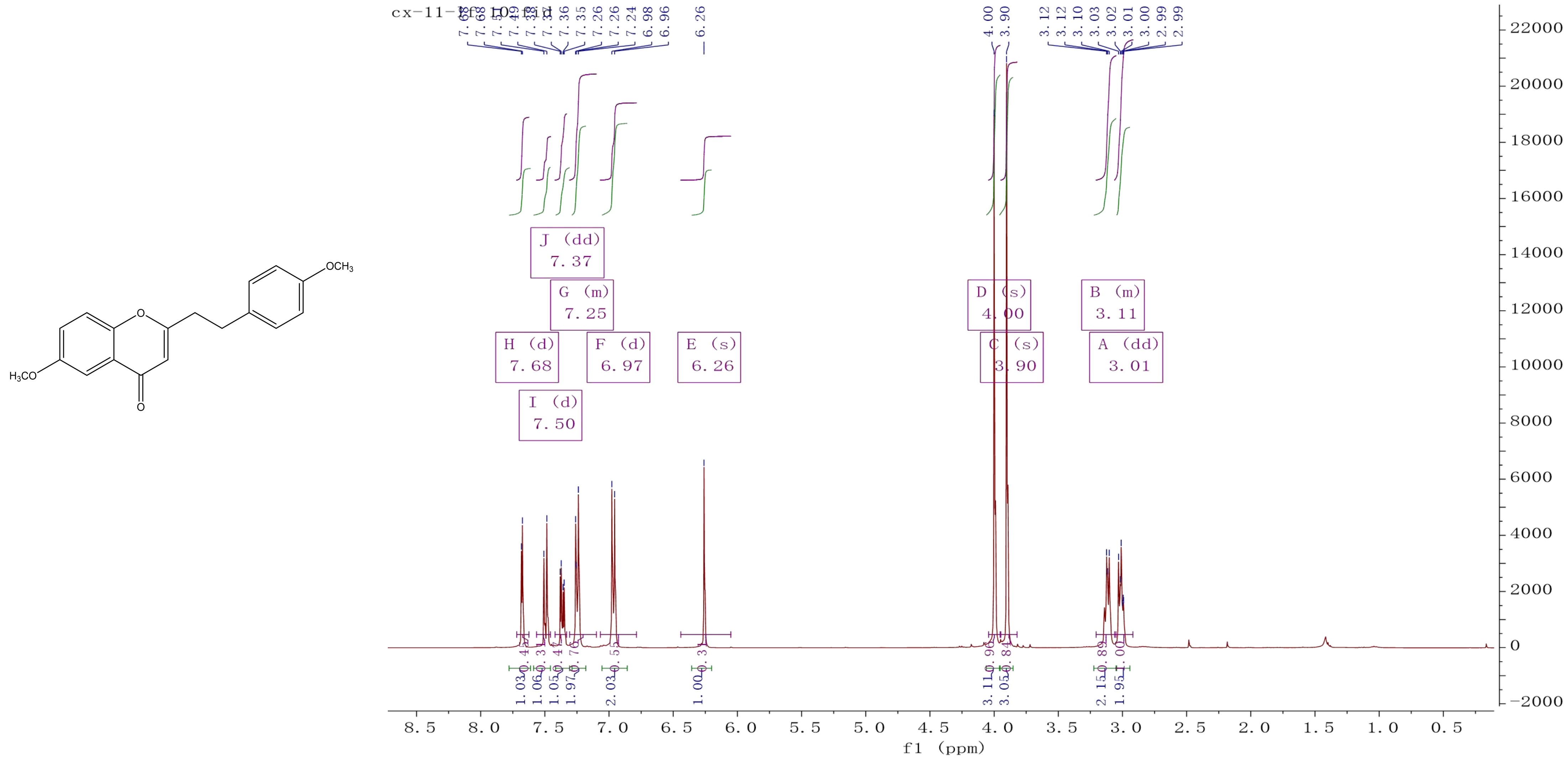

Fig S27. [1]HMNR of 6-methoxy-2-(4-methoxyphenethyl)-4H-chromen-4-one (400MHz, $CDCl_3$)

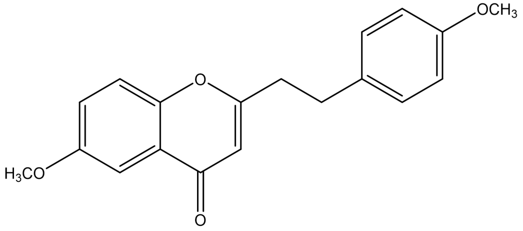

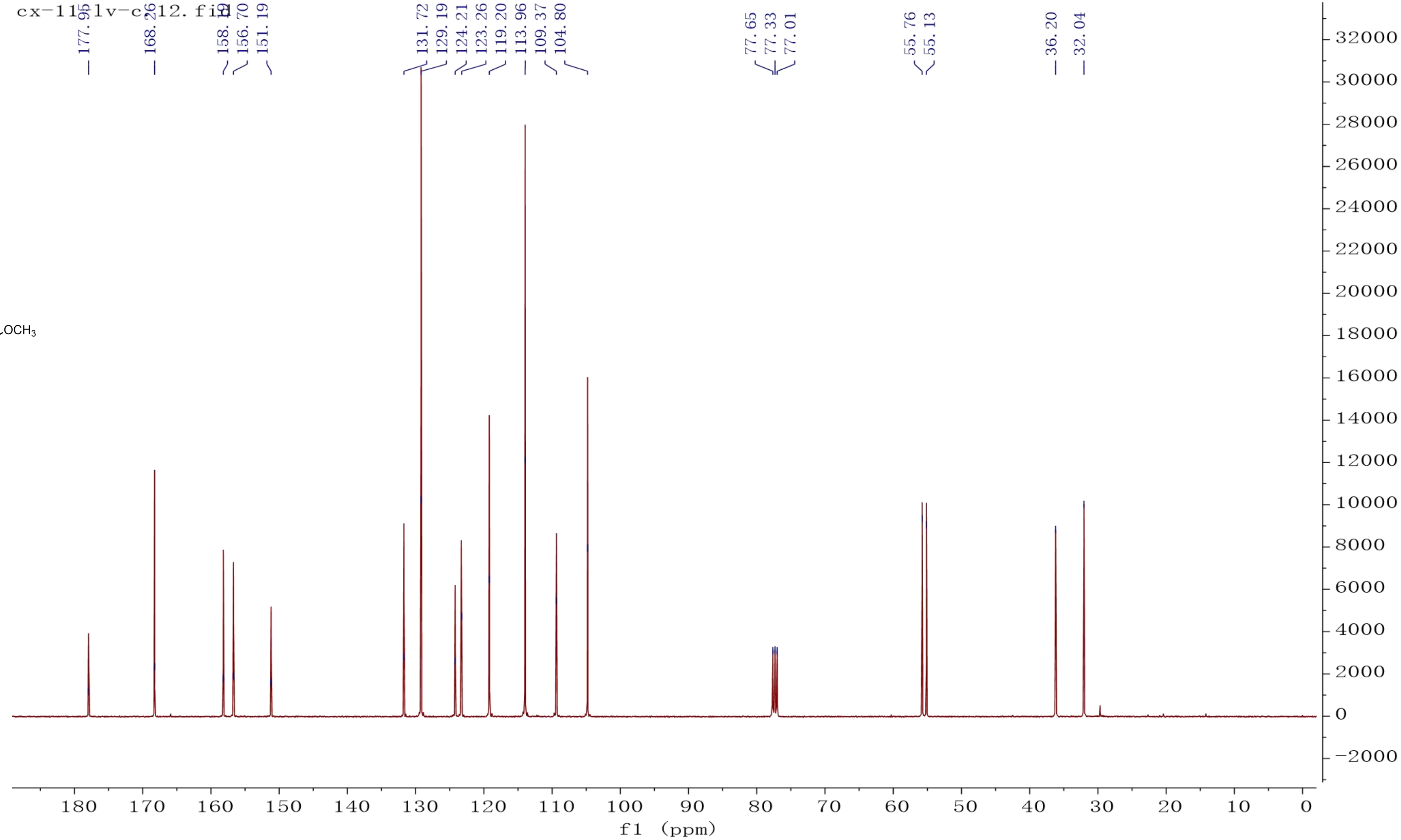

Fig S28. ^{13}CMNR of 6-methoxy-2-(4-methoxyphenethyl)-4H-chromen-4-one (100 MHz, $CDCl_3$)

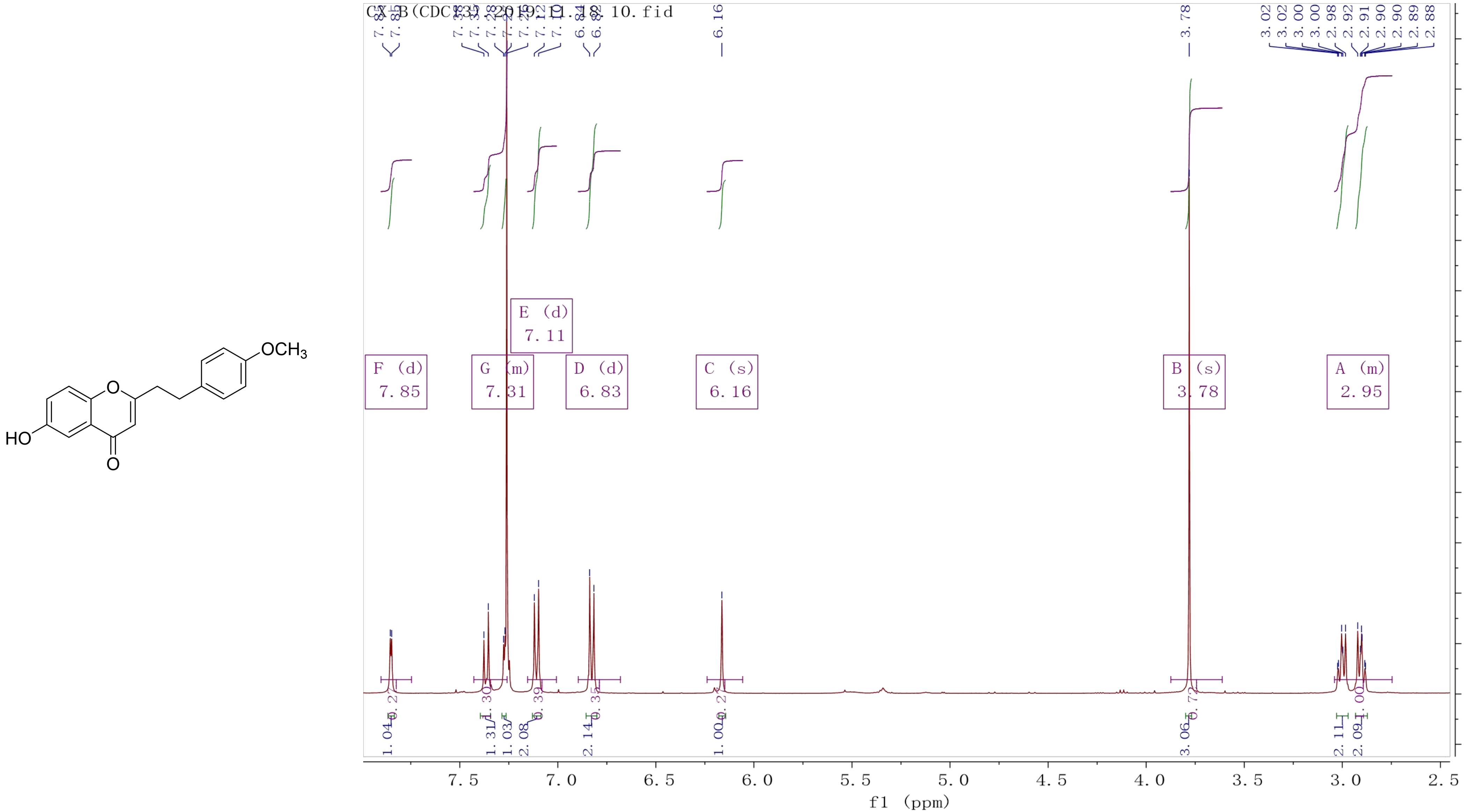

Fig. 29 ^{1}HNMR of 5-hydroxy-2-(4-methoxyphenethyl)-4H-chromen-4-one (400MHz, $CDCl_3$)

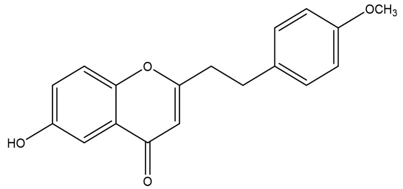

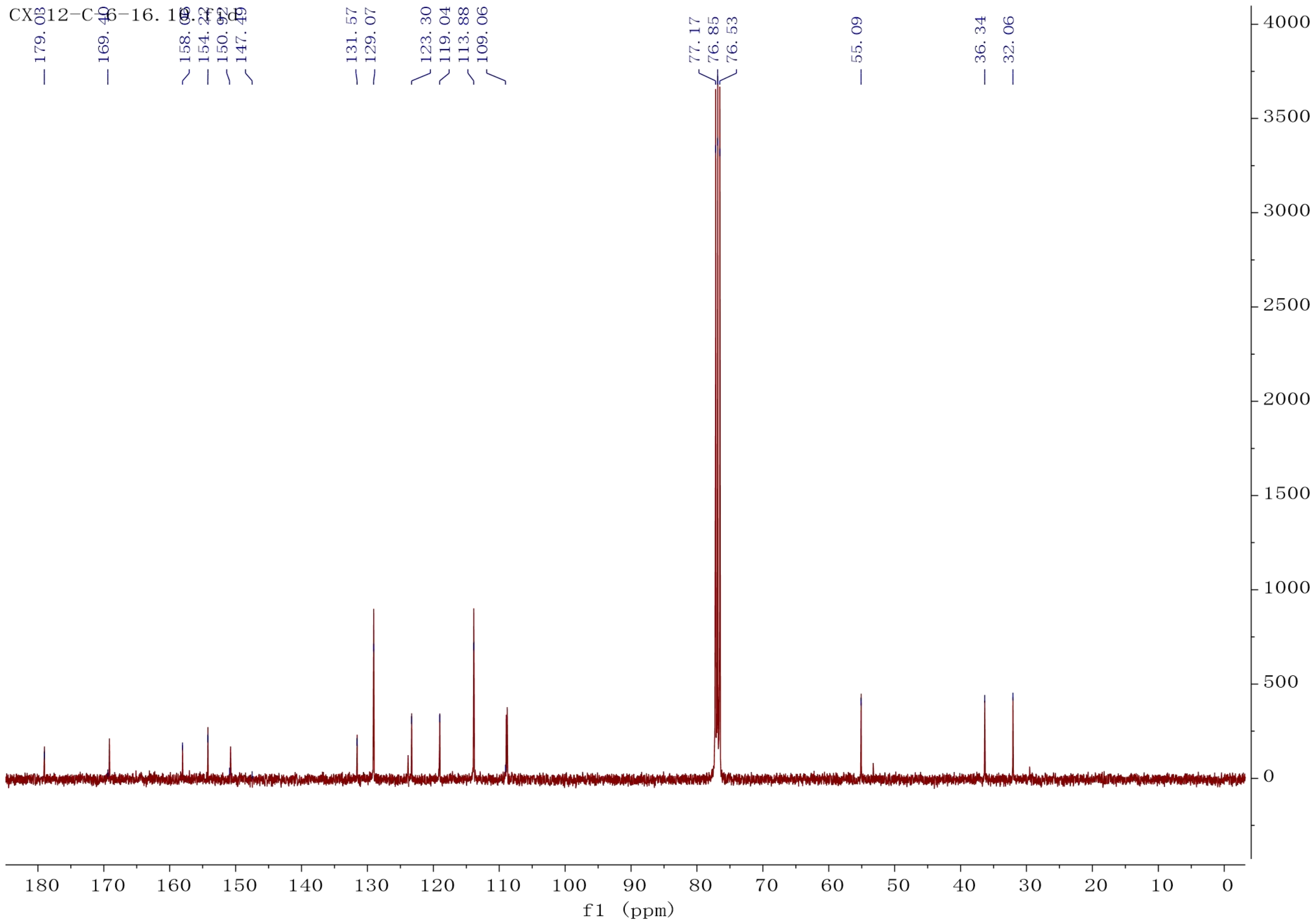

Fig. 30 ^{13}CNMR of 5-hydroxy-2-(4-methoxyphenethyl)-4H-chromen-4-one (100 MHz, $CDCl_3$)

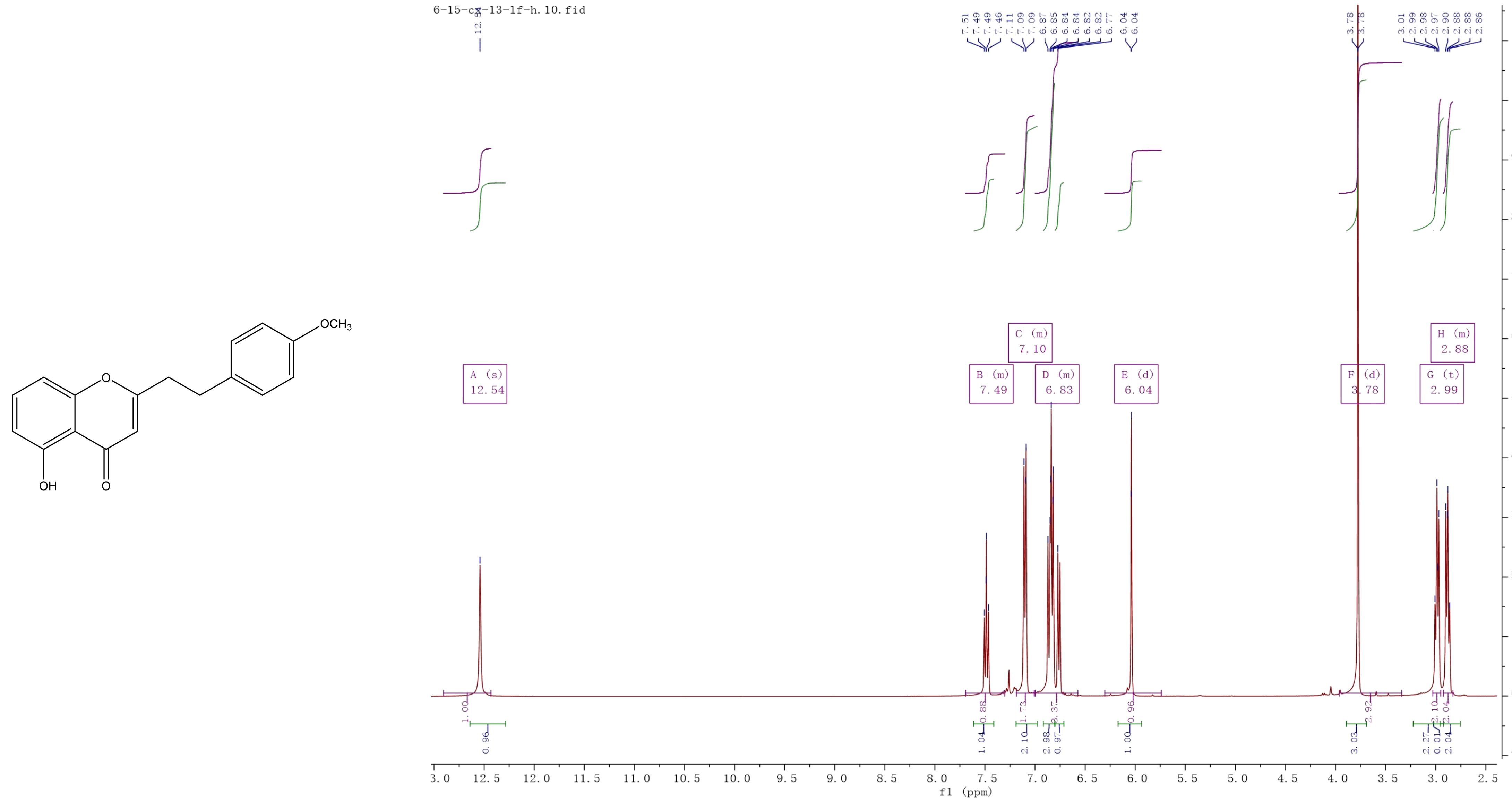

Fig. S32 ^{1}HNMR of 5-hydroxy-2-(4-methoxyphenethyl)-4H-chromen-4-one (400MHz, $CDCl_3$)

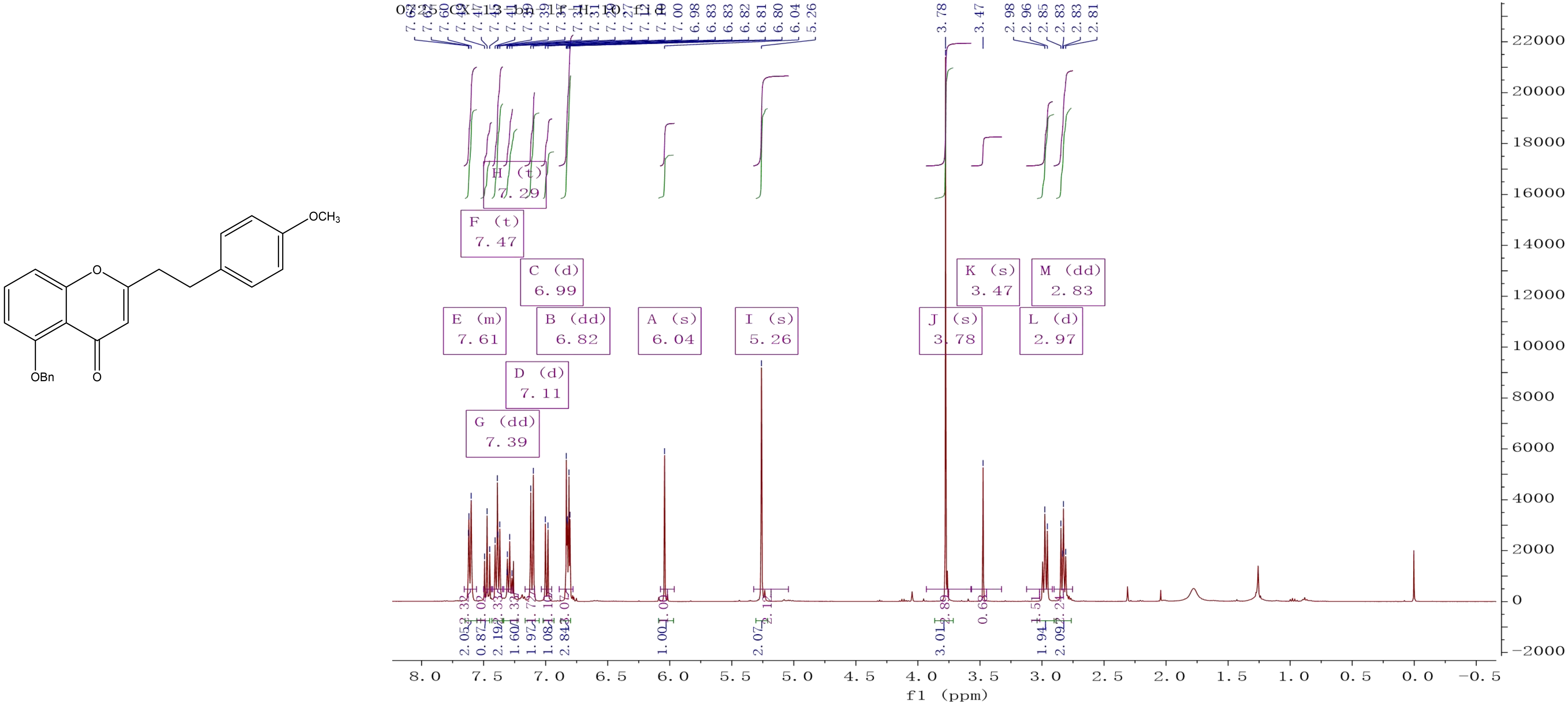

Fig.31 5-(benzyloxy)-2-(4-methoxyphenethyl)-4H-chromen-4-one (400MHz, $CDCl_3$))

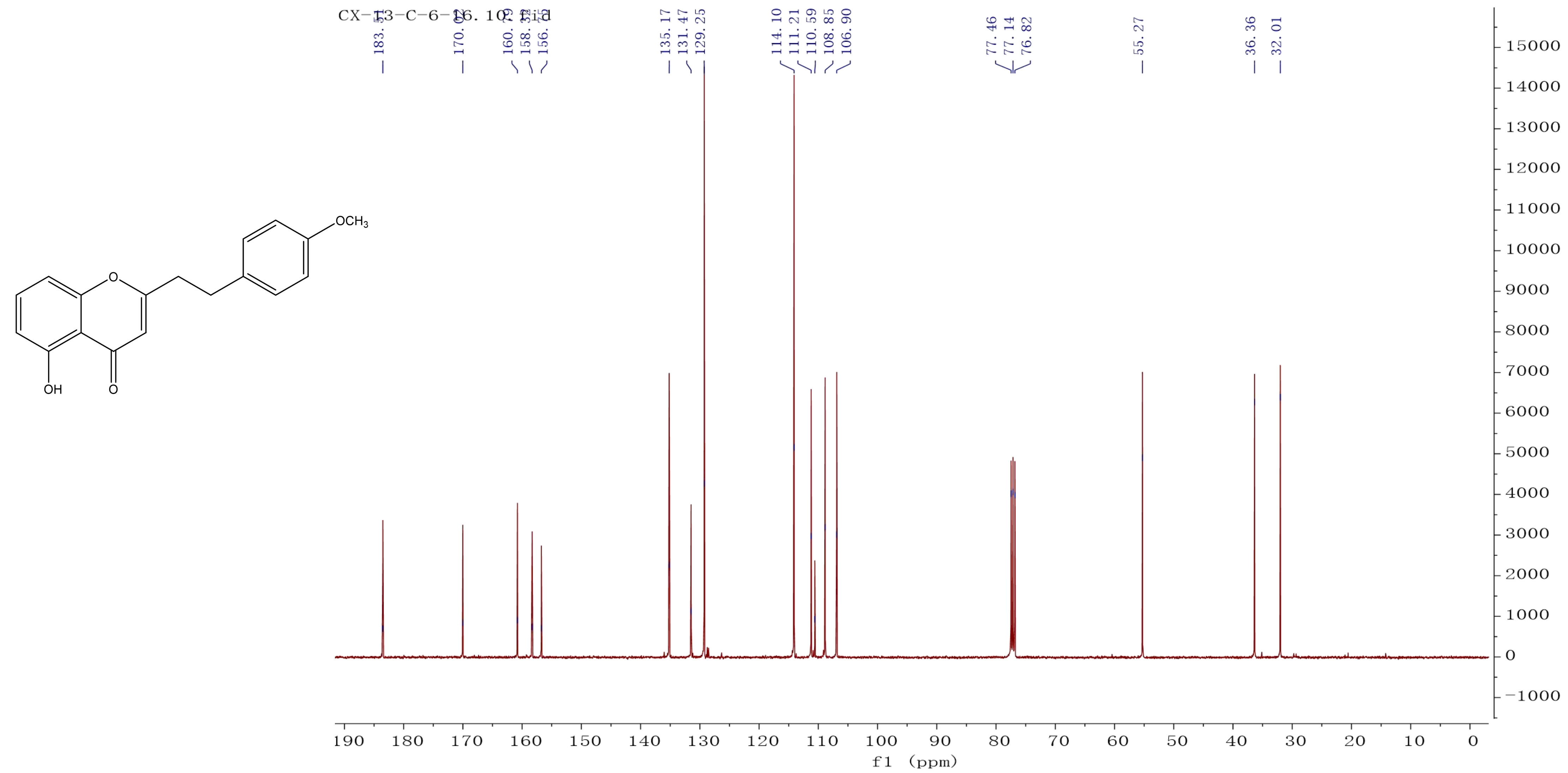

Fig. S33 ^{13}CNMR of 5-hydroxy-2-(4-methoxyphenethyl)-4H-chromen-4-one (100 MHz, $CDCl_3$)

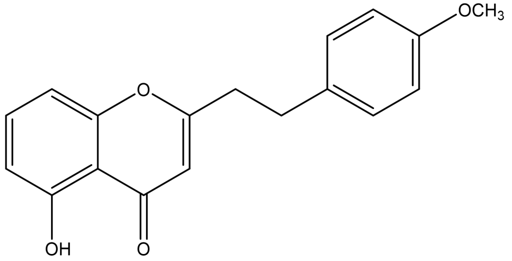

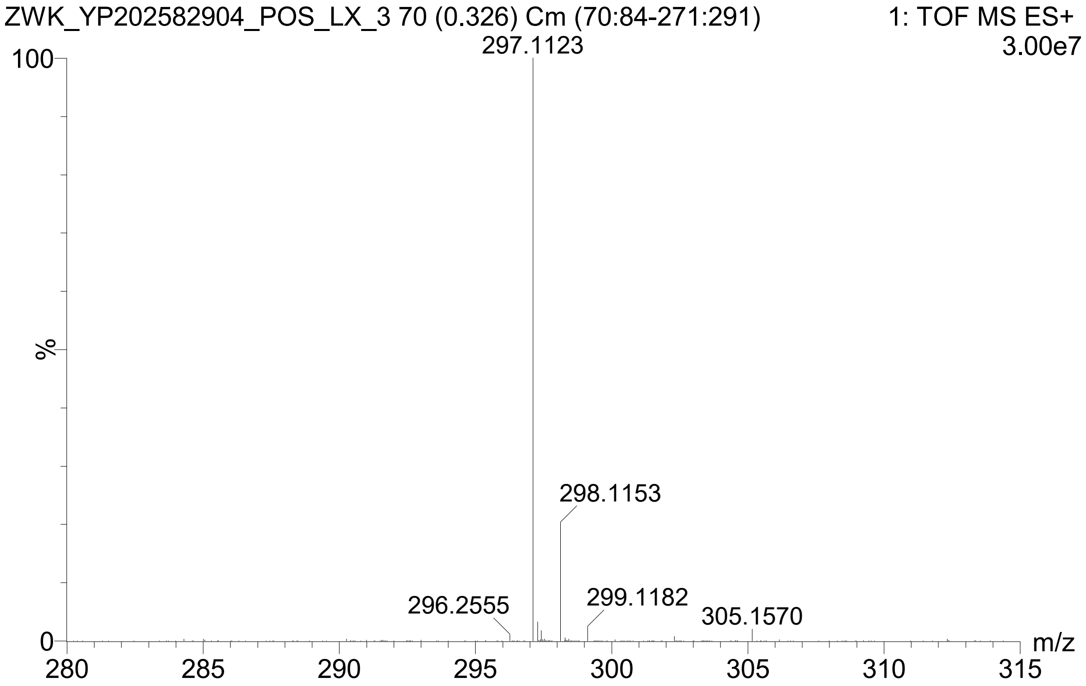

Fig. S34 HRMS of 5-hydroxy-2-(4-methoxyphenethyl)-4H-chromen-4-one (400MHz, $CDCl_3$)

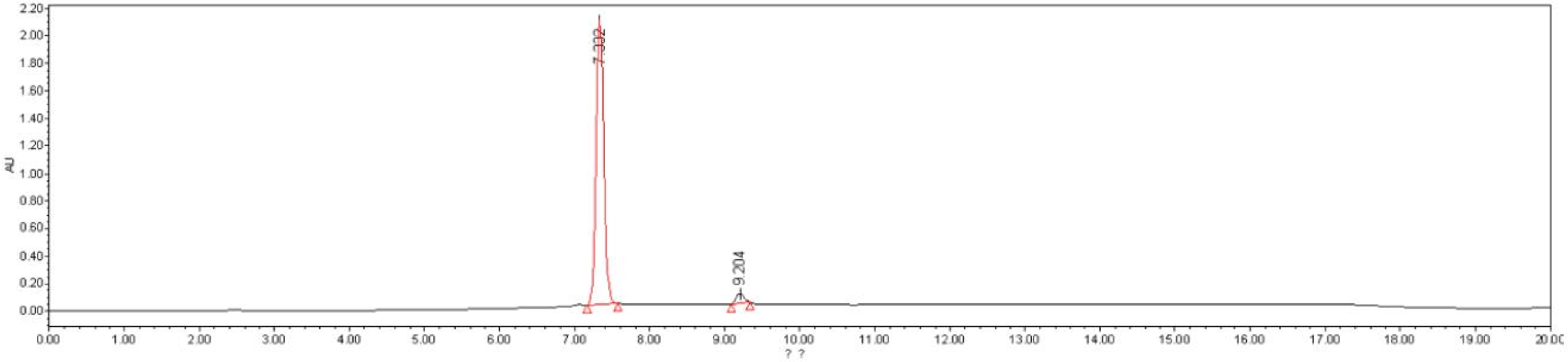

		Retention time	Area	Area ratio (%)
1		7.332	14324738	96.67
2	Impurity	9.204	493589	3.33

Fig. S35 HPLC of 5-hydroxy-2-(4-methoxyphenethyl)-4H-chromen-4-one (R.T.= 7.332 min, purity=96.67%)

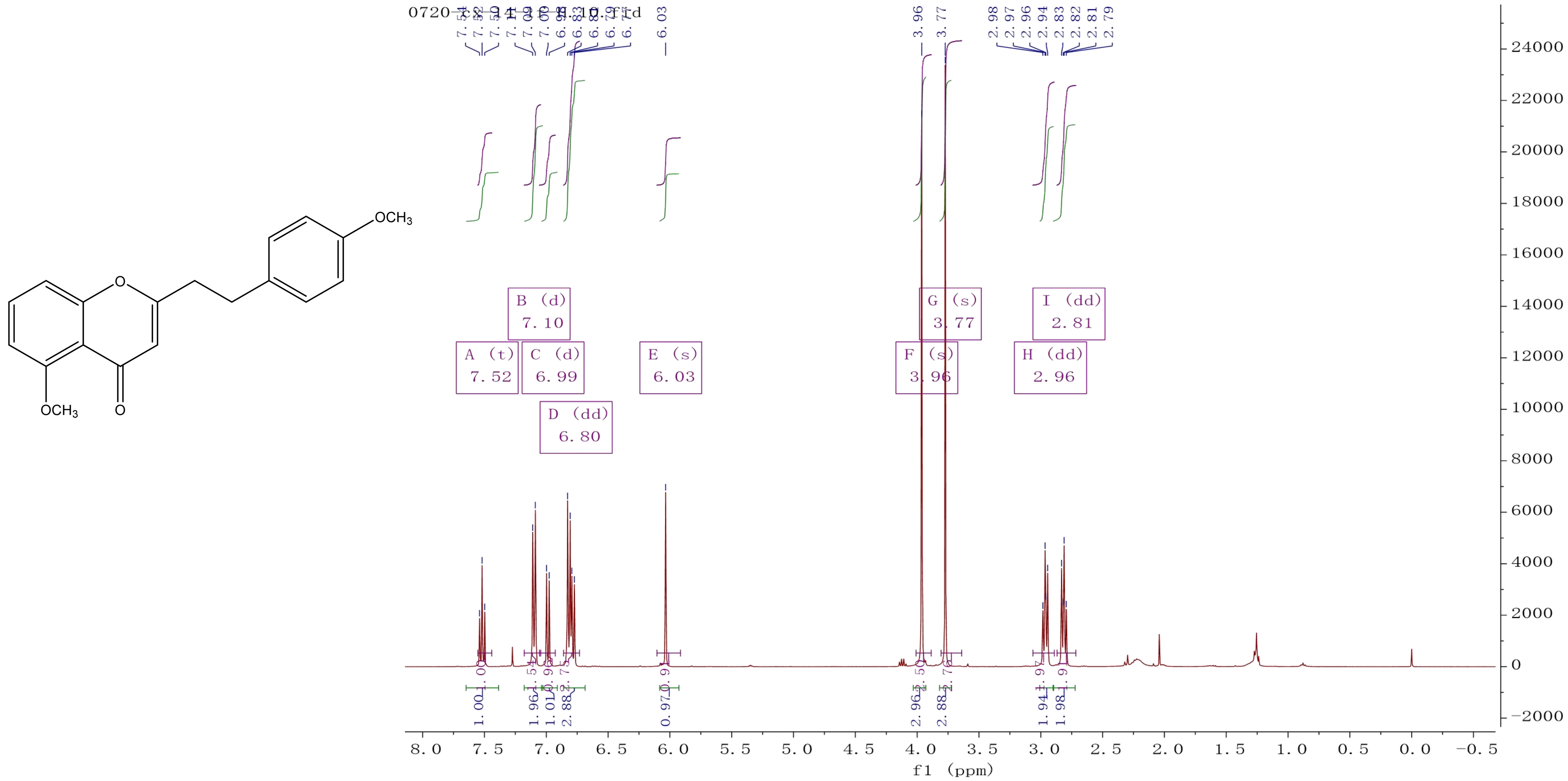

Fig.S36 ^{1}HNMR of 5-methoxy-2-(4-methoxyphenethyl)-4H-chromen-4-one (400MHz, $CDCl_3$)

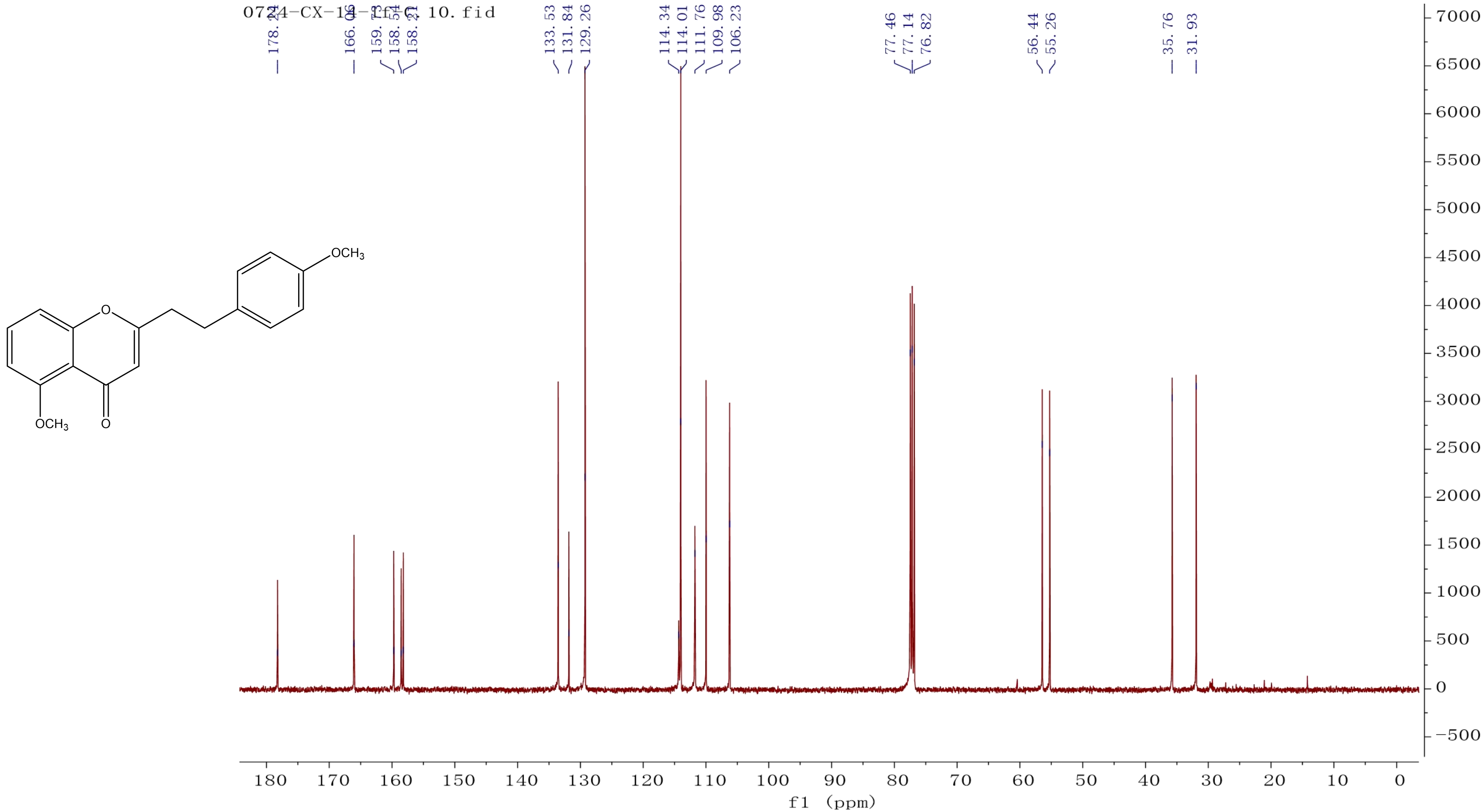

Fig.S37 ^{1}HNMR of 5-methoxy-2-(4-methoxyphenethyl)-4H-chromen-4-one (100 MHz, $CDCl_3$)

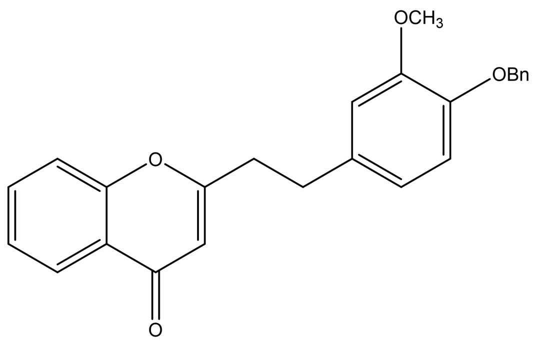

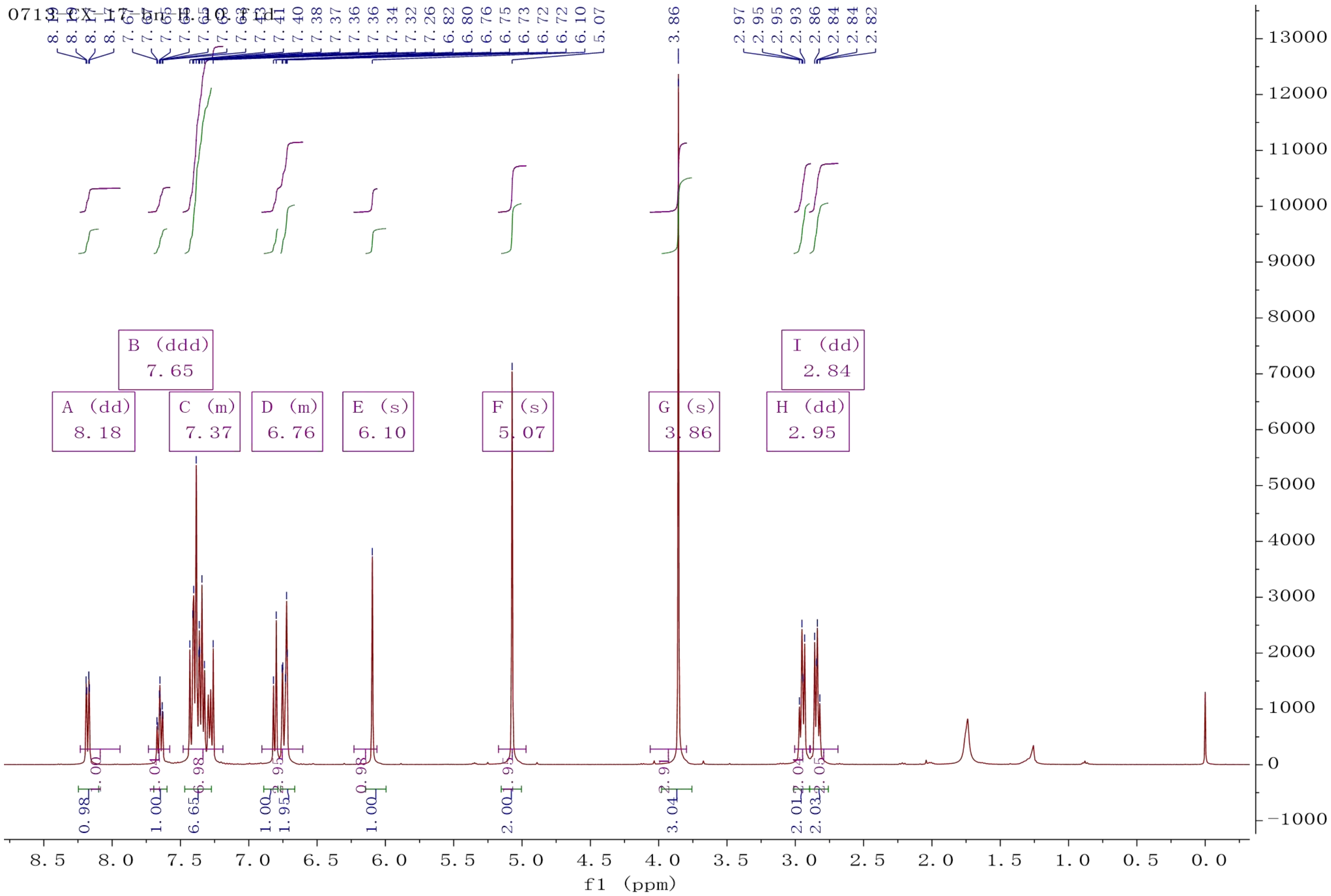

Fig. S 38 ^{1}HNMR of 2-(4-(benzyloxyl)-3-methoxyphenethyl)-4H-chromen-4-one (400MHz, $CDCl_3$)

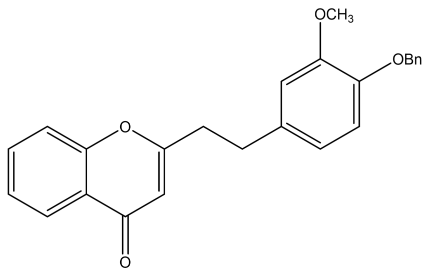

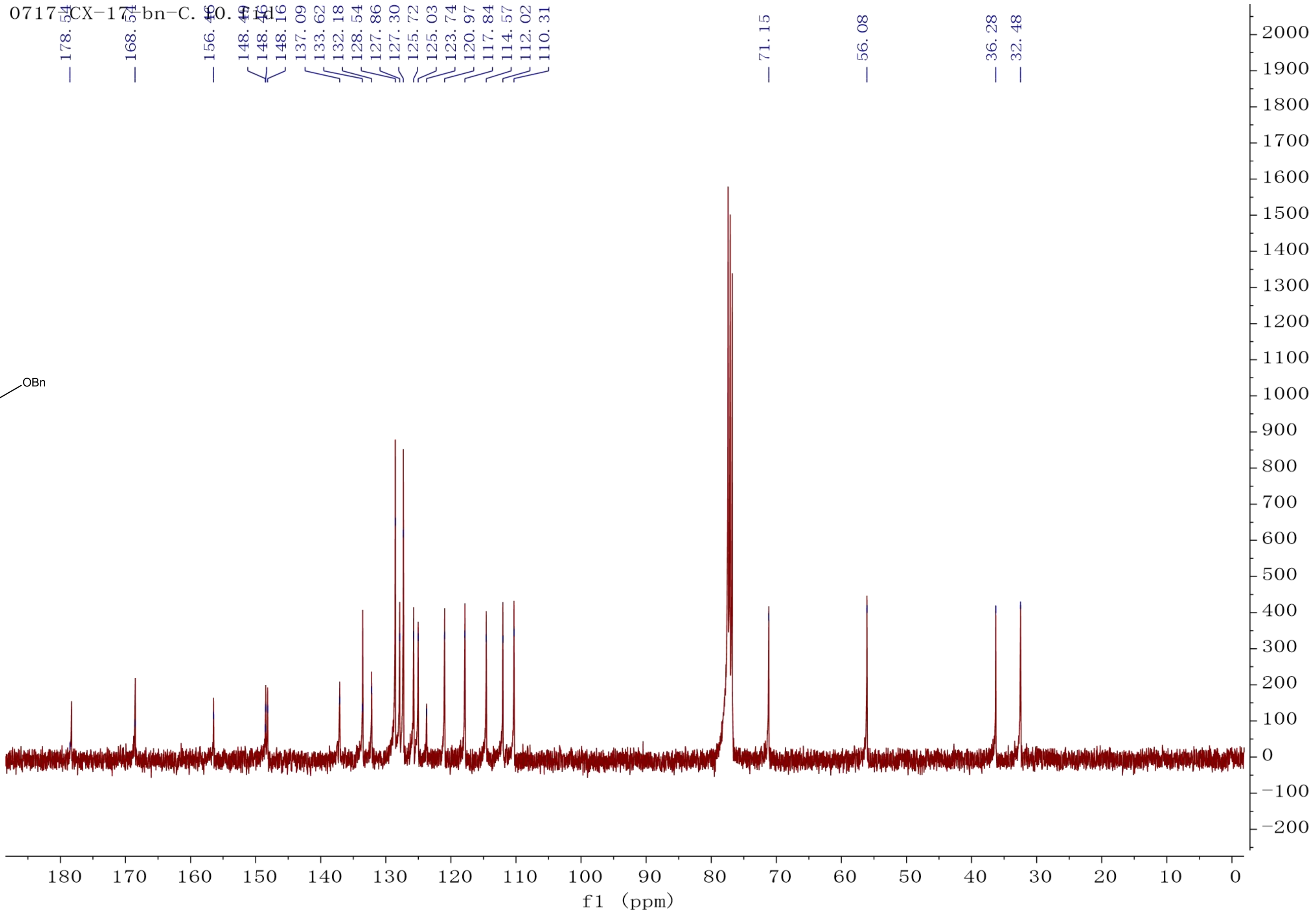

Fig.39 ^{13}CNMR of 2-(4-(benzyloxyl)-3-methoxyphenethyl)-4H-chromen-4-one(100MHz, $CDCl_3$)

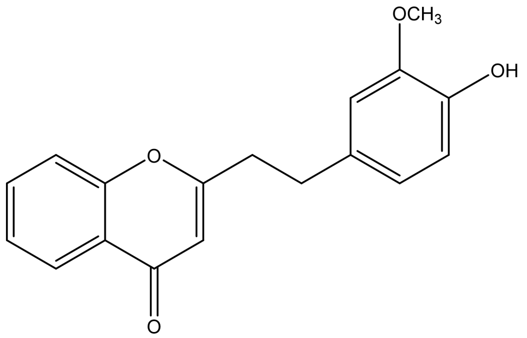

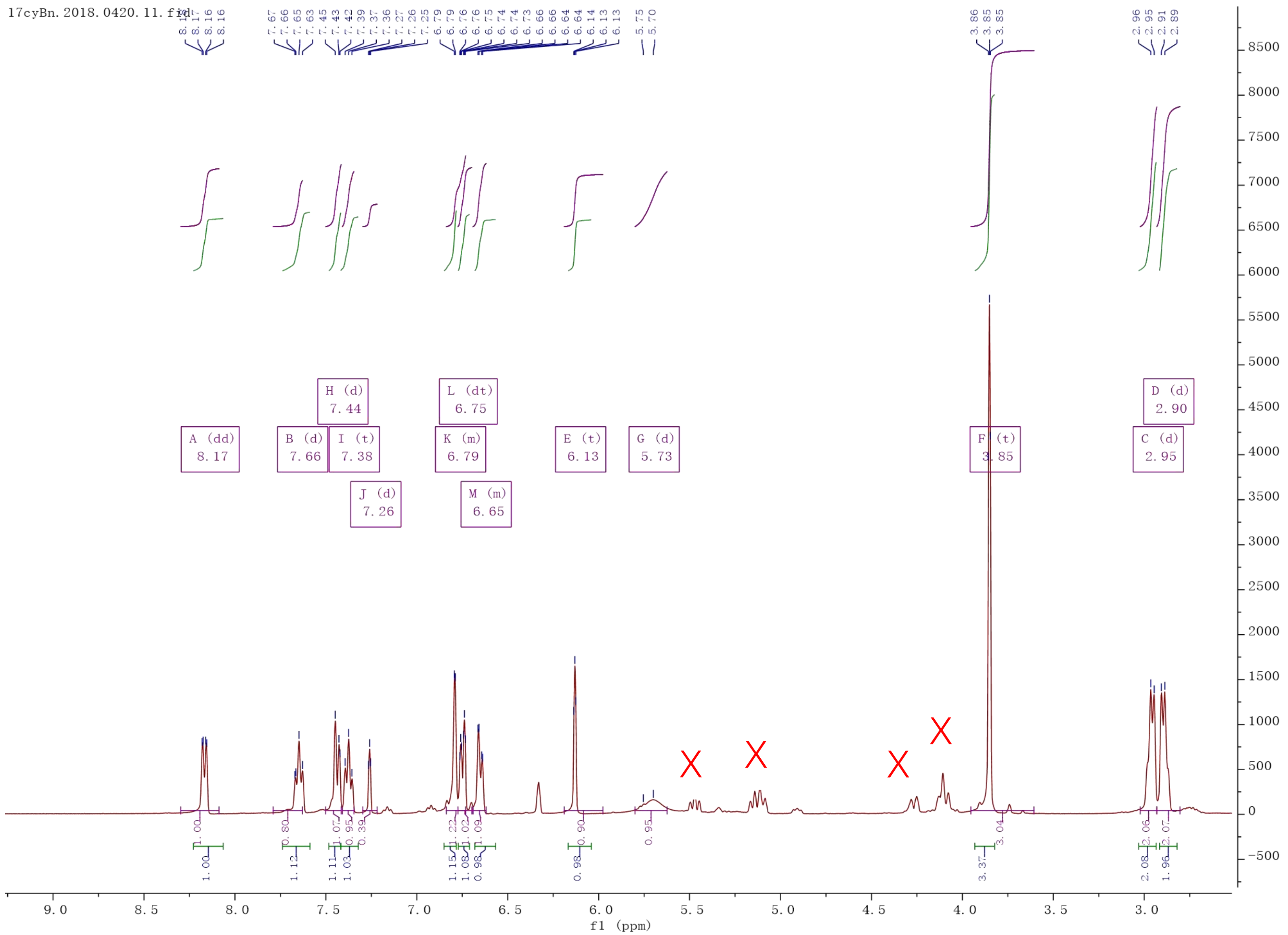

Fig.S40 ^{1}HNMR of 2-(4-(hydroxyl)-3-methoxyphenethyl)-4H-chromen-4-one (400 MHz, $CDCl_3$)

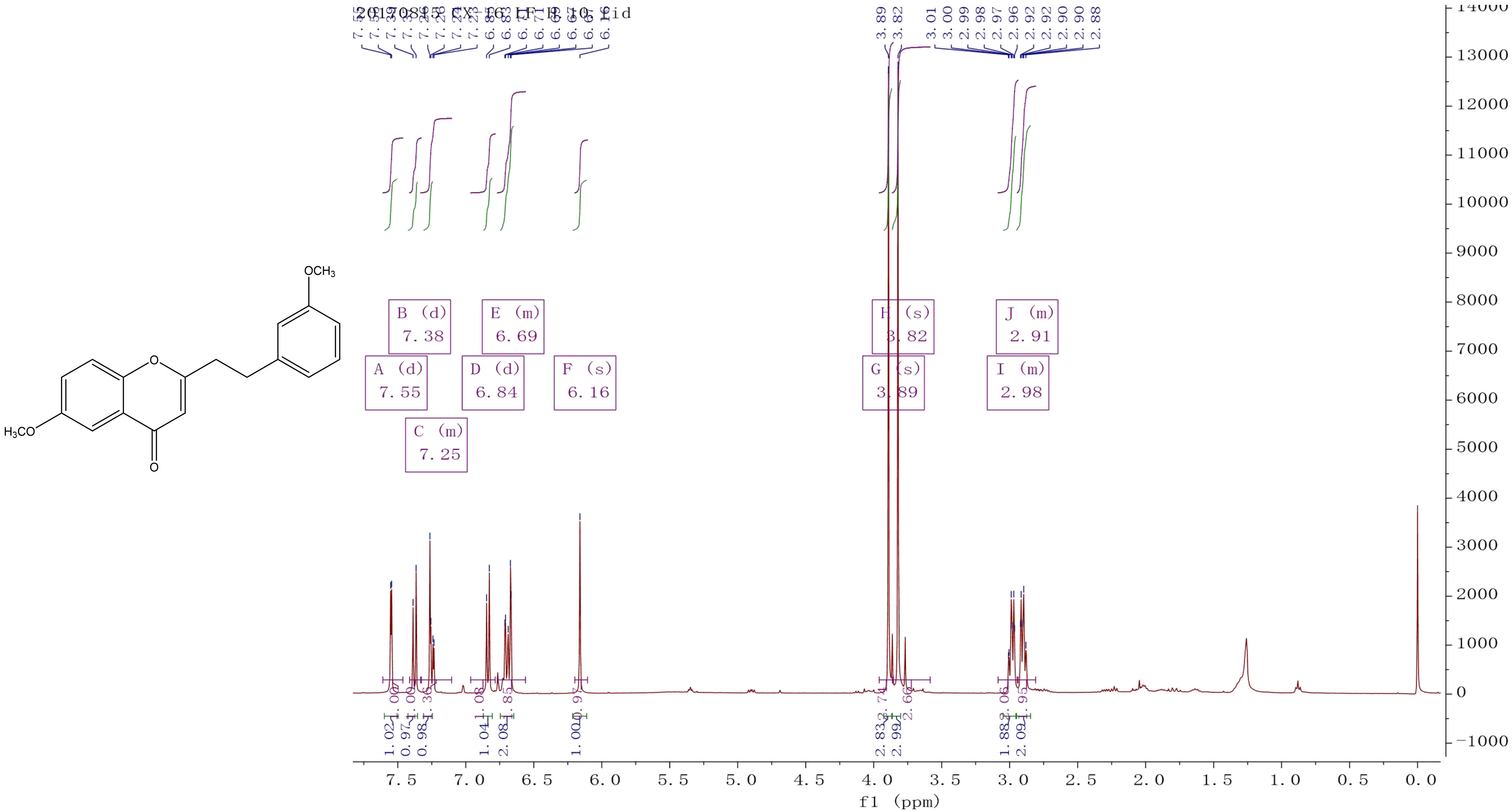

Fig. S41.¹HNMR of 6-methoxy-2-(3-methoxyphenethyl)-4H-chromen-4-one (400MHz, $CDCl_3$)

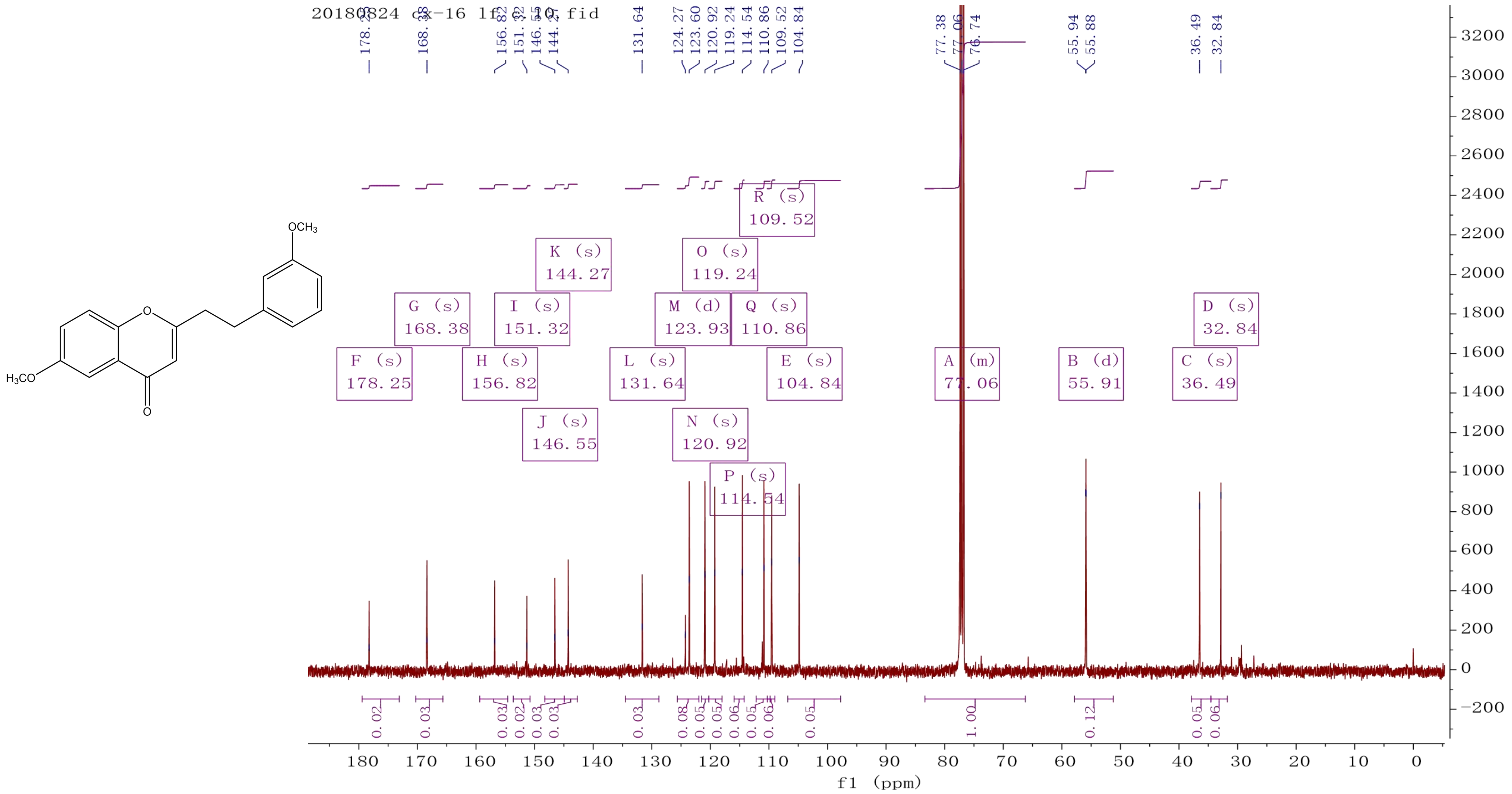

Fig. S42.^{13}CNMR of 6-methoxy-2-(3-methoxyphenethyl)-4H-chromen-4-one (100MHz, $CDCl_3$)

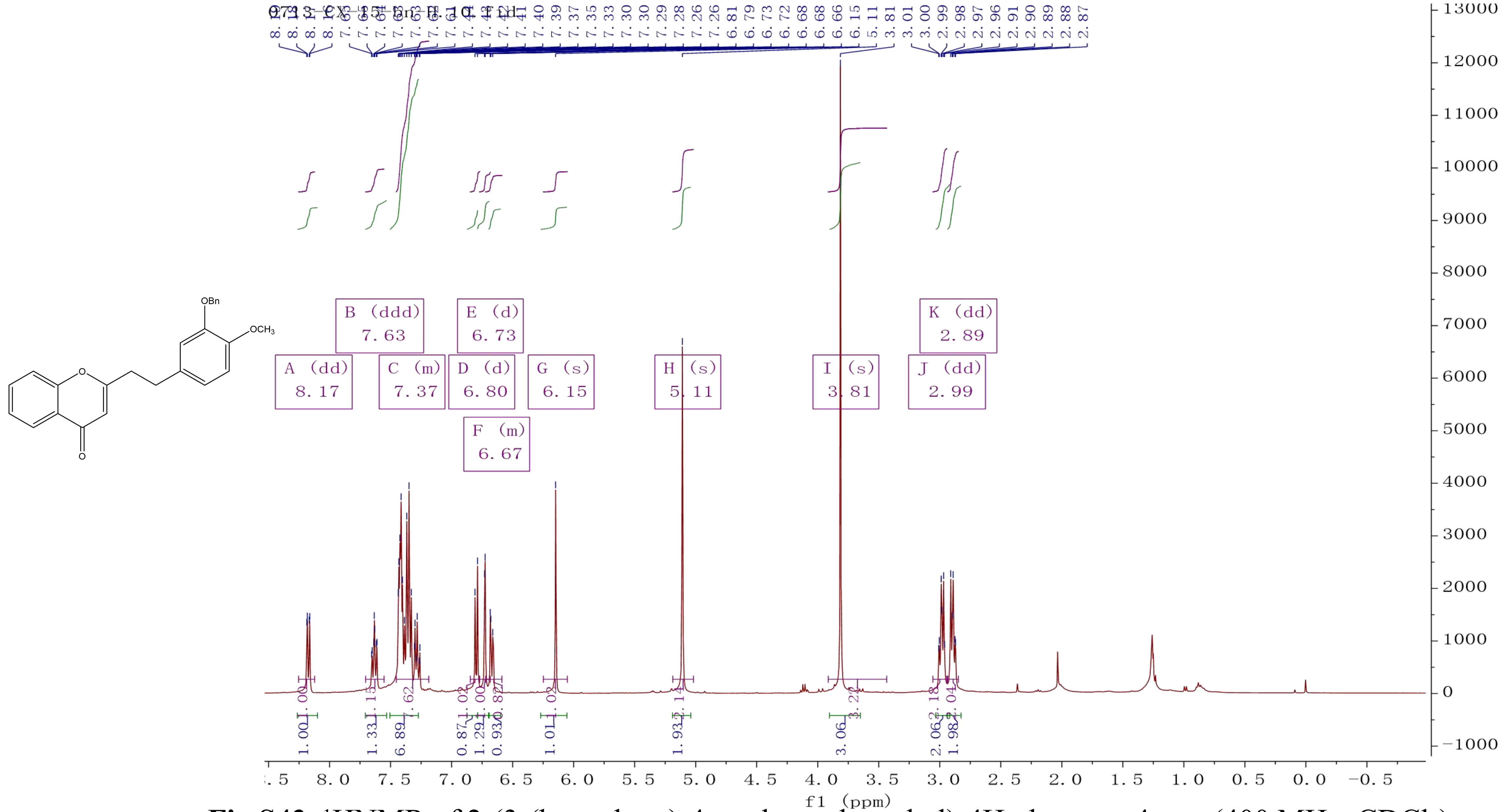

Fig S43. ^{1}HNMR of 2-(3-(benzyloxy)-4-methoxyphenethyl)-4H-chromen-4-one (400 MHz, $CDCl_3$)

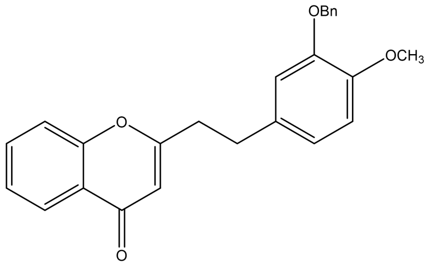

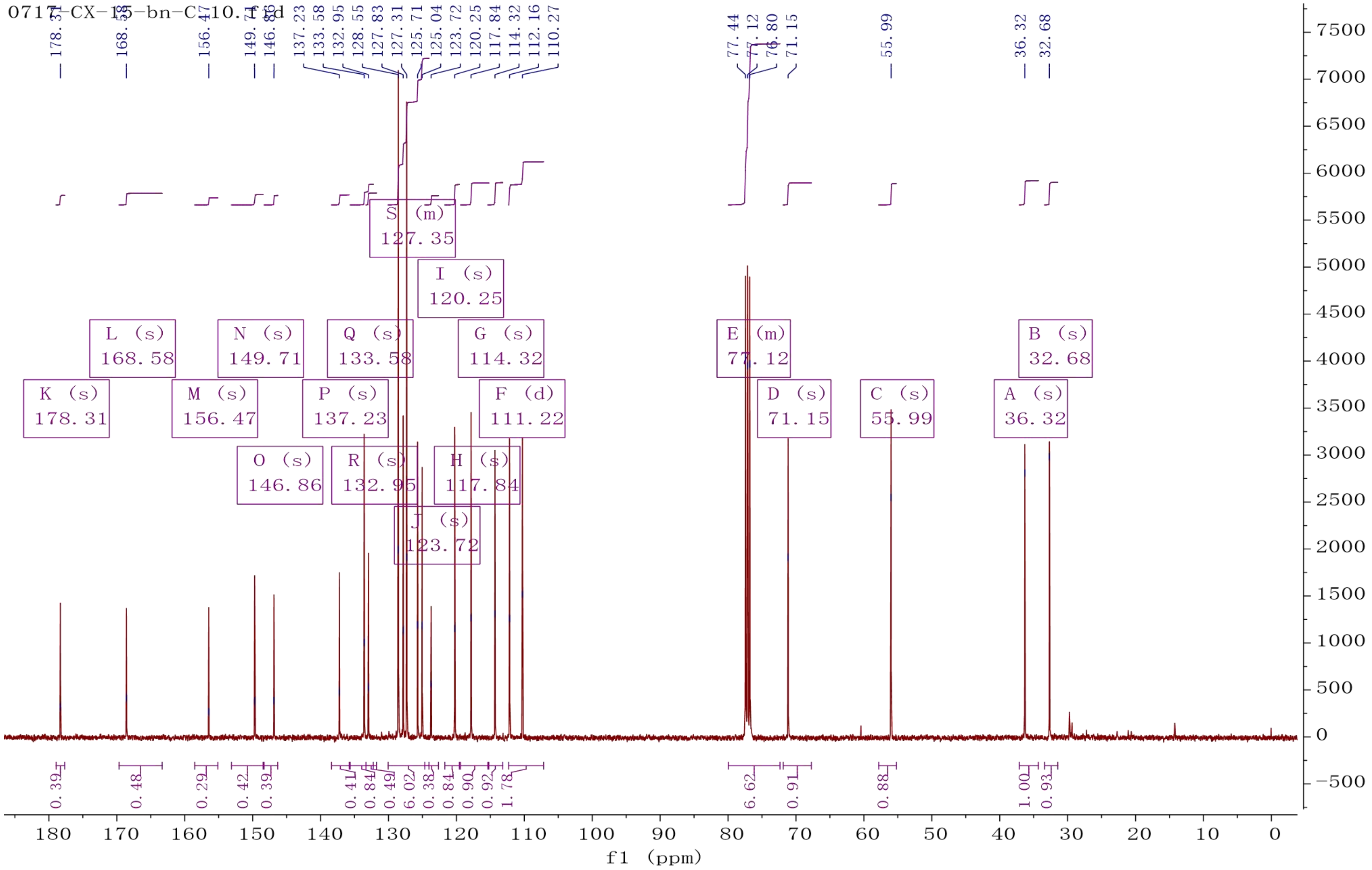

Fig.S44 ^{13}CNMR of 2-(3-(benzyloxy)-4-methoxyphenethyl)-4H-chromen-4-one (100MHz, $CDCl_3$)

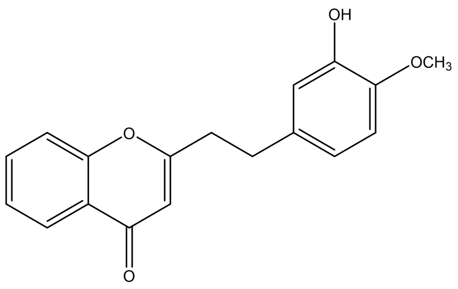

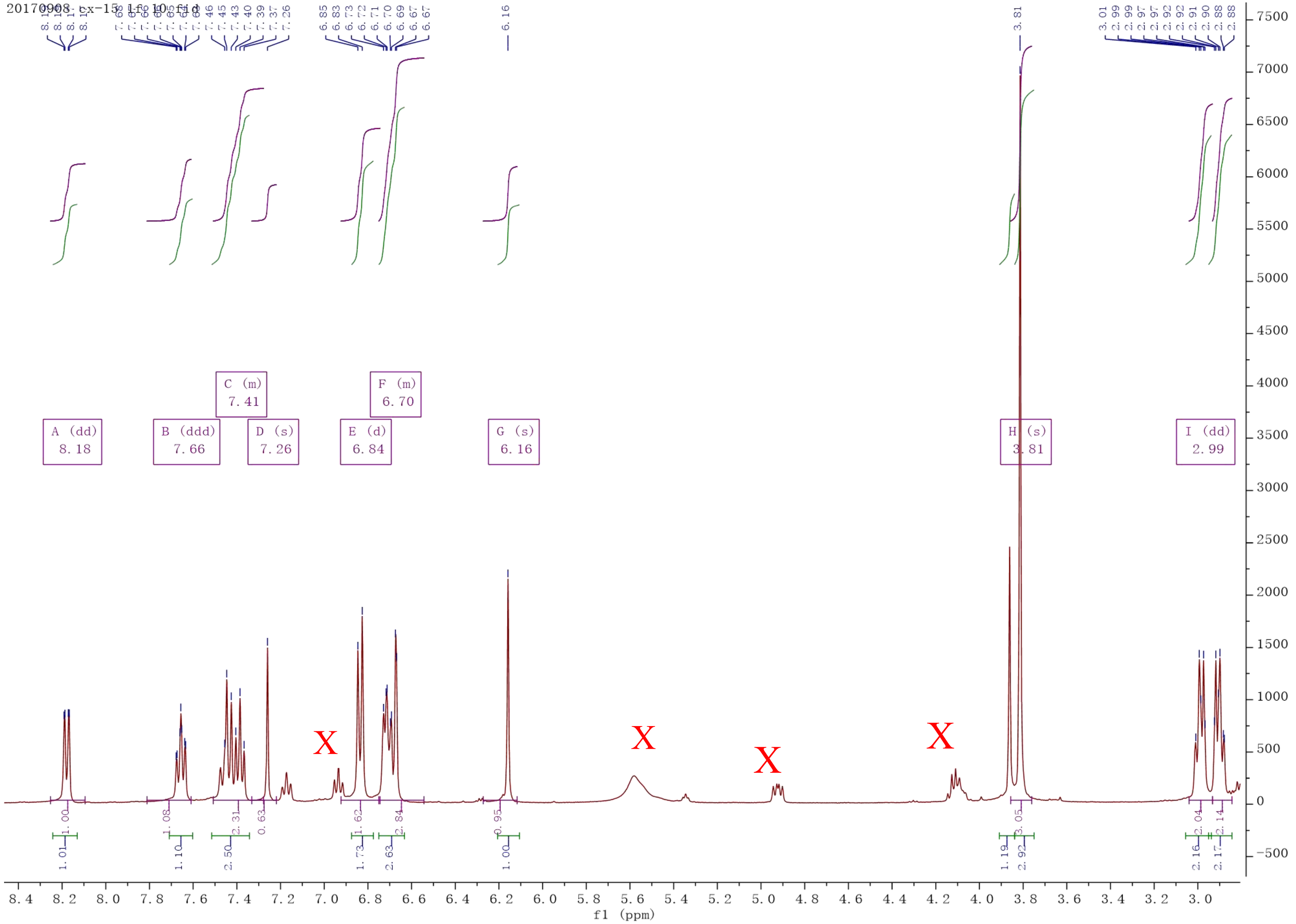

Fig.S45 2-(3-hydroxyl-4-methoxyphenethyl)-4H-chromen-4-one (400 MHz, $CDCl_3$)

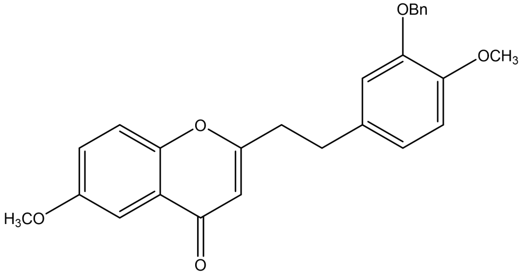

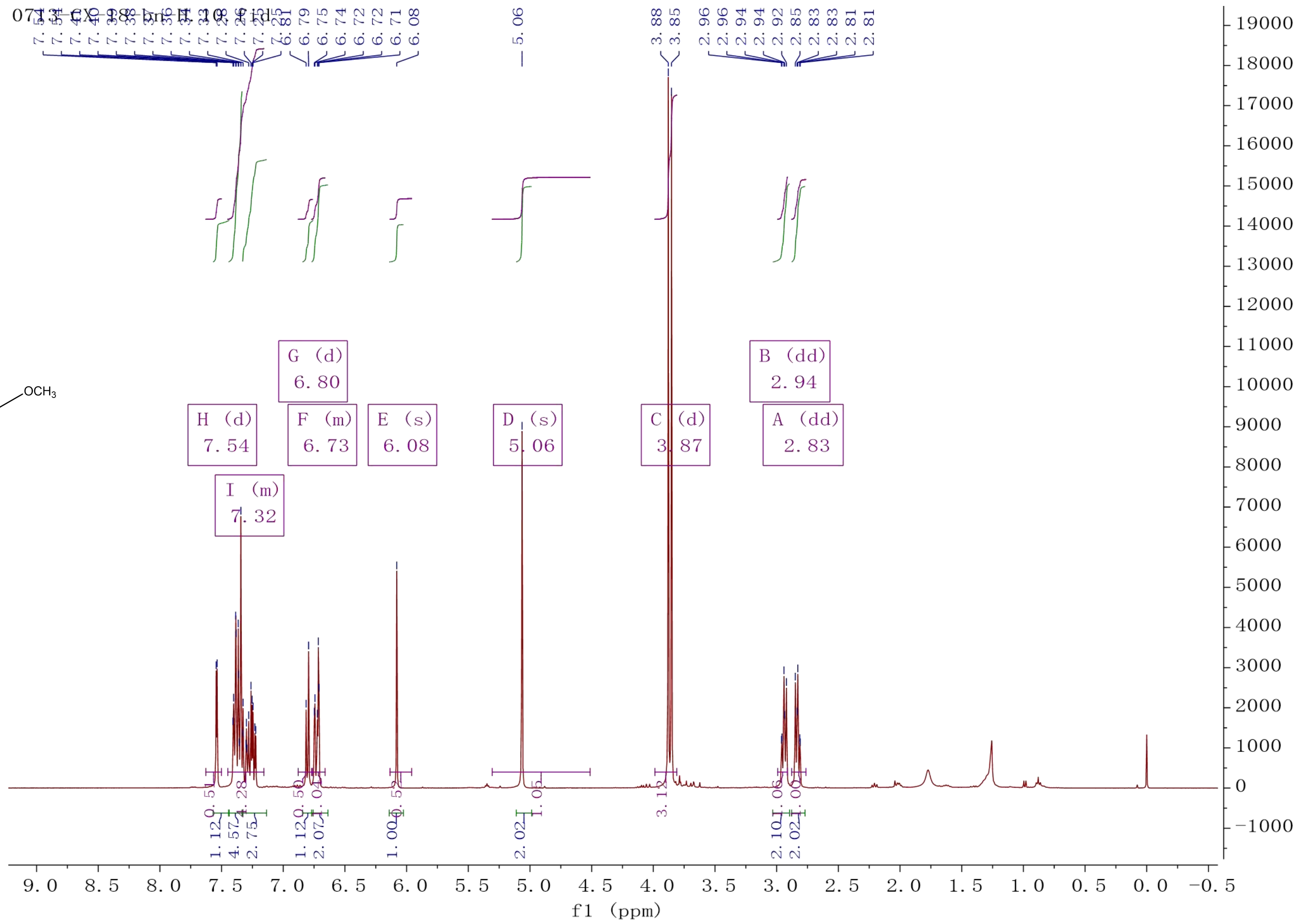

Fig. S46 ^{1}HNMR of 2-(3-(benzyloxy)-4-methoxyphenethyl)-6-methoxy-4H-chromen-4-one (400 MHz, $CDCl_3$)

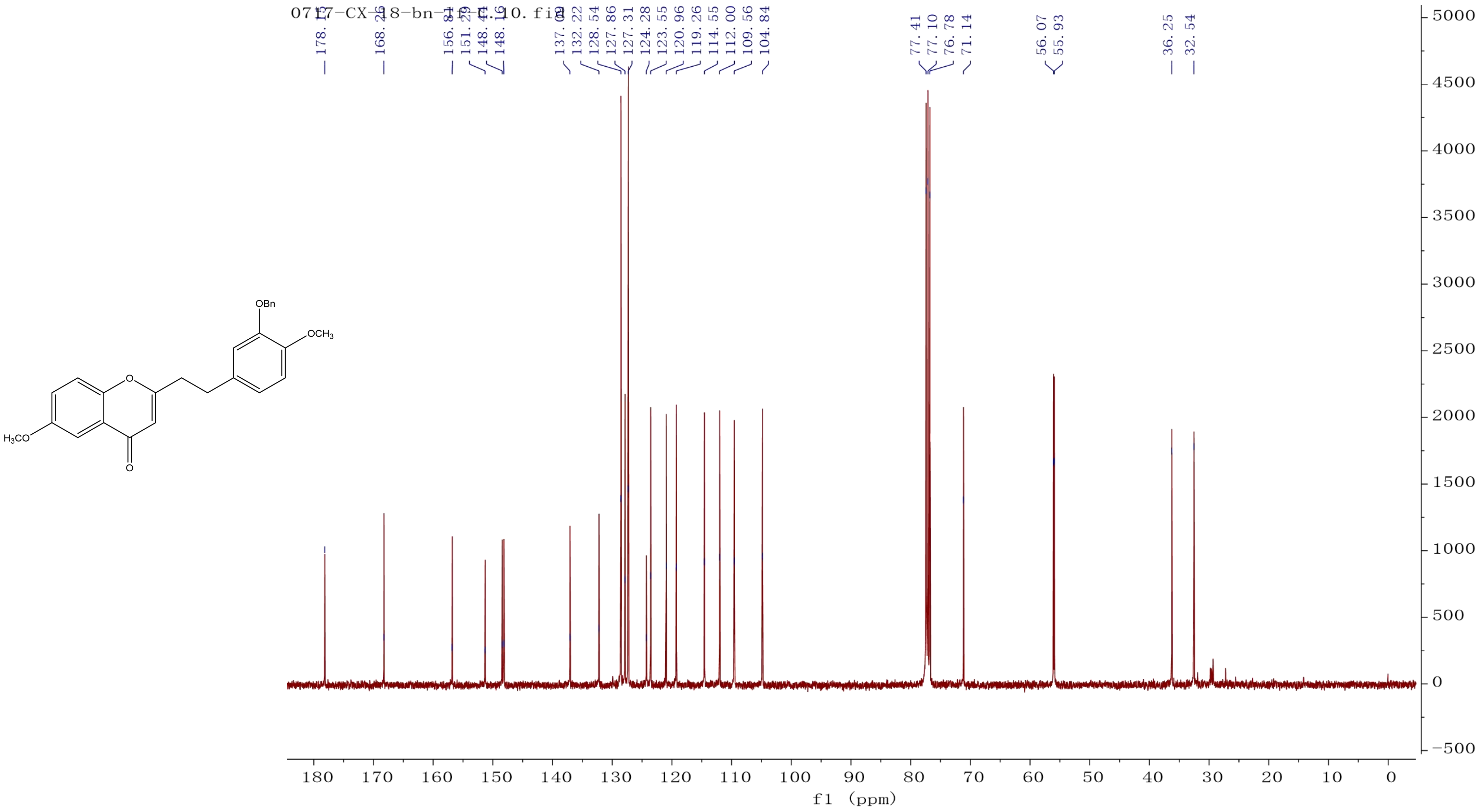

Fig. S47 ^{13}CNMR of 2-(3-(benzyloxy)-4-methoxyphenethyl)-6-methoxy-4H-chromen-4-one (100MHz, $CDCl_3$)

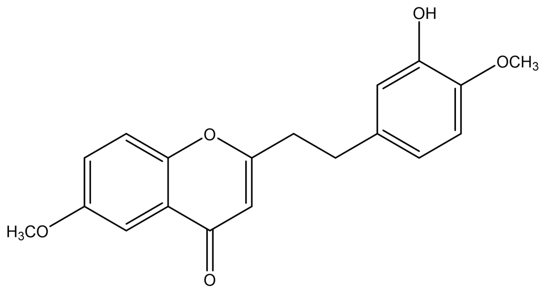

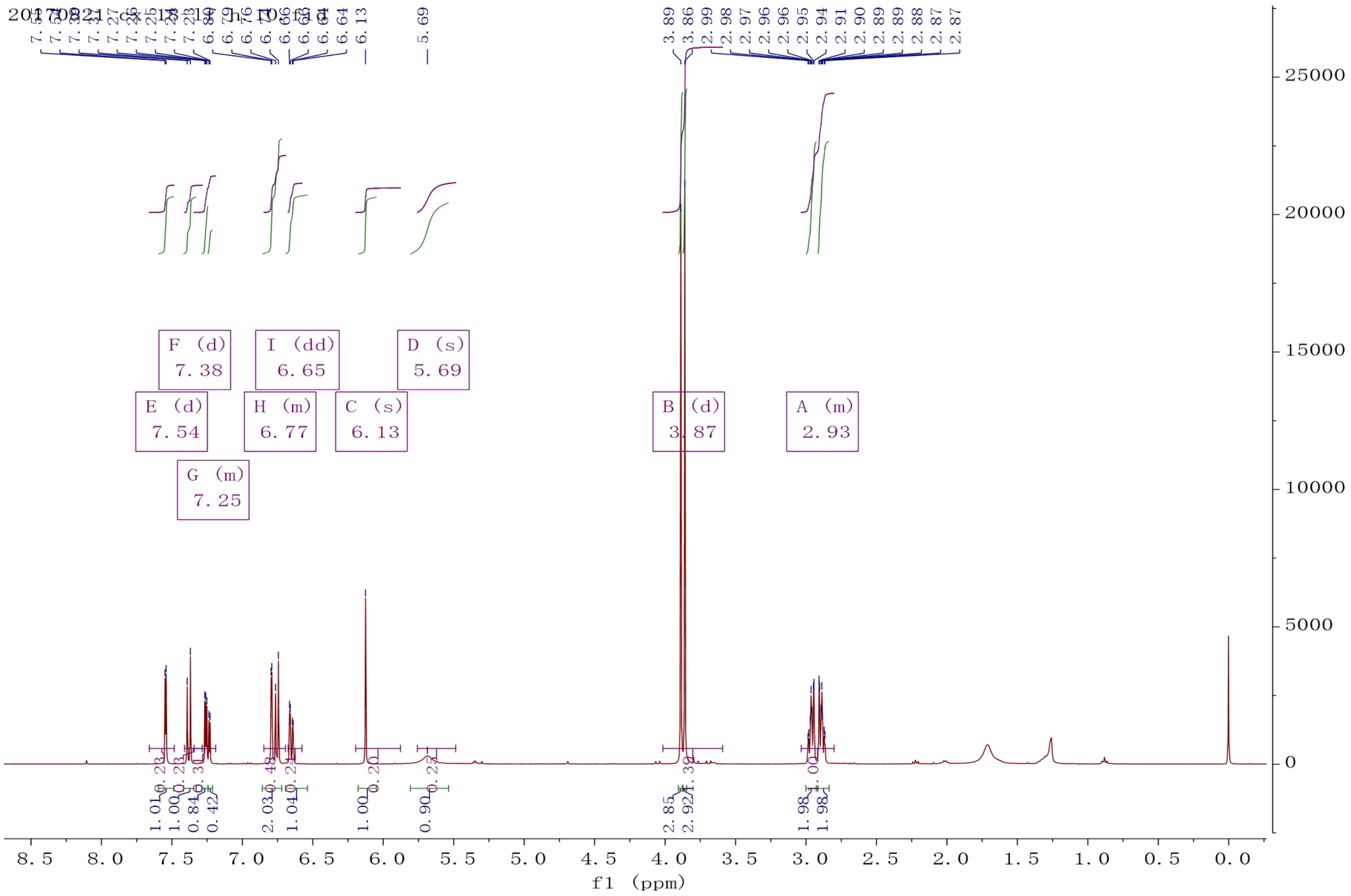

Fig.S48. ^{1}HNMR of 2-(3-hydroxy-4-methoxyphenethyl)-6-methoxy-4H-chromen-4-one (400 MHz, $CDCl_3$)

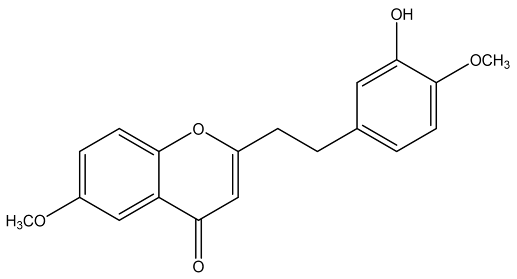

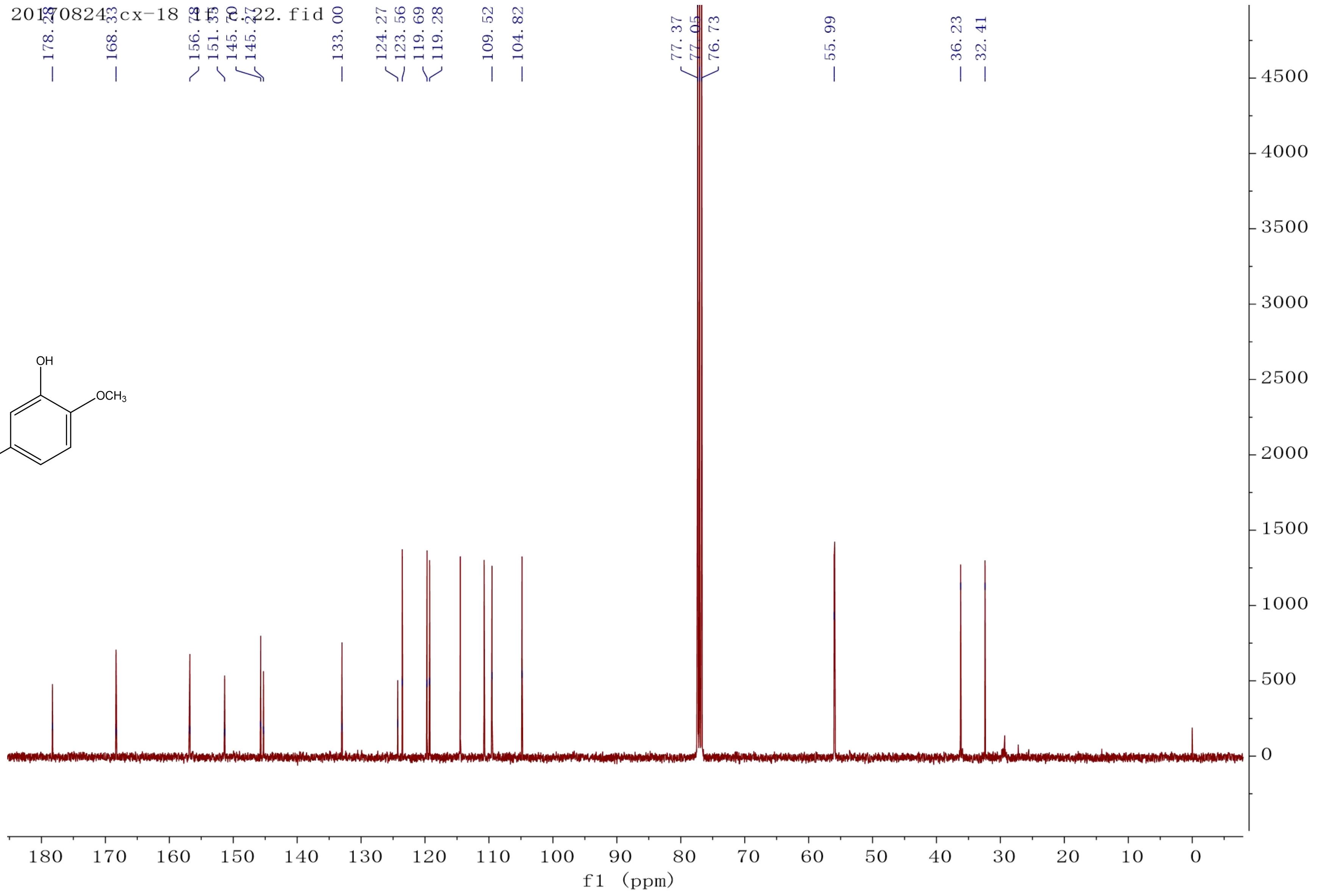

Fig.49. ^{13}C NMR of 2-(3-hydroxy-4-methoxyphenethyl)-6-methoxy-4H-chromen-4-one (100 MHz, $CDCl_3$)

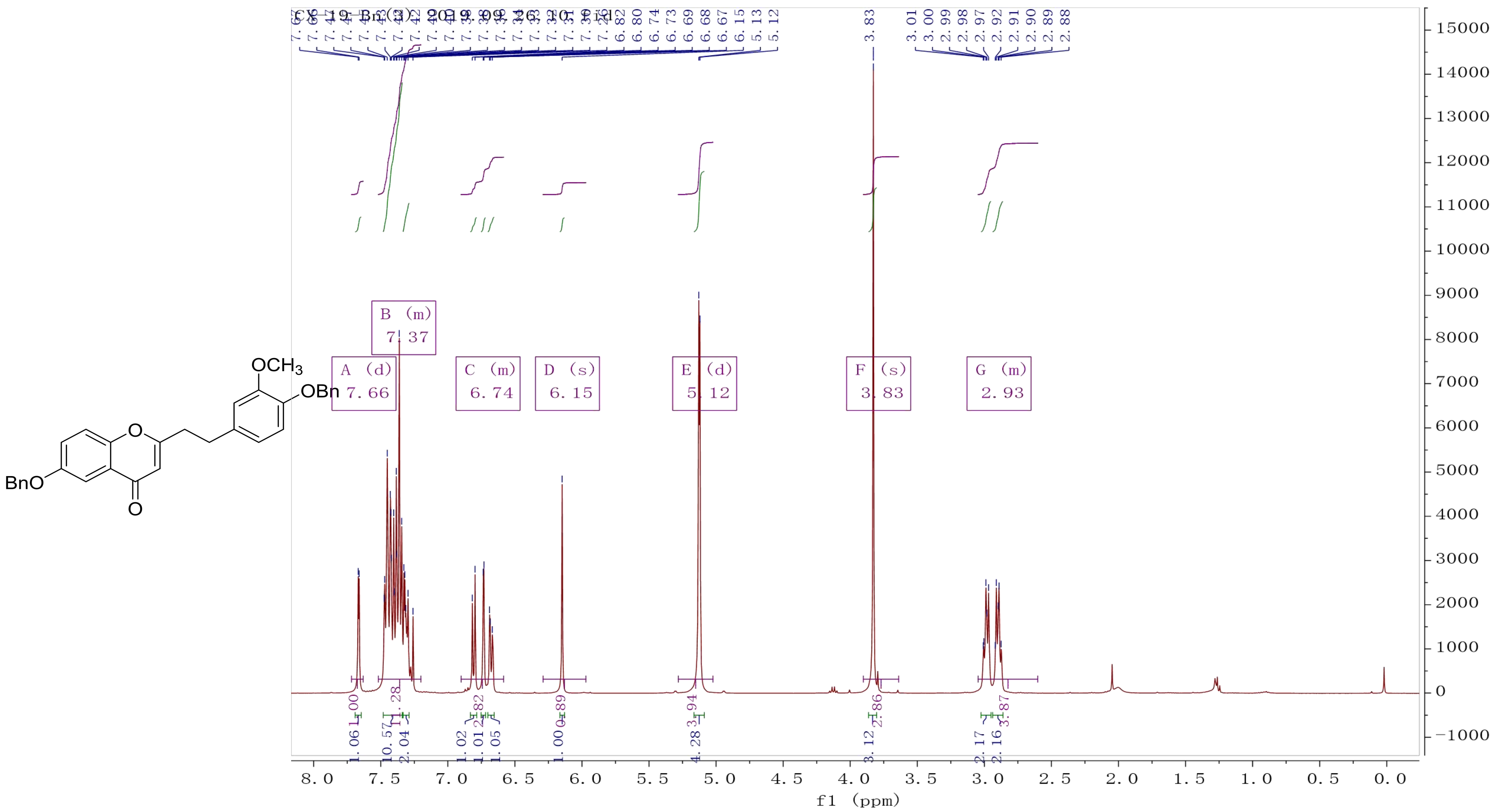

Fig. S 50 ^{1}HNMR of 6-(benzyloxy)-2-(4-(benzyloxy)-3-methoxyphenethyl)-4H-chromen-4-one (400MH, $CDCl_3$)

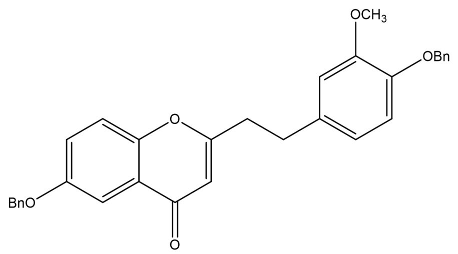

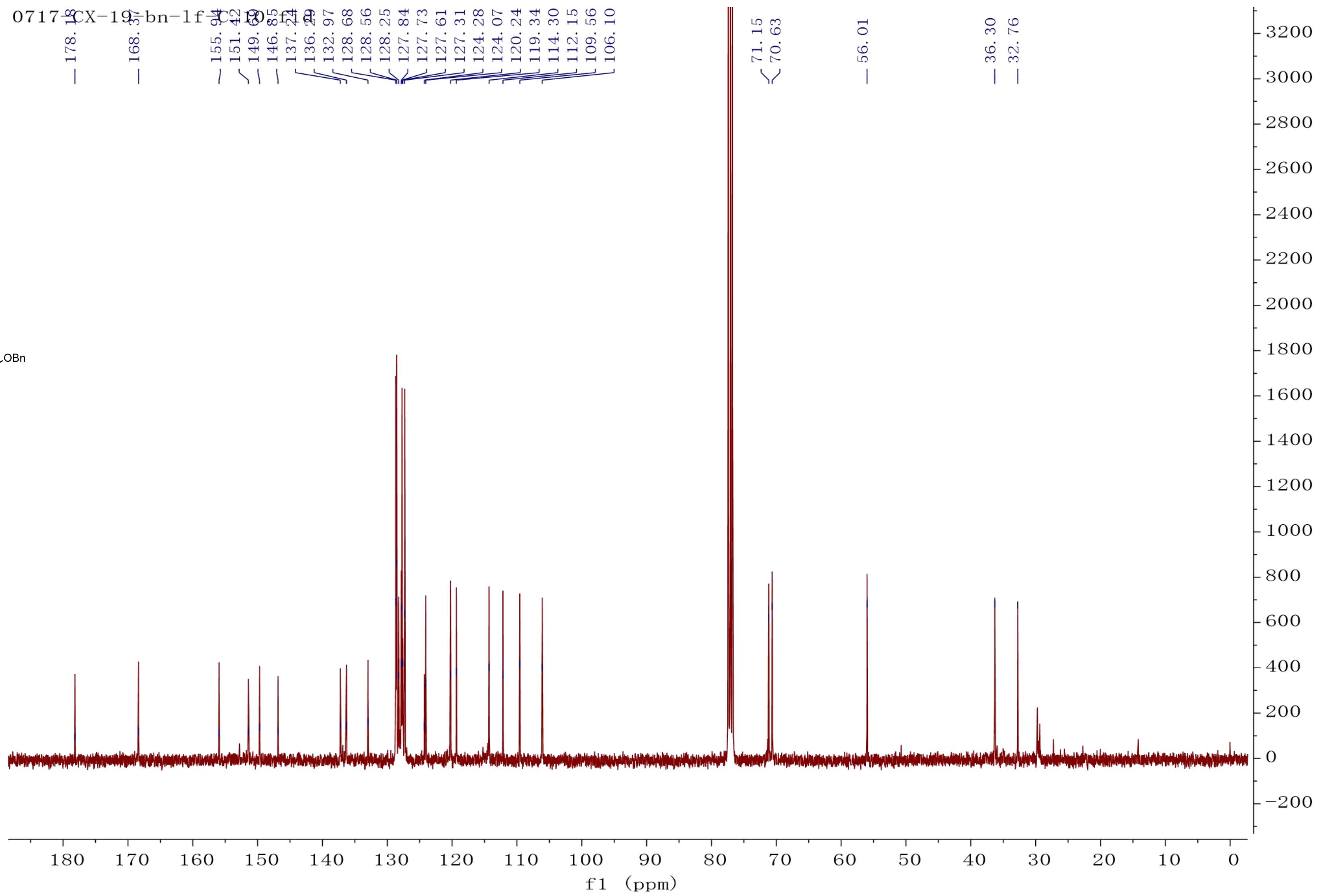

Fig. S 51 ^{13}CNMR of 6-(benzyloxy)-2-(4-(benzyloxy)-3-methoxyphenethyl)-4H-chromen-4-one (100MHz, $CDCl_3$)

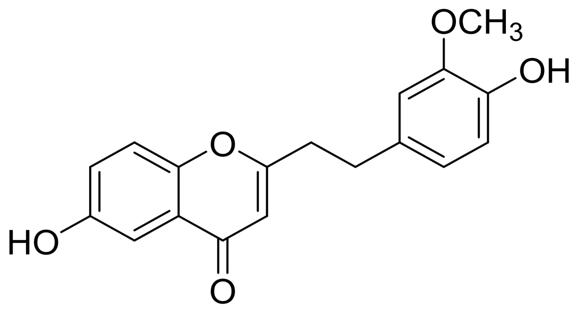

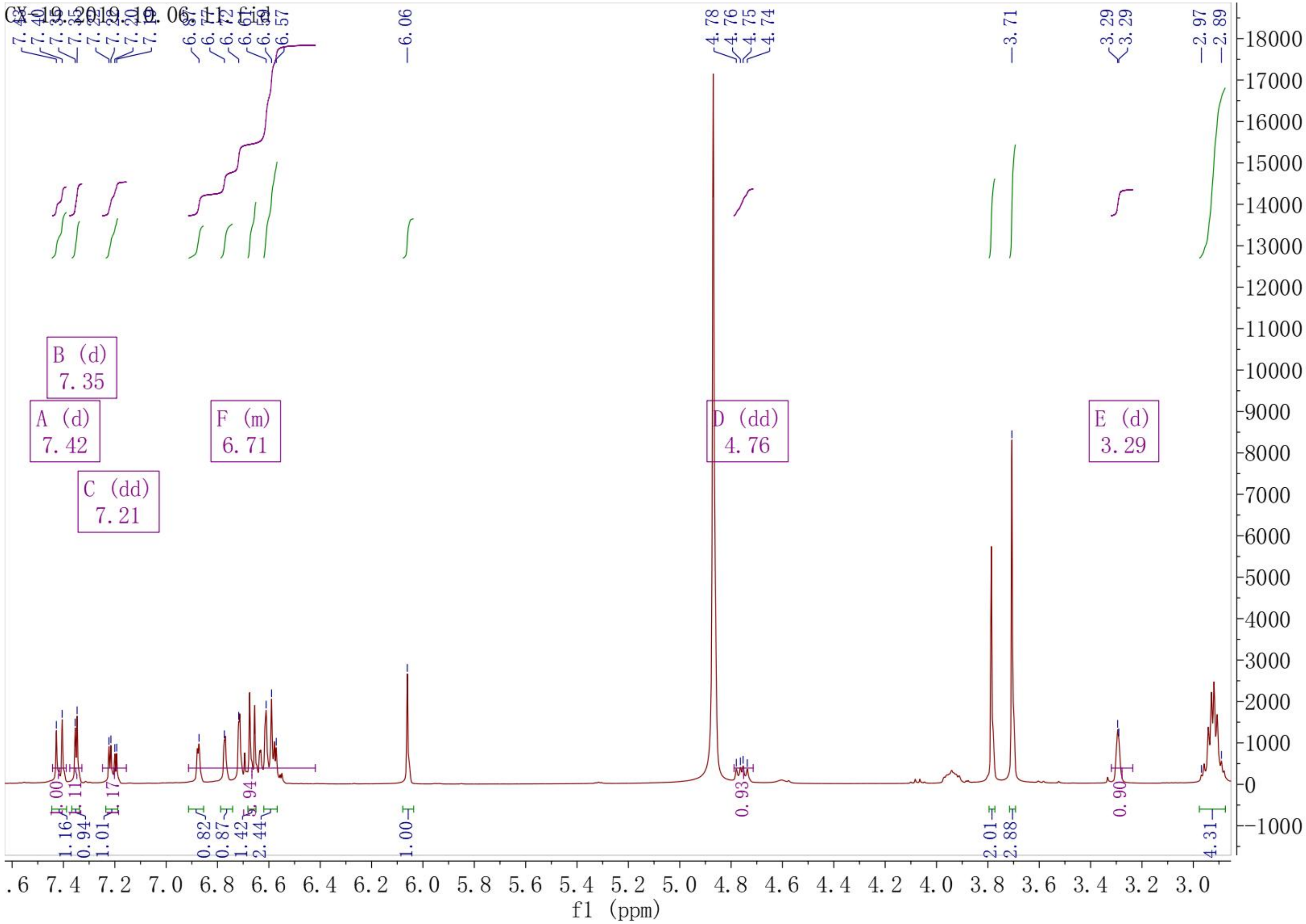

Fig. S 52 ^{1}HNMR of 6-hydroxy-2-(4-hydroxy-3-methoxyphenethyl)-4H-chromen-4-one (400 MHz, CD_3OD)

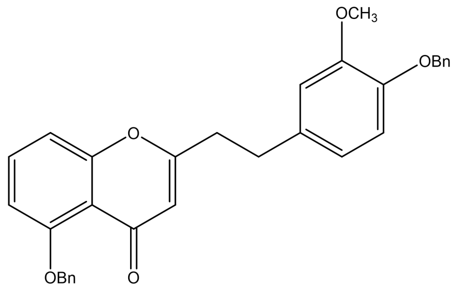

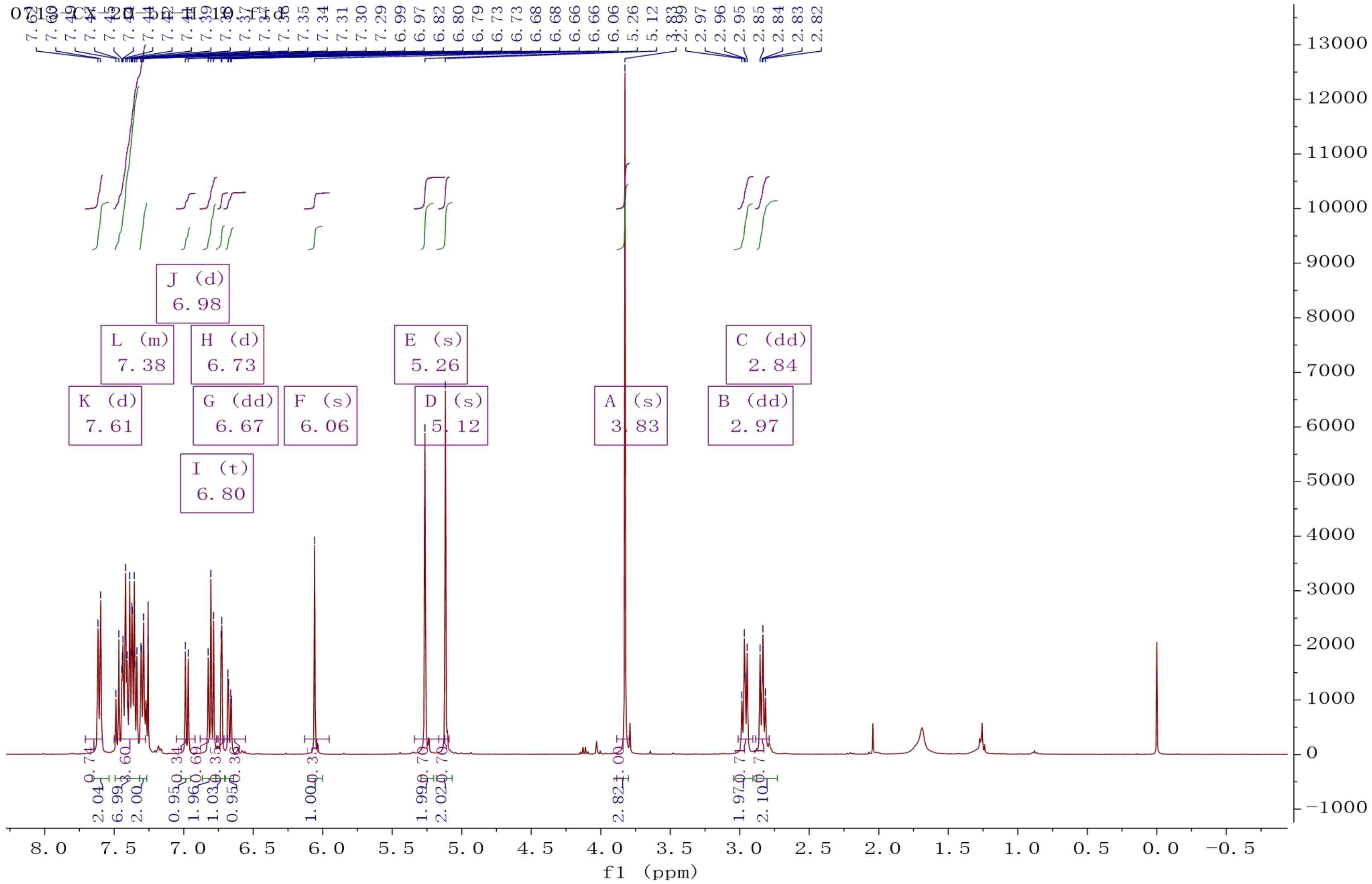

Fig S53 ^{1}HNMR of 5-(benzyloxy)-2-(4-(benzyloxy)-3-methoxyphenethyl)-4H-chromen-4-one (400MHz, $CDCl_3$)

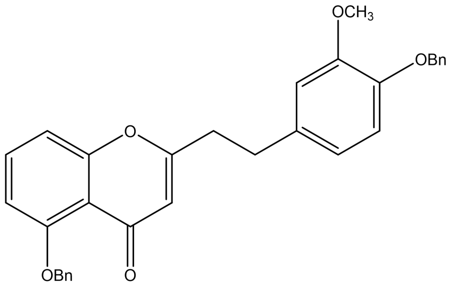

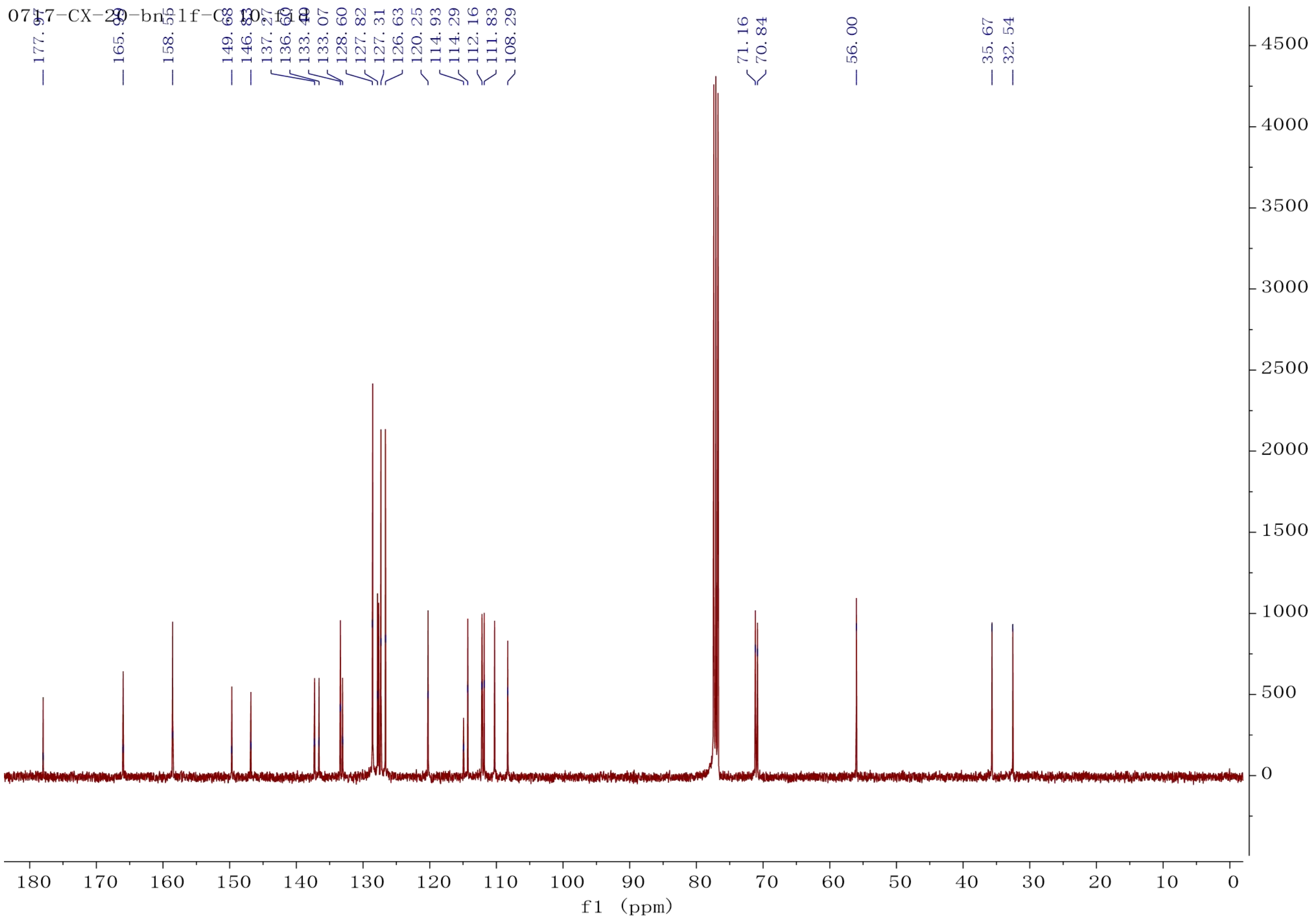

Fig S54 ^{13}CNMR of 5-(benzyloxy)-2-(4-(benzyloxy)-3-methoxyphenethyl)-4H-chromen-4-one (100MHz, $CDCl_3$)

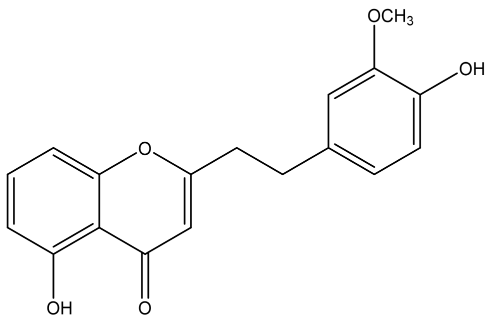

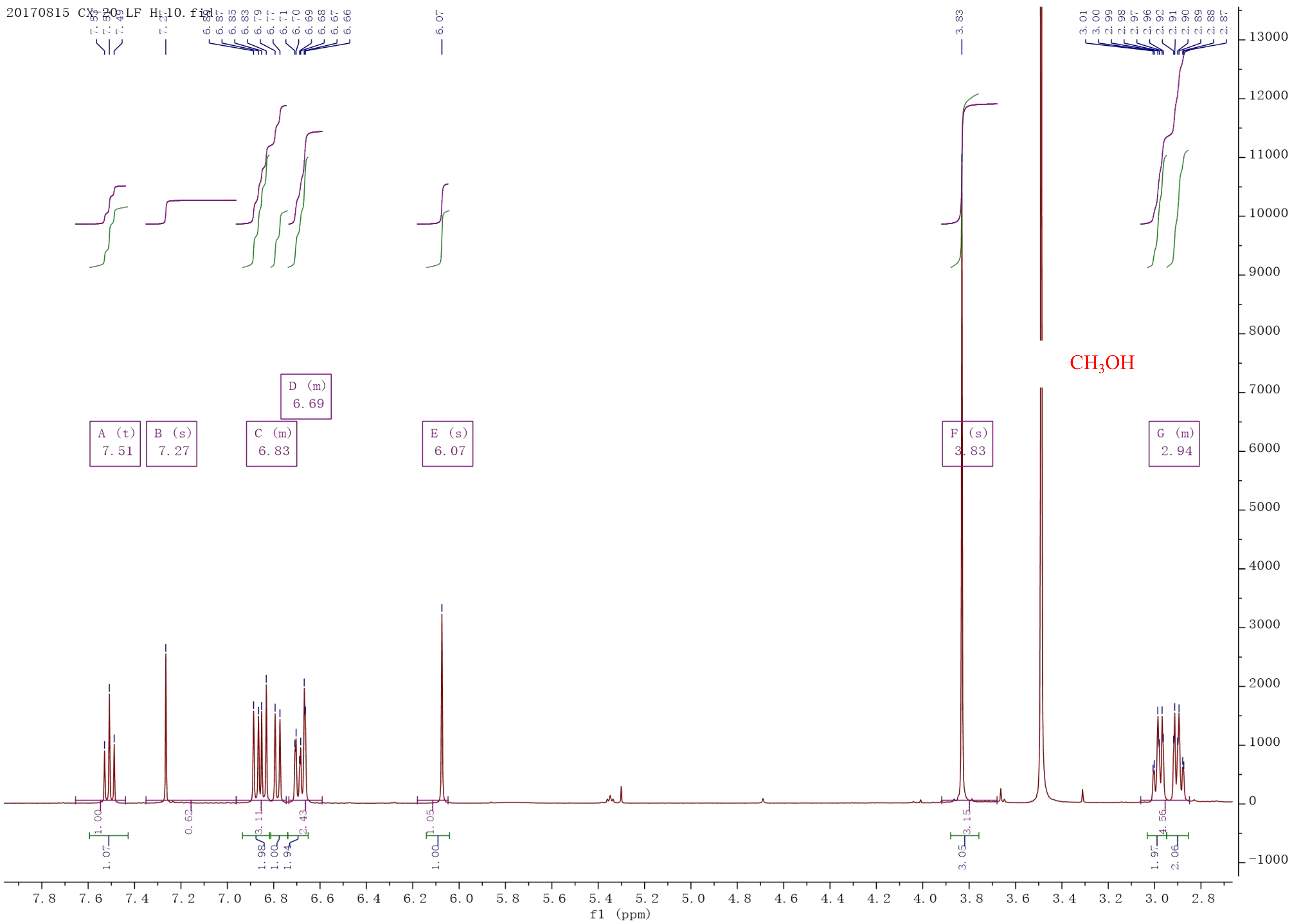

Fig S55 ¹HNMR of 5-hydroxyl-2-(4-hydroxyl-3-methoxyphenethyl)-4H-chromen-4-one (400MHz, $CDCl_3$)

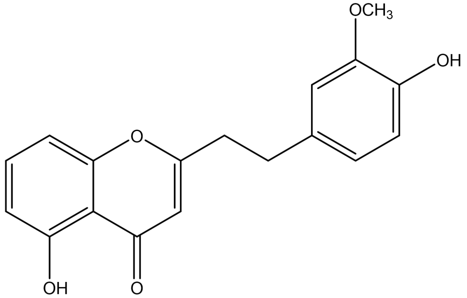

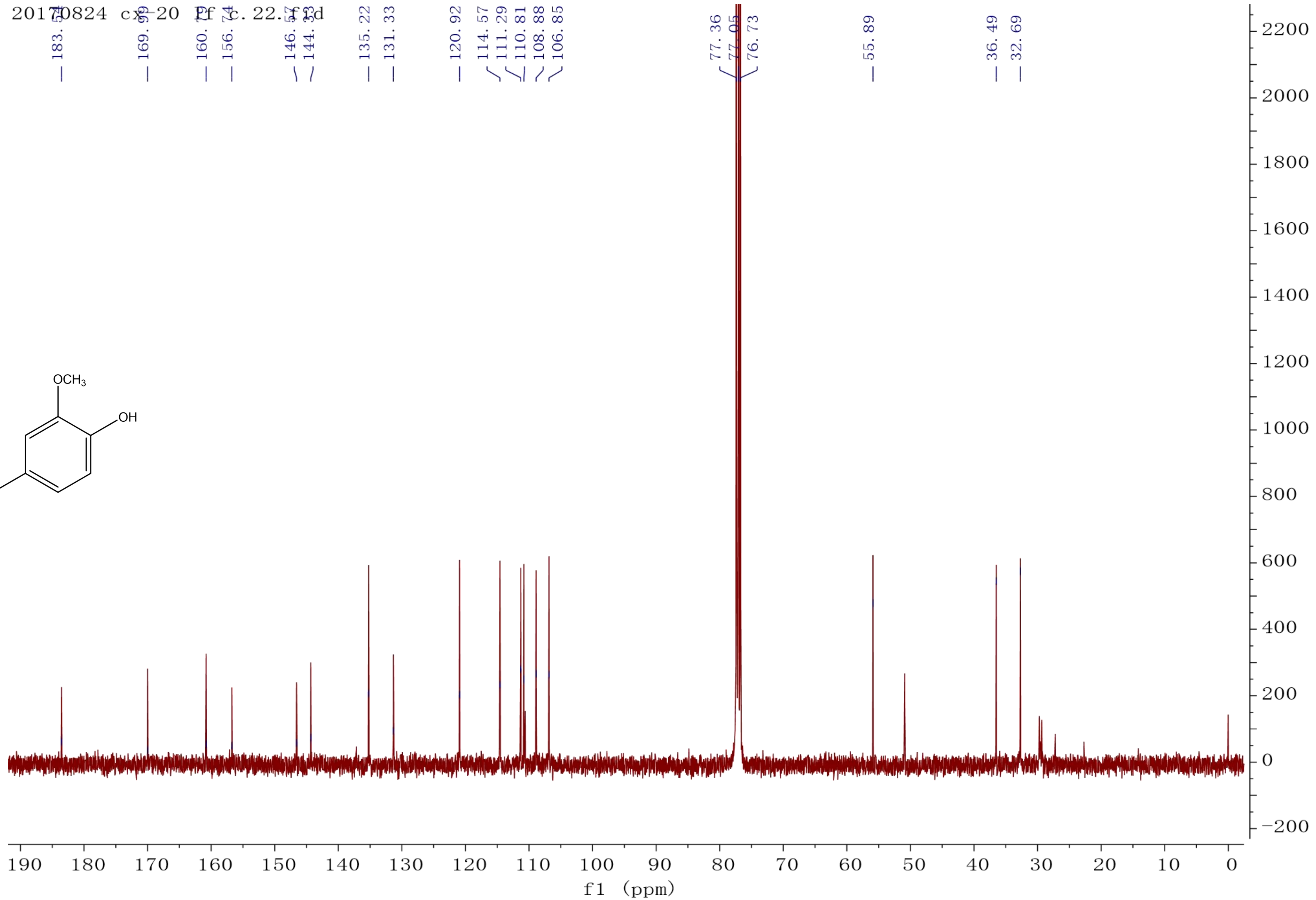

Fig S56 ^{13}CNMR of 5-hydroxyl-2-(4-hydroxyl-3-methoxyphenethyl)-4H-chromen-4-one (100MHz, $CDCl_3$)

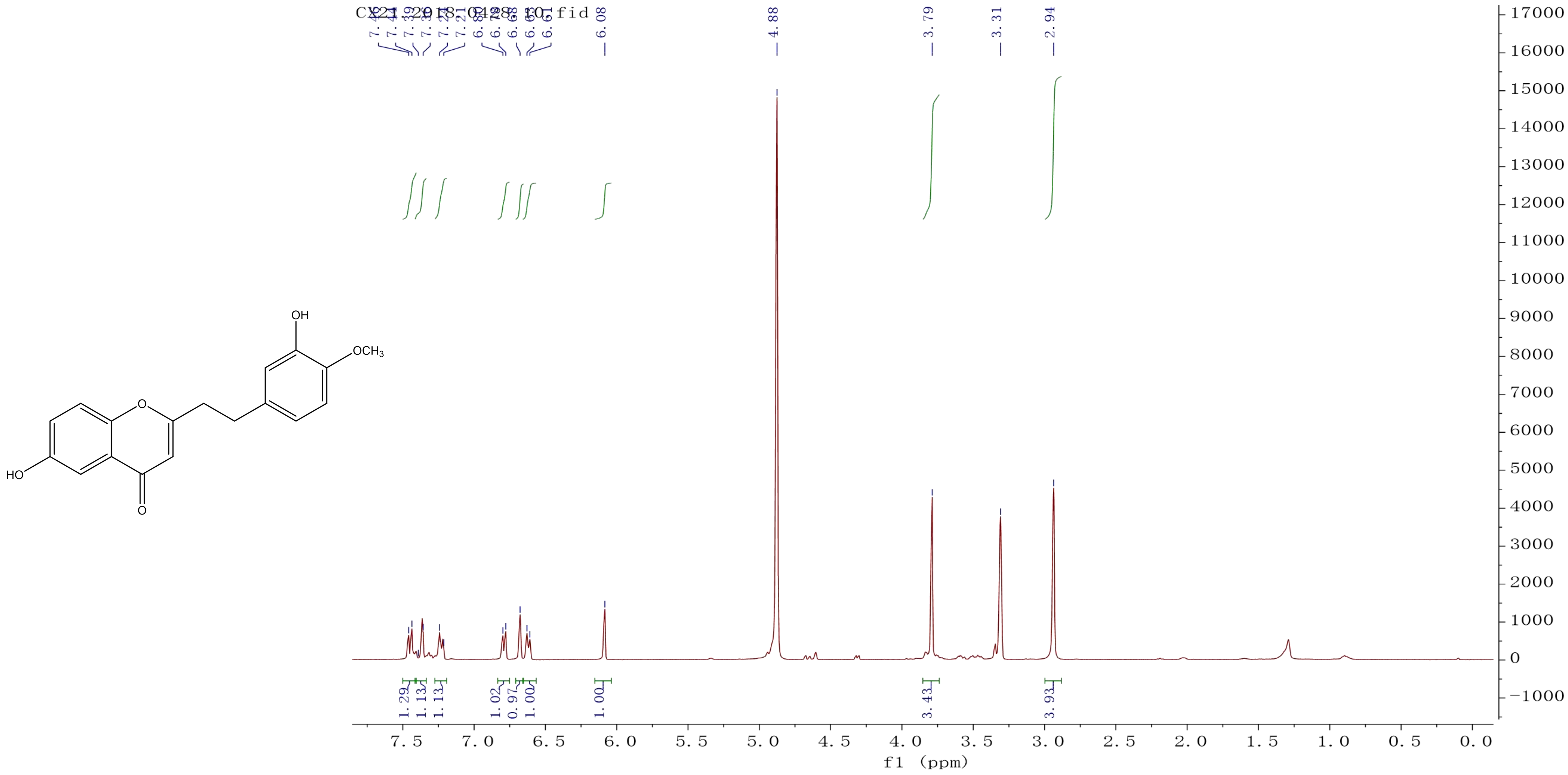

Fig S57 ^{1}HNMR of 6-hydroxyl-2-(3-hydroxyl-4-methoxyphenethyl)-4H-chromen-4-one(400MHz, CD_3OD)

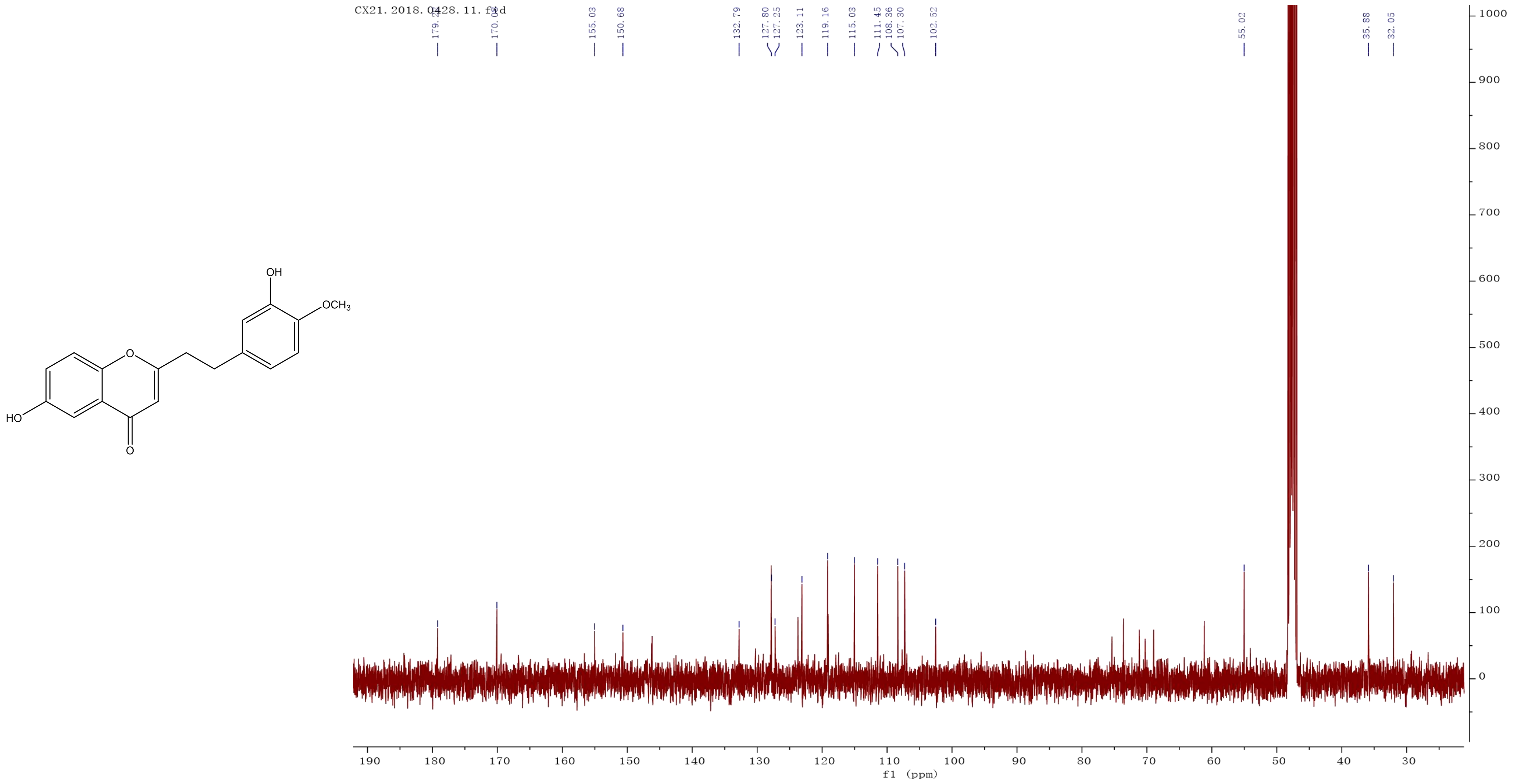

Fig S58 ^{13}CNMR of 6-hydroxyl-2-(3-hydroxyl-4-methoxyphenethyl)-4H-chromen-4-one (100 MHz, CD_3OD)

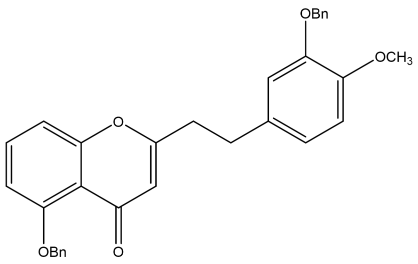

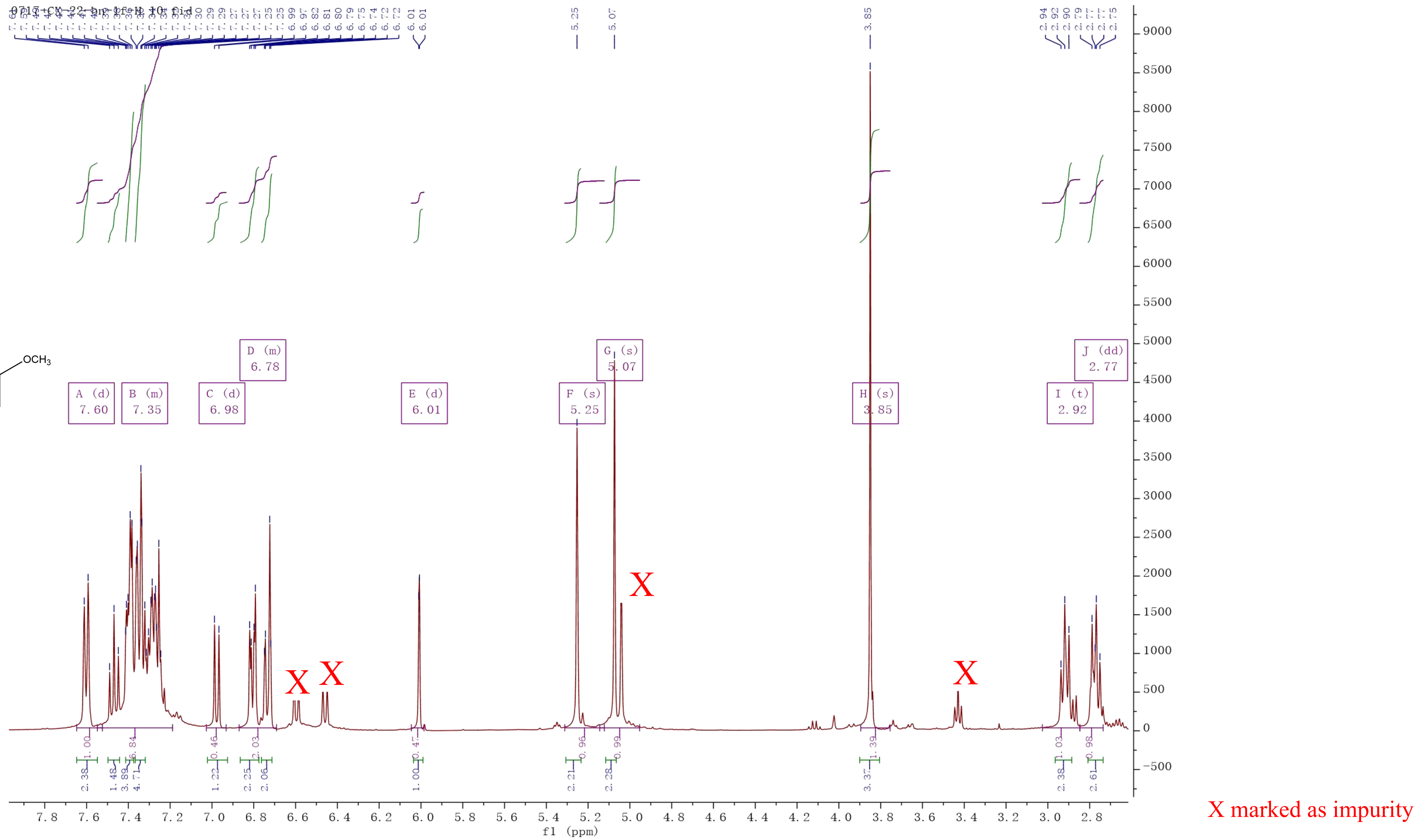

Fig. S59 ^{1}HNMR of 5-(benzyloxy)-2-(3-(benzyloxy)-4-methoxyphenethyl)-4H-chromen-4-one (400 MHz, $CDCl_3$)

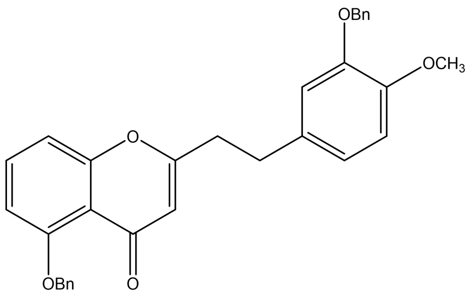

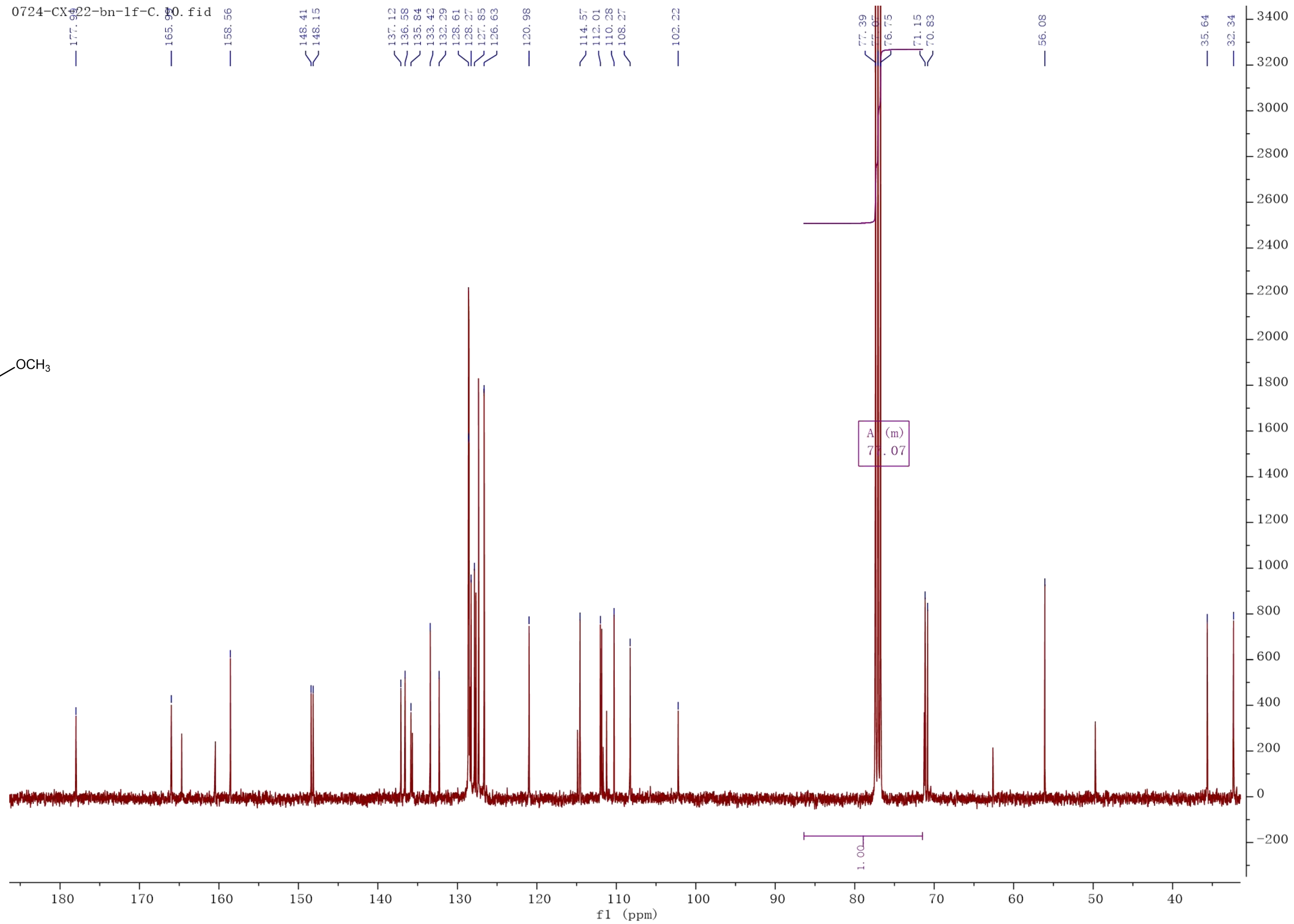

Fig. S60 ^{13}CNMR of 5-(benzyloxy)-2-(3-(benzyloxy)-4-methoxyphenethyl)-4H-chromen-4-one (100 MHz, $CDCl_3$)

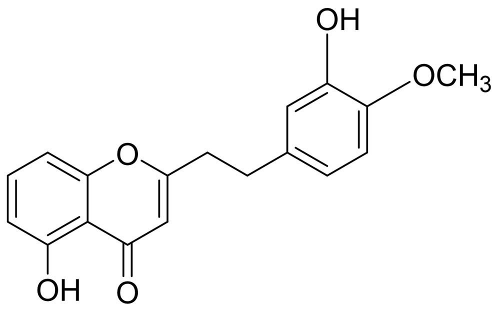

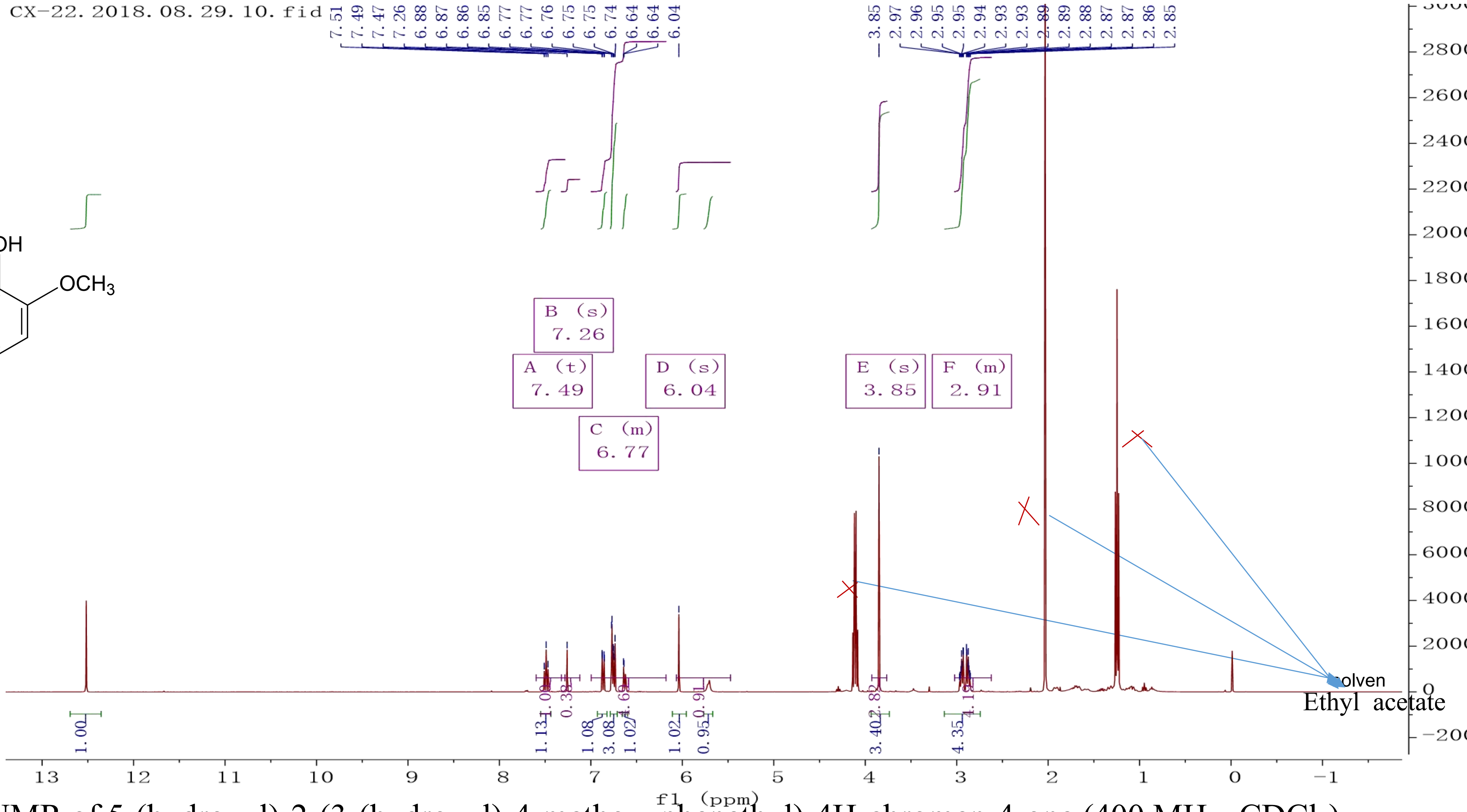

Fig. S61 ^{1}HNMR of 5-(hydroxyl)-2-(3-(hydroxyl)-4-methoxyphenethyl)-4H-chromen-4-one (400 MHz, $CDCl_3$)

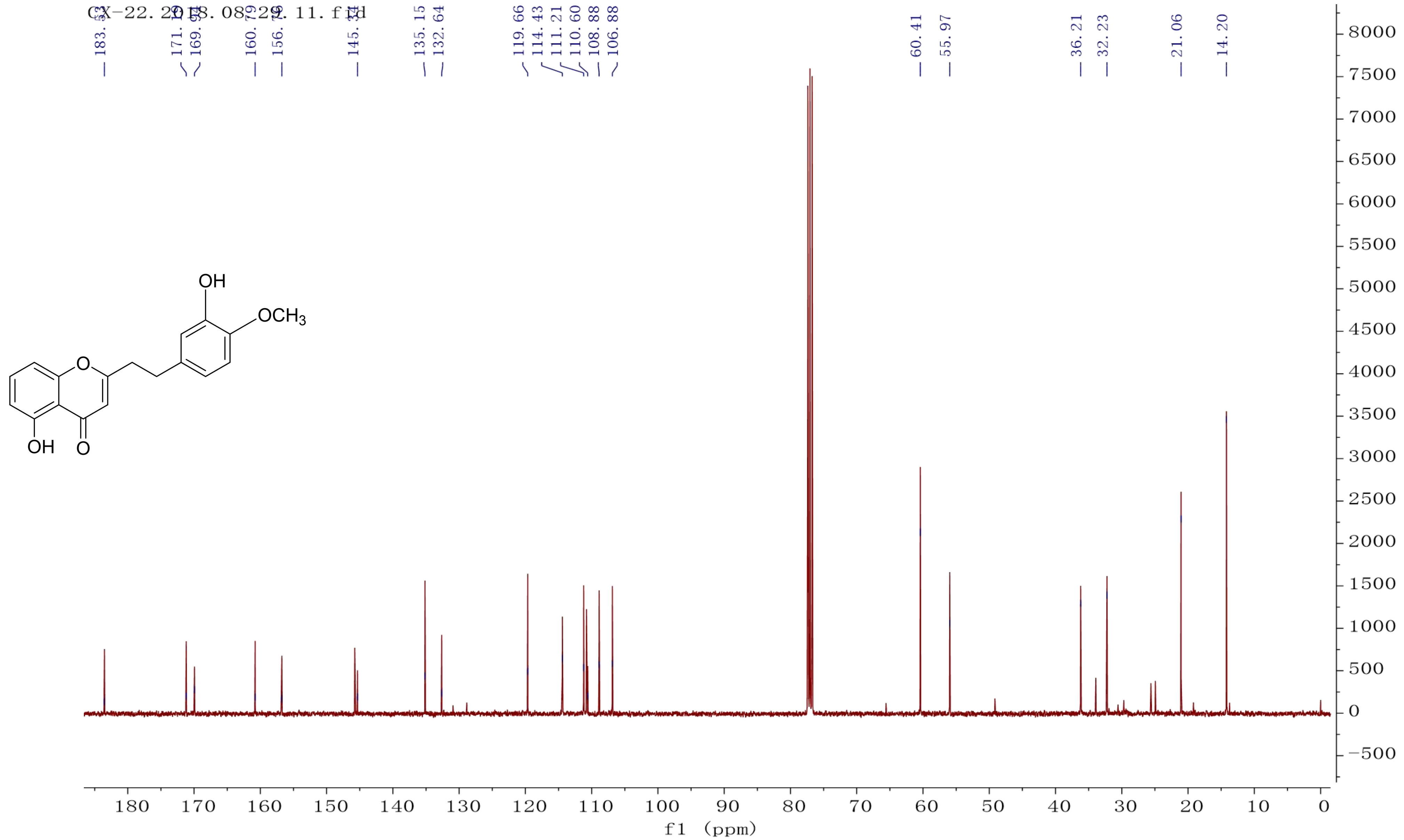

Fig. S62 ^{13}C NMR of 5-(hydroxyl)-2-(3-(hydroxyl)-4-methoxyphenethyl)-4H-chromen-4-one(100 MHz, $CDCl_3$)

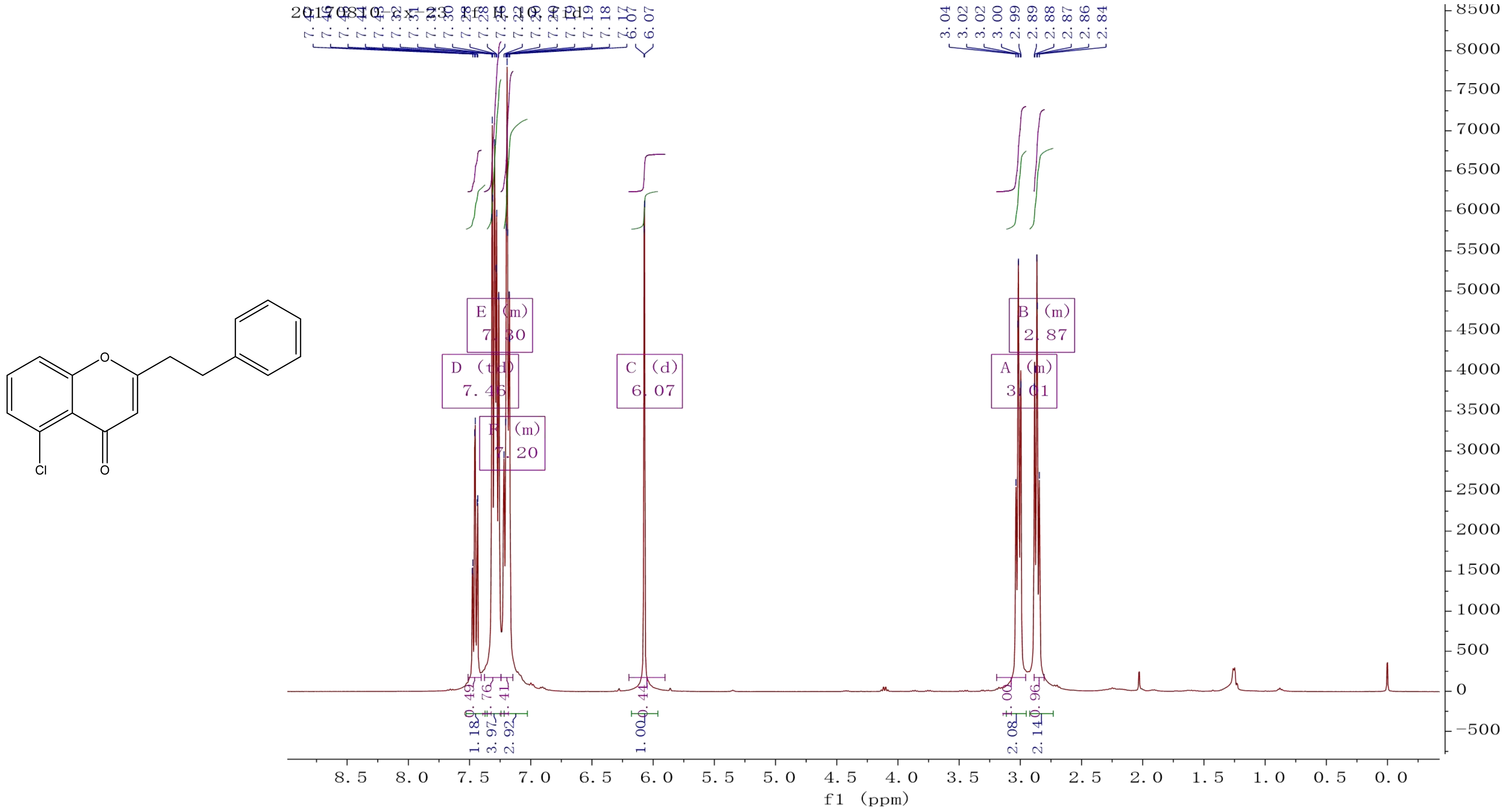

Fig S63 ^{1}HNMR of 5-chloro-2-phenethyl-4H-chromen-4-one (400MHz, $CDCl_3$)

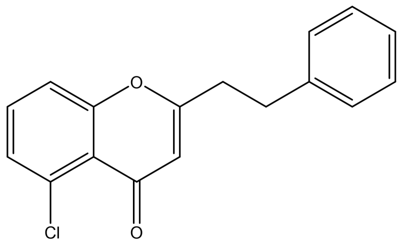

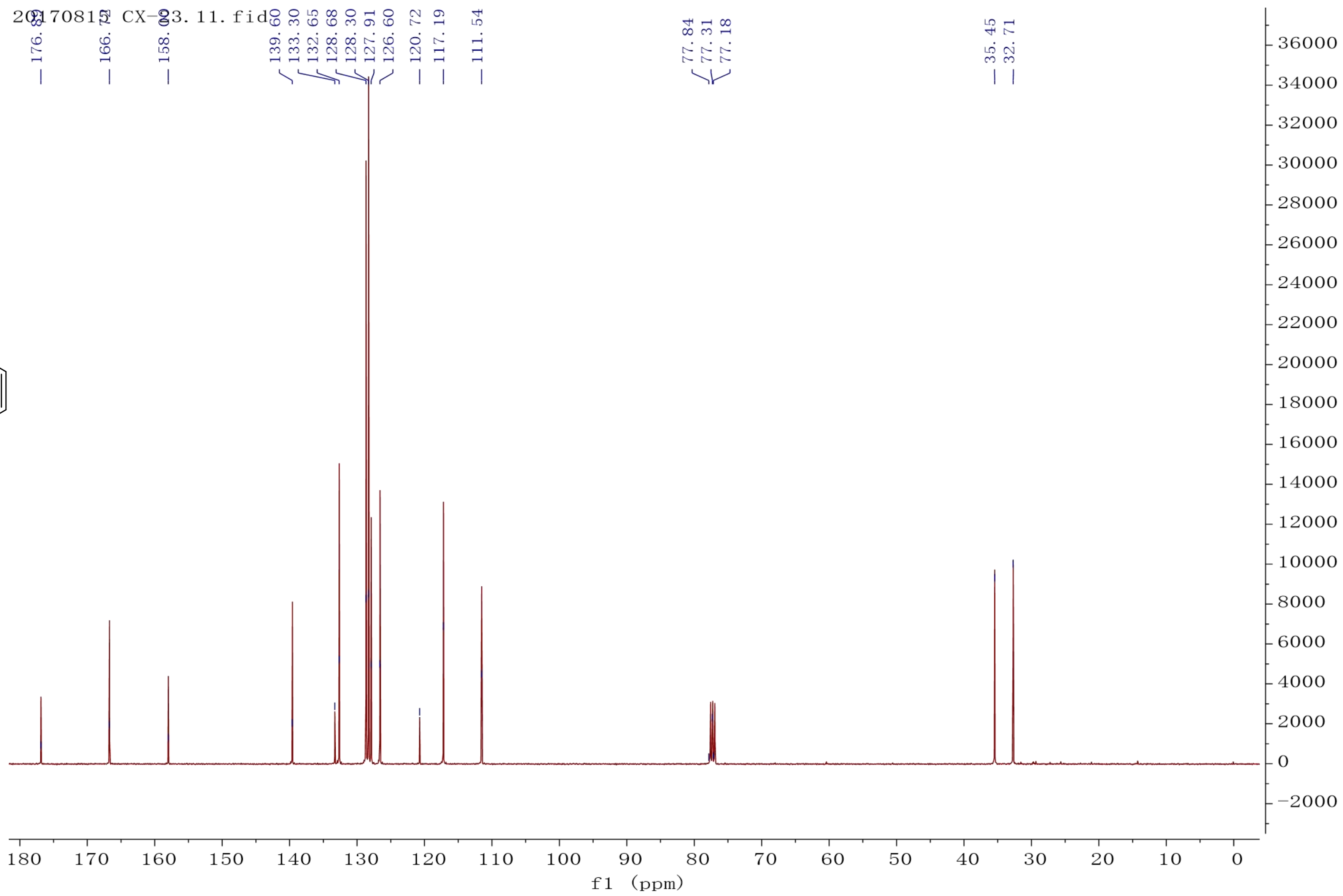

Fig. S64 ^{13}CNMR of 5-chloro-2-phenethyl-4H-chromen-4-one (100 MHz, $CDCl_3$)

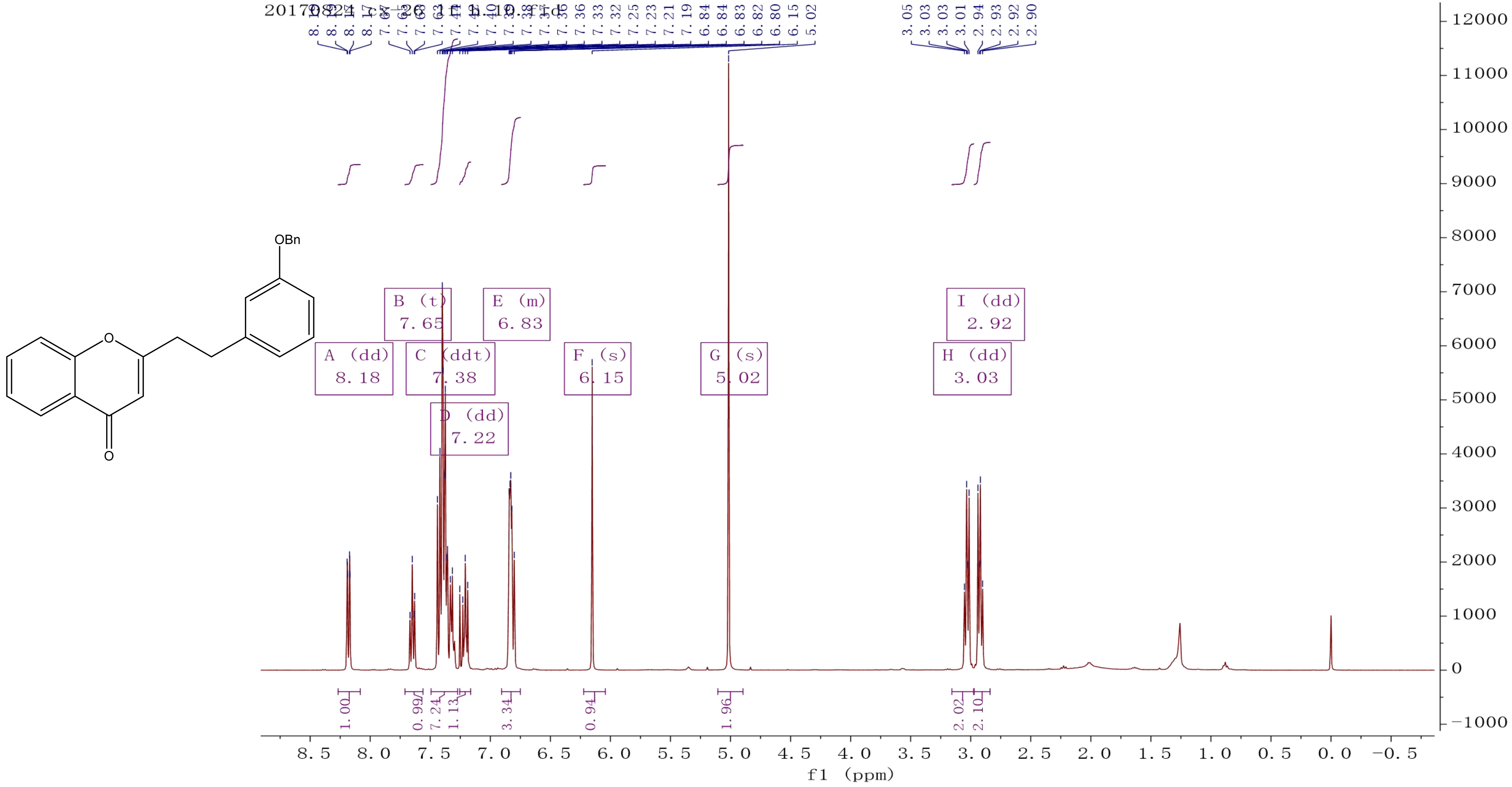

Fig. S65 ^{1}HNMR of 2-(3-(benzyloxy)phenethyl)-4H-chromen-4-one (400 MHz, $CDCl_3$)

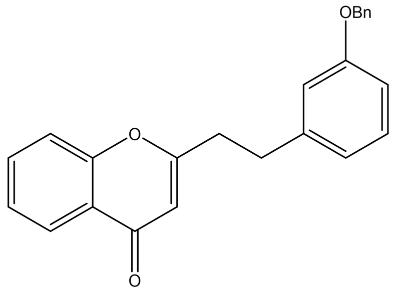

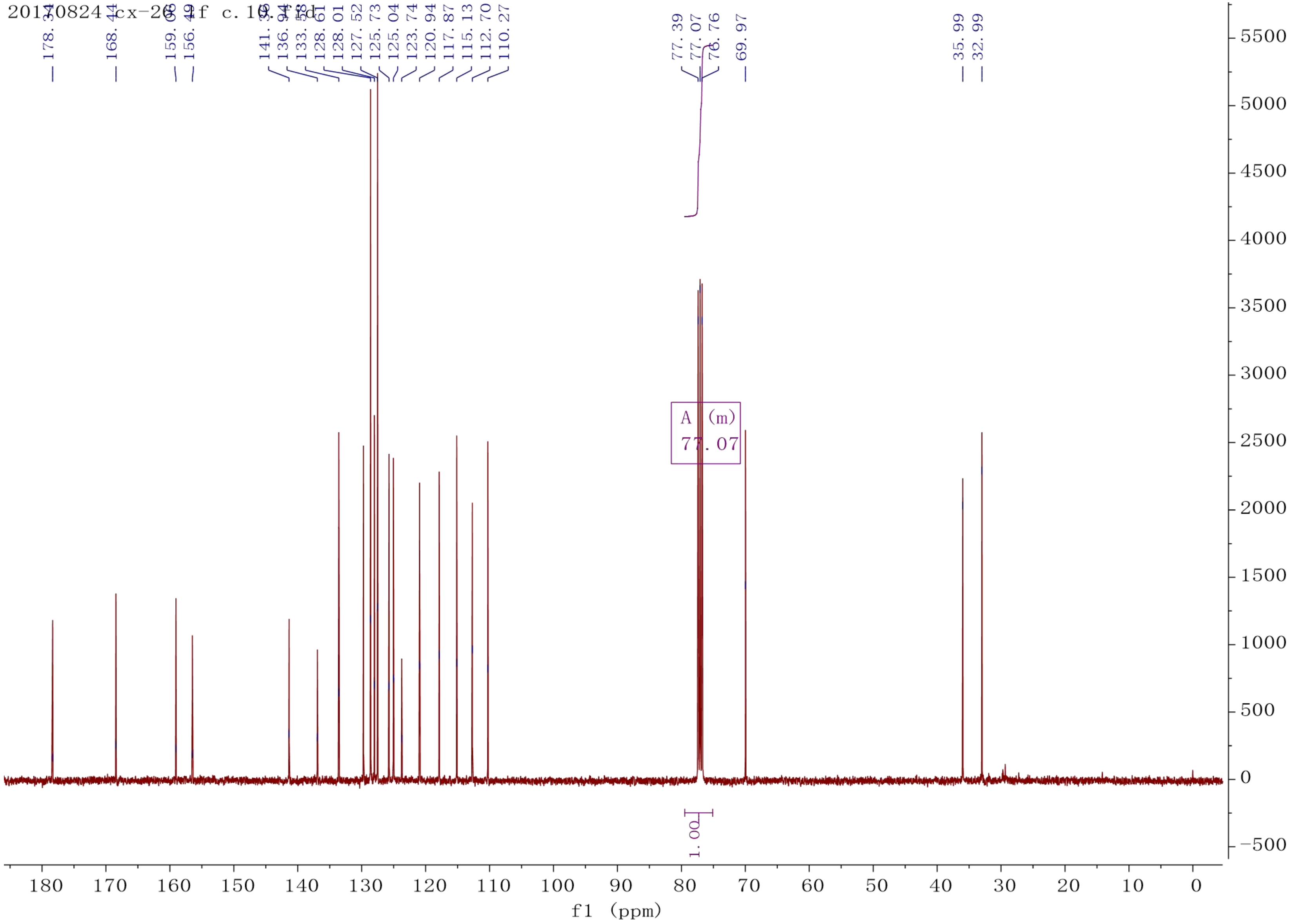

Fig S66 ^{13}CNMR of 2-(3-(benzyloxy)phenethyl)-4H-chromen-4-one (100 MHz, $CDCl_3$)

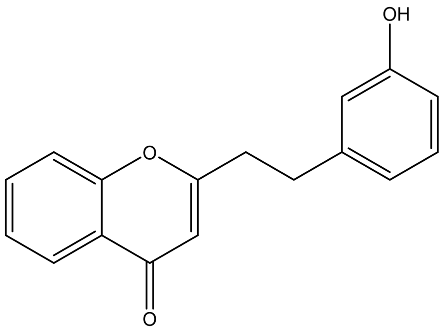

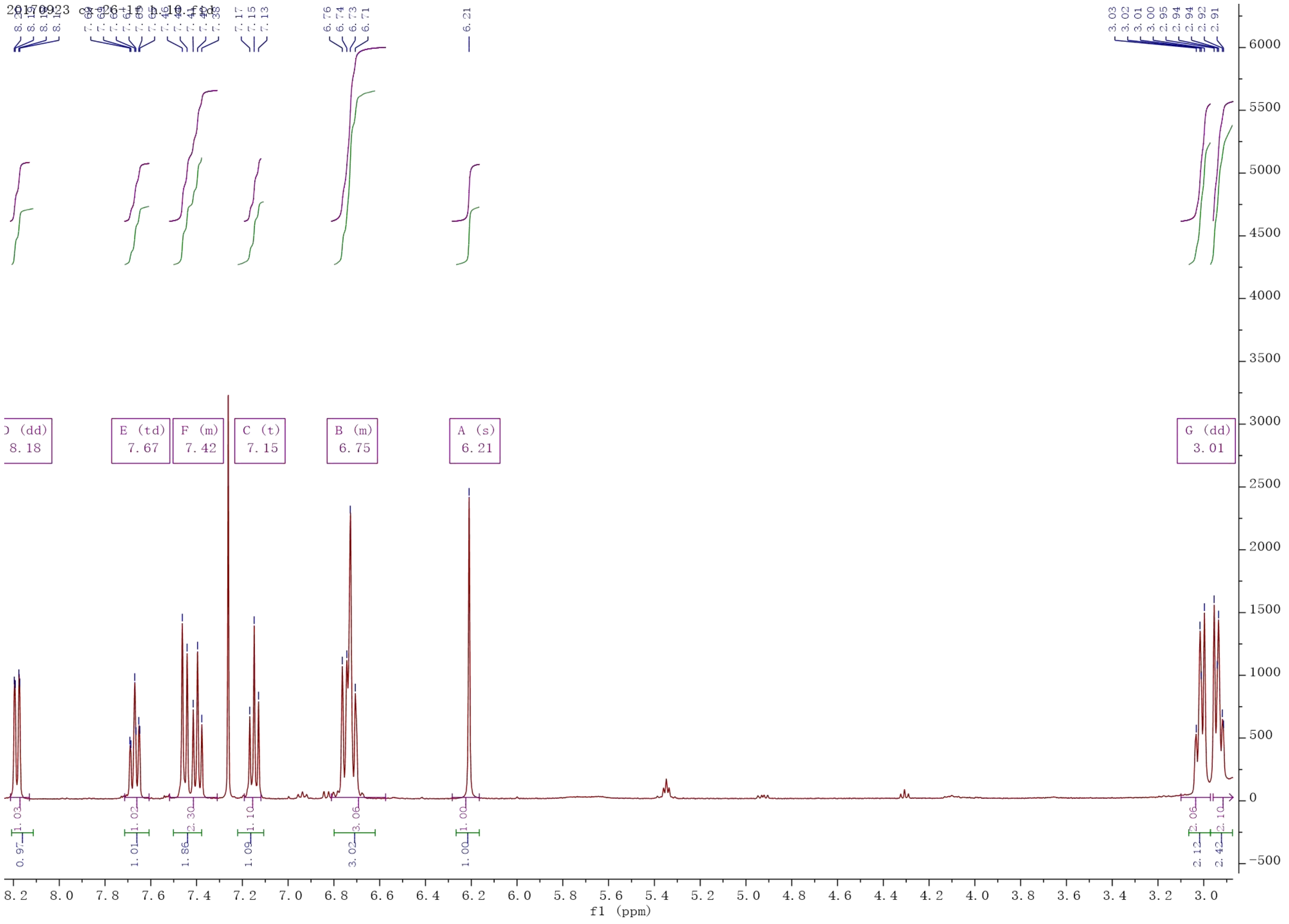

Fig S67 ^{1}HNMR of 2-(3-(hydroxy)phenethyl)-4H-chromen-4-one (400 MHz, $CDCl_3$)

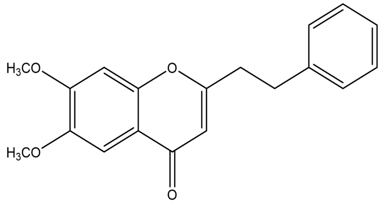

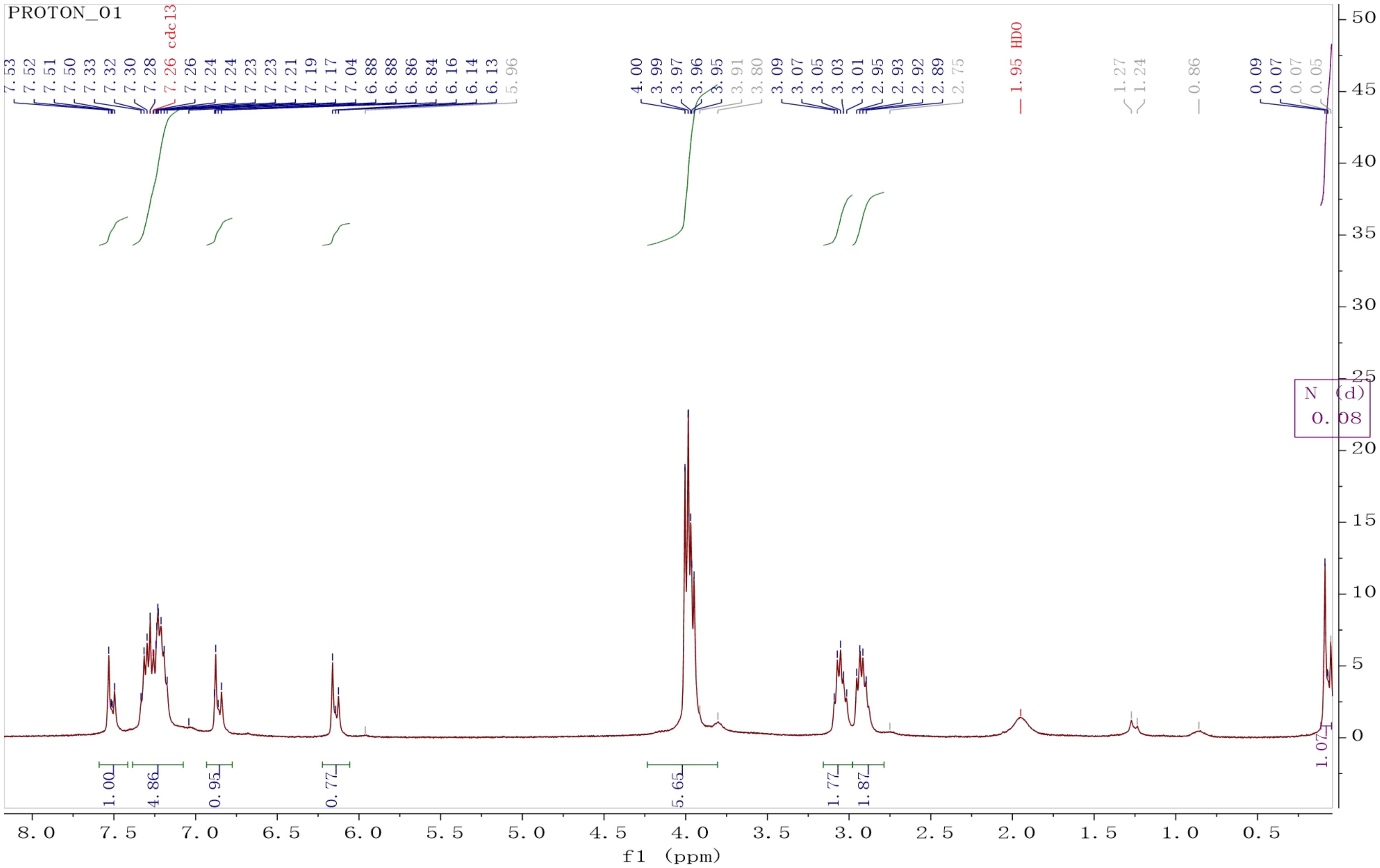

Fig S68 ^{1}HNMR 6,7-dimethoxy-2-phenethyl-4H-chromen-4-one (400 MHz, $CDCl_3$)

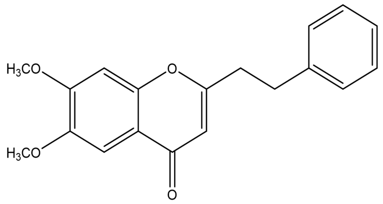

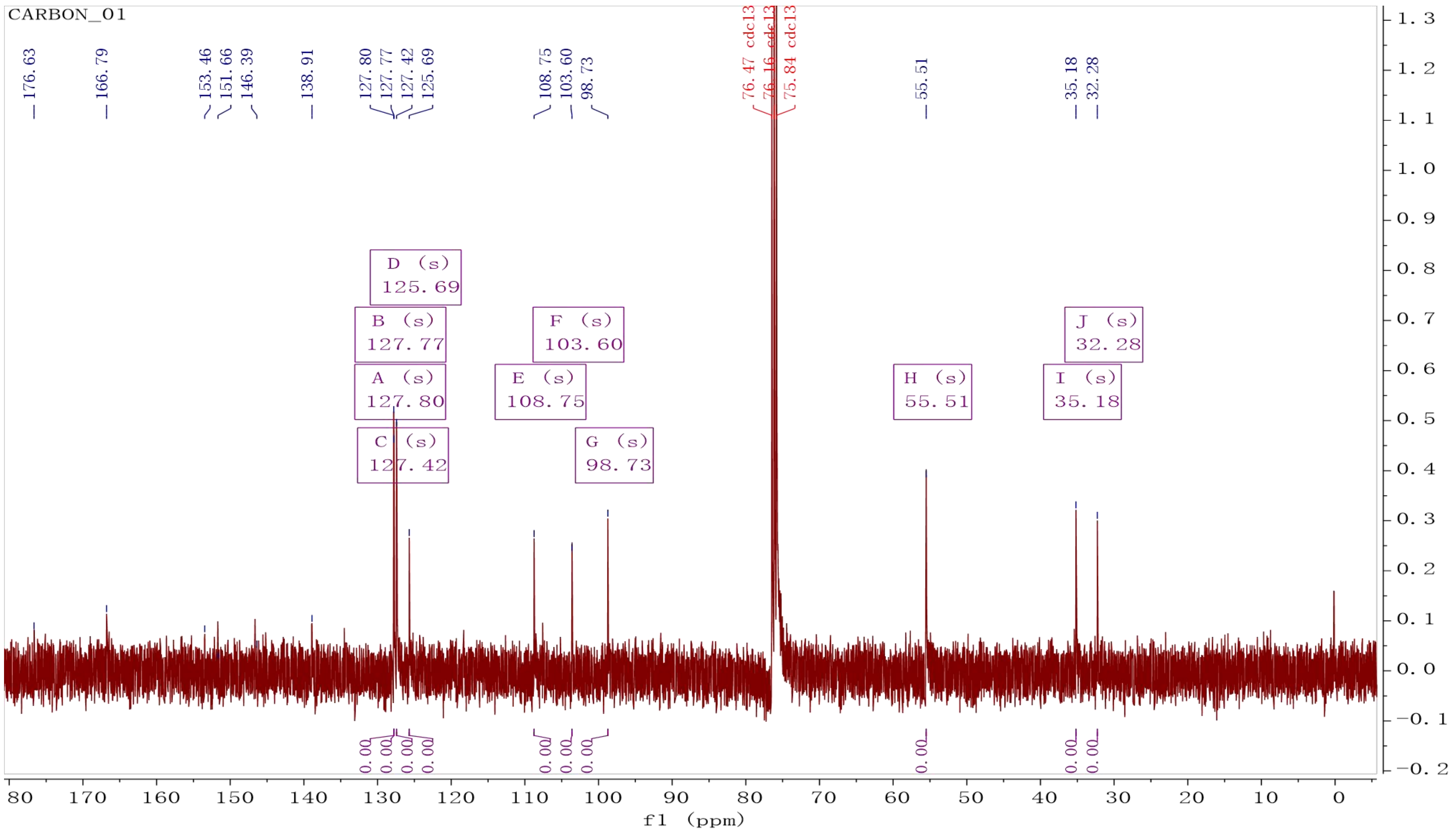

Fig S69 ^{13}C NMR 6,7-dimethoxy-2-phenethyl-4H-chromen-4-one (100 MHz, $CDCl_3$)

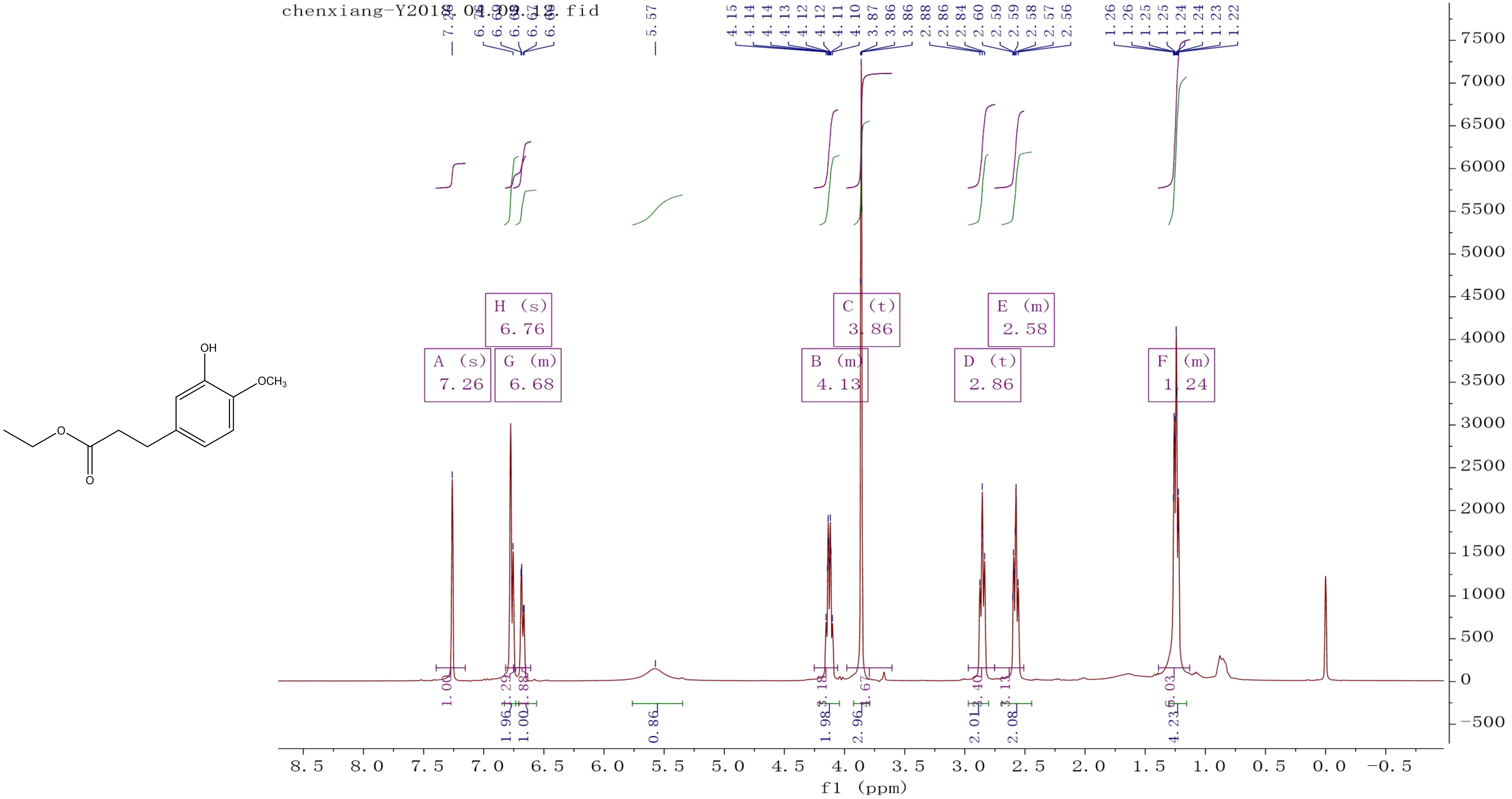

Fig. S 70 ^{1}HNMR of ethyl 3-(3-hydroxy-4-methoxyphenyl)propanoate (400MHz, $CDCl_3$)

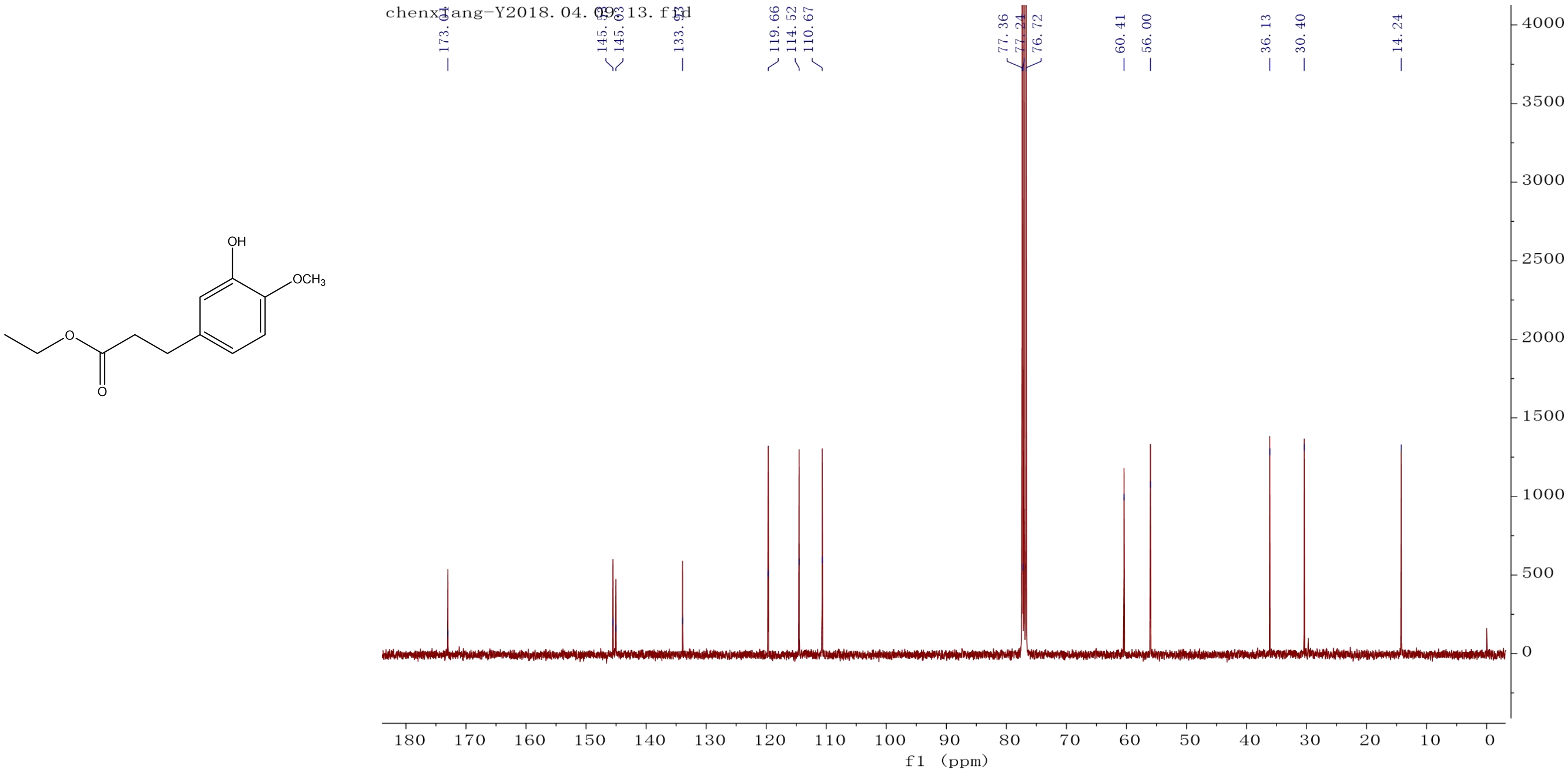

Fig. S71 ^{13}CNMR of ethyl 3-(3-hydroxy-4-methoxyphenyl)propanoate (100 MHz, $CDCl_3$)

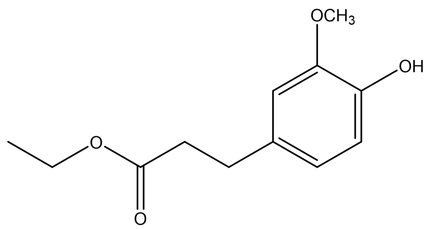

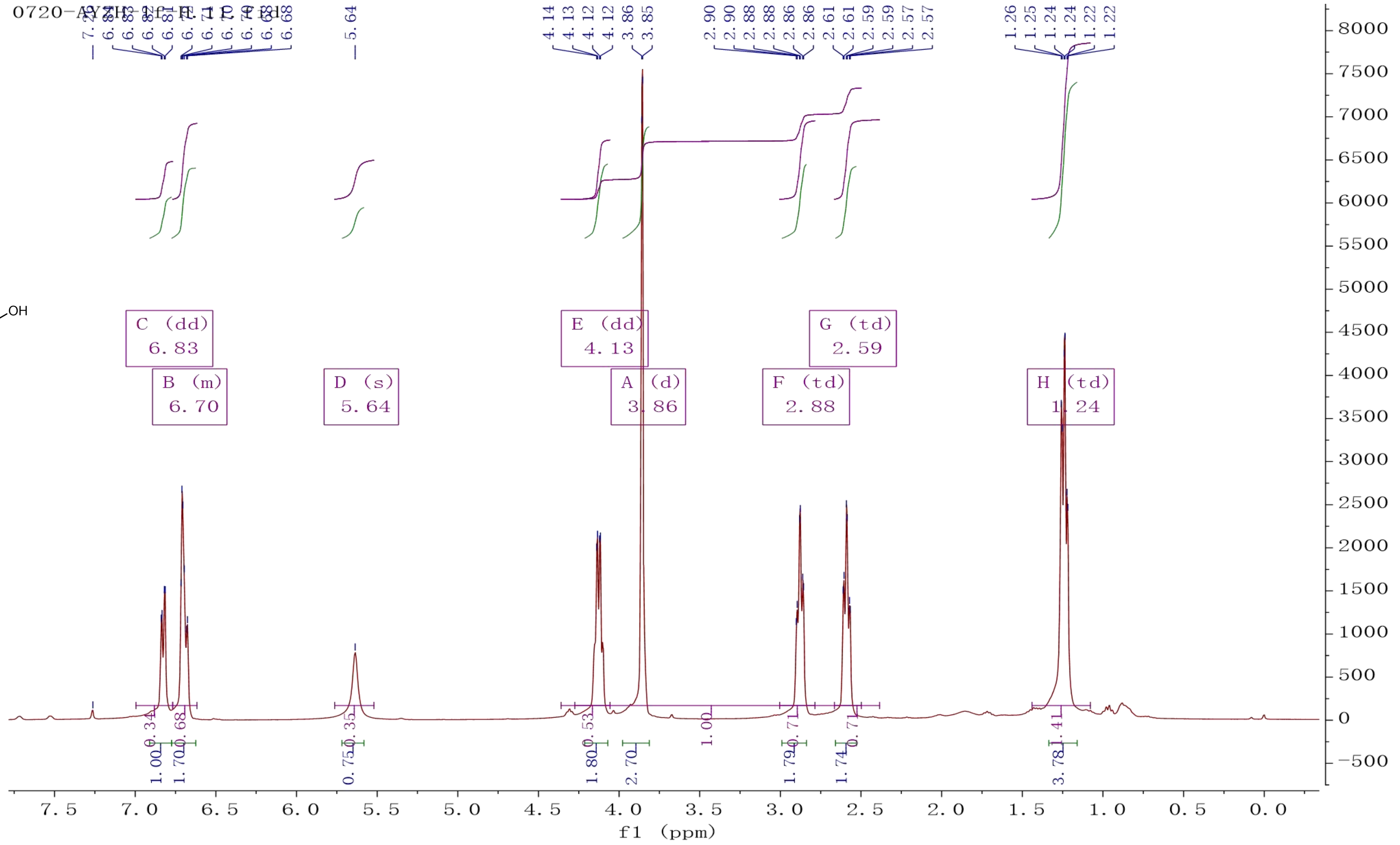

Fig. S 72 ^{1}HNMR of ethyl 3-(4-hydroxy-3-methoxyphenyl)propanoate (400 MHz, $CDCl_3$)

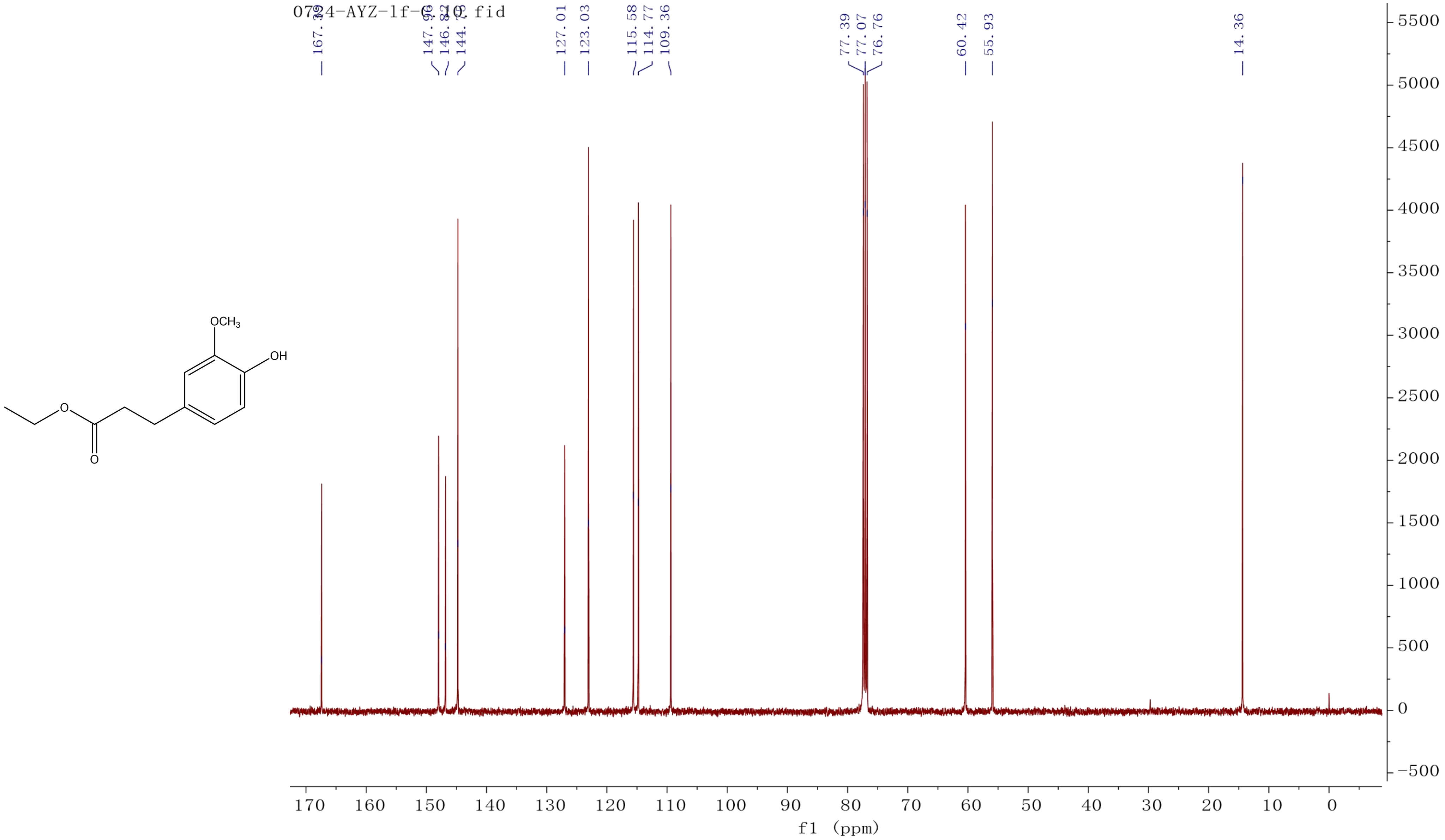

Fig. S 73 ^{13}C NMR of ethyl 3-(4-hydroxy-3-methoxyphenyl)propanoate (100 MHz, $CDCl_3$)

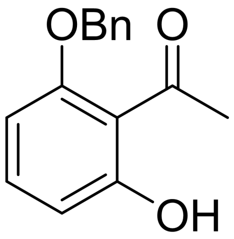

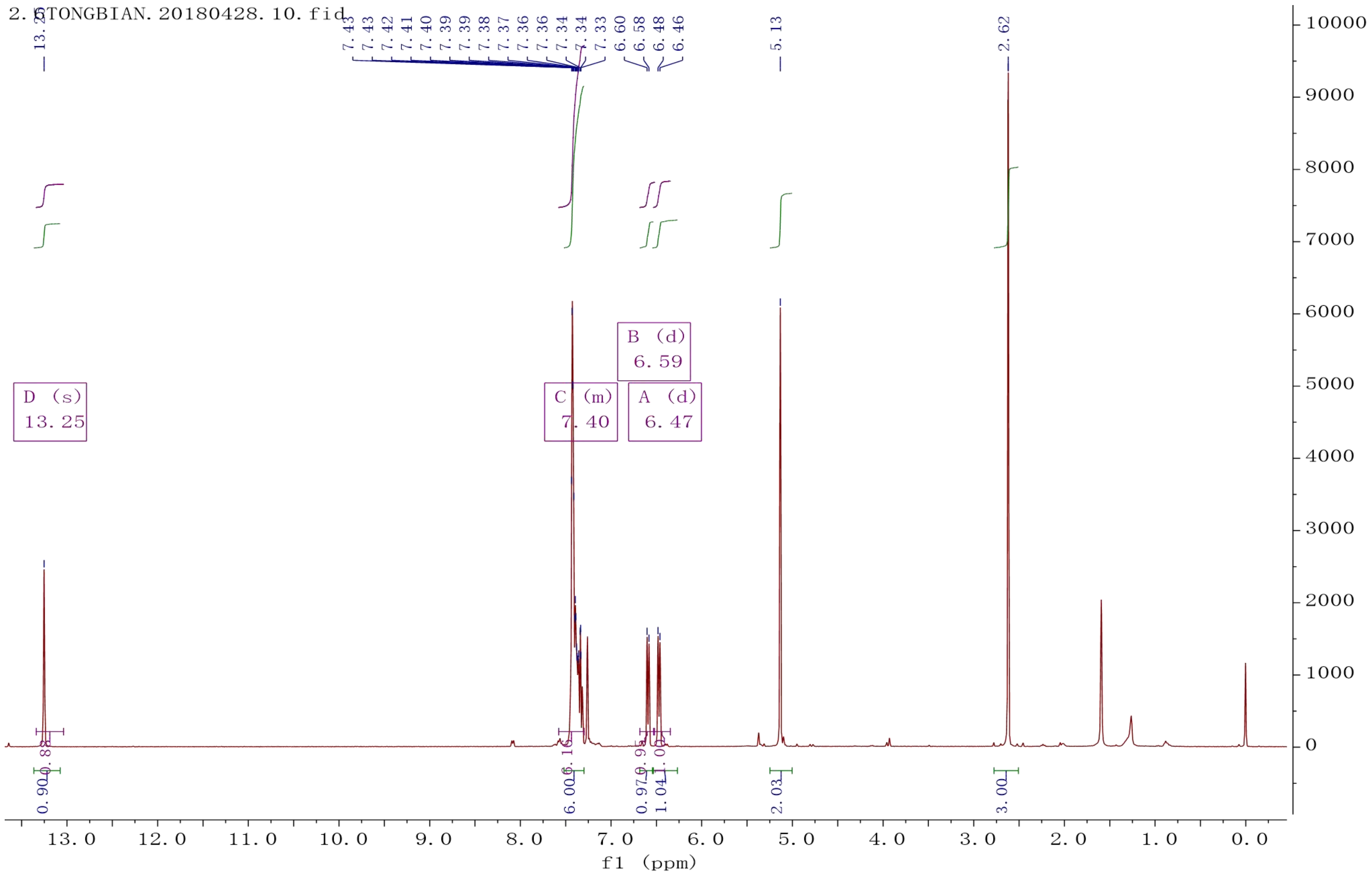

Fig S 74 ^{1}HNMR of 1-(2-(benzyloxy)-6-hydroxyphenyl)ethan-1-one (400MHz, $CDCl_3$)

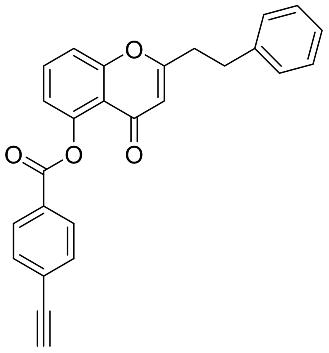

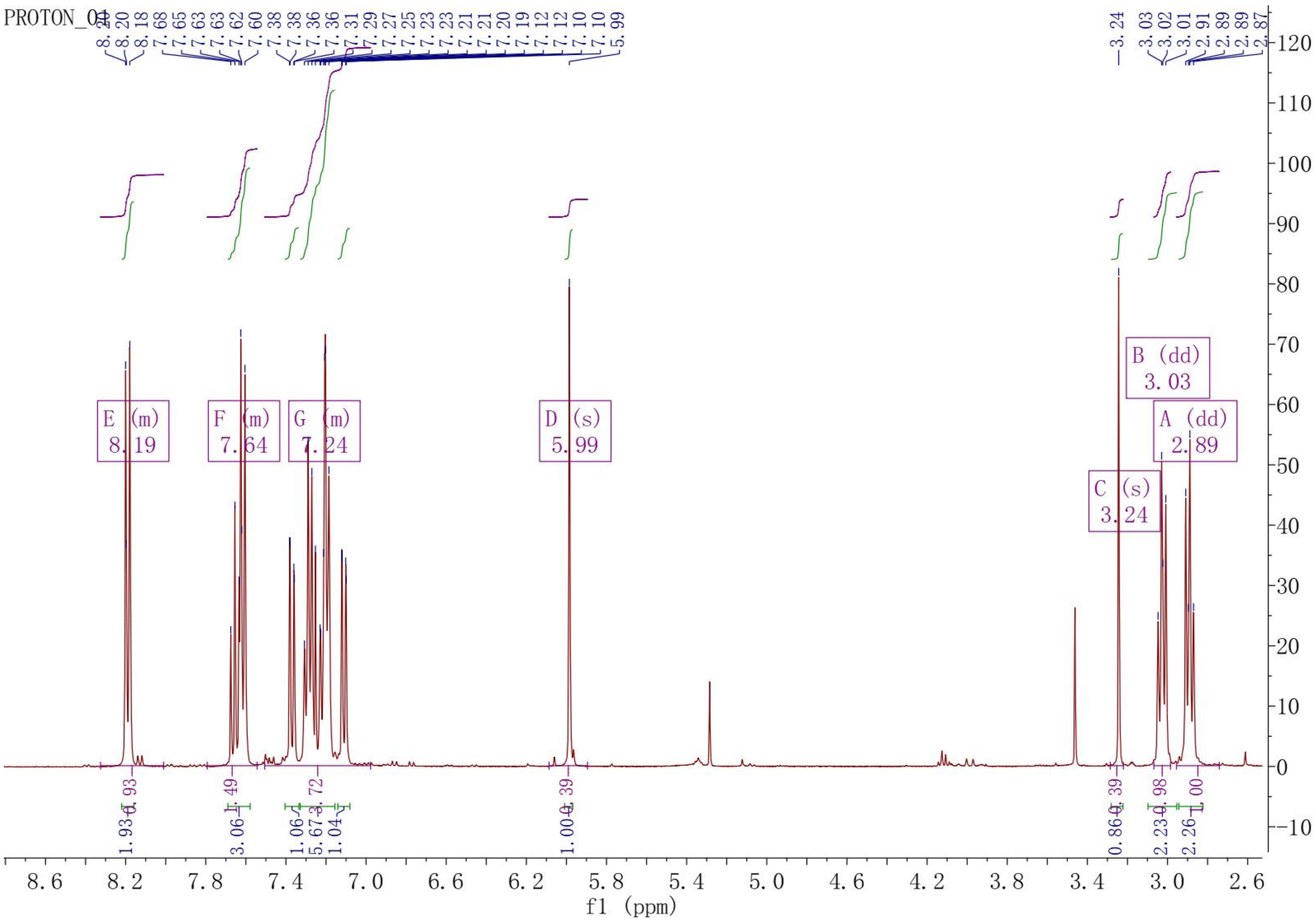

Fig S 75 ^{1}HNMR of 4-oxo-2-phenethyl-4H-chromen-5-yl 4-ethynylbenzoate (400 MHz, $CDCl_3$)

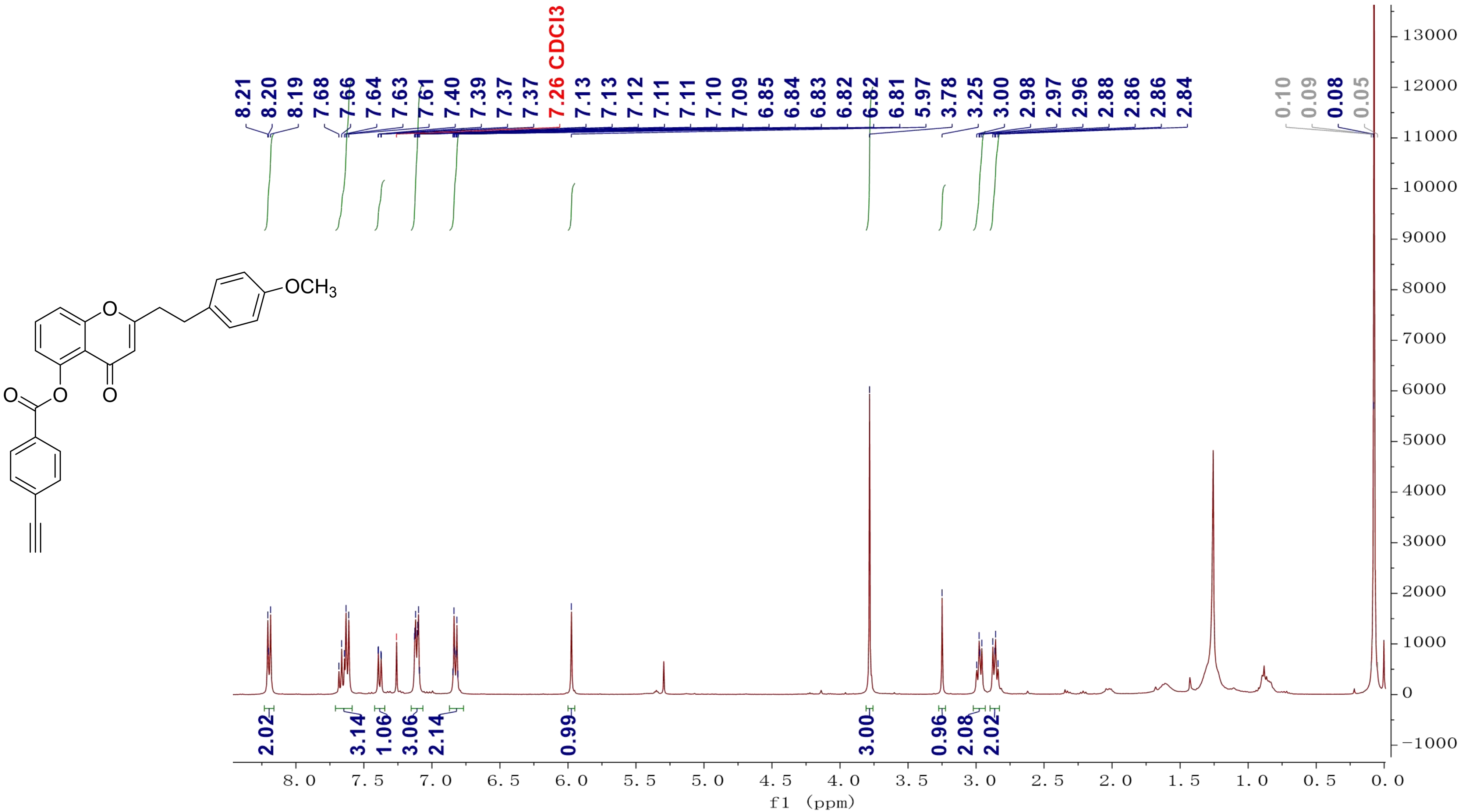

Fig. S 76 ^{1}H NMR of 2-(4-methoxyphenethyl)-4-oxo-4H-chromen-5-yl 4-ethynylbenzoate (400MHz, $CDCl_3$)

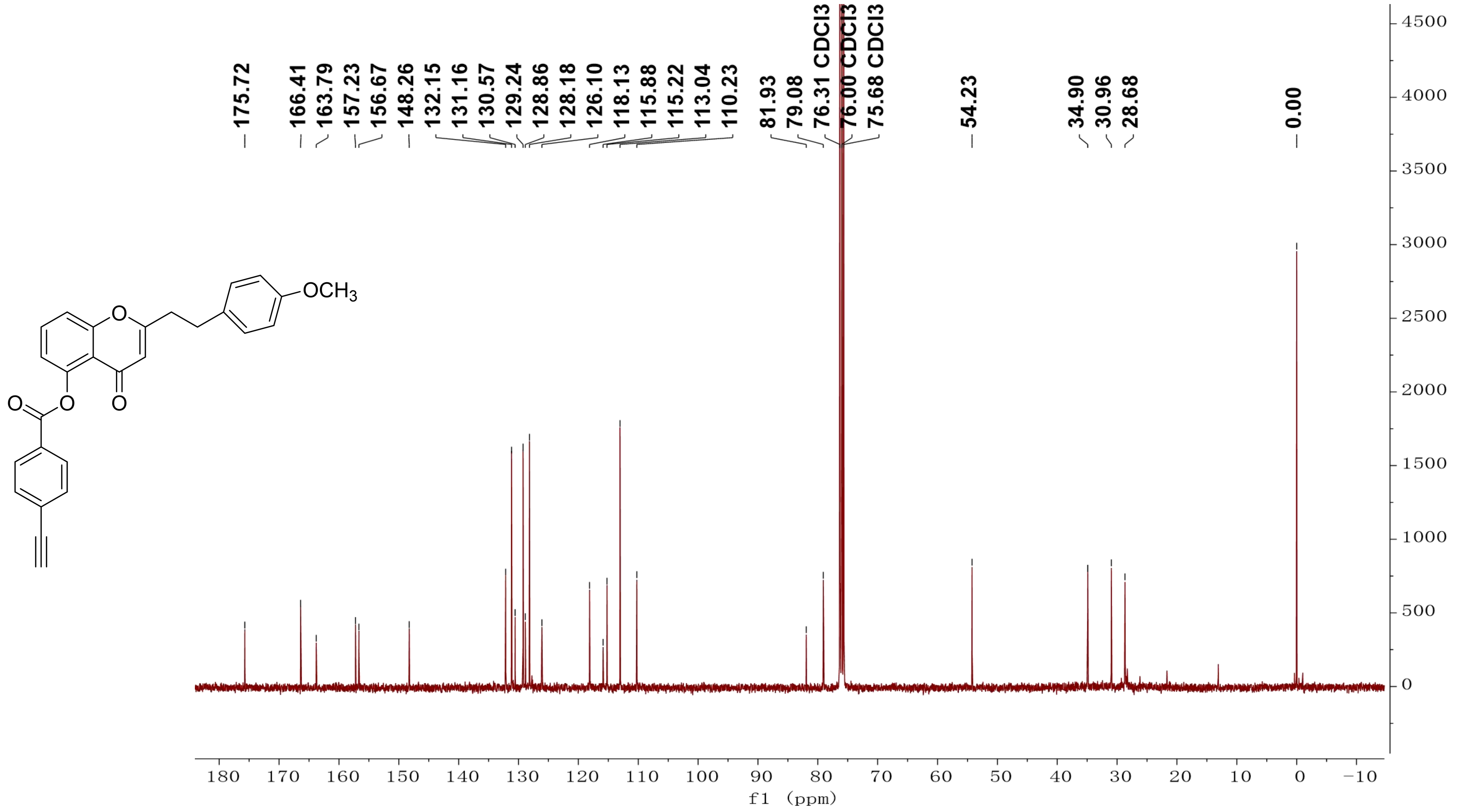

Fig S 77 ^{13}C NMR of 2-(4-methoxyphenethyl)-4-oxo-4H-chromen-5-yl 4-ethynylbenzoate (100MHz, $CDCl_3$)

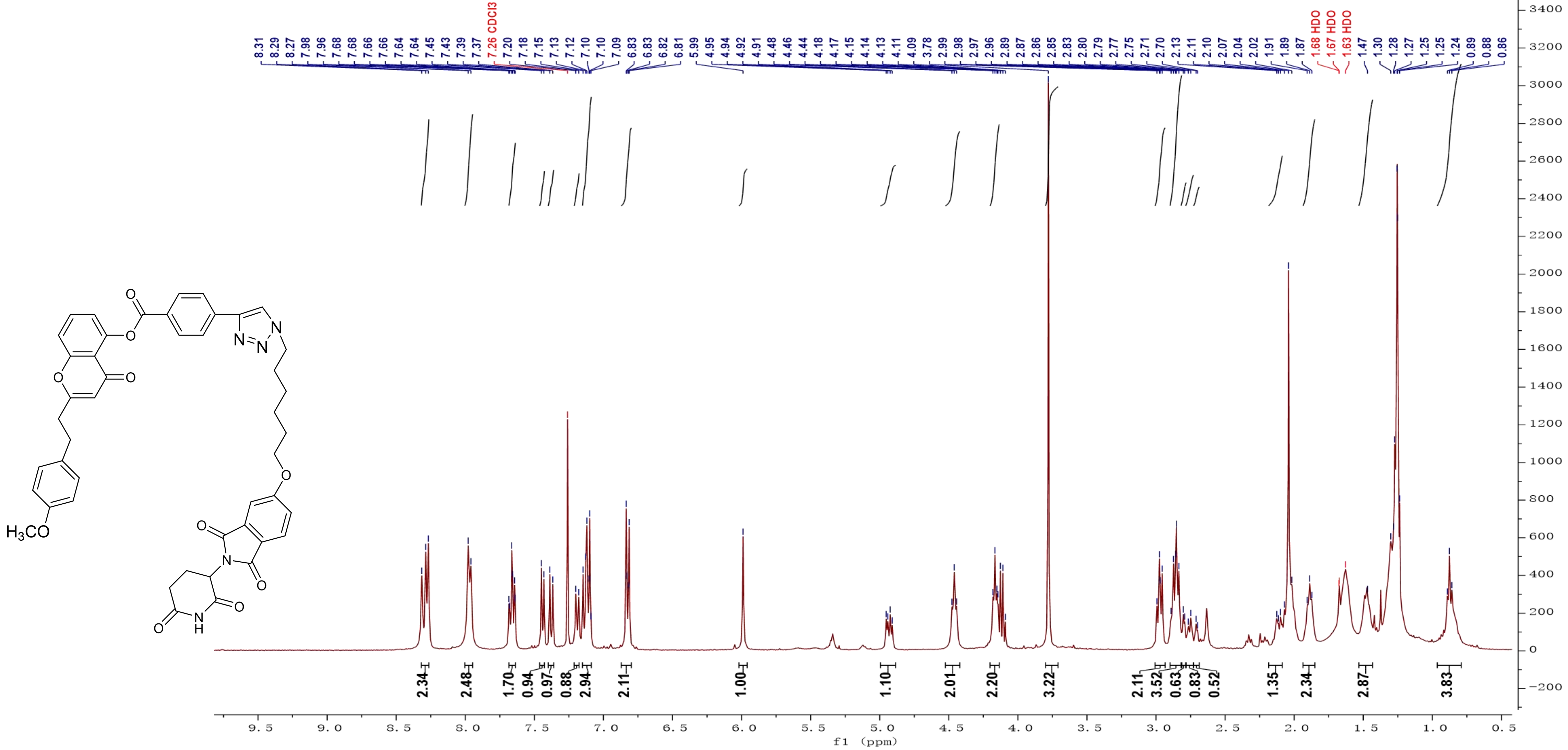

Fig. S 78 ^{1}H NMR of of 2-(4-methoxyphenethyl)-4-oxo-4H-chromen-5-yl 4-(1-(6-((2-(2,6-dioxopiperidin-3-yl)-1,3-dioxoisoindolin-5-yl)oxy)hexyl)-1H-1,2,3-triazol-4-yl)benzoate (400MHz, $CDCl_3$)

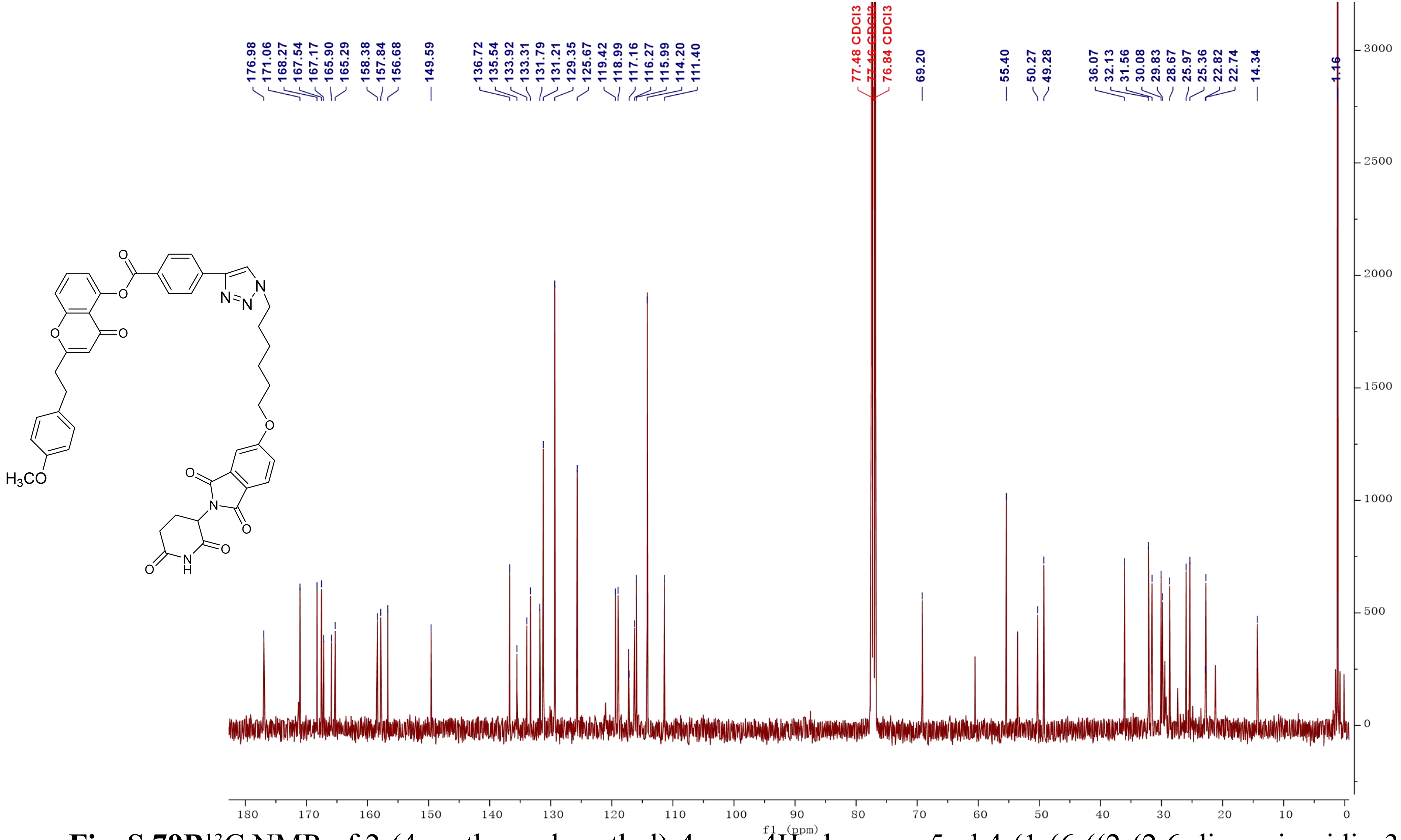

Fig. S 79B ^{13}C NMR of 2-(4-methoxyphenethyl)-4-oxo-4H-chromen-5-yl 4-(1-(6-((2-(2,6-dioxopiperidin-3-yl)-1,3-dioxoisoindolin-5-yl)oxy)hexyl)-1H-1,2,3-triazol-4-yl)benzoate (100MHz, $CDCl_3$)

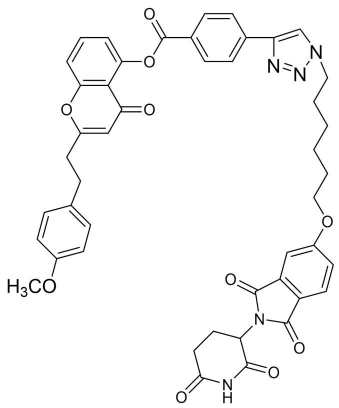

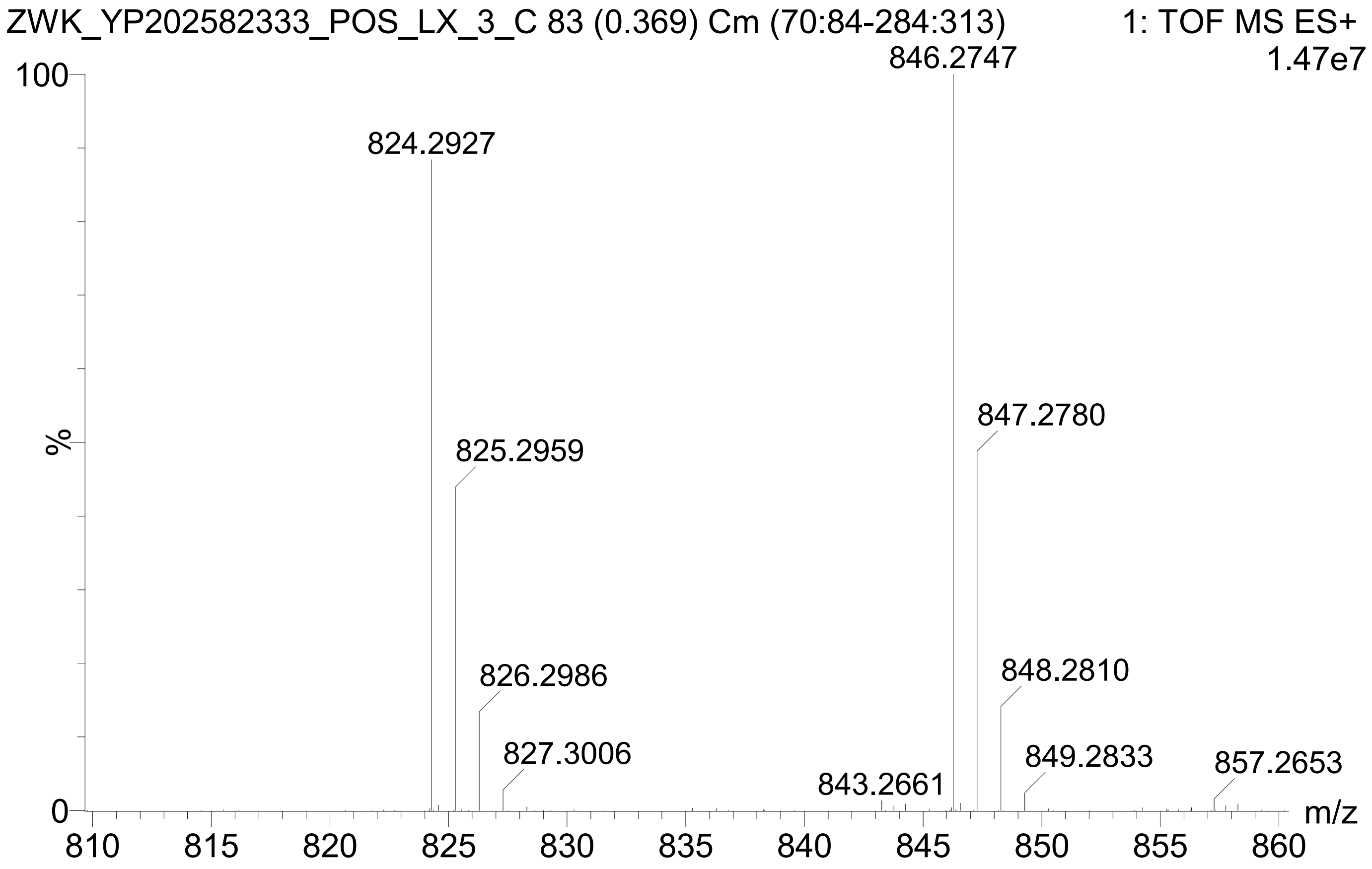

Fig. S 80 ESI(+)-HRMS of 2-(4-methoxyphenethyl)-4-oxo-4H-chromen-5-yl 4-(1-(6-((2-(2,6-dioxopiperidin-3-yl)-1,3-dioxoisoindolin-5-yl)oxy)hexyl)-1H-1,2,3-triazol-4-yl)

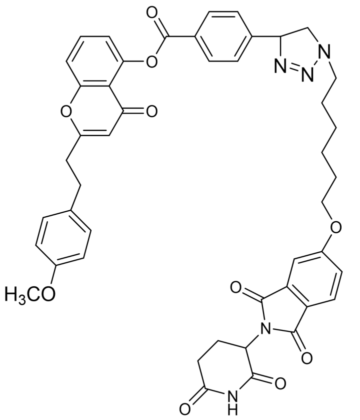

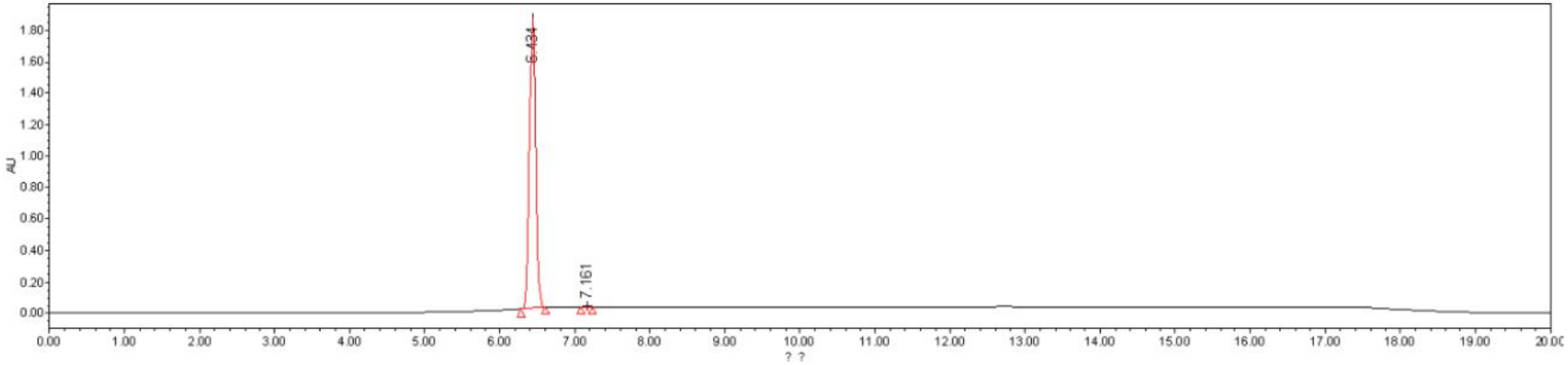

		Retention time	Area	Area ratio (%)
1		6.434	10560261	99.77
2		7.161	24539	0.23

Fig. S 81 HPLC of 2-(4-methoxyphenethyl)-4-oxo-4H-chromen-5-yl 4-(1-(6-((2-(2,6-dioxopiperidin-3-yl)-1,3-dioxoisoindolin-5-yl)oxy)hexyl)-4,5-dihydro-1H-1,2,3-triazol-4-yl)benzoate (R.T.=6.434, purity=99.77%)

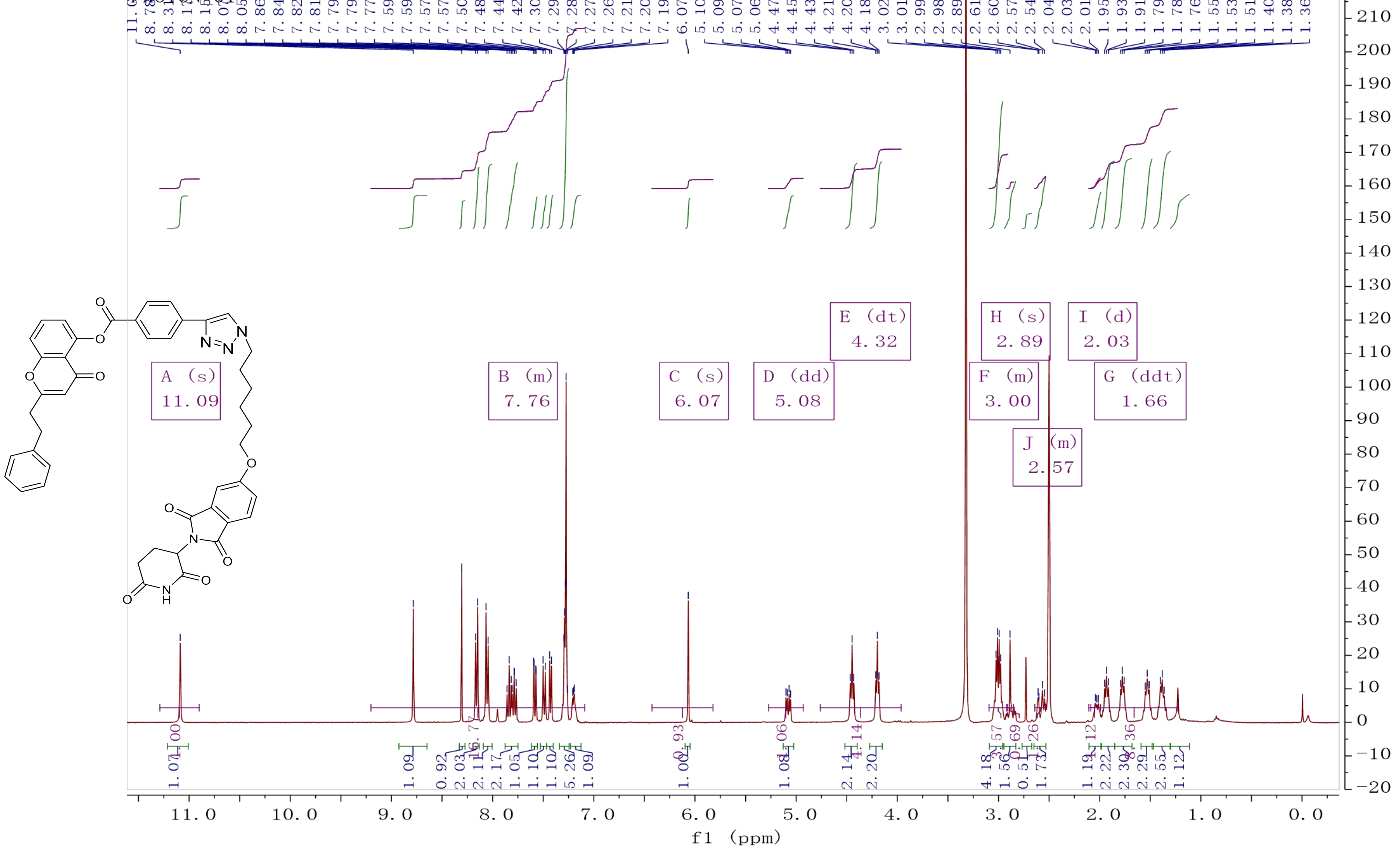

Fig. S 82 ¹H NMR of 4-oxo-2-phenethyl-4H-chromen-5-yl 4-(1-(6-((2-(2,6-dioxopiperidin-3-yl)-1,3-dioxoisoindolin-5-yl)oxy)hexyl)-1H-1,2,3-triazol-4-yl)benzoate (400MHz, DMSO-$d6$)

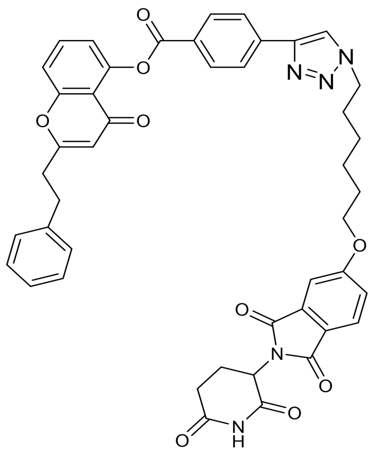

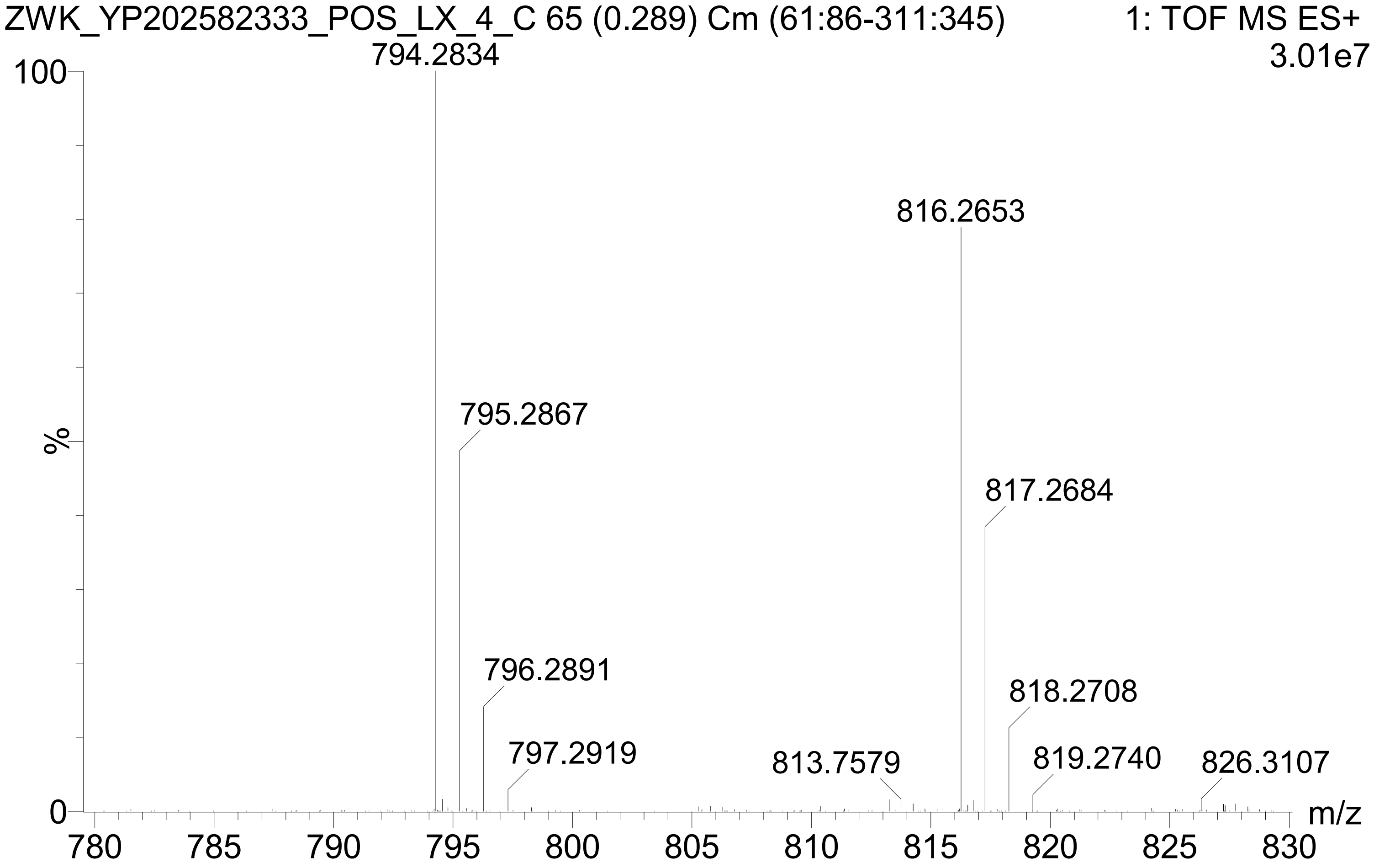

Fig. S 83 ESI(+)-H RMS of 4-oxo-2-phenethyl-4H-chromen-5-yl 4-(1-(6-((2-(2,6-dioxopiperidin-3-yl)-1,3-dioxoisoindolin-5-yl)oxy)hexyl)-4,5-dihydro-1H-1,2,3-triazol-4-yl)benzoate

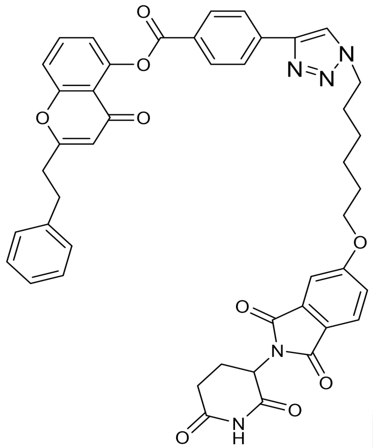

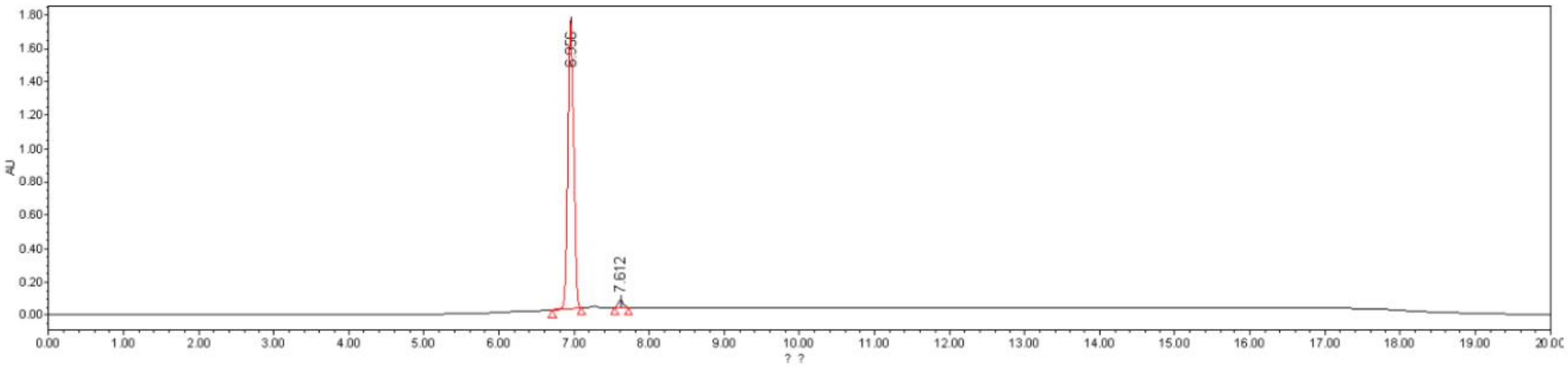

		Retention time	Area	Area ratio (%)
1		6.956	9489193	97.56
2		7.612	237734	2.44

Fig. S 84 HPLC of 4-oxo-2-phenethyl-4H-chromen-5-yl 4-(1-(6-((2-(2,6-dioxopiperidin-3-yl)-1,3-dioxoisoindolin-5-yl)oxy)hexyl)-4,5-dihydro-1H-1,2,3-triazol-4-yl)benzoate (R.T.=6.956 min; purity=97.56%)

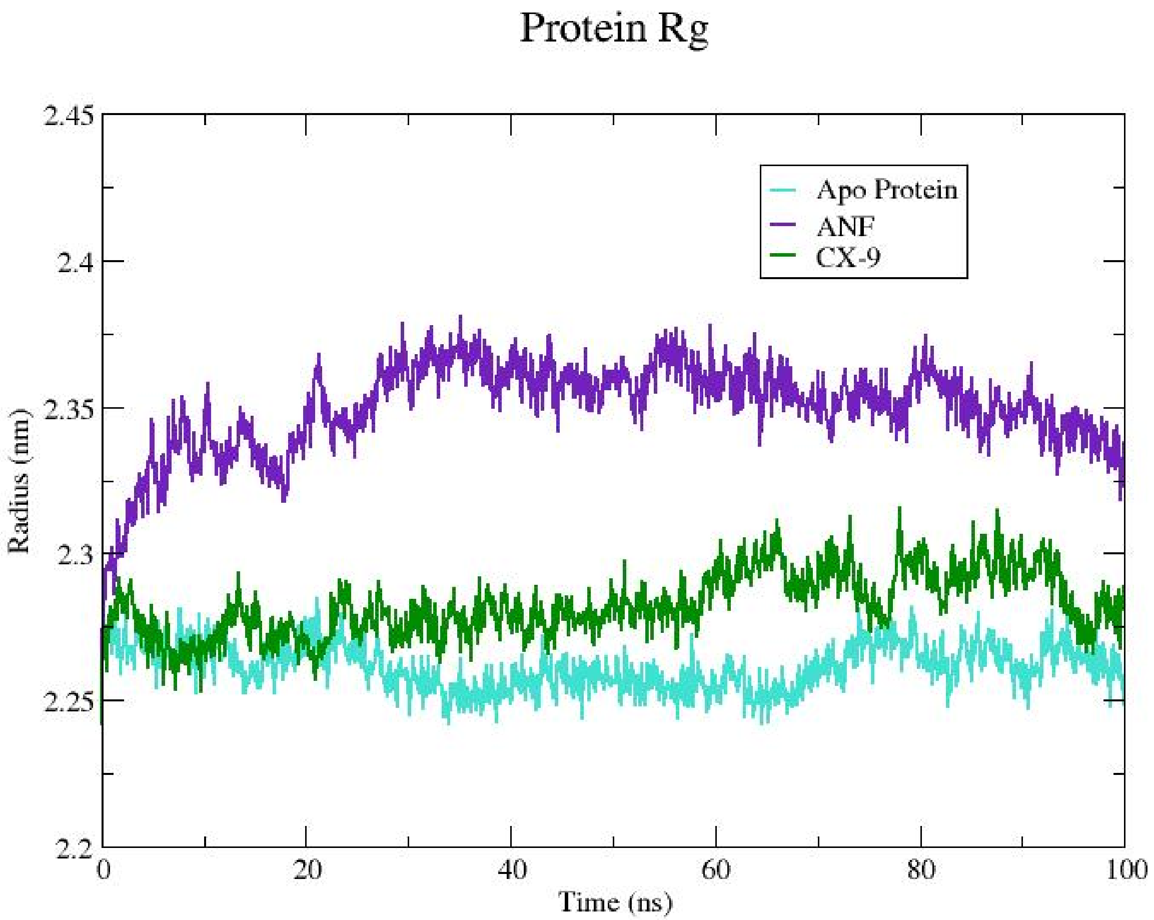

ANF shows a modest expansion relative to apo and CX-9

Fig. S85 : Time-evolution of Rg for protein compactness infulenced by **ANF** and **CX-9**, respectively.

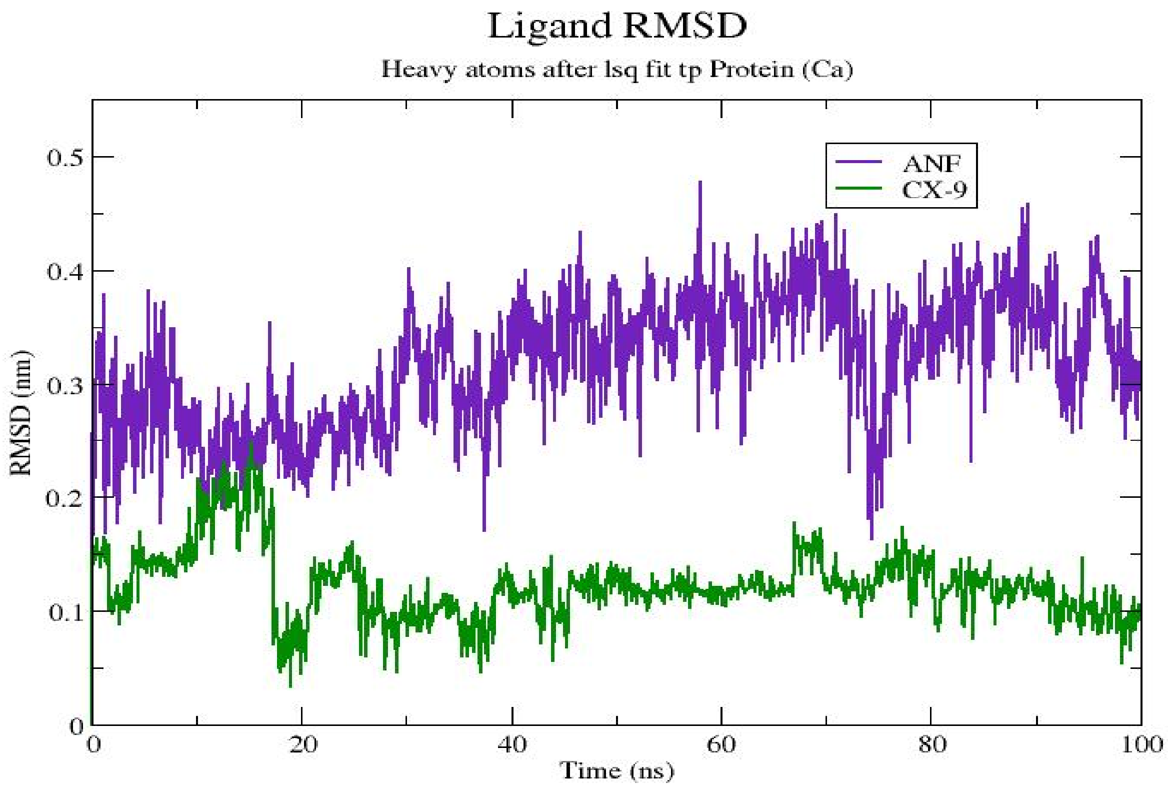

CX-9 maintains a lower and more stable RMSD than ANF over 100 ns.

Fig. S86. Ligand RMSD, heavy-atom RMSD of ANF and **CX-9** relative to the first bound frame after least-squares fit to protein Cα.

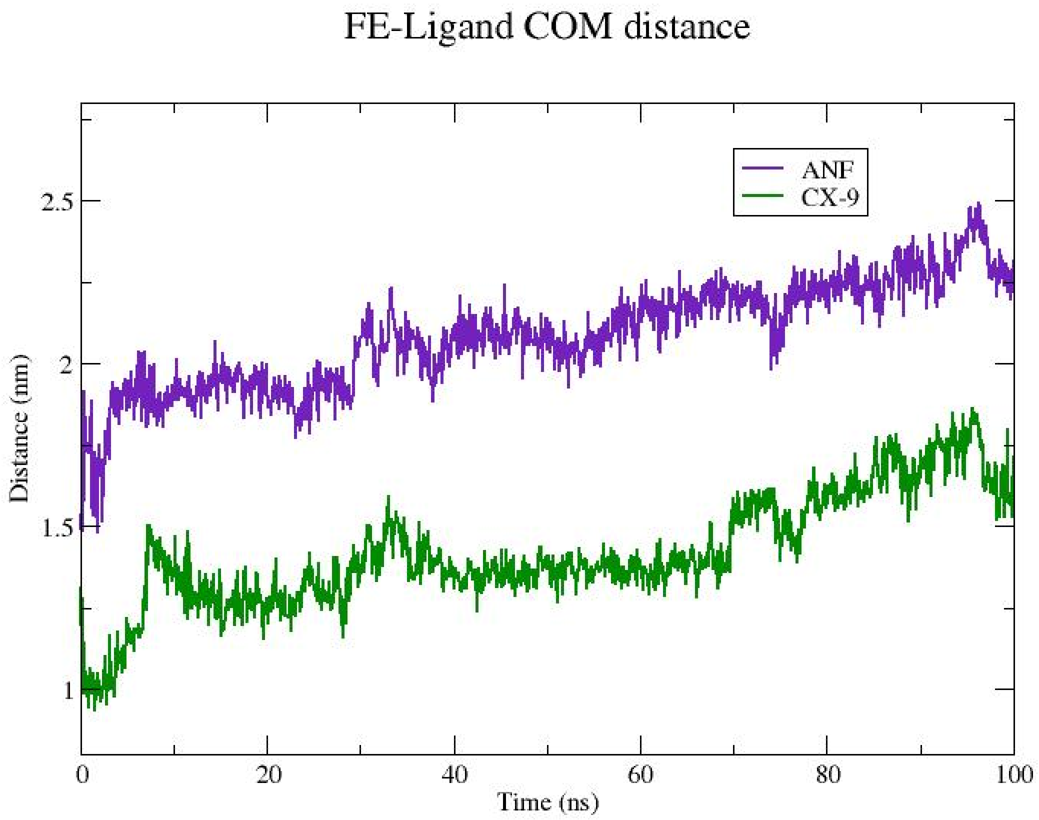

CX-9 remains consistently closer to Fe than ANF, indicating deeper, more stable engagement of the catalytic pocket.

Fig. S87 Fe–ligand COM distance, Tim**CX-9**e evolution of the distance between the heme Fe atom and the ligand heavy-atom COM of ANF and , respectively.
